# Copper-Based Targeted Nanocatalytic Therapeutics for Non-Small Cell Lung Cancer

**DOI:** 10.1007/s40820-025-01998-5

**Published:** 2026-01-12

**Authors:** Yongfei Fan, Jiao Chang, Xichun Qin, Meng Li, Yan Li, Leilei Wu, Kun Li, Zhimin Chen, Yani Li, Zhongmin Tang, Dong Xie, Jianlin Shi

**Affiliations:** 1 Department of Thoracic Surgery, Shanghai Pulmonary Hospital, School of Medicine, Tongji University, Shanghai, 200433 People’s Republic of China; 2 Department of Radiology, Tongji Hospital, School of Medicine, Tongji University, Shanghai, 200331 People’s Republic of China; 3 Shanghai Frontiers Science Center of Nanocatalytic Medicine, School of Medicine, Tongji University, Shanghai, 200331 People’s Republic of China; 4 Department of Thoracic Surgery, Zhejiang Cancer Hospital, Hangzhou Institute of Medicine, Chinese Academy of Sciences, Hangzhou, Zhejiang 310022 People’s Republic of China; 5 Department of Medical Oncology, Shanghai Pulmonary Hospital, School of Medicine, Tongji University, Shanghai, 200433 People’s Republic of China; 6 Shanghai Institute of Ceramics Chinese Academy of Sciences, Research Unit of Nanocatalytic Medicine in Specific Therapy for Serious Disease, Chinese Academy of Medical Sciences, Shanghai, 200050 People’s Republic of China

**Keywords:** Nanocatalytic medicine, Reactive oxygen species, Lung cancer therapy, Copper-based nanoparticles

## Abstract

Conventional treatments for non-small cell lung cancer (NSCLC) suffer from low remission rates, high drug resistance, and severe adverse effects. To leverage the therapeutic potential of reactive oxygen species (ROS), nanocatalytic medicine utilizes nanomaterials to generate ROS specifically within tumor sites, enabling efficient and targeted cancer treatment. In this study, hyaluronic acid (HA)-modified copper (Cu) and dimercaptosuccinic acid (DMSA) assembled nanoparticles (Cu-DMSA-HA NPs) are developed with tumor-targeting capability and efficiently catalyze ROS production via coordination chemistry. Targeted delivery is facilitated by HA surface modification through recognition of overexpressed cluster of differentiation 44 receptors on cancer cells, which enhances nanoparticle uptake. Once internalized, intracellular glutathione is depleted by the NPs, followed by a Fenton-like reaction that sustains ROS production. Both in vitro and in vivo studies demonstrate that this catalytic strategy effectively inhibits DNA replication, prevents cell cycle progression, downregulates glutathione peroxidase 4 expression, induces ferroptosis, and ultimately suppresses NSCLC progression. Overall, the readily prepared Cu-DMSA-HA NPs exhibit robust catalytic activity and tumor specificity, highlighting their strong potential for clinical translation in nanocatalytic cancer therapy.

**Supplementary Information:**

The online version contains supplementary material available at 10.1007/s40820-025-01998-5.

## Highlights

- Developed a novel type of nanoparticles (NPs)—hyaluronic acid (HA)-modified copper (Cu) and dimercaptosuccinic acid (DMSA)-assembled NPs copper (Cu) and dimercaptosuccinic acid (DMSA).
- The constructed NPs were surface-modified with HA to selectively target overexpressed cluster of differentiation 44 (CD44) receptors on cancer cells and catalytically generate highly efficient reactive oxygen species (ROS) via coordination chemistry.
- Such efficient ROS generation induced intracellular ROS accumulation, mitochondrial disruption, glutathione (GSH) depletion, and glutathione peroxidase 4 (GPX4) downregulation, ultimately triggering ferroptosis in cancer cells.

**Supplementary Information:**

The online version contains supplementary material available at 10.1007/s40820-025-01998-5.

## Graphical abstract

**Figure:**
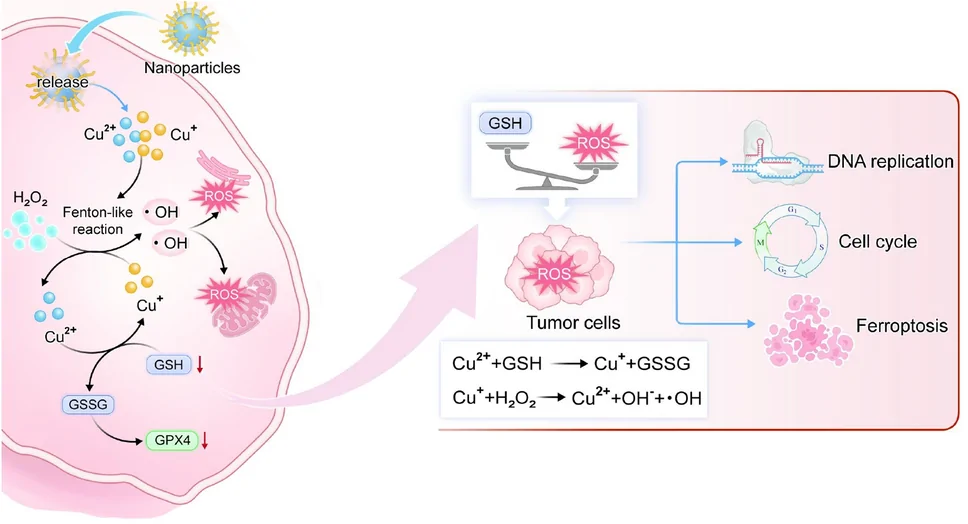

## Introduction

Lung cancer remains the leading cause of cancer-related morbidity and mortality worldwide, with an estimated 1.8 million deaths (18.7%) each year [1]. Among all subtypes, non-small cell lung cancer (NSCLC) accounts for a predominant proportion, approximately 80%–85% of all cases, and presents significant clinical challenges [1, 2]. A major challenge is that surgical resection is primarily effective for early-stage NSCLC patients, while treatment options for advanced-stage NSCLC patients largely rely on chemotherapeutic agents (e.g., cisplatin, paclitaxel, pemetrexed) and immunotherapeutic agents (e.g., pembrolizumab, nivolumab) [3–5]. However, these drugs often have limitations; for instance, chemotherapy drugs are frequently associated with a low remission rate, high drug resistance, and severe adverse effects (SAEs); immunotherapy drugs are usually accompanied by poor pathological response rates, which largely restricts their clinical effectiveness [6–8]. Therefore, developing novel therapeutic strategies is essential to improve clinical outcomes for patients with advanced-stage NSCLC.

Recent advances in materials science and nanotechnology have inspired novel therapeutic strategies by leveraging catalytic and redox-based mechanisms—such as piezocatalysis, nanocatalysis, and metal (e.g., copper)-mediated cell death, to address unmet clinical challenges [9–12]. Specifically, radiotherapy and several chemotherapeutic agents exert their therapeutic effects, in part, through the generation of reactive oxygen species (ROS) within tumor tissues, leading to oxidative damage and cell death [13–16]. Inspired by these clinical principles, nanocatalytic medicine has emerged as a promising strategy that employs nanoparticles (NPs) to initiate site-specific chemical reactions directly within the tumor microenvironment (TME), resulting in localized ROS production and enhanced therapeutic effects in situ. This approach not only offers improved treatment specificity and reduced systemic SAEs, but also leverages the favorable properties of NPs, such as extended circulation time and facile surface modification for further targeted delivery [10, 17–20]. Among nanocatalytic approaches, chemodynamic therapy (CDT) has garnered attention for its ability to exploit endogenous hydrogen peroxide (H₂O₂) in the TME to produce cytotoxic hydroxyl radicals (·OH) through Fenton or Fenton-like reactions [21–23]. Nevertheless, CDT is limited by the elevated levels of glutathione (GSH) in the tumor area, which can scavenge ROS, as well as by the low catalytic efficiency of many Fe-based NPs under mildly acidic or neutral pH conditions [22, 24, 25]. In contrast, Cu(II)-based NPs offer distinct advantages: Cu(II) ions can be readily reduced by intracellular GSH to Cu(I), which in turn drives efficient Fenton-like reactions under physiological conditions, thereby enabling sustained ROS production and enhanced therapeutic potential against cancer cells [26–29].

With all the above considered, we developed a novel type of NPs—hyaluronic acid (HA)-modified Cu and dimercaptosuccinic acid (DMSA) assembled NPs (Cu-DMSA-HA NPs), with tumor-targeting capability. These NPs leverage coordination chemistry to catalytically generate ROS with high efficiency. Surface modification with HA enables selective targeting of overexpressed cluster of differentiation 44 (CD44) receptors on cancer cells, facilitating NP uptake and subsequent catalytic reactions. This occurs through an initial reduction of GSH, followed by Fenton-like reaction that drives efficient ROS production for cancer cell therapy. Moreover, both in vitro and in vivo experiments—including subcutaneous and orthotopic lung tumor models—demonstrated that Cu-DMSA-HA NPs promoted intracellular ROS accumulation, mitochondrial disruption, GSH depletion, and downregulation of glutathione peroxidase 4 (GPX4) expression levels, ultimately triggering ferroptosis in cancer cells. Inspired by the promising therapeutic outcomes demonstrated in this study, we believe that nanocatalytic medicine represents a compelling strategy for NSCLC treatment and holds strong potential to bridge clinical challenges with innovative nanotechnological solutions, ultimately contributing to improving patient outcomes.

## Experimental Section

See the Supporting Information for the experimental details.

## Results and Discussion

### Preparation and Characterization of Cu-DMSA-HA NPs

**Fig. 1:**
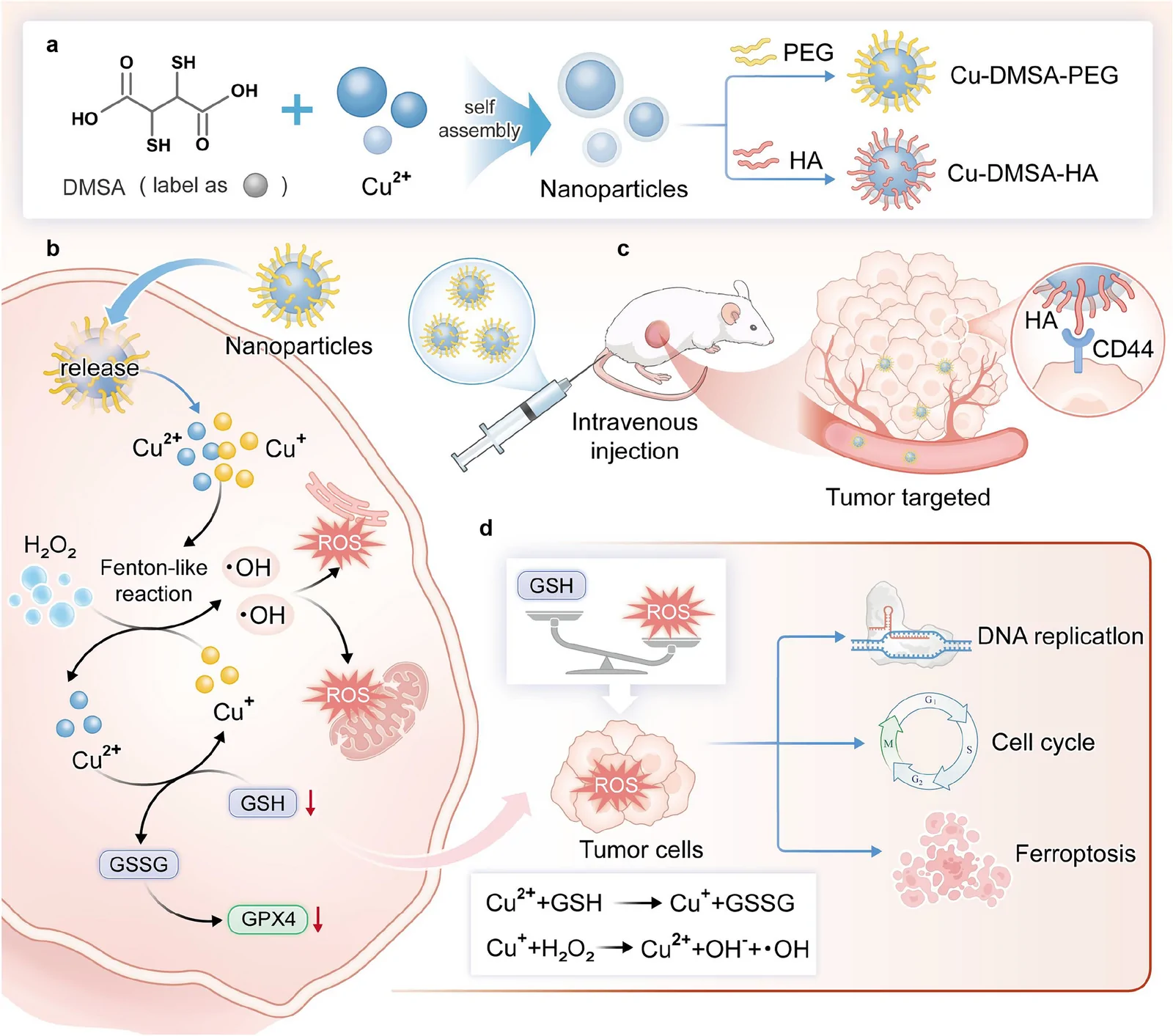
Preparation schematics of nanoparticles (NPs) and proposed therapeutic mechanism. **a** Schematic illustration of the synthesis of copper (Cu) and dimercaptosuccinic acid (DMSA) NPs modified with polyethylene glycol (Cu-DMSA-PEG NPs) and hyaluronic acid (HA) (Cu-DMSA-HA NPs), respectively. **b** Proposed biological mechanism of Cu-DMSA-HA-induced ferroptosis in tumor cells. Following cellular internalization, Cu-DMSA-HA NPs, which are assembled via coordination chemistry, catalyze intracellular reactive oxygen species (ROS) generation through glutathione (GSH)-mediated reduction and subsequent Fenton-like reactions. The resulting ROS accumulation triggers oxidative damage to mitochondria and the endoplasmic reticulum of tumor cells, leading to downregulation of glutathione peroxidase 4 (GPX4), a key regulator protecting against ferroptosis. **c** Mechanism of in vivo tumor targeting by Cu-DMSA-HA. Cu-DMSA-HA facilitates selective binding to cluster of differentiation 44 (CD44) receptors overexpressed on tumor cells, enhancing tumor-specific accumulation. **d** Antitumor mechanism of Cu-DMSA-HA in vivo. Cu-DMSA-HA inhibits tumor progression by suppressing DNA replication and cell cycle progression while inducing ferroptosis

**Fig. 2:**
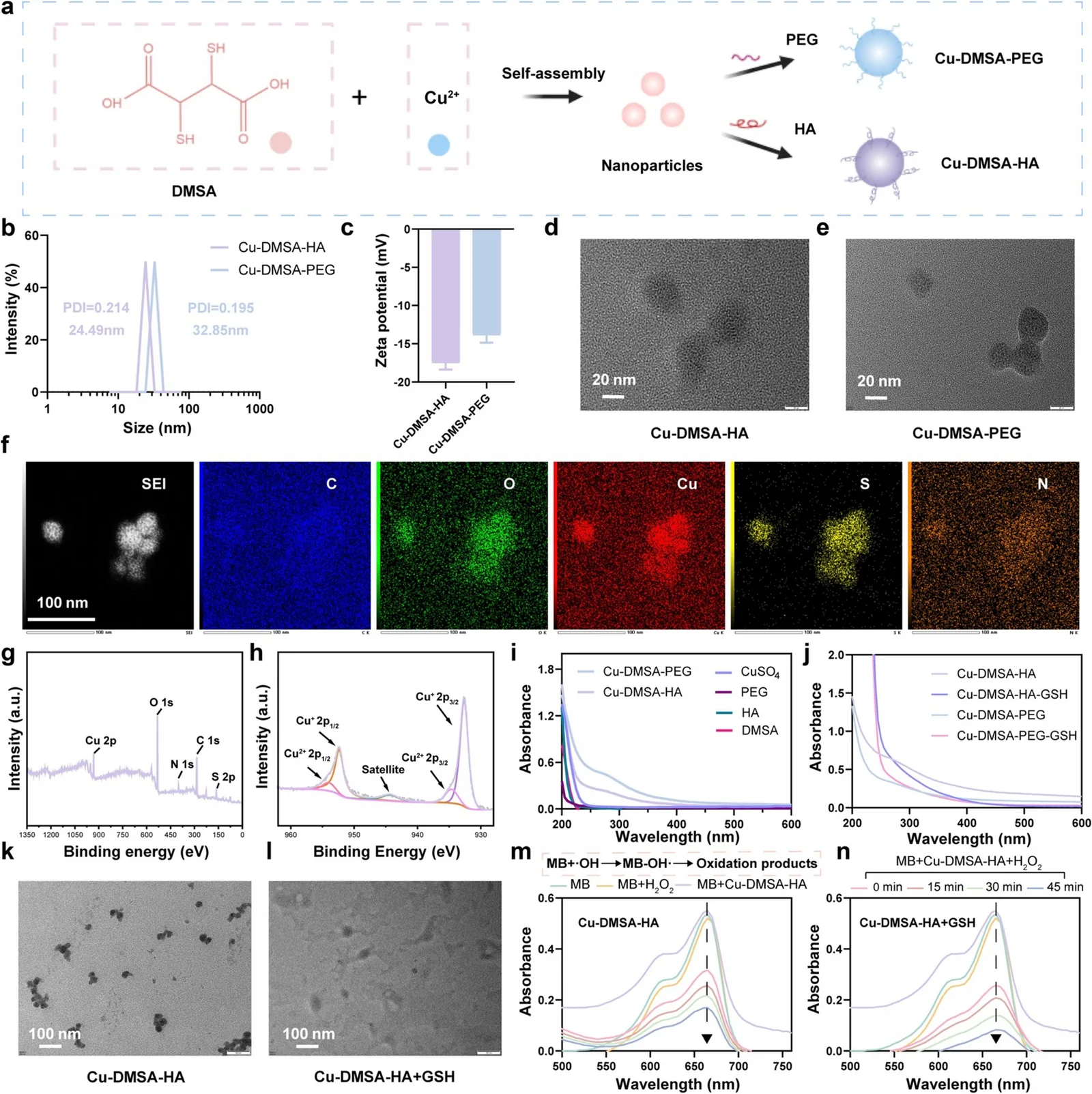
Preparation procedures and characterization of Cu-DMSA-HA NPs. **a** Schematic illustration of the construction for Cu-DMSA-HA. **b** Particle size distribution and **c** zeta potential of NPs. **d** Transmission electron microscopy (TEM) images of Cu-DMSA-HA and **e** Cu-DMSA-PEG. Scale bar: 20 nm. **f** High-angle annular dark-field scanning transmission electron microscopy (HAADF-STEM) image and elemental mapping images of the elements in the Cu-DMSA-HA. Scale bar: 100 nm. The X-ray photoelectron spectroscopy (XPS) survey scan **g** and Cu 2*p* scan **h** of Cu-DMSA-HA. **i** Ultraviolet–visible (UV–Vis) absorption spectra of CuSO_4_, DMSA, Cu-DMSA-PEG and Cu-DMSA-HA. **j** UV–Vis absorption spectra of Cu-DMSA-HA and Cu-DMSA-PEG before and after GSH addition. TEM images of **k** Cu-DMSA-HA and **l** Cu-DMSA-HA after GSH addition Scale bar: 100 nm. Time-dependent UV–Vis spectra of methylene blue (MB) degradation catalyzed by **m** Cu-DMSA-HA and **n** Cu-DMSA-HA + GSH

In the present study, we synthesized a novel type of NPs by utilizing the coordination interactions between copper and the chemical groups on dimercaptosuccinic acid (DMSA), as illustrated in Fig. 2a. In order to screen the optimal size and dispersion of NPs, we conducted synthesis reactions using CuSO_4_ in combination with DMSA at ratios of 2:1, 1:1, 1:2, and 1:4. Interestingly, we found that when the concentration of copper ions was excessive, the stability of the solution significantly decreased, and the hydrodynamic size reached the micron level. Pronounced aggregation and precipitation were observed after one day of synthesis, as shown in Fig. 1b. However, at the 1:1 ratio, we detected well-dispersed NPs with a particle size of approximately 18 nm (Fig. S1a). When DMSA was in excess, at the 1:2 ratio, the solution gradually changed from yellow to black, forming NPs with a higher degree of polymerization with slightly yellowish-black color. The zeta potential was close to being electrically neutral. This may be due to excessive DMSA leading to partial degradation of the material (1:4 ratio), no distinct NPs were detected. Transmission electron microscopy (TEM) analysis revealed that the particles were in an amorphous state (Fig. S2). This observation further indicates that the excess, highly reducing DMSA decomposes the previously formed NPs. In conclusion, we chose the 1:1 ratio for NP synthesis, and to improve both targeting specificity and stability, the NPs were subsequently modified with hyaluronic acid (Cu-DMSA-HA) and polyethylene glycol (Cu-DMSA-PEG), respectively.

As shown in Fig. 2b, dynamic light scattering (DLS) results revealed that the hydrodynamic size of Cu-DMSA-HA was approximately 24.49 nm, while that of Cu-DMSA-PEG measured around 32.85 nm. The zeta potential of the Cu-DMSA-HA was about − 17.46 ± 0.61 mV (Fig. 2c), compared to approximately − 13.8 ± 0.70 mV for Cu-DMSA-PEG. The difference in zeta potential between Cu-DMSA-PEG and Cu-DMSA-HA NPs is likely attributed to two factors: the higher copper ion content in Cu-DMSA-PEG and the intrinsic negative charge of HA in Cu-DMSA-HA. Inductively coupled plasma optical emission spectrometry (ICP-OES) analysis confirmed the copper loading content, which was calculated to be approximately 29.8157% for Cu-DMSA-HA and 38.7041% for Cu-DMSA-PEG (Table S1). TEM was employed to visualize the morphology and dimensions of the nanostructures, as shown in Fig. 2d, e. TEM images revealed that the NPs exhibited elliptical morphology with an average diameter of approximately 25 nm, consistent with the DLS measurements. Additionally, elemental mapping images clearly demonstrated the uniform distribution of carbon (C), oxygen (O), nitrogen (N), sulfur (S), and copper (Cu) within the Cu-DMSA-HA NPs, as depicted in Figs. 2f and S3. Complementary X-ray photoelectron spectroscopy (XPS) full-spectrum analysis further confirmed the successful integration of all elements, supporting the effective synthesis of the nanomaterial (Figs. 2g and S4a).

Notably, the ultraviolet–visible (UV–Vis) absorption spectra of Cu-DMSA-HA exhibited a characteristic peak at about 280 nm, which was distinct from the spectrum of individual CuSO₄ and DMSA (Fig. 2i). This peak likely arises from the formation of some new coordination structures. Furthermore, XPS analysis of the Cu 2*p* region (Figs. 2h and S4b) revealed that copper species within the nanostructure predominantly existed as Cu(I) (accounting for 84.72% of the total Cu content), with Cu(II) comprising 15.28%. This distribution is attributed to the strong reducing properties of DMSA. The high concentration of Cu(I) is favorable for enhancing the catalytic activity of Cu-DMSA-HA in Fenton-like reactions. Besides, the stability of Cu-DMSA-HA and Cu-DMSA-PEG NPs in PBS was detected in different times. The NPs can be stored in a powder form for a long time after freeze-drying, and they can remain relatively stable in solution around 48 h (Fig. S6).

To evaluate the catalytic generation of ROS, methylene blue (MB), a sensitive ROS indicator, was employed to assess the Fenton-like reaction between Cu and H_2_O_2_. ·OH generated from the reaction degraded MB, leading to a decrease in its absorbance at 664 nm. As depicted in Fig. 2m, neither H₂O₂ alone nor Cu-DMSA-HA in the absence of H₂O₂ exhibited significant MB degradation. In contrast, the addition of H₂O₂ to Cu-DMSA-HA led to rapid MB degradation, showing a time-dependent increase in degradation efficiency over the reaction period. The results of electron paramagnetic resonance (EPR) spectroscopy also showed that Cu-DMSA-HA can rapidly generate a large number of hydroxyl radicals in a short period of time (Fig. S7). More importantly, we found that the addition of GSH further accelerated the MB degradation process, indicating a tandem reaction between Cu-DMSA and GSH that promoted NP disassembly and enhanced the generation of ROS (Fig. 2n). This enhancement is attributed to redox cycling between Cu-DMSA-HA and GSH, which amplifies ROS production and facilitates the breakdown of Cu-DMSA-HA.

Moreover, the GSH-responsive degradation capability of the NPs was further corroborated by the disappearance of a characteristic absorbance peak at 280 nm upon the addition of GSH (Fig. 2j). This disappearance signifies the decomposition of the NP structure, indicating its sensitivity to the reductive intracellular environment. As shown in Fig. 2k, l, TEM images also showed the disassembly of Cu-DMSA-HA NPs in the presence of GSH. Furthermore, Cu-DMSA-HA exhibited greater efficiency for ROS generation in an acidic environment (pH 5.5) (Fig. S8). The pH-dependent enhancement may arise from the dissociation of coordination bonds in the acidic environments, which are stable at neutral pH.

### Cu-DMSA-HA NPs Target NSCLC Cells and Induce Apoptosis

**Fig. 3:**
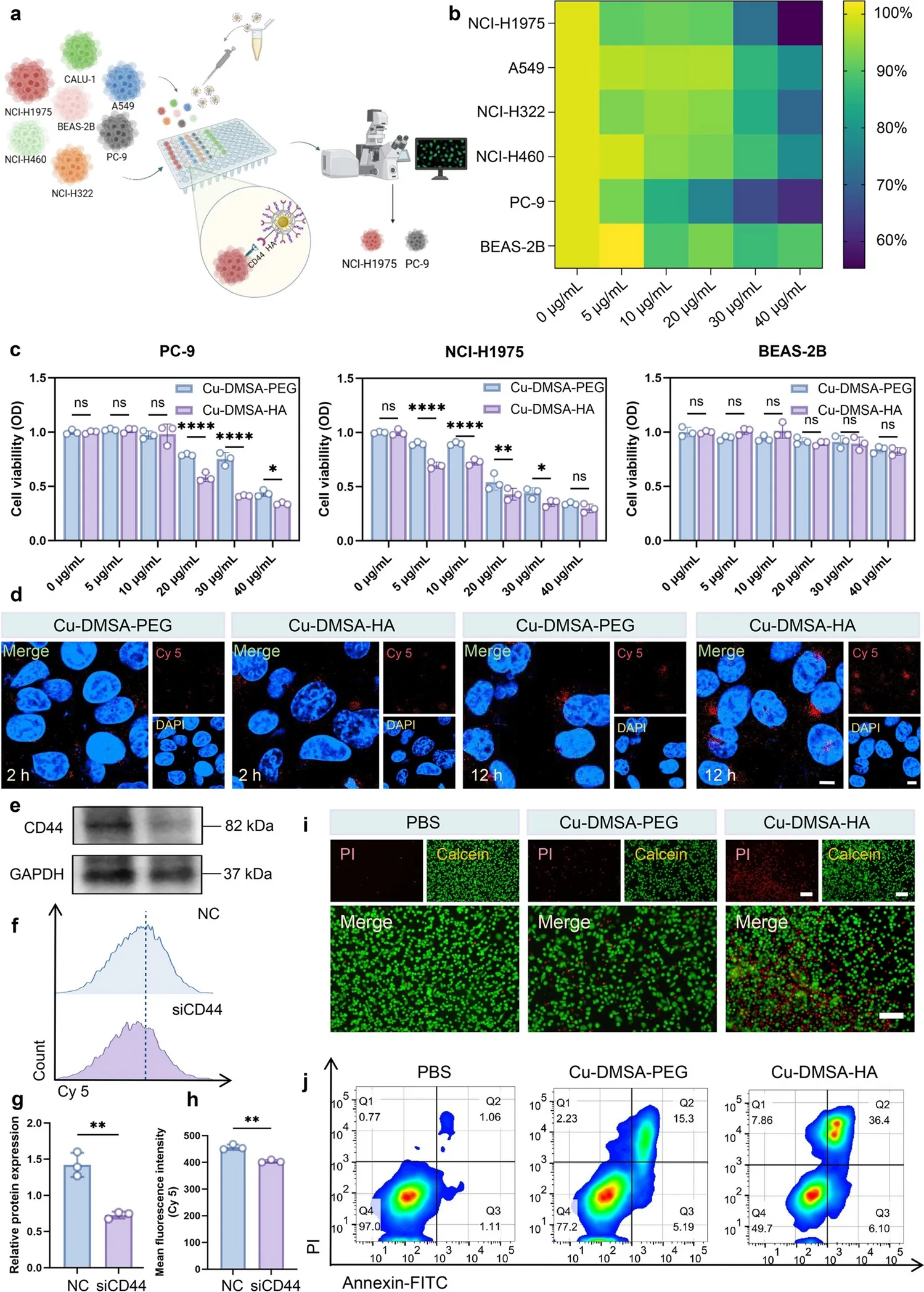
Evaluation of the in vitro targeting capability of Cu-DMSA-HA NPs. **a** Schematic diagram illustrating the experimental design for the targeting of Cu-DMSA-HA NPs to NSCLC cells. **b** Cell counting kit-8 (CCK-8) assay evaluating the cell viability of five NSCLC cell lines (PC-9, NCI-H460, NCI-H322, A549, and NCI-H1975) and one normal lung epithelial cell line (BEAS-2B) following Cu-DMSA-HA treatment at different concentrations. **c** CCK-8 assay assessing cell viability of PC-9, NCI-H1975, and BEAS-2B cells after treatment with various concentrations of Cu-DMSA-PEG or Cu-DMSA-HA (*n* = 3; mean ± standard deviation [SD]). Student’s t-test (unpaired, two-tailed). **d** Confocal laser scanning microscopy (CLSM) comparing cellular uptake of Cu-DMSA-PEG and Cu-DMSA-HA NPs in PC-9 cells at 2- and 12-h post-treatment. Red fluorescence: Cy5-labeled Cu-DMSA-PEG or Cu-DMSA-HA NPs; blue fluorescence: cell nuclei. Scale bar: 10 μm. **e** Western blot analysis and **g** corresponding quantitative analysis of CD44 protein expression in PC-9 cells treated with CD44 siRNA (siCD44) or negative control (N**C** (*n* = 3; mean ± SD). Student’s t-test (unpaired, two-tailed). **f** Flow cytometry analysis and **h** corresponding quantitative analysis of Cu-DMSA-HA uptake in PC-9 cells treated with siCD44 or NC (n = 3; mean ± SD). Student’s t-test (unpaired, two-tailed). **i** Live/dead staining comparing cell viability of PC-9 cells treated with phosphate-buffered saline (PBS), Cu-DMSA-PEG, or Cu-DMSA-HA. Red fluorescence: propidium iodide; green fluorescence: calcein-AM. Scale bar: 100 μm. **j** Annexin V-FITC apoptosis analysis via flow cytometry in PC-9 cells treated with PBS, Cu-DMSA-PEG, or Cu-DMSA-HA. *P < 0.05; **P < 0.01; ***P < 0.001; ****P < 0.0001; ns, not statistically significant

Previous studies have demonstrated that HA binds with high affinity to CD44 receptors [30–32]. The aberrant overexpression of CD44 in cancer cells is closely associated with malignant biological behaviors (e.g., invasion, metastasis) [33–35]. Building upon this foundation, we assessed the differential cytotoxic effects of Cu-DMSA-HA NPs between NSCLC cells and normal epithelial cells (Fig. 3a). Cytotoxicity heatmap analysis revealed that the Cu-DMSA-HA NPs treatment (5, 10, 20, 30, and 40 μg mL^−1^) induced a dose-dependent reduction in cell viability across the five NSCLC cell lines (PC-9, NCI-H460, NCI-H322, A549, and NCI-H1975), demonstrating their broad-spectrum antitumor activity (Fig. 3b). Notably, PC-9 and NCI-H1975 exhibited the highest sensitivity to Cu-DMSA-HA treatment at a concentration of 40 μg mL^−1^ (Fig. 3b), suggesting a potent cytotoxic response. In contrast, the normal human bronchial epithelial cell line (BEAS-2B) maintained high cell viability under the same conditions, highlighting the selective antitumor effect of Cu-DMSA-HA NPs (Fig. 3b). Consistently, both Cu-DMSA-HA and Cu-DMSA-PEG NPs induced concentration-dependent cytotoxicity on the growth of cancer cells (PC-9 and NCI-H1975; Fig. 3c). More importantly, at equivalent concentrations, Cu-DMSA-HA exhibited stronger inhibitory effects on the growth of cancer cells (PC-9 and NCI-H1975) compared to Cu-DMSA-PEG, while showing minimal cytotoxicity toward BEAS-2B cells (Fig. 3c). These results indicate that both Cu-DMSA-HA NPs and Cu-DMSA-PEG NPs possess favorable biosafety profiles. However, Cu-DMSA-HA NPs demonstrate superior tumor-selective cytotoxicity compared to Cu-DMSA-PEG NPs, suggesting enhanced efficiency in targeting and inhibiting tumor cell growth while sparing normal cells. This highlights Cu-DMSA-HA NPs’ potential as a safer and more effective therapeutic agent for NSCLC treatment.

To balance antitumor efficacy with epithelial cell safety and to distinguish between the activities of Cu-DMSA-HA and Cu-DMSA-PEG NPs, a 30 μg mL^−1^ concentration was selected for subsequent experiments.

Confocal laser scanning microscopy (CLSM) images showed significantly higher peritumoral accumulation of Cu-DMSA-HA compared to Cu-DMSA-PEG after 2- and 12-h incubation periods, respectively (Fig. 3d). This differential efficacy highlighted the tumor-selective targeting advantage conferred by HA-mediated CD44 recognition. Knockdown of CD44 expression in NSCLC cells using small interfering ribonucleic acid (siRNA) was confirmed by Western blot analysis, which showed a significant reduction in CD44 protein levels (*P* < 0.05; Fig. 3e, g). Subsequent flow cytometry analysis showed that Cu-DMSA-HA uptake was significantly reduced in PC-9 cells with CD44 knockdown compared to the control group (*P* < 0.05; Fig. 3f, h), indicating that Cu-DMSA-HA targeted the lung cancer cells via CD44 on the cell surface. Live/dead cell staining analysis revealed a higher proportion of dead PC-9 cells in the Cu-DMSA-HA treatment group compared to the Cu-DMSA-PEG group (Fig. 3i). These observations were further confirmed by flow cytometry analysis, which showed a significant increase in both early and late apoptotic cell populations in the Cu-DMSA-HA-treated group compared to the Cu-DMSA-PEG group (Fig. 3j). These findings demonstrate that Cu-DMSA-HA exhibited superior antitumor activity compared to Cu-DMSA-PEG. Interestingly, ICP-OES revealed lower copper ion content in Cu-DMSA-HA, yet it exhibited stronger antitumor effects (Table S1). This further demonstrates the antitumor targeting advantage of Cu-DMSA-HA and indicating that Cu-DMSA-HA is likely to achieve enhanced cellular uptake due to its strong selective targeting capability toward cancer cells, thereby enhancing its pro-apoptotic efficacy in NSCLC cells.

### Mechanism of Cu-DMSA-HA-Induced Apoptosis in NSCLC Cells

**Fig. 4:**
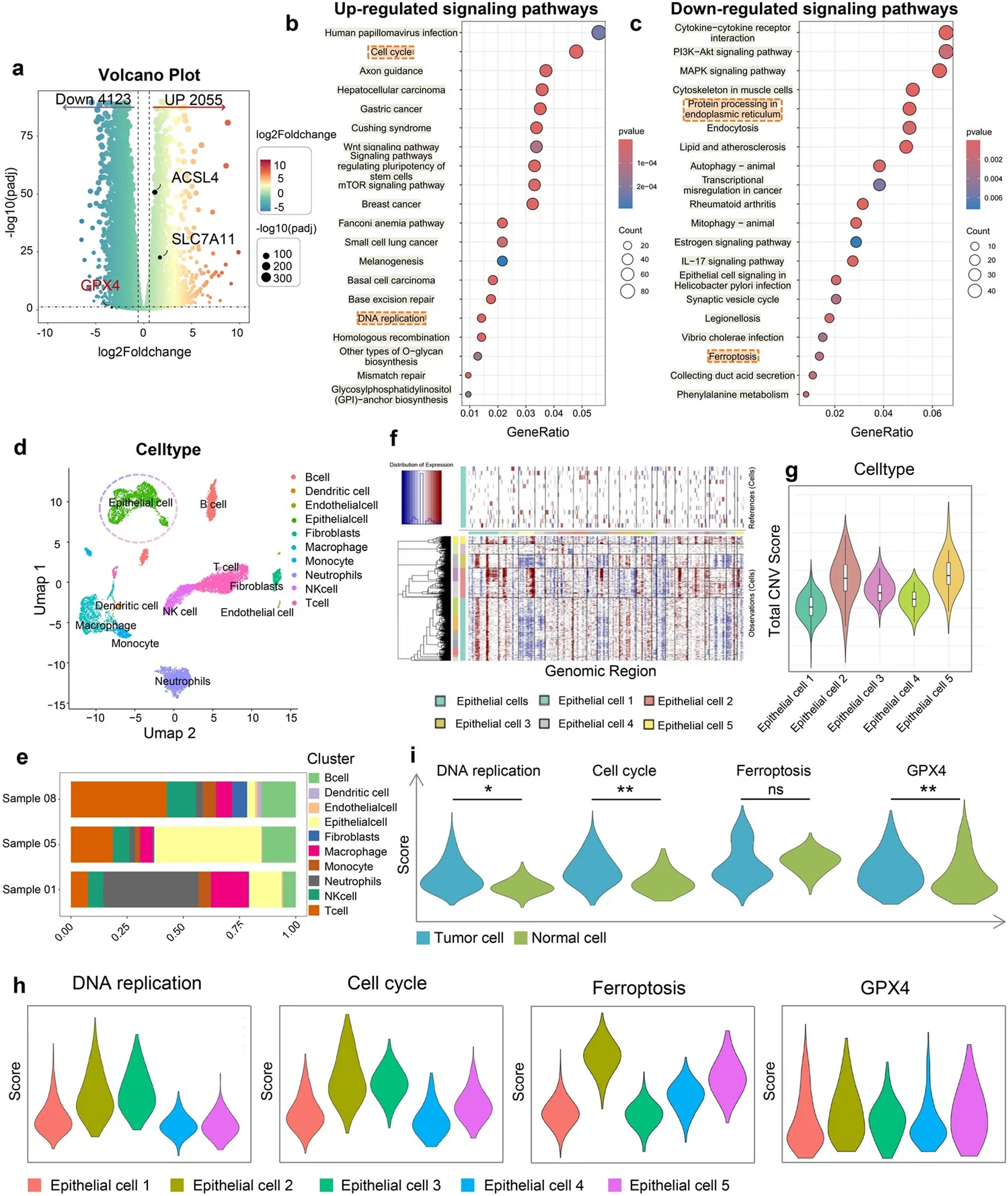
Integrated transcriptomic RNA sequencing (RNA-seq) and single-cell RNA sequencing (scRNA-seq) analysis revealing the antitumor mechanisms of Cu-DMSA-HA. **a** Volcano plot illustrating the distribution of differentially expressed genes (DEGs) in PC-9 cells treated with Cu-DMSA-HA compared to the PBS. Bubble plots depicting the Kyoto Encyclopedia of Genes and Genomes (KEGG) pathway enrichment analyses of **b** downregulated and **c** upregulated DEGs in the treatment group versus the control. **d** UMAP visualization of cell clusters obtained from dimensionality reduction and clustering analysis of scRNA-seq data from NSCLC patient tumor tissues. Each color representing a distinct cell population. **e** Bar graph depicting the distribution of various cell clusters across different NSCLC patient samples. **f** InferCNV algorithm (inferCNV) analysis demonstrating copy number variations (CNVs) among the different epithelial cell subclusters, using endothelial cells as the normal cell reference. **g** Violin plots visualizing CNV scores in epithelial cell subclusters. **h** Violin plots displaying the activity levels of DNA replication, cell cycle, ferroptosis-related gene sets, and glutathione peroxidase 4 (GPX4) gene among different epithelial cell subclusters. **i** Violin plots comparing the activity levels of DNA replication, cell cycle, ferroptosis-related gene sets, and GPX4 gene between tumor epithelial cells and normal epithelial cells. Wilcoxon rank-sum test (two-tailed). *P < 0.05; **P < 0.01; ns, not statistically significant

The volcano plot identified 6178 significant differentially expressed genes (DEGs) in the Cu-DMSA-HA group compared with the control group, of which 2055 genes were significantly upregulated and 4123 genes were significantly downregulated (Figs. 4a, S9a-c, and Table S2). Gene Ontology (GO) and Kyoto Encyclopedia of Genes and Genomes (KEGG) enrichment analyses revealed that the downregulated DEGs were predominantly associated with biological processes such as cell cycle regulation and DNA replication pathways closely linked to tumor proliferation and migration (Figs. 4b and S9d). In contrast, the upregulated DEGs were enriched in biological processes related to protein folding, cellular response to unfolded proteins, and endoplasmic reticulum (ER) stress (Fig. S9e). Notably, these processes are hallmark features of oxidative stress [36–38]. In line with these findings, KEGG pathway analysis further demonstrated significant activation of pathways involved in protein processing in the ER and ferroptosis (Fig. 4c). Ferroptosis is a form of programmed cell death characterized by excessive lipid peroxidation and is closely associated with oxidative stress. The accumulation of ROS induces oxidative damage to cellular components, particularly membrane lipids, ultimately leading to ferroptotic cell death [39–41].

Moreover, we found that key genes including ACSL4 and SLC7A11 were significantly upregulated and promoted ferroptosis, whereas the ferroptosis-inhibiting gene GPX4 was downregulated (Fig. 4a). GPX4 is a crucial suppressor of oxidative stress-induced ferroptosis, and its activity was regulated by glutathione [42–44]. These results suggested that Cu-DMSA-HA might have induced ferroptosis in NSCLC cells through an oxidative stress-mediated mechanism, highlighting a potential therapeutic pathway through the disruption of redox homeostasis.

To further elucidate the differential regulation of key signaling pathways by Cu-DMSA-HA in tumor and normal cells, we performed scRNA-seq analysis of human NSCLC tissues. The present study integrated three scRNA-seq datasets from NSCLC samples. Following stringent quality control and data filtering, transcriptomic data from 13,548 cells were retained for downstream analysis. Dimensionality reduction and unsupervised clustering identified 18 distinct clusters (Fig. S10). Based on canonical marker gene expression, these clusters were annotated as B cells, dendritic cells (DCs), endothelial cells, epithelial cells, fibroblasts, macrophages, monocytes, neutrophils, natural killer (NK) cells, and T cells (Figs. 4d, e, and S11a). Subsequent re-clustering of epithelial cells revealed five epithelial cell subpopulations (Figs. S11b, c, and S12). Using the inferCNV algorithm (inferCNV) and total copy number variation (CNV) scores, epithelial cell 1 and 4 were classified as normal epithelial subpopulations, while epithelial cell 2, 3, and 5 exhibited substantial CNV alterations, indicating a malignant epithelial phenotype (Fig. 4f, g). Module scoring analysis using the AddModuleScore function showed elevated activity of DNA replication and cell cycle-associated gene sets in malignant epithelial cells relative to their normal epithelial cell subpopulations (*P* < 0.05; Fig. 4h, i).

Although ferroptosis-related gene expression exhibited heterogeneity across epithelial subsets, malignant epithelial cells consistently displayed lower ferroptosis signature scores than their normal counterparts, suggesting a suppression of ferroptosis in NSCLC (Fig. 4h, i). This observation was corroborated by elevated module scores for GPX4, a key inhibitor of ferroptosis, which showed higher activity in malignant epithelial cells (Fig. 4h, i). Overall, integrated transcriptomic RNA-seq and scRNA-seq analyses provided compelling evidence that Cu-DMSA-HA may restore ferroptosis in NSCLC cells via oxidative stress, offering mechanistic insight into its tumor-selective therapeutic potential.

### Cu-DMSA-HA Inhibits NSCLC Cell Proliferation, Invasion, and Migration

**Fig. 5:**
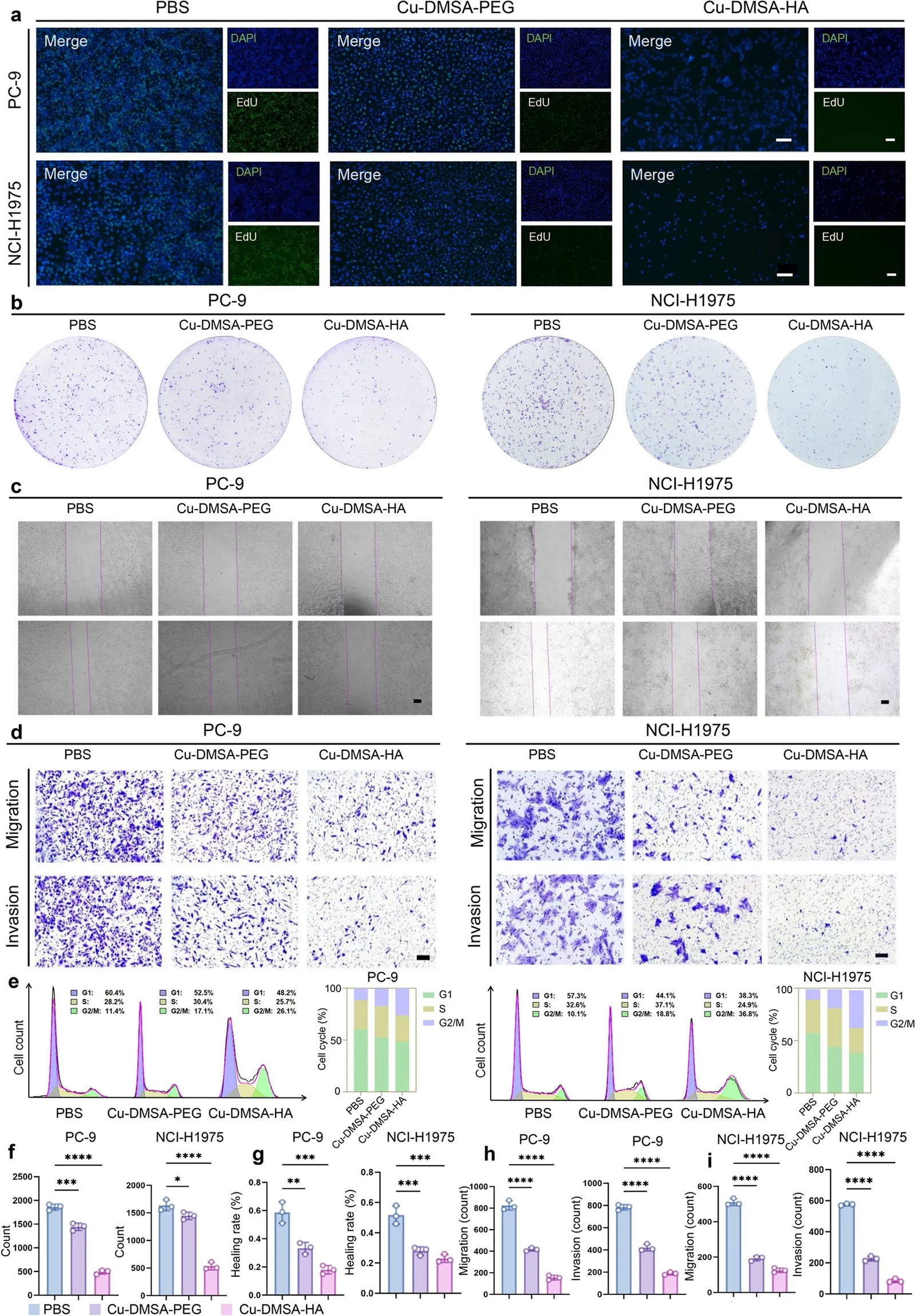
Evaluation of the capability of Cu-DMSA-HA in inhibiting the malignant biological behaviors of NSCLC cells. **a** 5-ethynyl-2’-deoxyuridine (EdU) assay comparing the DNA replication capabilities of PC-9 and NCI-H1975 cells following treatment with PBS, Cu-DMSA-PEG, or Cu-DMSA-HA. Green fluorescence: EdU-positive cells; blue fluorescence: nuclei. Scale bar: 100 μm. **b** Colony formation assay comparing the clonogenic capabilities of PC-9 and NCI-H1975 cells treated with PBS, Cu-DMSA-PEG, or Cu-DMSA-HA, and **f** corresponding quantitative analysis (n = 3; mean ± SD). One-way analysis of variance (ANOVA) followed by Tukey’s post hoc test. **c** Wound-healing assay evaluating cell migration in PC-9 and NCI-H1975 cells treated with PBS, Cu-DMSA-PEG, or Cu-DMSA-HA, and **g** corresponding quantitative analyses (*n* = 3; mean ± SD). One-way ANOVA followed by Tukey’s post hoc test. Scale bar: 500 μm. **d** Transwell assays comparing the invasive and migratory capabilities of PC-9 and NCI-H1975 cells treated with PBS, Cu-DMSA-PEG, or Cu-DMSA-HA, with **h and i** corresponding quantitative analyses (n = 3; mean ± SD). One-way ANOVA followed by Tukey’s post hoc test. Scale bar: 100 μm. **e** Flow cytometry analysis assessing cell division capacity and cell cycle percentage of PC-9 and NCI-H1975 cells following treatment with PBS, Cu-DMSA-PEG, or Cu-DMSA-HA. *P < 0.05; **P < 0.01; ***P < 0.001; ****P < 0.0001

To further validate the antitumor effect of Cu-DMSA-HA NPs, we conducted cell-based experiments. 5-Ethynyl-2’-deoxyuridine (EdU) assays demonstrated that Cu-DMSA-HA markedly inhibited DNA replication in PC-9 and NCI-H1975 cells, showing significantly stronger inhibitory effects compared with Cu-DMSA-PEG (Figs. 4a, S9a-c, and Table S2). Colony formation assays further confirmed that Cu-DMSA-HA treatment remarkably reduced clonogenic capacity of these NSCLC cells relative to Cu-DMSA-PEG treatment (*P* < 0.05; Fig. 5b, f). These results collectively indicated that Cu-DMSA-HA effectively suppressed NSCLC proliferation.

Wound-healing assays demonstrated that Cu-DMSA-HA markedly suppressed the migration capacity of PC-9 and NCI-H1975 cells, with significantly stronger inhibitory effects than Cu-DMSA-PEG (*P* < 0.05; Fig. 5c, g). Consistently, results from Transwell assays further validated that Cu-DMSA-HA markedly suppressed the invasion and migration abilities of NSCLC cells relative to Cu-DMSA-PEG treatment (*P* < 0.05; Fig. 5d, h, i). Taken together, these findings highlighted that Cu-DMSA-HA could effectively inhibit the invasive and metastatic behaviors of NSCLC cells.

Moreover, flow cytometric analysis of the cell cycle demonstrated that Cu-DMSA-HA treatment induced cell cycle arrest at the G2/M phase in both PC-9 and NCI-H1975 cells (Fig. 5e). Notably, Cu-DMSA-HA exhibited a more pronounced G2/M phase arrest effect compared to Cu-DMSA-PEG (Fig. 5e). These findings indicated that Cu-DMSA-HA could effectively suppress NSCLC cell proliferation by disrupting cell cycle progression.

Overall, Cu-DMSA-HA could effectively inhibit proliferation, invasion, migration, and cell cycle progression of NSCLC cells. These findings underscored the therapeutic potential of Cu-DMSA-HA as a promising antitumor agent in the treatment of NSCLC.

### Cu-DMSA-HA Inhibits NSCLC Progression through Oxidative Stress-induced Ferroptosis

**Fig. 6:**
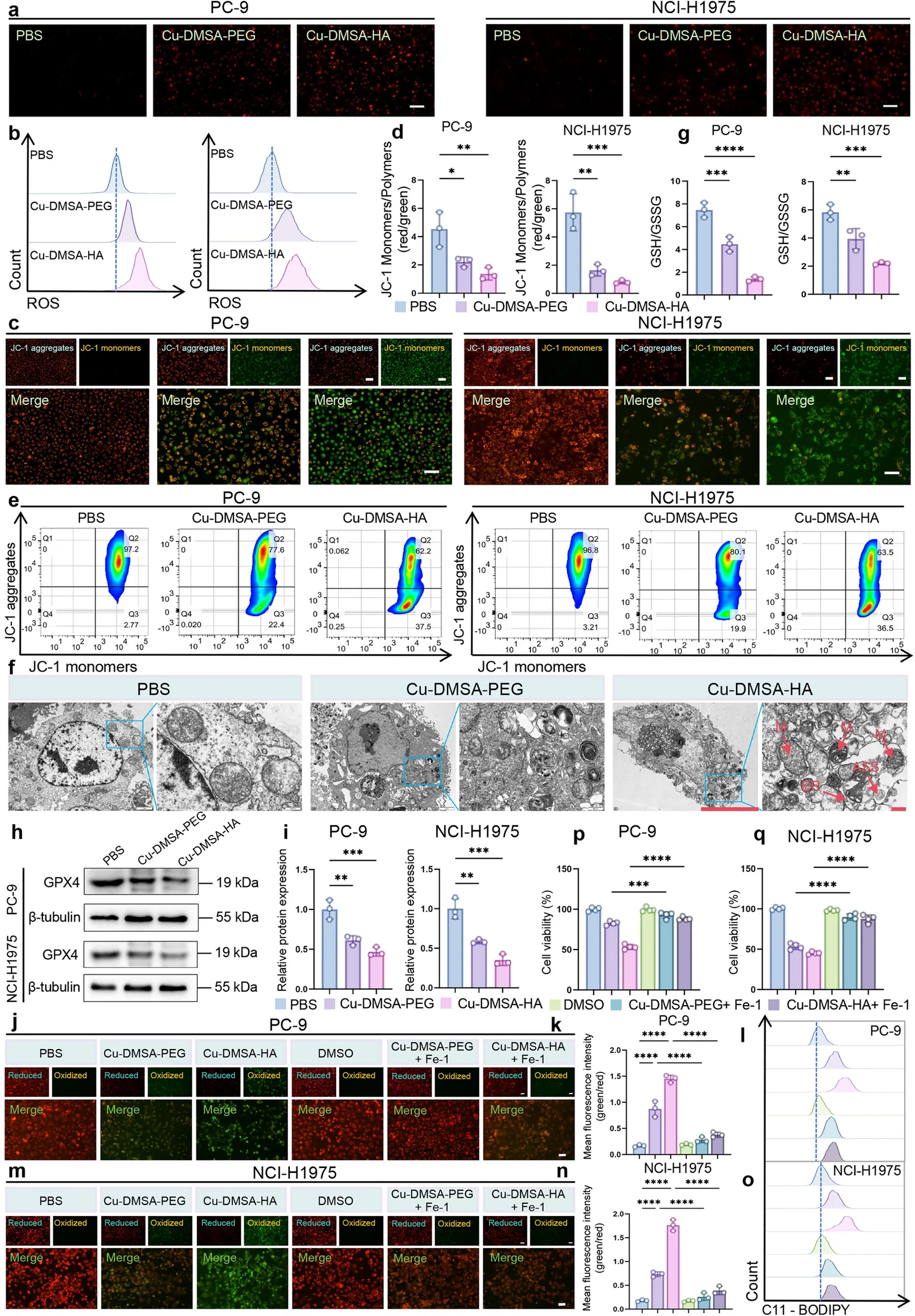
Evaluation of oxidative stress and ferroptosis induction by Cu-DMSA-HA in NSCLC cells. **a** ROS staining of PC-9 and NCI-H1975 cells following treatment with PBS, Cu-DMSA-PEG, or Cu-DMSA-HA. Scale bar: 100 μm. **b** Flow cytometry analysis of ROS levels in PC-9 and NCI-H1975 cells treated with PBS, Cu-DMSA-PEG, or Cu-DMSA-HA. **c** Mitochondrial membrane potential (Δ*ψm*) staining of PC-9 and NCI-H1975 cells following treatment with PBS, Cu-DMSA-PEG, or Cu-DMSA-HA, and **d** corresponding quantitative fluorescence intensity analyses (*n* = 3; mean ± SD). One-way ANOVA followed by Tukey’s post hoc test. Red fluorescence: JC-1 aggregates (healthy mitochondria, high membrane potential); green fluorescence: JC-1 monomers (damaged mitochondria, low membrane potential). Scale bar: 100 μm. **e** Flow cytometry analysis of Δ*ψm* in PC-9 and NCI-H1975 cells treated with PBS, Cu-DMSA-PEG, or Cu-DMSA-HA. **f** TEM images depicting ultrastructural changes in PC-9 and NCI-H1975 cells after treatment with PBS, Cu-DMSA-PEG, or Cu-DMSA-HA. N: nucleus; M: mitochondria; ER: endoplasmic reticulum; ASS: autolysosome. **g** Quantification of reduced GSH and GSSG levels in PC-9 and NCI-H1975 cells following treatments with PBS, Cu-DMSA-PEG, or Cu-DMSA-HA (*n* = 3; mean ± SD). One-way ANOVA followed by Tukey’s post hoc test. **h** Western blot analysis and **i** quantification of GPX4 protein expression in PC-9 and NCI-H1975 cells after treatments with PBS, Cu-DMSA-PEG, or Cu-DMSA-HA (*n* = 3; mean ± SD). One-way ANOVA followed by Tukey’s post hoc test. C11-BODIPY staining and fluorescence microscopy analysis of lipid ROS accumulation in **j-k** PC-9 and **m–n** NCI-H1975 cells after treatments with PBS, Cu-DMSA-PEG, Cu-DMSA-HA, DMSO, Cu-DMSA–PEG + ferrostatin-1 (Fer-1), or Cu-DMSA-HA + Fer-1 (*n* = 3; mean ± SD). One-way ANOVA followed by Tukey’s post hoc test. C11-BODIPY staining flow cytometry quantification of lipid ROS levels in **l** PC-9 and **o** NCI-H1975 cells after treatments with PBS, Cu-DMSA-PEG, Cu-DMSA-HA, DMSO, Cu-DMSA-PEG + Fer-1, or Cu-DMSA-HA + Fer-1. CCK-8 assay of cell viability in **p** PC-9 and **q** NCI-H1975 cells after treatments with DMSO, Cu-DMSA-PEG, Cu-DMSA-HA, or in combination with Fer-1 (*n* = 4; mean ± SD). One-way ANOVA followed by Tukey’s post hoc test. *P < 0.05; **P < 0.01; ***P < 0.001; ****P < 0.0001

Previous studies have demonstrated that copper-based nanomaterials are capable of generating ROS via Fenton-like reactions [45–47]. However, it remained to be determined whether Cu-DMSA-HA could induce similar oxidative stress in NSCLC cells through coordination-triggered catalytic ROS generation. Transcriptomic RNA-seq analysis revealed that oxidative stress-related signaling pathways were upregulated in lung cancer cells (PC-9, NCI-H1975) following Cu-DMSA-HA treatment (Figs. 4c and S9e). This finding was further supported by ROS detection assays, which demonstrated a significant increase in intracellular ROS levels in PC-9 and NCI-H1975 cells upon treatment with both Cu-DMSA-HA and Cu-DMSA-PEG. Notably, Cu-DMSA-HA induced higher ROS accumulation compared to Cu-DMSA-PEG (Fig. 6a, b).

High levels of ROS could induce depolarization of the mitochondrial membrane potential (Δ*ψm*). JC-1 staining showed a reduction in red fluorescence (JC-1 aggregates) and an increase in green fluorescence (JC-1 monomers) in PC-9 and NCI-H1975 cells following treatment with Cu-DMSA-HA or Cu-DMSA-PEG, which indicated a significant decrease in Δ*ψm* (*P* < 0.05; Fig. 6c, d). Flow cytometry analysis further confirmed a marked reduction in Δ*ψm* following Cu-DMSA-HA treatment, with a greater decrease observed compared to Cu-DMSA-PEG (Fig. 6e). The reduction in Δ*ψm* was a hallmark of mitochondrial dysfunction and suggested the initiation of apoptosis.

TEM further revealed mitochondrial structural damage in cells treated with Cu-DMSA-HA or Cu-DMSA-PEG. In contrast to the well-preserved cellular architecture observed in the control group, treated cells exhibited irregular morphology alterations, including irregular cell shapes and nuclear deformation. In the cytoplasm, mitochondria appeared shrunken with increased membrane electron density, along with loss, thickening, and blurring of cristae. Additionally, moderate ER dilation and an increased number of autolysosomes were observed (Fig. 6f). These ultrastructural alterations were consistent with oxidative stress-induced mitochondrial injury and further supported the role of Cu-DMSA-HA in triggering mitochondria-dependent cell death pathways.

Transcriptomic RNA-seq analysis revealed that ferroptosis-related signaling pathways were upregulated following Cu-DMSA-HA treatment (Fig. 4c). Consistently, TEM demonstrated characteristic ultrastructural features associated with ferroptosis, including condensed and shrunken mitochondria, reduced or absent cristae, and increased mitochondrial membrane density, particularly in the Cu-DMSA-HA-treated group (Fig. 6f). GSH is a vital antioxidant that maintained cellular redox homeostasis and protected cells from lipid peroxidation. Ferroptosis, a regulated form of cell death driven by iron-dependent accumulation of lipid ROS, is closely associated with GSH depletion [48–50]. In the present study, a significant reduction in the GSH/oxidized glutathione (GSSG) ratio was observed in both PC-9 and NCI-H1975 cells following the Cu-DMSA-HA treatment, compared to the control and Cu-DMSA-PEG groups, indicating pronounced oxidative stress and impaired antioxidant defense (*P* < 0.05; Fig. 6g). The depletion of intracellular GSH compromises the activity of GPX4, a key enzyme that inhibits ferroptosis by reducing lipid hydroperoxides [42, 51].

Moreover, Western blot analysis revealed a marked downregulation of GPX4 protein levels upon treatment with both Cu-DMSA-HA and Cu-DMSA-PEG, further supporting the notion that redox imbalance and disruption of the antioxidant system contributed to the induction of ferroptosis (*P* < 0.05; Fig. 6h, i). Consistently, C11-BODIPY staining demonstrated that both Cu-DMSA-PEG and Cu-DMSA-HA treatments led to a significant accumulation of lipid ROS in PC-9 and NCI-H1975 cells, as confirmed by fluorescence microscopy and flow cytometry analyses (*P* < 0.05; Fig. 6j–o). Importantly, the addition of ferrostatin-1 (Fer-1), a specific ferroptosis inhibitor, markedly reduced lipid ROS accumulation and rescued cell viability, as evidenced by both fluorescence microscopy analysis and CCK-8 assays (*P* < 0.05; Fig. 6p, q). These results indicate that the observed oxidative stress was primarily attributable to ferroptotic lipid peroxidation. Taken together, Cu-DMSA-HA promoted ferroptosis through oxidative stress-induced redox imbalance and antioxidant system breakdown in NSCLC cells.

To further elucidate the selective sensitivity of Cu-DMSA-HA NPs toward specific NSCLC cell lines and to provide a mechanistic rationale for identifying clinically responsive patient subgroups, we investigated key molecular determinants based on the known antitumor mechanisms of Cu-DMSA–-HA. First, considering the CD44-targeting capability of HA, we assessed CD44 expression levels across commonly used NSCLC cell lines based on the Human Protein Atlas database (https://www.proteinatlas.org/). The results showed that PC-9 and NCI-H1975 cells exhibited markedly higher CD44 expression compared with A549, NCI-H322, and NCI-H460 cells (Fig. S14a), suggesting that enhanced NPs internalization via HA-CD44 interactions may contribute to their increased susceptibility to Cu-DMSA-HA. Second, we examined the ferroptosis-related gene ACSL4, a key enzyme involved in promoting lipid peroxidation and a recognized marker of ferroptosis susceptibility [52, 53]. Analysis revealed that ACSL4 was also more highly expressed in PC-9 and NCI-H1975 cells, indicating a potentially greater sensitivity to lipid peroxidation triggered by intracellular ROS accumulation (Fig. S14b). Third, to evaluate the intrinsic antioxidant capacity of these cell lines, we measured the intracellular GSH/GSSG ratio. Notably, PC-9 and NCI-H1975 cells exhibited lower GSH/GSSG ratios compared to other cell lines (Fig. S14c), suggesting a relatively weakened redox buffering capacity that may render them more vulnerable to ROS-induced cytotoxicity upon Cu-DMSA-HA treatment. Collectively, these findings indicate that the heightened sensitivity of PC-9 and NCI-H1975 cells to Cu-DMSA-HA NPs results from the combined effects of efficient CD44-mediated NP uptake, increased ferroptosis susceptibility, and diminished intrinsic antioxidant capacity. These molecular features may serve as potential biomarkers for identifying patients who are more likely to benefit from Cu-DMSA-HA-based therapy.

### Cu-DMSA-HA Targets NSCLC Cells In Vivo

Given the excellent antitumor activity of Cu-DMSA-HA observed in vitro, a hemocompatibility assay was first performed to evaluate its suitability for intravenous administration in vivo*.* The results showed that no hemolysis occurred even at a concentration of 2.5 mg mL^−1^, indicating that Cu-DMSA-HA possessed excellent hemocompatibility and was suitable for intravenous (IV) administration (Fig. S15).

**Fig. 7:**
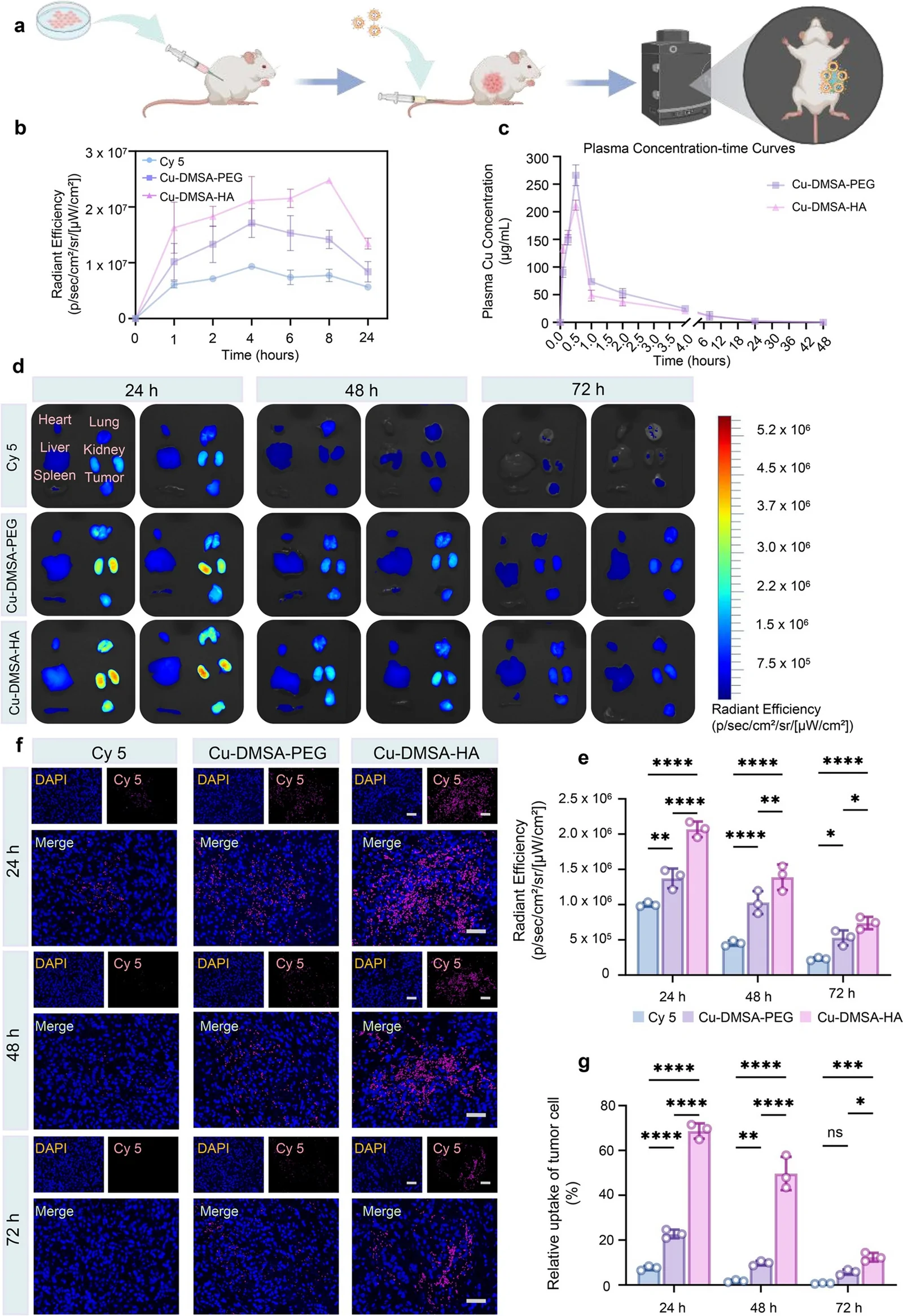
Evaluation of the in vivo tumor-targeting capability and organ clearance of Cu-DMSA-HA NPs. **a** Schematic illustration of the in vivo biodistribution experiment of NPs (*n* = 3). **b** Time-dependent changes in the average fluorescence intensity around the tumor at 0, 1, 2, 4, 6, 8, and 24 h after intravenous (IV) injection of free Cy5 (10 nmol), Cy5-labeled Cu-DMSA-PEG NPs (2 mg kg^−1^, containing 10 nmol Cy5), or Cy5-labeled Cu-DMSA-HA NPs (2 mg kg^−1^, containing 10 nmol Cy5). **c** Plasma concentration–time curves of Cu-DMSA-PEG and Cu-DMSA-HA NPs (*n* = 3; mean ± SD). Plasma concentrations of Cu were measured at predetermined time points after intravenous administration of Cy5-labeled Cu-DMSA-PEG or Cu-DMSA-HA NPs (2 mg kg^−1^) in mice. **d** Ex vivo fluorescence images of major organs (heart, liver, spleen, lung, kidneys) and tumors at 24-h, 48-h, and 72-h post-intravenous injection of free Cy5, Cu-DMSA-PEG, or Cu-DMSA-HA NPs. **e** Quantitative analysis of tumor tissue radiant efficiency at 24 h, 48 h, and 72 h following intravenous injection of free Cy5, Cu-DMSA-PEG, or Cu-DMSA-HA NPs (*n* = 3; mean ± SD). One-way ANOVA followed by Tukey’s post hoc test. **f** Representative immunofluorescence (IF) images of tumor sections at 24 h, 48 h, and 72 h after treatment with Cy5, Cu-DMSA-PEG, or Cu-DMSA-HA. Nuclei were stained with DAPI (blue) and Cy5-labeled NPs are shown in magenta. Scale bar: 50 μm. **g** Quantitative analysis of Cy5 signals in tumor sections at 24 h, 48 h, and 72 h after treatment with Cy5, Cu-DMSA-PEG, or Cu-DMSA-HA, reflecting the relative uptake by tumor cells (n = 3; mean ± SD). One-way ANOVA followed by Tukey’s post hoc test. *P < 0.05; **P < 0.01; ***P < 0.001; ****P < 0.0001; ns, not statistically significant

To compare the in vivo tumor-targeting capability of Cu-DMSA-HA and Cu-DMSA-PEG NPs, both formulations were conjugated with the fluorescent dye Cy5 and administered via IV injection in a subcutaneous tumor model. Real-time in vivo fluorescence imaging was then performed to monitor the biodistribution of the NPs over time (Fig. 7a). In the Cy5 and Cu-DMSA-PEG groups, fluorescence intensity surrounding the tumor region increased during the first 4 h post-injection and then gradually declined (Figs. 7b and S16). In contrast, the Cu-DMSA-HA group exhibited a progressive increase in fluorescence intensity around the tumor over the first 8 h, with pronounced accumulation at the tumor site. Notably, the Cu-DMSA-HA group exhibited significantly higher overall fluorescence intensity compared to the Cy5 and Cu-DMSA-PEG groups, a trend that persisted up to 24 h post-injection (Figs. 7b and S16).

To further characterize the in vivo pharmacokinetic (PK) profiles of the NPs, plasma concentration–time curves of Cu-DMSA-PEG and Cu-DMSA-HA were established following intravenous administration at a dose of 2 mg kg^−1^ in mice. Noncompartmental analysis (NCA) was performed following standard PK procedures as previously described in related nanomedicine studies, which provided methodological guidance for parameter estimation and data processing [54]. As shown in Table S3, both formulations exhibited the same time to reach maximum concentration (*T*_max_ = 0.5 h). The maximum plasma concentration (*C*_max_) of Cu-DMSA-HA (212.2 ± 8.9 μg mL^−1^) was slightly lower than that of Cu-DMSA-PEG (275.7 ± 13.7 μg mL^−1^), consistent with the ICP–OES quantification results (Table S1). Copper-based NPs generally suffer from a short in vivo elimination half-life (*t*_1/2_), which limits their sustained therapeutic efficacy [55, 56]. However, Cu-DMSA-HA exhibited a prolonged *t*_1/2_ (7.8 ± 0.4 h) and mean residence time (MRT = 12.4 ± 0.5 h) compared with Cu-DMSA-PEG (*t*_1/2_ = 6.4 ± 0.8 h; MRT = 9.2 ± 0.9 h), and the *t*_1/2_ of Cu-DMSA-HA was also slightly longer than that of other copper-based NPs reported by Li et al. (*t*_1/2_ = 7.23 ± 1.63) [57], indicating enhanced circulation stability. Interestingly, Cu-DMSA-HA displayed a higher plasma clearance (CL = 4.20 ± 0.15 mL h^−1^ kg^−1^) and a larger apparent volume of distribution (Vss = 52.1 ± 2.8 mL kg^−1^) than Cu-DMSA-PEG (CL = 3.15 ± 0.12 mL h^−1^ kg^−1^; Vss = 29.0 ± 3.0 mL kg^−1^), suggesting improved tissue distribution likely attributed to the HA-mediated tumor-targeting effect. Although the area under the plasma concentration–time curve from zero to infinity (AUC₀–∞ = 476.3 ± 15.0 μg·h mL^−1^) of Cu-DMSA-HA was slightly lower than that of Cu-DMSA-PEG (635.8 ± 24.6 μg·h mL^−1^), its prolonged circulation and enhanced tissue distribution highlight superior PK characteristics and strong potential for tumor-targeted drug delivery (Fig. 7c and Table S3).

To further evaluate the in vivo tumor-targeting capability and off-target clearance of Cu-DMSA-HA NPs, ex vivo fluorescence imaging was performed on major organs (heart, liver, spleen, lung, kidneys) and tumors at 24-, 48-, and 72-h post-intravenous injection in tumor-bearing mice. The Cu-DMSA-HA group exhibited markedly higher fluorescence intensity in tumors at 24 and 48 h, compared to both the free Cy5 and Cu-DMSA-PEG groups (*P* < 0.05; Figs. 7d, e, and S17a). Notably, at 72 h, tumor accumulation in the Cu-DMSA-HA group remained superior to the Cu-DMSA-PEG group, suggesting sustained intratumoral retention (Figs. 7d, e, and S17a). Consistent with macroscopic biodistribution patterns, immunofluorescence (IF) staining at the cellular level further confirmed enhanced accumulation of Cu-DMSA-HA within tumor tissues across all time points, highlighting its superior cellular uptake and retention compared to control groups (*P* < 0.05; Fig. 7f, g).

Regarding off-target distribution, both Cu-DMSA-PEG and Cu-DMSA-HA NPs exhibited pronounced accumulation in the lungs and kidneys at 24 h, which gradually declined over time. By 72 h, fluorescence signals in these organs were minimal, indicating efficient metabolic clearance (Figs. 7d, e, and S17b–k). Importantly, the heart, liver, and spleen consistently showed negligible fluorescence signals at all examined time points, suggesting minimal nonspecific uptake in these critical organs (Figs. 7d, e, and S17b–k).

Collectively, these findings demonstrate that Cu-DMSA-HA NPs possess excellent tumor-targeting specificity, sustained intratumoral retention, and rapid clearance from non-target tissues, underscoring their potential as a safe and effective nanoplatform for in vivo delivery.

### Cu-DMSA-HA Inhibits NSCLC Progression In Vivo

**Fig. 8:**
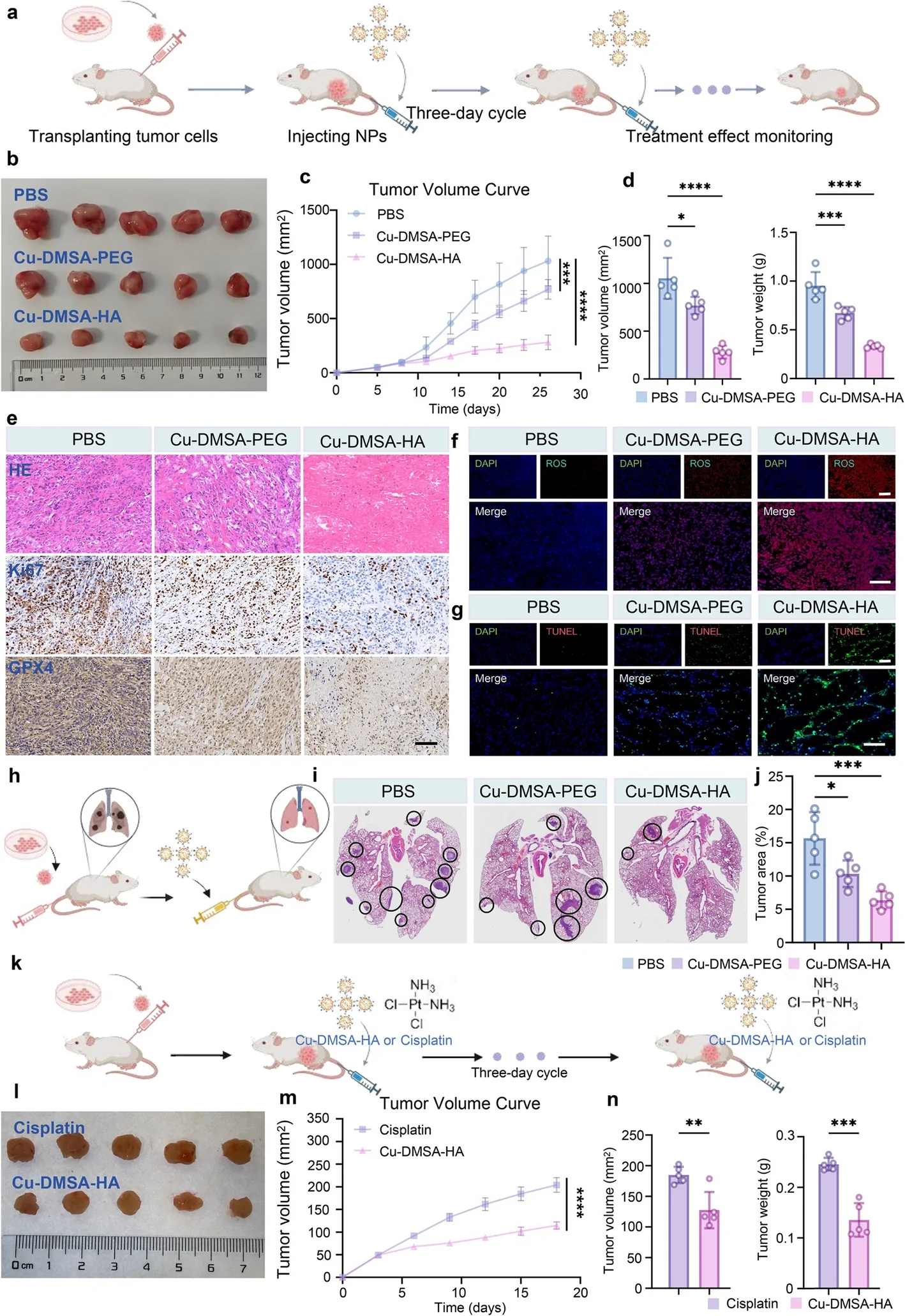
Evaluation of the in vivo antitumor efficacy of Cu-DMSA-HA. **a** Schematic diagram illustrating the establishment of the subcutaneous xenograft tumor model and treatment protocol with PBS, Cu-DMSA-PEG, or Cu-DMSA-HA (*n* = 5). **b** Photograph of excised tumors at the experimental endpoint. **c** Average tumor growth curves for each treatment group (*n* = 5; mean ± SD). One-way ANOVA followed by Tukey’s post hoc test.** d** Comparison of excised tumor volumes and tumor weights at the experimental endpoint (*n* = 5; mean ± SD). One-way ANOVA followed by Tukey’s post hoc test. **e** Representative images of hematoxylin and eosin (H&E) staining and immunohistochemistry (IH**C** staining (Ki-67 and GPX4) of tumor tissues following treatments with PBS, Cu-DMSA-PEG, or Cu-DMSA-HA. IF staining for **f** ROS and **g** terminal deoxynucleotidyl transferase dUTP nick end labeling (TUNEL) of tumor tissues after treatment with PBS, Cu-DMSA-PEG, or Cu-DMSA-HA. Red fluorescence: ROS; green fluorescence: TUNEL; blue fluorescence: nuclei. Scale bar: 200 μm. **h** Schematic illustration of lung metastasis model construction and treatment (*n* = 5). **i** H&E staining of lung tissues collected at the endpoint following PBS, Cu-DMSA-PEG, or Cu-DMSA-HA treatment. **j** The proportion of metastatic lesions to the total lung area after treatment (*n* = 5; mean ± SD). One-way ANOVA followed by Tukey’s post hoc test. **k** Schematic illustration of the subcutaneous xenograft tumor model and treatment regimen with Cu-DMSA-HA or cisplatin (*n* = 5). **l** Photograph of excised tumors at the experimental endpoint. **m** Tumor growth curves during treatment with Cu-DMSA-HA or cisplatin (*n* = 5; mean ± SD). One-way ANOVA followed by Tukey’s post hoc test. **n** Comparison of excised tumor volumes and tumor weights at the experimental endpoint (*n* = 5; mean ± SD) Student’s t-test (unpaired, two-tailed). *P < 0.05; **P < 0.01; ***P < 0.001; ****P < 0.001

Results presented in section “2.6 Cu-DMSA-HA inhibits NSCLC cells in vivo” demonstrated the efficient biodistribution and tumor-targeting capability of Cu-DMSA-HA NPs in vivo, which prompted further evaluation of its therapeutic efficacy in PC-9 tumor-bearing BALB/c mice (Fig. 8a). Following tumor formation, tumor-bearing mice received intravenous injection of phosphate-buffered saline (PBS), Cu-DMSA-PEG, or Cu-DMSA-HA, respectively. Notably, Cu-DMSA-HA exhibited superior antitumor efficacy relative to Cu-DMSA-PEG (P < 0.05; Figs. 8b, c, and S18a). During the treatment period, tumor growth curves revealed that both Cu-DMSA-PEG and Cu-DMSA-HA significantly inhibited tumor progression compared to the PBS group (Figs. 8c and S18b). At the treatment endpoint, ex vivo analysis excised tumor tissues yielded consistent findings, further corroborating the enhanced therapeutic effect of Cu-DMSA-HA (*P* < 0.05; Fig. 8d).

Tumor tissues were harvested and sectioned for histological analysis. Hematoxylin and eosin (H&E) staining, along with Ki-67 immunohistochemistry (IHC), revealed that both Cu-DMSA-PEG and Cu-DMSA-HA treatments significantly inhibited tumor cell proliferation (*P* < 0.05; Figs. 8e and S18c, d). IHC analysis further showed a marked downregulation of GPX4 expression in treated tumors, indicating that both NPs could induce ferroptosis in tumor cells in vivo (*P* < 0.05; Figs. 8e and S18e). ROS fluorescence staining demonstrated a substantial increase in ROS levels within tumor tissues from mice treated with Cu-DMSA-PEG and Cu-DMSA-HA compared to the control group (*P* < 0.05; Figs. 8j and S18f). In addition, terminal deoxynucleotidyl transferase dUTP nick end labeling (TUNEL) staining confirmed significant apoptosis in the tumor sections from both treatment groups, with a notably higher abundance of apoptotic cells observed in the Cu-DMSA-HA group than in the Cu-DMSA-PEG group (*P* < 0.05; Figs. 8k and S18g).

Furthermore, a lung metastasis model was established to evaluate the therapeutic effect of Cu-DMSA-HA for advanced metastatic NSCLC (Fig. 8h). The results demonstrated that Cu-DMSA-HA treatment significantly reduced the size of pulmonary metastatic lesions compared to the PBS control group and exhibited a superior therapeutic effect compared to Cu-DMSA-PEG (*P* < 0.05; Fig. 8i, j). These results collectively demonstrated that Cu-DMSA-HA exerted potent antitumor effects in vivo and exhibited superior therapeutic efficacy compared to PBS and Cu-DMSA-PEG groups.

### Comparison of Antitumor Efficacy and Biosafety between Cu-DMSA-HA NPs and Cisplatin in NSCLC In Vitro and In Vivo

To evaluate the therapeutic potential of Cu-DMSA-HA NPs relative to standard clinical treatment, we compared their antitumor efficacy with cisplatin, a first-line chemotherapeutic agent for NSCLC, using PC-9 and NCI-H1975 cell lines. First, the half-maximal inhibitory concentration (IC_50_) of Cu-DMSA-HA and cisplatin were determined after 72 h of treatment. Both agents exhibited dose-dependent inhibition of cell viability. The IC_50_ values of Cu-DMSA-HA were 6.64 and 1.59 μg mL^−1^ for PC-9 and NCI-H1975 cells, respectively, while cisplatin yielded IC_50_ values of 4.43 μM and 19.94 μM for the same cell lines (Fig. S19a, b). To further assess therapeutic efficacy under clinically relevant conditions, two complementary approaches were employed:

IC_50_-Normalized Dose Comparison.

PC-9 and NCI-H1975 cells were treated with Cu-DMSA-HA or cisplatin at concentrations of 0.5×, 1×, 1.5×, and 2× their respective IC₅₀ values, and cell viability was evaluated at 24, 48, and 72 h. As shown in the heatmaps (Fig. S19c, d), Cu-DMSA-HA consistently exhibited stronger cytotoxic effects than cisplatin across all tested doses and time points. This enhanced efficacy may be attributed to the superior catalytic ROS-generating capacity of the Cu-DMSA-HA nanoplatform.

Fixed-Dose Comparison.

To mimic therapeutic exposure conditions, Cu-DMSA-HA was administered at a fixed concentration of 30 μg mL^−1^—a dose previously validated to be non-toxic in vivo—while cisplatin was applied at its respective IC_50_. As illustrated in Fig. S19e, f, Cu-DMSA-HA significantly suppressed cell viability in both NSCLC cell lines at all time points, consistently outperforming cisplatin (*P* < 0.05).

Collectively, these results demonstrate that Cu-DMSA-HA NPs possess superior dose- and time-dependent cytotoxic activity compared to cisplatin in vitro, highlighting their potential as a nanocatalytic therapeutic alternative for NSCLC.

Based on these encouraging in vitro findings, we next sought to evaluate the comparative antitumor efficacy and biosafety profiles of Cu-DMSA-HA and cisplatin in an in vivo xenograft mouse model.

To evaluate the therapeutic efficacy of Cu-DMSA-HA NPs in vivo, a subcutaneous xenograft model was established by injecting PC-9 cells into nude mice (Fig. 8k). Mice were intravenously administered either Cu-DMSA-HA NPs or cisplatin. Following treatment, the tumor volumes in the Cu-DMSA-HA group were markedly smaller than those in the cisplatin group (Fig. 8i). The tumor growth curves further confirmed that Cu-DMSA-HA NPs exhibited significantly greater tumor suppression than cisplatin throughout the treatment period (*P* < 0.05; Fig. 8m). At the end of treatment, both the tumor volume and tumor weight were significantly lower in the Cu-DMSA-HA group compared with the cisplatin group (*P* < 0.05; Fig. 8n), indicating superior in vivo antitumor efficacy.

Notably, mice in the cisplatin group experienced a progressive decline in body weight during treatment, suggesting systemic toxicity. In contrast, mice treated with Cu-DMSA-HA NPs maintained stable body weight (Fig. S20), reflecting favorable safety characteristics. To further assess biosafety, complete blood count (CBC) analysis and H&E staining of major organs were performed after treatment. Mice in the cisplatin group showed reduced blood cell counts, indicating hematological toxicity (Fig. S21). Additionally, H&E staining revealed pathological damage in the liver and kidney tissues of cisplatin-treated mice, including hepatic vacuolar degeneration and inflammatory cell infiltration in the kidneys. In contrast, no significant toxic effects were observed in mice treated with Cu-DMSA-HA NPs, either in blood parameters or in histological examination of major organs (Fig. S22).

These results collectively demonstrated that Cu-DMSA-HA NPs possess potent antitumor activity and favorable biosafety, making them suitable candidates for in vivo therapeutic applications in NSCLC.

## Conclusions

In this study, we introduced a facile synthesis approach to develop the copper-based and tumor-targeting Cu-DMSA-HA NPs for the efficient treatment of NSCLC via cellular ROS generation by GSH depletion and then Fenton-like reaction. Typically, the NPs were formed by the coordination of copper ions with DMSA and then modified with HA, which could actively target overexpressed CD44 receptors on the surface of lung cancer cells. After targeting, the NPs react with GSH to generate abundant Cu(I), which then efficiently triggers the Fenton-like reaction, generating ROS for cancer cell therapy. The transcriptomic RNA-seq analysis data revealed that Cu-DMSA-HA NPs significantly inhibited DNA replication and cell cycle progression in cancer cells, thus elevating oxidative stress levels and activating ferroptosis signaling pathways. Both in vitro and in vivo results demonstrated the significantly reduced GSH/GSSG ratio in cancer cells following Cu-DMSA-HA NPs treatment, indicating GSH consumption, severe oxidative stress, and compromised antioxidant defense. In addition, the GSH depletion further inhibited the activity of GPX4, ultimately inducing ferroptosis in NSCLC cells.

Compared with previously reported copper-based nanoplatforms [58–61], our Cu-DMSA-HA NPs exhibit several unique advantages. First, the facile coordination assembly of Cu-DMSA-HA NPs offers a simple and reproducible preparation method. Second, HA modification provides active and specific tumor targeting via CD44 receptor recognition, distinguishing this system from many non-targeted copper nanomaterials. Third, the sequential catalytic mechanism—GSH depletion followed by a Fenton-like reaction—ensures sustained ROS production and efficient ferroptosis induction, thereby enhancing therapeutic efficacy. These differentiated design features highlight the novelty and translational potential of Cu-DMSA-HA NPs.

In summary, the newly developed Cu-DMSA-HA NPs not only exhibit satisfactory biocompatibility but are also capable of suppressing NSCLC progression in both subcutaneous and orthotopic lung tumor models by selectively inducing ferroptosis through Fenton-like reaction. However, this research is in its infancy, there are still plenty of challenges that need to be resolved, including long-term biosafety and the potential drug resistance, etc. Overall, we believe with confidence that the developed NPs, as one representative of nanocatalytic medicine, is of clinical translation potential for benefiting patients in the future.

## Supplementary Information

Below is the link to the electronic supplementary material.

Supplementary file 1 (DOCX 5.55 MB)
