# Correction: Copper-Based Targeted Nanocatalytic Therapeutics for Non-Small Cell Lung Cancer

**DOI:** 10.1007/s40820-026-02239-z

**Published:** 2026-07-28

**Authors:** Yongfei Fan, Jiao Chang, Xichun Qin, Meng Li, Yan Li, Leilei Wu, Kun Li, Zhimin Chen, Yani Li, Zhongmin Tang, Dong Xie, Jianlin Shi

**Affiliations:** 1 Department of Thoracic Surgery, Shanghai Pulmonary Hospital, School of Medicine, Tongji University, Shanghai, 200433 People’s Republic of China; 2 Department of Radiology, Tongji Hospital, School of Medicine, Tongji University, Shanghai, 200331 People’s Republic of China; 3 Shanghai Frontiers Science Center of Nanocatalytic Medicine, School of Medicine, Tongji University, Shanghai, 200331 People’s Republic of China; 4 Department of Thoracic Surgery, Zhejiang Cancer Hospital, Hangzhou Institute of Medicine, Chinese Academy of Sciences, Hangzhou, Zhejiang 310022 People’s Republic of China; 5 Department of Medical Oncology, Shanghai Pulmonary Hospital, School of Medicine, Tongji University, Shanghai, 200433 People’s Republic of China; 6 Shanghai Institute of Ceramics Chinese Academy of Sciences, Research Unit of Nanocatalytic Medicine in Specific Therapy for Serious Disease, Chinese Academy of Medical Sciences, Shanghai, 200050 People’s Republic of China

**Correction to** **:** **Nano-Micro Lett. (2026) 18:152.** 10.1007/s40820-025-01998-5

Following publication of the original article [1], the authors reported the article needed to be updated. The required corrections are detailed below.

1. The **full name of DMSA** was incorrectly written at its first occurrence in the **Highlights, Abstract, Introduction, Fig. 1 legend, and Supplementary Information (Section S1, Experimental Section)**. The correct expression is **“copper (Cu) and dimercaptosuccinic acid (DMSA).”**
2. The **chemical **equations** in the Table of Contents graphic (TOC) and Fig. 1** were incorrectly written. The correct expressions are “**Cu⁺ + H₂O₂ → Cu**^**2**^**⁺ + OH⁻ + •OH.”**
3. Due to an error during image assembly involving copy-and-paste operations, **the statistical plot for PC-9 in Fig. 6i was incorrectly duplicated from that of NCI-H1975.**

All the above corrections do not affect the final conclusions of the article. The correct Table of Contents graphic, Figs. 1 and 6 have been provided in this Correction. The Supplementary file has been updated as well.

The incorrect Table of Contents graphic is:
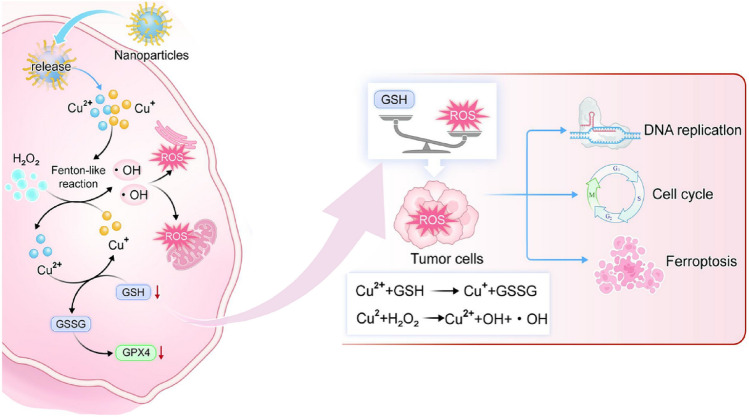


The correct Table of Contents graphic is:
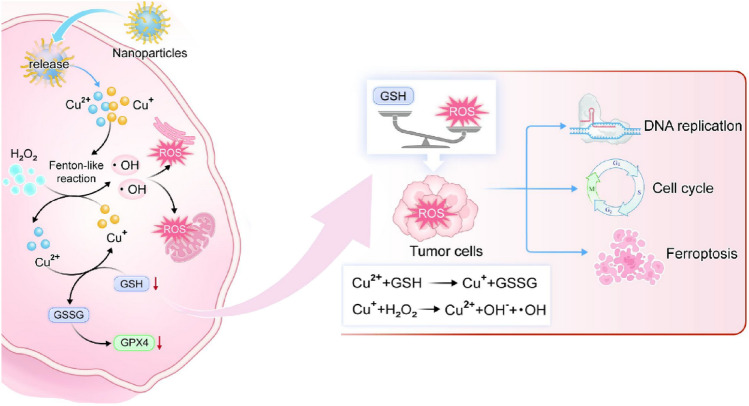


The incorrect Fig. 1 is:

**Fig. 1:**
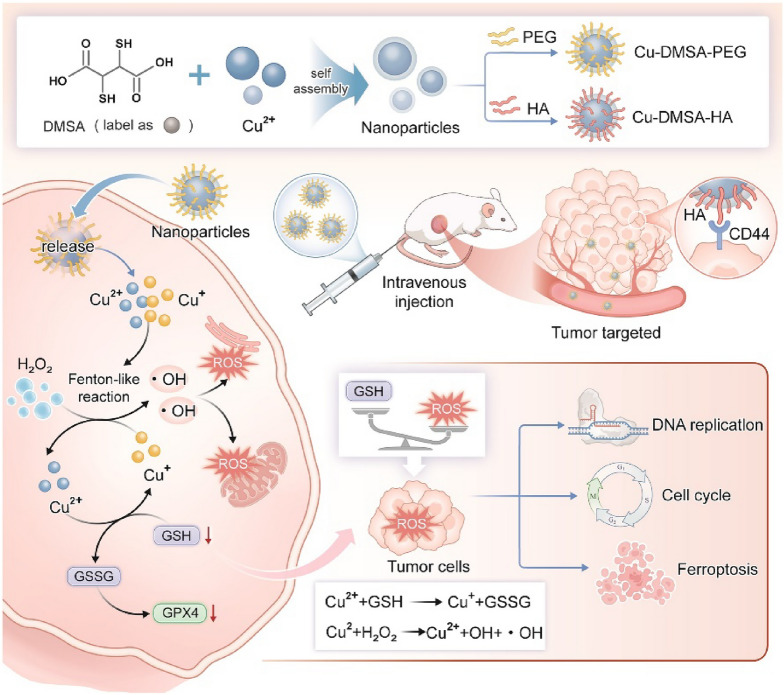
Preparation schematics of nanoparticles (NPs) and proposed therapeutic mechanism. **a** Schematic illustration of the synthesis of copper–N,N-dimethyl-N-phenylsulfonylbisamine (DMSA) NPs modified with polyethylene glycol (Cu-DMSA-PEG NPs) and hyaluronic acid (HA) (Cu-DMSA-HA NPs), respectively. **b** Proposed biological mechanism of Cu-DMSA-HA-induced ferroptosis in tumor cells. Following cellular internalization, Cu-DMSA-HA NPs, which are assembled via coordination chemistry, catalyze intracellular reactive oxygen species (ROS) generation through glutathione (GSH)-mediated reduction and subsequent Fenton-like reactions. The resulting ROS accumulation triggers oxidative damage to mitochondria and the endoplasmic reticulum of tumor cells, leading to downregulation of glutathione peroxidase 4 (GPX4), a key regulator protecting against ferroptosis. **c** Mechanism of in vivo tumor targeting by Cu-DMSA-HA. Cu-DMSA-HA facilitates selective binding to cluster of differentiation 44 (CD44) receptors overexpressed on tumor cells, enhancing tumor-specific accumulation. **d** Antitumor mechanism of Cu-DMSA-HA in vivo. Cu-DMSA-HA inhibits tumor progression by suppressing DNA replication and cell cycle progression while inducing ferroptosis.

The correct Fig. 1 is:

**Fig. 1:**
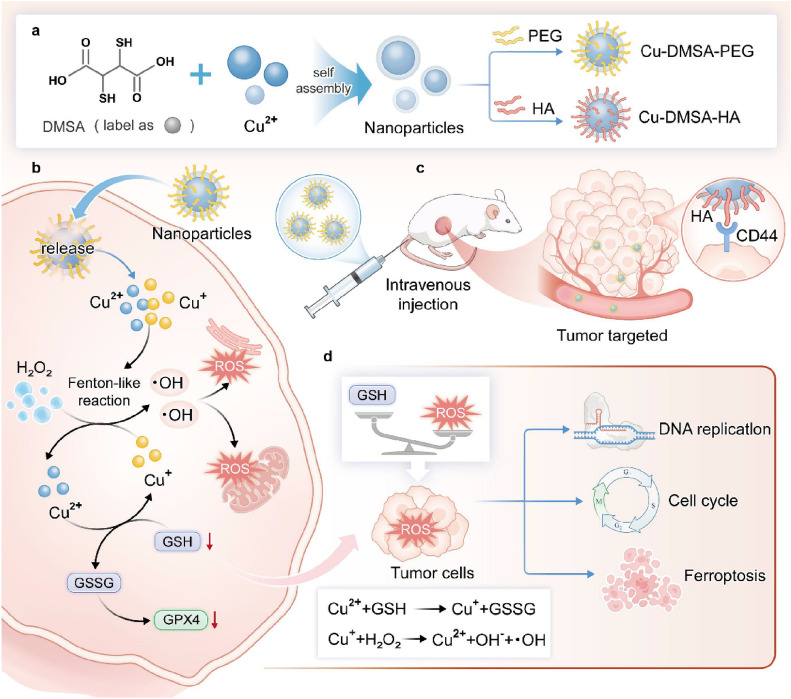
Preparation schematics of nanoparticles (NPs) and proposed therapeutic mechanism. **a** Schematic illustration of the synthesis of copper––dimercaptosuccinic acid (DMSA) NPs modified with polyethylene glycol (Cu-DMSA-PEG NPs) and hyaluronic acid (HA) (Cu-DMSA-HA NPs), respectively. **b** Proposed biological mechanism of Cu-DMSA-HA-induced ferroptosis in tumor cells. Following cellular internalization, Cu-DMSA-HA NPs, which are assembled via coordination chemistry, catalyze intracellular reactive oxygen species (ROS) generation through glutathione (GSH)-mediated reduction and subsequent Fenton-like reactions. The resulting ROS accumulation triggers oxidative damage to mitochondria and the endoplasmic reticulum of tumor cells, leading to downregulation of glutathione peroxidase 4 (GPX4), a key regulator protecting against ferroptosis. **c** Mechanism of in vivo tumor targeting by Cu-DMSA-HA. Cu-DMSA-HA facilitates selective binding to cluster of differentiation 44 (CD44) receptors overexpressed on tumor cells, enhancing tumor-specific accumulation. **d** Antitumor mechanism of Cu-DMSA-HA in vivo. Cu-DMSA-HA inhibits tumor progression by suppressing DNA replication and cell cycle progression while inducing ferroptosis.

The incorrect Fig. 6 is:

**Fig. 6:**
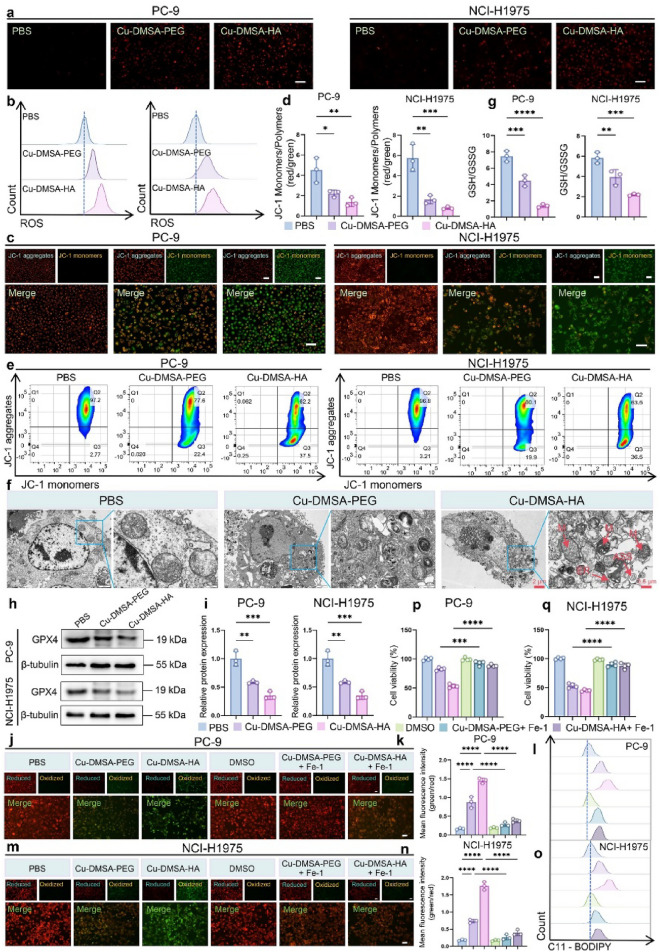
Evaluation of oxidative stress and ferroptosis induction by Cu-DMSA-HA in NSCLC cells. **a** ROS staining of PC-9 and NCI-H1975 cells following treatment with PBS, Cu-DMSA-PEG, or Cu-DMSA-HA. Scale bar: 100 μm. **b** Flow cytometry analysis of ROS levels in PC-9 and NCI-H1975 cells treated with PBS, Cu-DMSA-PEG, or Cu-DMSA-HA. **c** Mitochondrial membrane potential (Δψm) staining of PC-9 and NCI-H1975 cells following treatment with PBS, Cu-DMSA-PEG, or Cu-DMSA-HA, and **d** corresponding quantitative fluorescence intensity analyses (n = 3; mean ± SD). Oneway ANOVA followed by Tukey’s post hoc test. Red fluorescence: JC-1 aggregates (healthy mitochondria, high membrane potential); green fluorescence: JC-1 monomers (damaged mitochondria, low membrane potential). Scale bar: 100 μm.** e** Flow cytometry analysis of Δψm in PC-9 and NCI-H1975 cells treated with PBS, Cu-DMSA-PEG, or Cu-DMSA-HA. **f** TEM images depicting ultrastructural changes in PC-9 and NCI-H1975 cells after treatment with PBS, Cu-DMSA-PEG, or Cu-DMSA-HA. N: nucleus; M: mitochondria; ER: endoplasmic reticulum; ASS: autolysosome. **g** Quantification of reduced GSH and GSSG levels in PC-9 and NCI-H1975 cells following treatments with PBS, Cu-DMSA-PEG, or Cu-DMSAHA (n = 3; mean ± SD). Oneway ANOVA followed by Tukey’s post hoc test. **h** Western blot analysis and **i** quantification of GPX4 protein expression in PC-9 and NCI-H1975 cells after treatments with PBS, Cu-DMSA-PEG, or Cu-DMSA-HA (n = 3; mean ± SD). Oneway ANOVA followed by Tukey’s post hoc test. C11-BODIPY staining and fluorescence microscopy analysis of lipid ROS accumulation in **j-k** PC-9 and **m–n** NCI H1975 cells after treatments with PBS, Cu-DMSA-PEG, Cu-DMSA-HA, DMSO, Cu-DMSA–PEG + ferrostatin-1 (Fer-1), or Cu-DMSA-HA + Fer-1 (n = 3; mean ± SD). Oneway ANOVA followed by Tukey’s post hoc test. C11-BODIPY staining flow cytometry quantification of lipid ROS levels in l PC-9 and **o** NCI H1975 cells after treatments with PBS, Cu-DMSA-PEG, Cu-DMSA-HA, DMSO, Cu-DMSA-PEG + Fer-1, or Cu-DMSAHA + Fer-1. CCK-8 assay of cell viability in **p** PC-9 and **q** NCIH1975 cells after treatments with DMSO, Cu-DMSA-PEG, Cu-DMSA-HA, or in combination with Fer-1 (n = 4; mean ± SD). Oneway ANOVA followed by Tukey’s post hoc test. *P < 0.05; **P < 0.01; ***P < 0.001; ****P < 0.0001.

The correct Fig. 6 is:

**Fig. 6:**
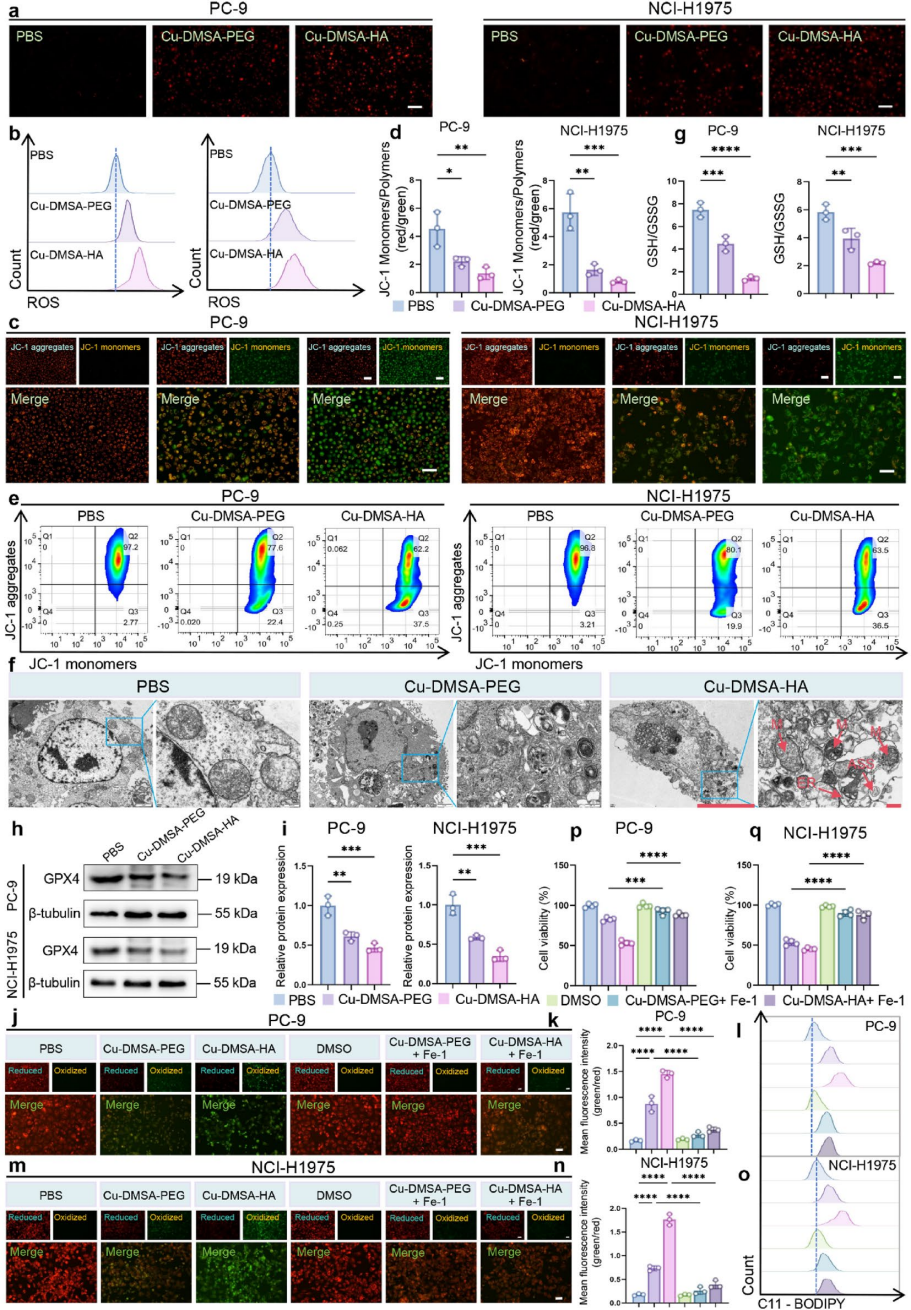
Evaluation of oxidative stress and ferroptosis induction by Cu-DMSA-HA in NSCLC cells. **a** ROS staining of PC-9 and NCI-H1975 cells following treatment with PBS, Cu-DMSA-PEG, or Cu-DMSA-HA. Scale bar: 100 μm. **b** Flow cytometry analysis of ROS levels in PC-9 and NCI-H1975 cells treated with PBS, Cu-DMSA-PEG, or Cu-DMSA-HA. **c** Mitochondrial membrane potential (Δψm) staining of PC-9 and NCI-H1975 cells following treatment with PBS, Cu-DMSA-PEG, or Cu-DMSA-HA, and **d** corresponding quantitative fluorescence intensity analyses (n = 3; mean ± SD). Oneway ANOVA followed by Tukey’s post hoc test. Red fluorescence: JC-1 aggregates (healthy mitochondria, high membrane potential); green fluorescence: JC-1 monomers (damaged mitochondria, low membrane potential). Scale bar: 100 μm.** e** Flow cytometry analysis of Δψm in PC-9 and NCI-H1975 cells treated with PBS, Cu-DMSA-PEG, or Cu-DMSA-HA. **f** TEM images depicting ultrastructural changes in PC-9 and NCI-H1975 cells after treatment with PBS, Cu-DMSA-PEG, or Cu-DMSA-HA. N: nucleus; M: mitochondria; ER: endoplasmic reticulum; ASS: autolysosome. **g** Quantification of reduced GSH and GSSG levels in PC-9 and NCI-H1975 cells following treatments with PBS, Cu-DMSA-PEG, or Cu-DMSAHA (n = 3; mean ± SD). Oneway ANOVA followed by Tukey’s post hoc test. **h** Western blot analysis and **i** quantification of GPX4 protein expression in PC-9 and NCI-H1975 cells after treatments with PBS, Cu-DMSA-PEG, or Cu-DMSA-HA (n = 3; mean ± SD). Oneway ANOVA followed by Tukey’s post hoc test. C11-BODIPY staining and fluorescence microscopy analysis of lipid ROS accumulation in **j-k** PC-9 and **m–n** NCI-H1975 cells after treatments with PBS, Cu-DMSA-PEG, Cu-DMSA-HA, DMSO, Cu-DMSA–PEG + ferrostatin-1 (Fer-1), or Cu-DMSA-HA + Fer-1 (n = 3; mean ± SD). Oneway ANOVA followed by Tukey’s post hoc test. C11-BODIPY staining flow cytometry quantification of lipid ROS levels in l PC-9 and **o** NCI-H1975 cells after treatments with PBS, Cu-DMSA-PEG, Cu-DMSA-HA, DMSO, Cu-DMSA-PEG + Fer-1, or Cu-DMSAHA + Fer-1. CCK-8 assay of cell viability in **p** PC-9 and **q** NCIH1975 cells after treatments with DMSO, Cu-DMSA-PEG, Cu-DMSA-HA, or in combination with Fer-1 (n = 4; mean ± SD). Oneway ANOVA followed by Tukey’s post hoc test. *P < 0.05; **P < 0.01; ***P < 0.001; ****P < 0.0001.

The original article [1] has been corrected.
