# Supplementary material for: Copper-Based Targeted Nanocatalytic Therapeutics for Non-Small Cell Lung Cancer
Source: Nanomicro Lett. 2026 Jan 12;18:152. doi: 10.1007/s40820-025-01998-5 (PMC12791101; doi:10.1007/s40820-025-01998-5)
Supplement: Supplementary file 1 — (DOCX 5.55 MB) [file 40820_2025_1998_MOESM1_ESM.docx]

Supporting Information for

**Copper-Based Targeted Nanocatalytic Therapeutics for Non-Small Cell Lung Cancer**

Yongfei Fan^1,2#^, Jiao Chang^2,3#^, Xichun Qin^1^, Meng Li^2,3^, Yan Li^2,3^, Leilei Wu^4^, Kun Li^1^, Zhimin Chen^5^, Yani Li^2,3^, Zhongmin Tang^1,2,3*^, Dong Xie^1*^, Jianlin Shi^6^

^1^ Department of Thoracic Surgery, Shanghai Pulmonary Hospital, School of Medicine, Tongji University, Shanghai 200433, P. R. China

^2^ Department of Orthopaedics, Shanghai Tenth People's Hospital, School of Medicine, Tongji University, Shanghai 200072, P. R. China

^3^ Shanghai Frontiers Science Center of Nanocatalytic Medicine, School of Medicine, Tongji University, Shanghai 200072, P. R. China

^4^ Department of Thoracic Surgery, Zhejiang Cancer Hospital, Hangzhou Institute of Medicine, Chinese Academy of Sciences, Hangzhou, Zhejiang 310022, P. R. China

^5^ Department of Medical Oncology, Shanghai Pulmonary Hospital, School of Medicine, Tongji University, Shanghai 200433, P. R. China

^6^ Shanghai Institute of Ceramics Chinese Academy of Sciences, Research Unit of Nanocatalytic Medicine in Specific Therapy for Serious Disease, Chinese Academy of Medical Sciences, Shanghai 200050, P. R. China

^#^Yongfei Fan and Jiao Changcontributed equally to this work.

*Corresponding authors. E-mail: [xiedong@tongji.edu.cn](mailto:xiedong@tongji.edu.cn) (Dong Xie); [zhongmintang@tongji.edu.cn](mailto:zhongmintang@tongji.edu.cn) (Zhongmin Tang)

**S1 Experimental Section**

## **S1.1 Materials**

Cupric Sulfate (CuSO_4_), polyethylene glycol (PEG) 4000, hyaluronic acid (HA), dimercaptosuccinic acid (DMSA) and N,N-Dimethylformamide (DMF) were all bought from Aladdin (Shanghai, China).

## **S1.2 The formulation of copper (Cu) and DMSA NPs modified with polyethylene glycol nanoparticles (NPs) (Cu–DMSA–PEG NPs) and HA nanoparticles (Cu–DMSA–HA NPs)**

To synthesize the Cu-DMSA-HA and Cu-DMSA-PEG NPs, the CuSO_4_ solution (10 mg/mL, 1 mL) and the HA/PEG solution (10 mg/mL, 500 $\text{μ}$L) were mixed in 10 mL deionized water by stirring for 10 minutes (min) until homogeneity was achieved. Subsequently, a DMSA solution (10 mg/mL, 1 mL, prepared in DMF) was added dropwise to the mixture and stirred for 2 h at room temperature to form Cu-DMSA-HA and Cu-DMSA-PEG NPs. The pure NPs were obtained through centrifugation (8000 rpm, 15 min) and washing with deionized water for 3 times, followed by dialysis for two days by using a 10000D molecular weight dialysis bag, and finally freeze-dried for storage.

hCu-DMSA-HA and Cu-DMSA-PEG NPs were dispersed in water at concentration of 0.5 mg/mL. The resulting dispersions were then mixed with a cyanine 5 (Cy 5) solution (100 $\text{μ}$L, 0.2 mg/mL) and stirred continuously for 24 h to facilitate the electrostatic adsorption of the fluorescent dye onto the nanoparticle surface. Subsequently, the mixtures underwent hyperfiltration, followed by two washes with deionized water to yield Cy 5-labeled Cu-DMSA-HA and Cu-DMSA-PEG NPs. In addition, the filtrate solutions were also collected to quantify the encapsulated Cy 5 content using UV-vis spectroscopy.

## **S1.3 Characterization of Cu-DMSA-HA and Cu-DMSA-PEG NPs**

The size and polydispersity index (PDI) of Cu-DMSA-HA and Cu-DMSA-PEG NPs were measured by a dynamic light scattering (DLS) instrument (Nano-ZS 90, Malvern). Additionally, the morphology and size of Cu-DMSA-HA and Cu-DMSA-PEG NPs were observed using both transmission electron microscopy (TEM) and scanning electron microscope (SEM). The characteristic peak absorbance was measured with a UV-Vis spectrophotometer (Cry50), while the Cu concentration in Cu-DMSA-HA and Cu-DMSA-PEG NPs were quantified using inductively coupled plasma optical emission spectrometry (ICP-OES) (Agilent 725). The Cu-DMSA-HA and Cu-DMSA-PEG NPs were dispersed in PBS buffer, and their particle sizes were detected using DLS at the predetermined time points to evaluate their stability.

## **S1.4 Reactive oxygen species (ROS) generation and glutathione (GSH) depletion ability in solution**

The ROS generation was quantified by using electron paramagnetic resonance (EPR) spectroscopy and a methylene blue (MB) assay. Specifically, the characteristic peak of MB at 664 nm decreased with the increasing concentration of ROS. For this experiment, a 2 mL phosphate-buffered saline (PBS) solution containing Cu-DMSA-HA and Cu-DMSA-PEG NPs, MB (0.5 μg/mL), and H_2_O_2_ (100 μM) was prepared, and co-incubated for 45 min. Changes in absorbance were measured by UV-Vis spectrometer. For GSH depletion assay, a 2 mL PBS solution containing Cu-DMSA-HA and Cu-DMSA-PEG NPs, MB (0.5 μg/mL) and H_2_O_2_ (100 μM) were mixed with GSH (5 mM), then co- incubated for 45 min. Changes in absorbance were measured by UV-Vis spectrometer.

## **S1.5 Cell culture**

The PC-9 (Research Resource Identifier [RRID]: CVCL_B260), NCI-H460 (RRID: CVCL_0459), NCI-H322 (RRID: CVCL_1556), BEAS-2B (RRID: CVCL_0168), A549 (RRID: CVCL_0023), and NCI-H1975 (RRID: CVCL_1511) cell lines were purchased from Zhongqiao Xinzhou Biotechnology (Shanghai, China) in 2023. All cell lines were authenticated by short tandem repeat (STR) profiling and confirmed to be mycoplasma-free prior to use.

PC-9, NCI-H460, and NCI-H1975 cells were cultured in Roswell Park Memorial Institute 1640 (RPMI-1640) medium (PM150110; Procell, Wuhan, China). NCI-H322, BEAS-2B, and A549 cells were cultured in Dulbecco’s modified eagle medium (DMEM) (PM150210; Procell, Wuhan, China). Both types of culture media were supplemented with 10% fetal bovine serum (FBS; 164210; Procell, Wuhan, China) and 1% penicillin-streptomycin (C0222; Beyotime, Shanghai, China). All cells were maintained in a humidified incubator (Thermo Fisher Scientific, Waltham, MA, USA) at 37 °C with 5% CO₂.

## **S1.6 Cell viability assay**

### ***S1.6.1 CCK-8 assay***

Logarithmically growing cells were seeded into 96-well plates at a density of 8 × 10³ cells per well. After 24 h of incubation, cells were treated with varying concentrations (0, 5, 10, 20, 30, and 40 μg/mL) of Cu-DMSA-HA or Cu-DMSA-PEG NPs, respectively. Following a further 24 h incubation, 100 μL of culture medium containing 10% CCK-8 solution (Cat# C0046; Beyotime, Shanghai, China) was added to each well. The plates were then incubated at 37 °C in the dark for 30 min, and the optical density (OD) values were measured using a microplate reader (Infinite 200 Pro; Tecan, Männedorf, Switzerland) to determine cell viability.

### ***S1.6.2 Live/dead assay***

The PC-9 cells with a density of 70% were seeded in 96-well plates and cultured for 24 h. Cells were subsequently treated with PBS (control), Cu-DMSA-PEG NPs (30 μg/mL), or Cu-DMSA-HA NPs (30 μg/mL) for an additional 24 h. Following this, cells were incubated with Calcein-AM and propidium iodide (PI) at 37°C for 30 min in the dark. Fluorescence signals were then observed and imaged using an inverted fluorescence microscope (NIB-610; Nikon, Tokyo, Japan).

## **S1.7 Cellular uptake assay of Cu-DMSA-PEG NPs and Cu-DMSA-HA NPs**

To assess the cellular uptake of Cu-DMSA-PEG and Cu-DMSA-HA NPs, PC-9 cells were seeded into confocal culture dishes at a density of 1 × 10⁵ cells per dish and incubated at 37°C for 24 h. Subsequently, Cy 5-labeled Cu-DMSA-PEG NPs (30 μg/mL) or Cu-DMSA-HA NPs (30 μg/mL) were added to the culture medium, respectively. Cells were incubated with the two NPs for 2 and 12 h, respectively. Following incubation, nanoparticle-treated cells washed with PBS, and the cell nuclei were stained with 4′,6-diamidino-2-phenylindole (DAPI, a blue fluorescence DBA-binding dye, for 10 min, and the cellular uptake was visualized using a confocal laser scanning microscope (CLSM; Eclipse Ti2-E with A1R; Nikon, Tokyo, Japan).

## **S1.8 Cell transfection**

A siRNA specifically targeting CD44 (sequence: 5′-CCUCCCAGUAUGACAUATT-3′) was synthesized by GenePharma (Shanghai, China) and dissolved in RNase-free water to a final stock concentration of 20 μM, following the manufacturer’s instructions. PC-9 cells in the logarithmic growth phase were seeded into 6-well plates at a density of 2 × 10⁵ cells per well. After 24 h of incubation, the culture medium was replaced with fresh medium, and transfection was performed using FanRi-Trans transfection reagent (FanRi-Trans CE; FanRi® company, Xiamen, China). Cells were then incubated for an additional 48 h prior to subsequent analysis.

## **S1.9 Flow cytometry**

### ***S1.9.1 Apoptosis, cell cycle, ROS and Δψm assays***

PC-9 and NCI-H1975 cells in the logarithmic growth phase were seeded into 6-well plates at a density of 2 × 10⁵ cells per well. After 24 h of incubation, the culture medium was replaced with fresh medium containing PBS (control), Cu-DMSA-PEG (30 μg/mL), or Cu-DMSA-HA (30 μg/mL). After an additional 24-hour treatment, cells were harvested for subsequent flow cytometric analyses.

Apoptotic PC-9 and NCI-H1975 cells were identified using an Annexin V-FITC and PI kit (KGA1300-100; KeyGEN, Nanjing, China), staining was performed according to the manufacturer’s instructions. For cell cycle analysis, cells were fixed in 75% ethanol and subsequently stained with a DNA content detection reagent (KGA511; KeyGEN, Nanjing, China). Mitochondrial membrane potential (Δψm) was assessed using the JC-1 dye (KGA603; KeyGEN, Nanjing, China), and intracellular ROS levels were measured using a commercial ROS assay kit (C1300-2; Pulilai, Beijing, China). All samples were analyzed using a flow cytometer (LSRFortessa; BD Biosciences, USA) and the acquired data were processed with FlowJo software (version 10.8.1).

### ***S1.9.2 Cellular uptake assays of PC-9 and NCI-H1975 cells***

PC-9 and NCI-H1975 cells in the logarithmic growth phase were seeded into 6-well plates at a density of 2 × 10⁵ cells per well and incubated for 24 h. The culture medium was then replaced with Cy 5-labeled Cu-DMSA-PEG (30 μg/mL), or Cy 5-labeled Cu-DMSA-HA (30 μg/mL). After 12 h of incubation, cells were then harvested and analyzed using a flow cytometer to assess the intracellular uptake of Cy 5-labeled NPs. The mean fluorescence intensity of Cy 5 was recorded and used as a quantitative indicator of nanoparticle internalization.

## **S1.10 Cell proliferation assay**

### ***S1.10.1 EdU assay***

PC-9 and NCI-H1975 cells were seeded into 24-well plates at a density of 5 × 10⁴ cells per well and incubated for 24 h. The culture medium was replaced with fresh complete medium containing PBS (control), Cu-DMSA-PEG (30 μg/mL), or Cu-DMSA-HA (30 μg/mL). After 24 h of treatment, 20 μM EdU working solution (Beyotime, Shanghai, China) was added to each well and incubated at 37°C for 2 h. Following incubation, cells were fixed with 4% paraformaldehyde (C0071S; Beyotime, Shanghai, China) for 15 min and permeabilized with 0.3% Triton X-100 (36317ES60; Yeasen, Shanghai, China) for 20 min. The cells were then incubated with the Click-iT reaction cocktail in the dark for another 30 min. After washing three times with PBS, cell nuclei were counterstained with DAPI for 10 min. Finally, EdU-positive cells were observed and imaged using a fluorescence microscope (NIB-610; Nikon, Tokyo, Japan).

### ***S1.10.2 Colony formation assay***

PC-9 and NCI-H1975 cells were seeded into 6-well plates at a density of 2,000 cells per well and incubated for 24 h. The medium was then replaced with fresh medium containing PBS (control), Cu-DMSA-PEG (30 μg/mL), or Cu-DMSA-HA (30 μg/mL). After 24 h of treatment, the medium was removed and replaced with drug-free fresh medium. Cells were then cultured for an additional 10 days, with medium changes every 3 days. At the end of the incubation period, cell colonies were fixed with 4% paraformaldehyde (P0099-3L; Beyotime, Shanghai, China) for 15 min and stained with 0.1% crystal violet solution (C0121-100ml; Beyotime, Shanghai, China) for 20 min. Images of stained colonies were captured, and the number of colonies was quantified using ImageJ software (version 10.8.1).

## **S1.11 Cell invasion and migration assays**

### ***S1.11.1 Wound healing assay***

PC-9 and NCI-H1975 cells were seeded into 6-well plates at a density of 2 × 10⁵ cells per well and cultured until approximately 90% confluency was reached. A straight scratch (“wound”) was made across the cell monolayer using a sterile 200 μL pipette tip. Detached cells were gently removed by washing with PBS, followed by replacement of the culture medium with fresh medium containing PBS (control), Cu-DMSA-PEG (30 μg/mL), or Cu-DMSA-HA (30 μg/mL). Subsequently, cells were incubated at 37°C, and wound area images were captured at 0 and 24 h using a light microscope (NIB-610; Nikon, Tokyo, Japan). The wound area was quantified using ImageJ software (version 10.8.1), and the migration rate was calculated as:

Migration rate (%)$\text{=}\frac{\text{wound area (0 h) - wound area (24 h)}}{\text{wound area (0 h)}}$× 100%

### ***S1.11.2 Transwell assay***

PC-9 and NCI-H1975 cells were pretreated with PBS (control), Cu-DMSA-PEG (30 μg/mL), or Cu-DMSA-HA (30 μg/mL) in complete medium for 24 h. After treatment, the cells were harvested and resuspended in serum-free medium. They were then seeded into the upper chambers of Transwell inserts with 8.0 μm pore size polycarbonate membranes (Cat. No. 3422; Corning, Corning, NY, USA) at a density of 5 × 10⁴ cells per well to perform migration assays. For the invasion assay, Matrigel (BL5597B; Corning, Corning, NY, USA) was diluted 1:8 in serum-free medium, and 60 μL of the diluted solution was added to the upper chamber. The chambers were then incubated at 37°C for 3 h to allow the formation of a thin gel layer prior to cell seeding. For both assays, the lower chambers were filled with complete medium containing 10% FBS as a chemoattractant.

After 24 h of incubation, non-migrated or non-invaded cells on the upper surface of the membrane were gently removed with a cotton swab. Cells on the lower surface were fixed with 4% paraformaldehyde for 15 min and stained with 0.1% crystal violet for 15 min. Images were then captured under a light microscope, and the number of migrated or invaded cells was quantified using ImageJ software (version 10.8.1).

## **S1.12 Western blot assay**

PC-9 and NCI-H1975 cells were treated with PBS (control), Cu-DMSA-PEG (30 μg/mL), or Cu-DMSA-HA (30 μg/mL) in complete medium for 48 h. After treatment, the cells were harvested and lysed using a total protein extraction kit (C510003-0050; Sangon, Shanghai, China) according to the manufacturer’s protocol. Protein concentrations were determined using a bicinchoninic acid (BCA) protein assay kit (P0010S; Beyotime, Shanghai, China).

Equal amounts of protein (10 μg per lane) were loaded and separated by sodium dodecyl sulfate–polyacrylamide gel electrophoresis (SDS-PAGE) using 10% gels for CD44 and 12.5% gels (PG112; Epizyme, Shanghai, China) for GPX4. Proteins were subsequently transferred onto polyvinylidene difluoride (PVDF) (0.45 μm; IPVH00010; Millipore, Billerica, MA, USA) membranes that had been pre-activated in methanol for 10 min. Following the transfer, the membranes were blocked with a rapid blocking buffer (PS108P; Epizyme, Shanghai, China) for 15 min at room temperature. The membranes were then incubated overnight at 4°C with primary antibodies diluted 1:1000: anti-CD44 (Cat No. 15675-1-AP; Proteintech, Wuhan, China) and anti-GPX4 (A02059-1; Boster, Wuhan, China). After washing three times with Tris-buffered saline containing 0.1% Tween-20 (TBST), the membranes were incubated with horseradish peroxidase (HRP)-labeled Goat Anti-Rabbit IgG (H+L) (1:2000; BA1039; Boster, Wuhan, China) at room temperature for 1.5 h. Following three additional TBST washes, protein bands were visualized using the Omni-enhanced chemiluminescence (ECL) ultra-sensitive chemiluminescence substrate (SQ201; Epizyme, Shanghai, China) and imaged with a Tanon imaging system (Tanon 5200CE; Tanon, Shanghai, China).

## **S1.13 Measurement of GSH and oxidized glutathione (GSSG) levels**

PC-9 and NCI-H1975 cells were treated with PBS (control), Cu-DMSA-PEG (30 μg/mL), or Cu-DMSA-HA (30 μg/mL) in complete medium for 24 h. Following treatment, cells were harvested and lysed using a GSH/GSSG assay kit (S0053; Beyotime, Shanghai, China) in accordance with the manufacturer’s protocol. Lysates were centrifuged at 10,000 × g for 10 min at 4 °C, and supernatants were collected for further analysis. Total glutathione levels were determined by mixing samples with the assay working solution and incubating at room temperature. For specific quantification of GSSG, GSH was masked with a scavenger reagent, and GSSG levels were measured via an enzymatic recycling assay. Absorbance was recorded using a microplate reader, and the concentrations of GSH and GSSG were calculated against a standard curve. The GSH/GSSG ratio was used to assess the cellular redox status.

**S1.14 C11-BODIPY Staining**

Lipid peroxidation was detected using the fluorescent probe C11-BODIPY 581/591 (S0043S; Beyotime, Shanghai, China). PC-9 and NCI-H1975 cells were treated with PBS, Cu–DMSA–PEG (30 μg/mL), Cu–DMSA–HA (30 μg/mL), DMSO, Cu–DMSA–PEG+ferrostatin-1 (Fer-1), or Cu–DMSA–HA+Fer-1. Fer-1 was dissolved in DMSO to prepare a 2 mM stock solution. Cells were pretreated with Fer-1 (2 μM) for 1 hour prior to the addition of NPs, followed by co-incubation for 24 h. After treatment, fluorescence images were acquired using an inverted fluorescence microscope (NIB-610; Nikon, Tokyo, Japan). Lipid ROS levels were quantified by flow cytometry (LSRFortessa; BD Biosciences, USA). Cell viability was measured using a microplate reader (Infinite 200 Pro; Tecan, Männedorf, Switzerland).

**S1.15 Half-maximal inhibitory concentration (IC₅₀) Determination**

PC-9 and NCI-H1975 cells were seeded in 96-well plates at a density of 8 × 10³ cells per well and allowed to adhere overnight. Cells were treated with various concentrations of Cu–DMSA–HA NPs (0.001, 0.01, 0.1, 1, 10, and 100 μg/mL) or cisplatin (0.001, 0.01, 0.1, 1, 10, and 100 μM) for 72 h. Cell viability was assessed using the CCK-8 assay. The IC₅₀ was calculated by nonlinear regression analysis using GraphPad Prism (version 10.1.2).

***S1.15.1 IC₅₀-Normalized Dose Comparison Assay***

To compare cytotoxicity within clinically relevant dosing ranges, PC-9 and NCI-H1975 cells were treated with Cu–DMSA–HA or cisplatin at 0.5×, 1×, 1.5×, and 2× of their respective IC₅₀ values. Cell viability was measured at 24 h , 48 h , and 72 h post-treatment using the CCK-8 assay.

***S1.15.2 Fixed-Dose Comparison Assay***

In the fixed-dose comparison experiment, Cu–DMSA–HA was applied at a concentration of 30 μg/mL, a dose previously verified to be non-toxic to normal lung epithelial cells. Cisplatin was applied at its IC₅₀ concentration. Cell viability was determined at 24 h , 48 h , and 72 h after treatment using the CCK-8 assay.

## **S1.16 Transcriptomic RNA sequencing (RNA-seq) analysis**

### ***S1.16.1 Transcriptomic RNA-seq***

PC-9 cells were seeded in 6-well plates at a density of 2 × 10⁵ cells per well and incubated for 24 h. The culture medium was subsequently replaced with fresh medium containing either PBS (control) or Cu-DMSA-HA (case; 30 μg/mL), followed by a further 48-hour incubation. Total RNA was isolated using TRIzol reagent (Thermo Fisher Scientific, USA) according to the manufacturer’s instructions. Extracted RNA samples were then processed for mRNA transcriptome sequencing on the Illumina platform (APExBIO, Shanghai, China).

### ***S1.16.2 Differentially expressed genes (DEGs) analysis***

DEGs between the Cu-DMSA-HA group and the control group were identified using the “limma” package in R (version 4.1.3). Genes with an adjusted p value < 0.05 and an absolute $\text{log}_{2}$ fold change (|log₂FC|) > 1 were defined as significantly differentially expressed.

### ***S1.16.3 Gene Ontology (GO) and Kyoto Encyclopedia of Genes and Genomes (KEGG) enrichment analysis***

GO and KEGG enrichment analyses were performed to identify significantly enriched biological processes (BP), cellular components (CC), molecular functions (MF), and pathways related to metabolism and signal transduction. DEGs with log₂FC > 1 were defined as upregulated, while those with log₂FC < –1 was defined as downregulated. GO and KEGG analyses were conducted using the “clusterProfiler” package in R (version 4.1.3).

## **S1.17 Single-cell RNA sequencing (scRNA-seq) analysis**

### ***S1.17.1 ScRNA-seq data acquisition and processing***

Human non-small cell lung cancer (NSCLC) scRNA-seq data (GSE207422) were obtained from the Gene Expression Omnibus (GEO, https://www.ncbi.nlm.nih.gov/geo/) database. Datasets were selected based on the inclusion criteria of samples collected prior to surgery without exposure to chemotherapy, targeted therapy, immunotherapy, or other biological treatments. Data integration and batch effect correction were performed using the “Harmony” R package. Subsequent quality control, normalization, visualization, dimensionality reduction, and clustering were conducted with the “Seurat” R package. Quality control criteria were applied to exclude low-quality cells based, defined by the following thresholds: (1) total unique molecular identifiers (UMIs) ≤ 1,000; (2) number of detected genes ≤ 500; (3) mitochondrial gene percentage > 20%. Cells failing to meet these criteria were excluded, while the remaining high-quality cells were retained for downstream analysis.

Gene expression data were normalized, followed by principal component analysis (PCA) for dimensionality reduction. Graph-based clustering was performed to identify cell subpopulations, and the results were visualized using uniform manifold approximation and projection (UMAP).

### ***S1.17.2 Cellular subpopulation annotation***

Cell subpopulations were annotated by identifying cluster-specific marker genes using the “FindAllMarkers” function in Seurat. Subsequent automated cell type prediction was then performed with the “SingleR” R package, employing the HumanPrimaryCellAtlasData as the reference dataset. To improve annotation accuracy, predictions were manually refined by cross-referencing established marker genes from the literature.

### ***S1.17.3 Copy number variation (CNV) analysis***

To infer putative tumor cell subpopulations, CNV analysis was conducted using the “inferCNV” R package, with endothelial cells designated as the reference (normal) population. Relative gene expression levels for each target cell were computed against the reference group, enabling the generation of CNV heatmaps that visualize large-scale chromosomal amplifications and deletions at single-cell resolution.

### ***S1.17.4 AddModuleScore***

Gene sets related to DNA replication, cell cycle, and ferroptosis were obtained from the Molecular Signatures Database (MSigDB; https://www.gsea-msigdb.org/gsea/msigdb). The “AddModuleScore” function in the Seurat R package was applied to assess the activity of these gene sets at the single-cell level. This function calculates a module score for each cell by averaging the expression levels of genes within the gene set and subtracting the average expression of control gene sets with comparable expression levels.

## **S1.18 Animal experiments**

This study was reviewed and approved by the Ethics Committee of Shanghai Pulmonary Hospital (Approval No. K22-390) and conducted in accordance with the institutional guidelines for the care and use of laboratory animals, consistent with the National Institutes of Health Guide for the Care and Use of Laboratory Animals. Six-week-old BALB/c nude mice were purchased from Model Organisms Center (Shanghai, China). The mice were housed in a specific pathogen-free (SPF) facility under controlled conditions: 21 – 22°C temperature, 39 – 50 % humidity, and a 12-hour light/dark cycle.

### ***S1.18.1 Hemocompatibility assay***

Whole blood (1 mL) was collected from the BALB/c nude mice and centrifuged at 2,000 rpm for 10 min to isolate red blood cells (RBCs). The RBCs were washed and diluted with PBS to a final volume of 10 mL. For the hemolysis assay, 0.2 mL of the diluted RBC suspension was mixed with 0.8 mL of deionized water (positive control), PBS (negative control), or Cu-DMSA-HA at various concentrations (0.01, 0.05, 0.1, 0.2, 0.5, 1, 2, and 5 mg/mL). All mixtures were incubated at room temperature for 4 h. The morphology and lysis of RBCs were subsequently observed and recorded to evaluate the hemocompatibility of Cu-DMSA-HA.

### ***S1.18.2 Tissue targeting distribution***

BALB/c nude mice were subcutaneously injected with 2x10^6^ PC-9 cells into the right flank. Once tumor volumes reached approximately 100 mm³, mice were randomly allocated into three groups (n = 5 per group) and intravenously administered 100$\text{μL}$ of free Cy5 (10 nmol), Cy5-labeled Cu-DMSA-PEG NPs (2 mg/kg, containing 10 nmol Cy5), or Cy5-labeled Cu-DMSA-HA NPs (2 mg/kg, containing 10 nmol Cy5). Whole-body fluorescence imaging was performed at 0, 1, 2, 4,6, 8, and 24 h post-injection using an *in vivo* imaging system (IVIS) imaging (NightOwl II LB 983; Berthold, Germany) to assess biodistribution and tumor-targeting capabilities of the nanomaterials. At 24 h, 48 h, and 72 h post-injection, mice were sacrificed, and major organs including the heart, liver, spleen, lungs, kidneys, and tumors were harvested for *ex vivo* fluorescence imaging. Quantitative analysis of radiant efficiency was performed using consistent region-of-interest (ROI) settings to assess nanoparticle accumulation and clearance in tumor and non-target tissues. In addition, fluorescence imaging of tissue cryosections was performed to visualize the cellular-level distribution of Cy5-labeled NPs.

***S1.18.3 Pharmacokinetic (PK) Study***

To evaluate the *in vivo* PK behavior of the NPs, Cy5-labeled Cu-DMSA-PEG and Cu-DMSA-HA NPs were administered intravenously (IV) to BALB/c mice (female, 6 weeks) at a dose of 2 mg/kg. At predetermined time points (0, 0.083, 0.25, 0.5, 1, 2, 4, 8, 24, and 48 h) post-injection, approximately 10 μL of blood was collected from the retro-orbital plexus into heparinized tubes (n = 3 mice per group per time point). Plasma was obtained by centrifugation at 4,000 rpm for 10 min and stored at –80 °C until analysis.

The Cu concentration in plasma was quantified by ICP–OES (Agilent 7800) after acid digestion with 1% HNO₃. PK parameters, including maximum plasma concentration (C_max_), time to reach C_max_ (T_max_), terminal elimination half-life (t₁/₂), mean residence time (MRT), area under the plasma concentration–time curve (AUC₀–_t_ and AUC₀–∞), volume of distribution (Vss), and plasma clearance (CL), were calculated using noncompartmental analysis (NCA) performed in GraphPad Prism (version 10.1.2).

### ***S1.18.4 In Vivo therapeutic efficacy in subcutaneous xenograft nude mice***

PC-9 cells were subcutaneously injected into the right flank of BALB/c nude mice at 2 × 10⁶ cells each. When the tumor volume reached approximately 50 mm³, mice were randomly divided into three groups (n = 5 per group): PBS, Cu-DMSA-PEG, or Cu-DMSA-HA. Each group received a tail vein injection of 100 $\text{μ}$L PBS or nanomaterial solutions at a concentration of 2 mg/kg. Treatments were administered once every three days for a total of five administrations. Tumor volumes and mouse body weights were monitored throughout the study to evaluate the treatment response and systemic tolerance. Tumor volume was calculated using the formula:

Volume =$\frac{\text{(width² × length) }}{2}$

End-point criteria included the following: (1) tumor volume exceeding 1500 mm³; (2) more than 15% body weight loss from baseline or significant deterioration in health; (3) a maximum study duration of 25 days. At the end of the study, mice were euthanized, and tumors were excised, measured, and weighed.

### ***S1.18.5 In Vivo therapeutic efficacy in a nude mouse lung metastasis model***

PC-9 cells were injected into BALB/c nude mice via tail vein at 3 × 10^5^ cells each. Five days after injection and micro-metastatic lesions had formed in the lungs, the mice were randomly divided into three groups (n = 5). Mice were administered tail vein injections of either 100 μL PBS or nanoparticle solutions (Cu-DMSA-PEG or Cu-DMSA-HA) at a dose of 2 mg/kg. Injections were given once every three days, for a total of five doses. Three days after the final injection, mice were euthanized, and lung tissues were collected for H&E staining. Tumor burden was quantified by calculating the proportion of tumor area relative to the total lung cross-sectional area using ImageJ software to assess therapeutic efficacy.

***S1.18.6 In vivo therapeutic efficacy of Cu–DMSA–HA versus cisplatin in a subcutaneous xenograft nude mouse model***

PC-9 cells (2 × 10⁶ per mouse) were subcutaneously injected into the right flank of BALB/c nude mice. When the tumor volume reached approximately 50 mm³, mice were randomly divided into two groups (n = 5 per group): Cu–DMSA–HA and cisplatin. Mice received tail vein injections of Cu–DMSA–HA nanoparticles (2 mg/kg) or cisplatin (2 mg/kg) every three days, for a total of five administrations.

Tumor volumes and body weights were monitored throughout the treatment period to evaluate antitumor efficacy and systemic tolerance. After the fifth treatment cycle, mice were euthanized, and tumors were harvested for further analysis, including tumor volume, weight, and histological evaluation.

***S1.18.7 Immunohistochemistry (IHC) assay***

Following harvesting tumors, tissue sections were deparaffinized and subjected to antigen retrieval by overnight incubation in citrate buffer at 60°C. Endogenous peroxidase activity was quenched with 3% H_2_O_2_ for 10 min. To prevent non-specific binding, sections were blocked with 5% bovine serum albumin (BSA) for 1 hour at room temperature. Primary antibody incubation was performed overnight at 4°C, followed by a 1-hour incubation with a incubated with HRP-conjugated secondary antibody at room temperature. Signal detection was carried out using DAB substrate, and nuclei were counterstained with hematoxylin. Images were obtained using a light microscope.

### ***S1.18.8 Immunofluorescence (IF) assay***

Tissue sections were first deparaffinized and subjected to antigen retrieval under the same conditions as described for IHC. Following antigen retrieval, sections were permeabilized with 0.1% Triton X-100 for 20 min and then blocked with 5% BSA at room temperature for 1 hour. Primary antibody incubation was performed overnight at 4°C, followed by PBS washes and a 1-hour incubation with the fluorescently labeled secondary antibody at room temperature. Nuclei were counterstained with DAPI. Fluorescence images were captured using a fluorescence microscope.

## **S1.19 Statistical analysis**

Statistical analysis was performed using R (version 4.1.3) and GraphPad Prism (version 10.1.2). Comparisons between two groups were conducted using Student’s t-test or Wilcoxon rank-sum test, and comparisons among three or more groups were performed using one-way analysis of variance (ANOVA) followed by appropriate post hoc tests. Data are presented as mean $\text{±}$ standard deviation (SD). Statistical significance was defined as *p<0.05, **p<0.01, ***p<0.001, and ****p<0.0001.

**S2 Supplementary Figures**


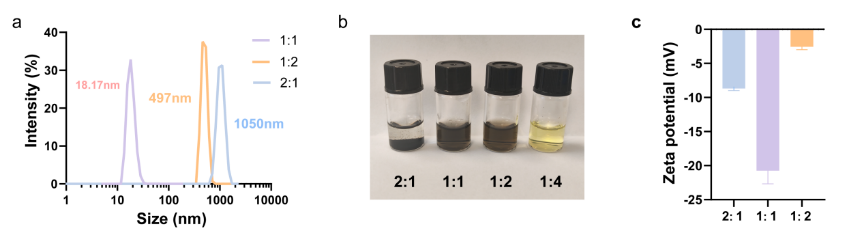


**Fig. S1 (a)** The size distribution, **(b)** the photo and **(c)** zeta potentials of nanoparticles (NPs) under different synthesis conditions


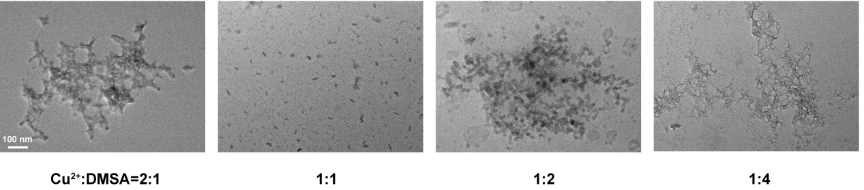


**Fig. S2** Transmission electron microscopy (TEM) images of the nanoparticles formed by different proportions of Cu^2+^ and dimercaptosuccinic acid (DMSA). Scale bar: 100 nm


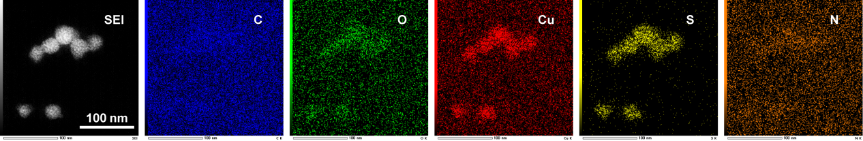


**Fig. S3** High-angle annular dark scanning transmission electron microscopy (HAADF-STEM) image and elemental mapping images of the elements in the Cu-DMSA-PEG. Scale bar: 100 nm


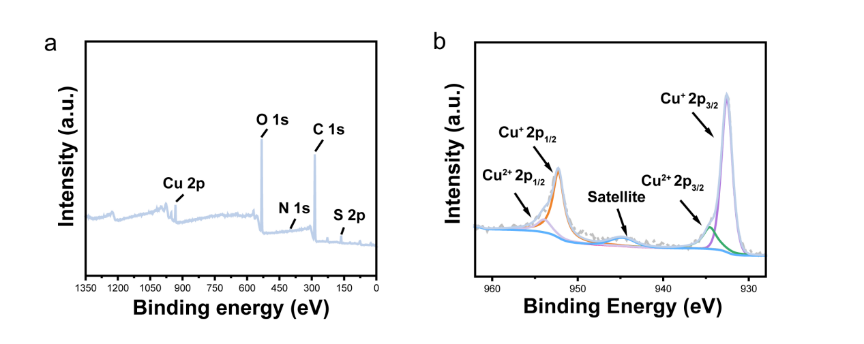


**Fig. S4 (a)** The X-ray photoelectron spectroscopy (XPS) survey scan and Cu 2p scan **(B)** of Cu-DMSA-PEG


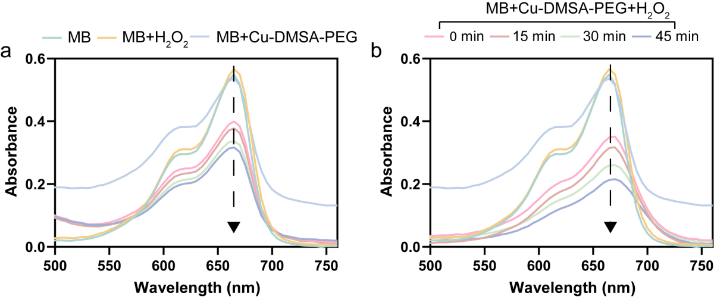


**Fig. S5 (a)** Time-dependent ultraviolet–visible (UV–Vis) spectra of methylene blue (MB) degradation catalyzed by Cu-DMSA-PEG and **(b)** Cu-DMSA-PEG + glutathione (GSH)


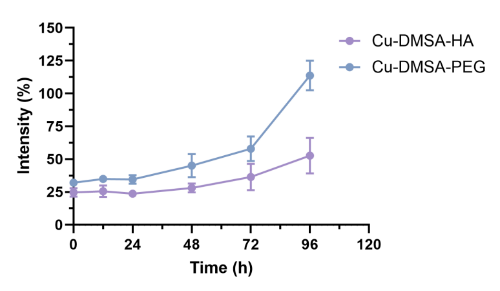


**Fig. S6** The stability of Cu-DMSA-HA and Cu-DMSA-PEG NPs in phosphate-buffered saline (PBS)


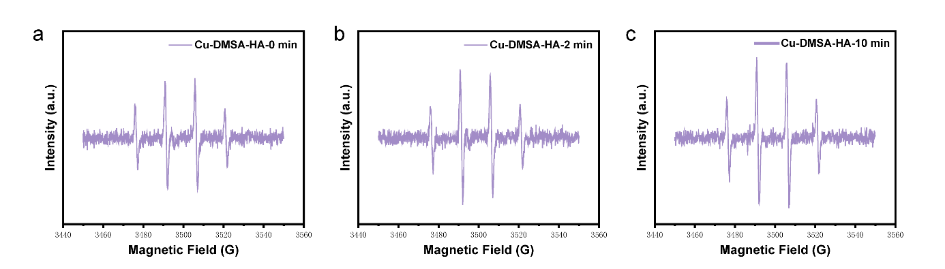


**Fig. S7** EPR spectrum of ROS generation by Cu-DMSA-HA NPs in **(a)** 0 min, **(b)** 2 min and **(c)** 10 min


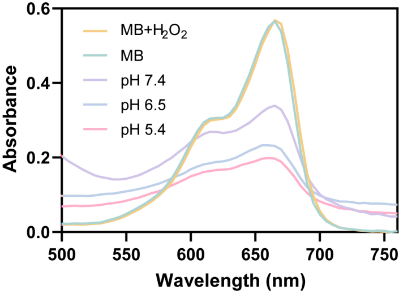


**Fig. S8** UV-Vis spectra of MB degradation after incubation with Cu-DMSA-HA at pH 5.5, 6.5 and 7.4 in the presence of H_2_O_2_


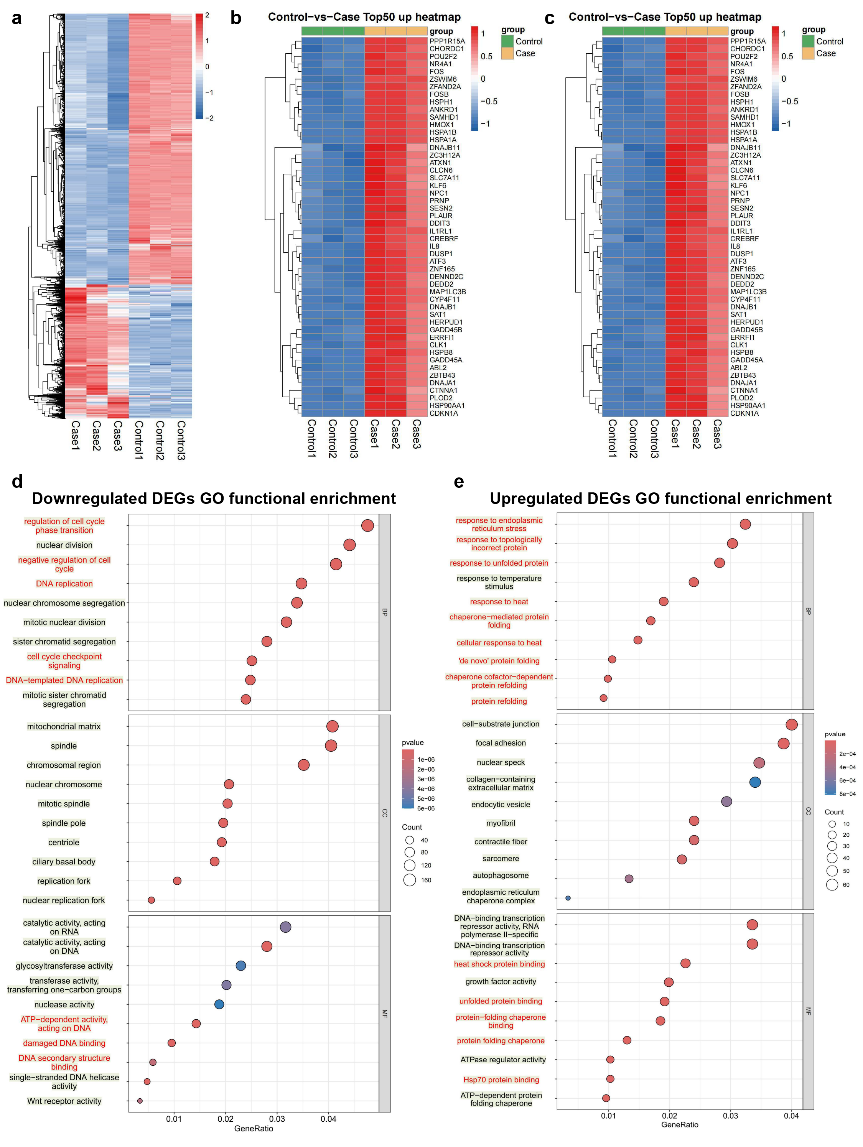


**Fig. S9** Transcriptomic RNA sequencing (RNA-seq) analysis revealing the antitumor mechanisms of Cu-DMSA-HA. **(a)** Heatmap displaying differentially expressed genes (DEGs) between Cu-DMSA-HA treatment group (case) and PBS group (control). **(b)** Heatmap illustrating the top 50 downregulated DEGs in the case group compared with the control group. **(c)** Heatmap illustrating the top 50 upregulated DEGs in the case group compared with the control group. **(d)** Bubble plot showing Gene Ontology (GO) enrichment analysis of DEGs downregulated in the case group compared to the control group. **(e)** Bubble plot showing GO enrichment analysis of DEGs upregulated in the case group compared to the control group


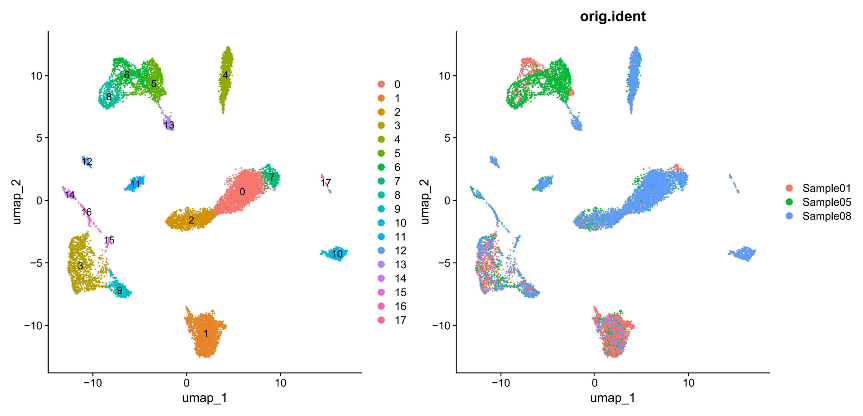


**Fig. S10** UMAP visualization of single-cell RNA sequencing (scRNA-seq) data from non-small cell lung cancer (NSCLC) patient samples following batch effect correction using Harmony integration


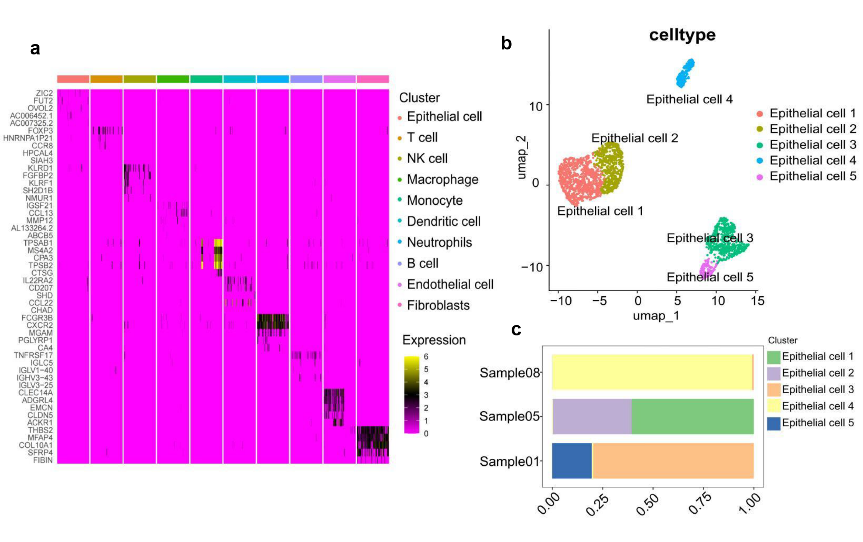


**Fig. S11 (a)** Heatmap displaying the top five marker genes for each identified cell cluster. **(b)** UMAP plot showing the five epithelial cell subclusters identified after further dimensionality reduction and clustering of epithelial cells. **(c)** Bar graph illustrating the distribution of epithelial cell subclusters across different NSCLC patient samples.


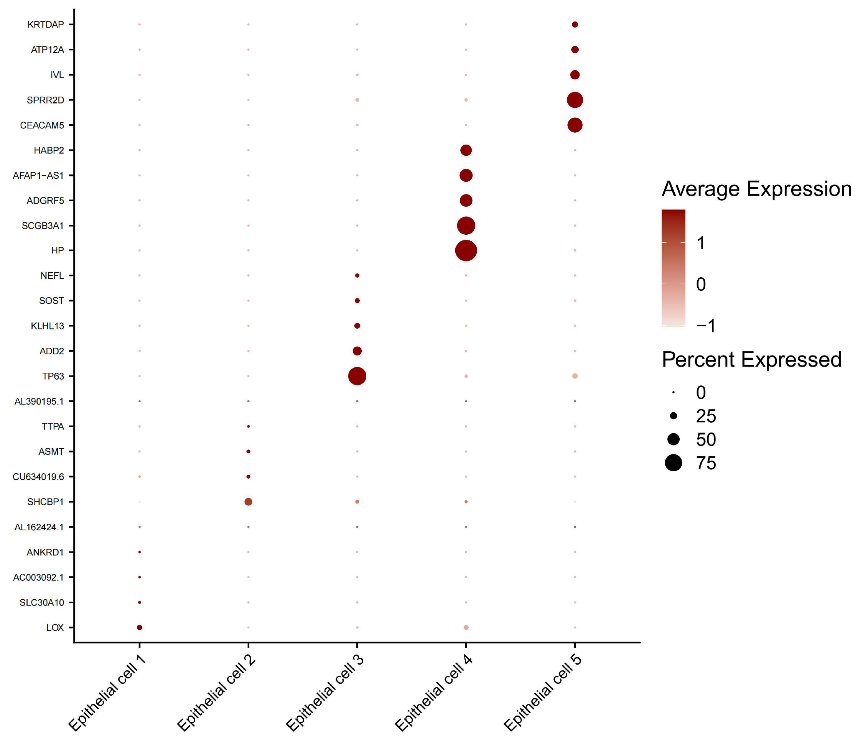


**Fig. S12** Bubble plot displaying the top five marker genes for epithelial cell subclusters


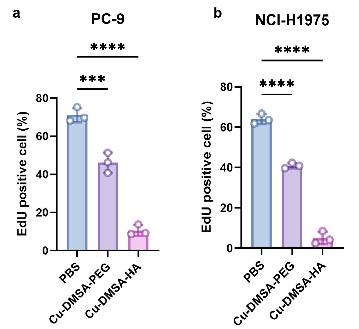


**Fig. S13** 5-ethynyl-2’-deoxyuridine (EdU) assay quantification analyzing comparing the DNA replication capabilities of **(a)** PC-9 and **(b)** NCI-H1975 cells following treatment with PBS, Cu-DMSA-PEG, or Cu-DMSA-HA (n = 3; mean ± standard deviation [SD]). One-way analysis of variance (ANOVA) followed by Tukey’s post hoc test. ***P < 0.001; ****P < 0.0001


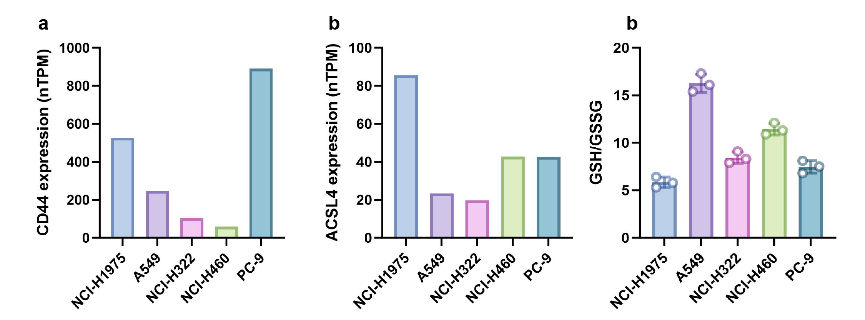


**Fig. S14** **(a)** CD44 and **(b)** ACSL4 mRNA expression levels [normalized Transcripts Per Million (nTPM)] in five NSCLC cell lines (PC-9, NCI-H460, NCI-H322, A549, and NCI-H1975) retrieved from the Human Protein Atlas (HPA, https://www.proteinatlas.org/). **(c)** Intracellular GSH/oxidized glutathione (GSSG) ratios measured via biochemical assays, reflecting the antioxidative capacity of each cell line


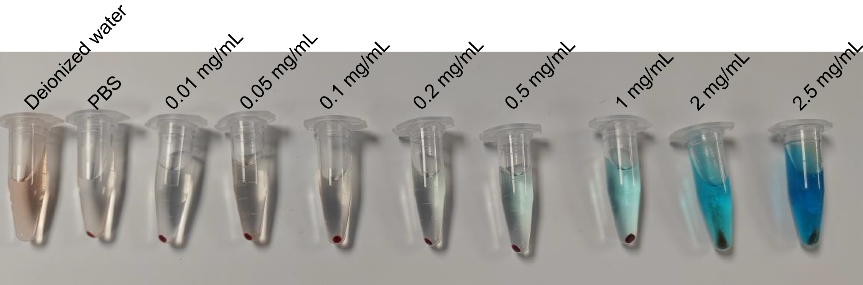


**Fig. S15** Hemolysis assay of Cu-DMSA-HA at different concentrations


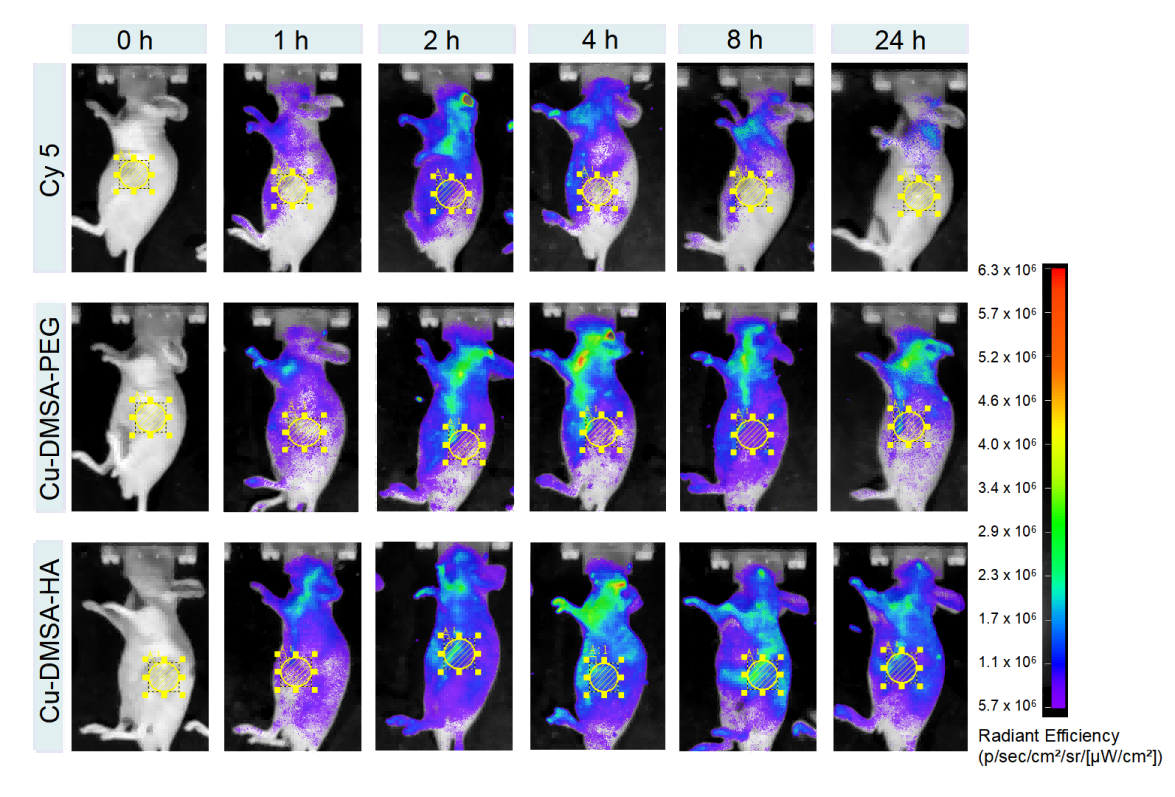


**Fig. S16** *In vivo* fluorescence images of mice at 0, 1, 2, 4, and 8 h after intravenous injection of Cy5, Cu-DMSA-PEG, or Cu-DMSA-HA


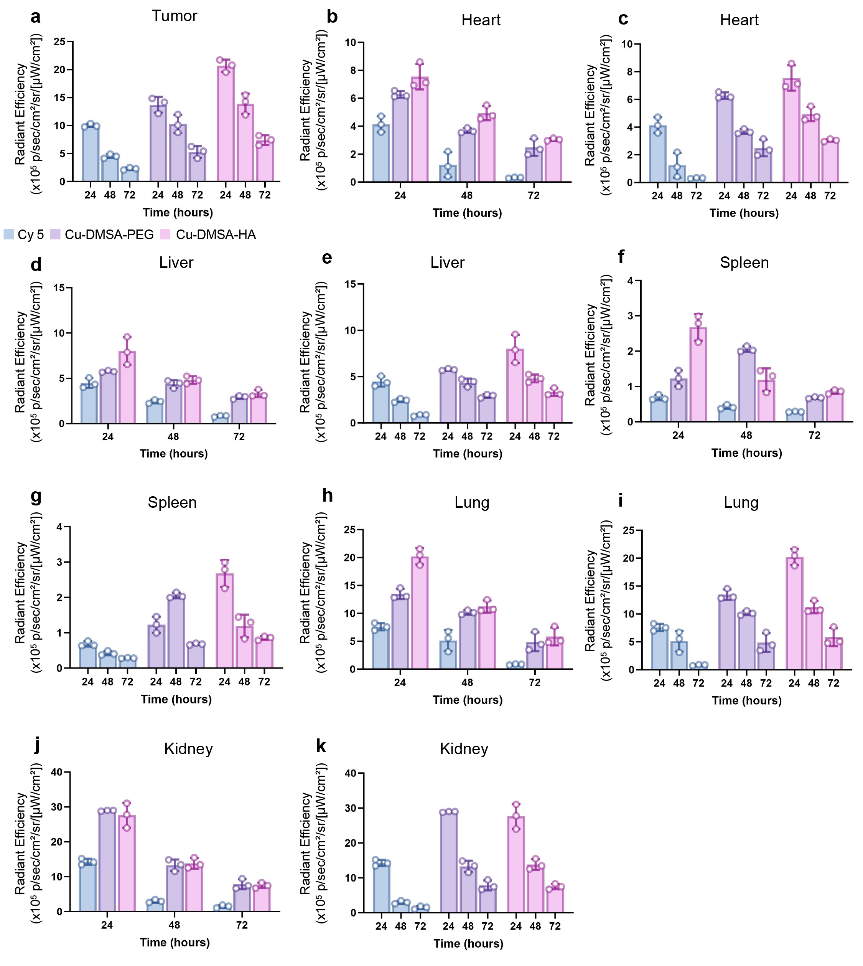


**Fig. S17 (a)** Quantitative analysis of tumor tissue radiant efficiency at 24 h, 48 h, and 72 h following intravenous injection of free Cy5, Cu-DMSA-PEG, or Cu-DMSA-HA NPs (n = 3; mean ± SD). **(b-k)** Quantitative analysis of radiant efficiency in heart, liver, spleen, lung, and kidneys tissues at 24 h, 48 h, and 72 h following intravenous injection of free Cy5, Cu-DMSA-PEG, or Cu-DMSA-HA NPs (n = 3; mean ± SD)


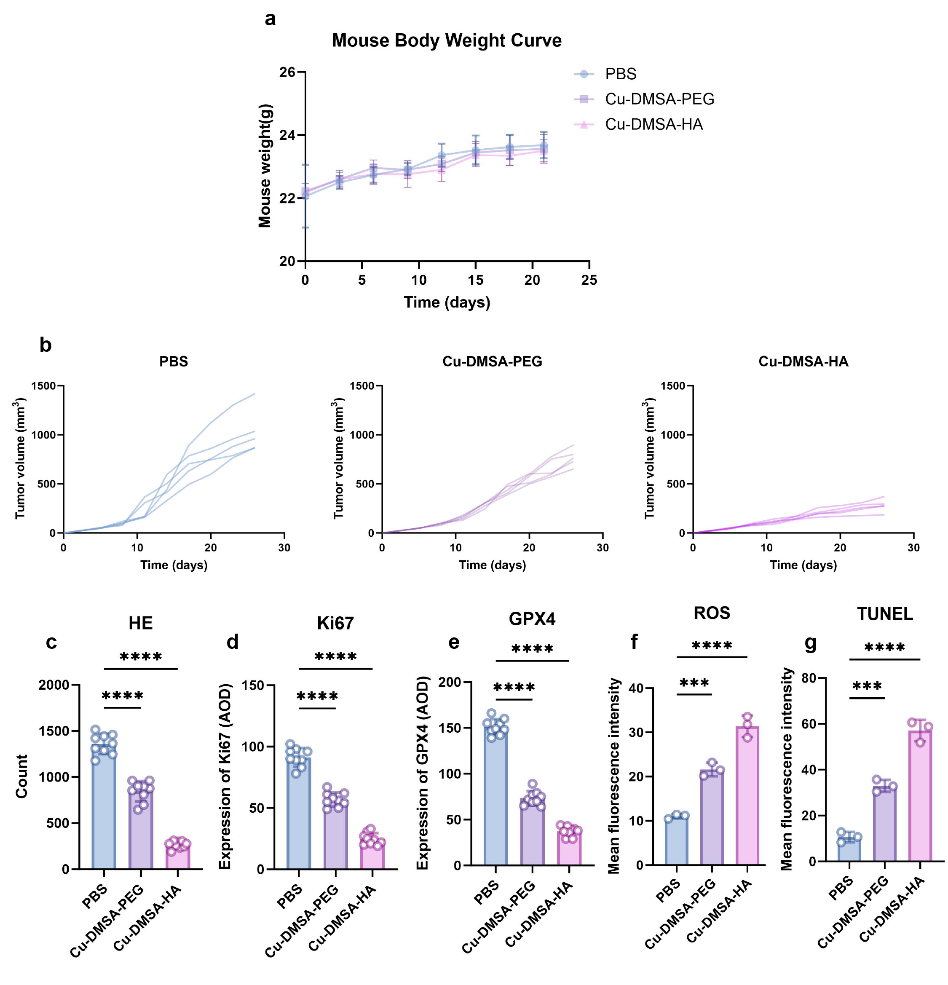


**Fig. S18 (a)** Body weight curves of mice during treatment (n = 5; mean ± SD). **(b)** Individual tumor growth curves of mice treated with PBS, Cu-DMSA-PEG, or Cu-DMSA-HA (n = 5; mean ± SD). Quantification analysis of **(c)** hematoxylin and eosin (H&E) staining and **(d-e)** immunohistochemistry (IHC) staining (Ki67 and GPX4) of tumor tissues following treatments with PBS, Cu-DMSA-PEG, or Cu-DMSA-HA (n = 3; mean ± SD). For each sample, three random high-power fields were selected for quantification. Statistical analysis was performed using one-way ANOVA followed by Tukey’s post hoc test. Quantification of mean fluorescence intensity of immunofluorescence (IF) staining by **(f)** ROS and **(g)** terminal deoxynucleotidyl transferase dUTP nick end labeling (TUNEL) after treatment of tumor tissues with PBS, Cu-DMSA-PEG or Cu-DMSA-HA. One-way ANOVA followed by Tukey’s post hoc test. ***P < 0.001; ****P < 0.0001


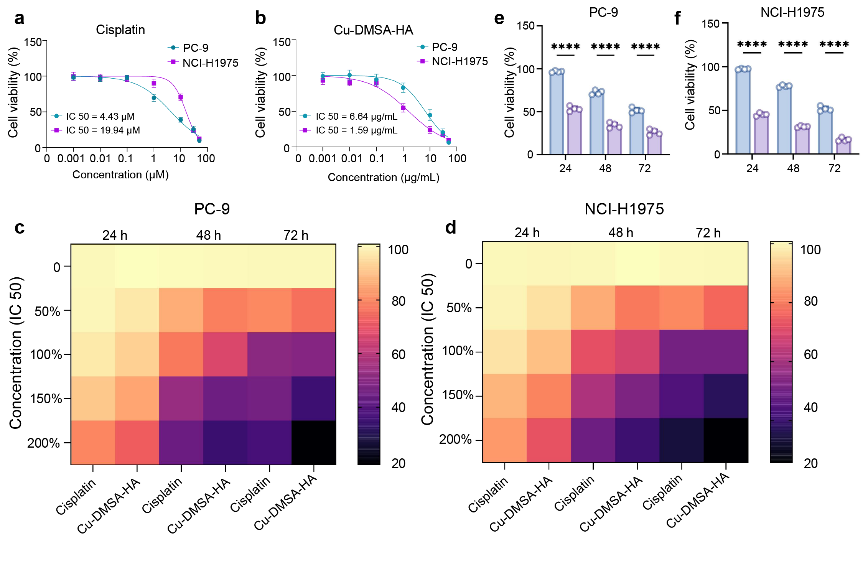


**Fig. S19** Comparative *in vitro* and *in vivo* antitumor efficacy of Cu–DMSA–HA NPs and cisplatin in NSCLC. **(a–b)** Dose–response curves of **(a)** cisplatin (0.001, 0.01, 0.1, 1, 10, and 100 μM) and **(a)** Cu–DMSA–HA (0.001, 0.01, 0.1, 1, 10, and 100 μg/mL) in PC-9 and NCI-H1975 cells after 72 h treatment. **(c–d)** Heatmaps showing changes in cell viability of **(c)** PC-9 and **(d)** NCI-H1975 cells treated with the half-maximal inhibitory concentration (IC_50_) -normalized doses (0.5×, 1×, 1.5×, and 2× IC₅₀) of Cu–DMSA–HA or cisplatin for 24 h, 48 h, and 72 h. **(e–f)** Fixed-dose comparison of **(e)** Cu–DMSA–HA (30 μg/mL) and **(f)** cisplatin (at IC₅₀ concentration) at different time points (24 h, 48 h, and 72 h) in PC-9 and NCI-H1975 cells


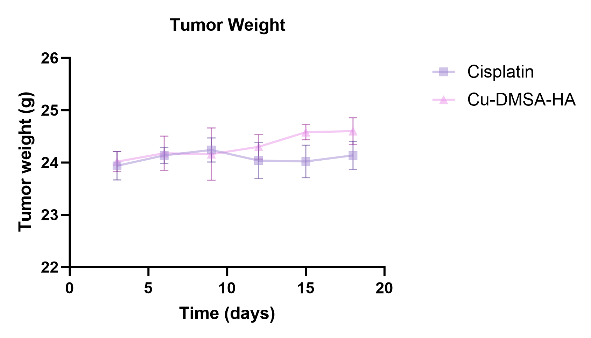


**Fig. S20** Body weight curves of mice treated with cisplatin or Cu-DMSA-HA


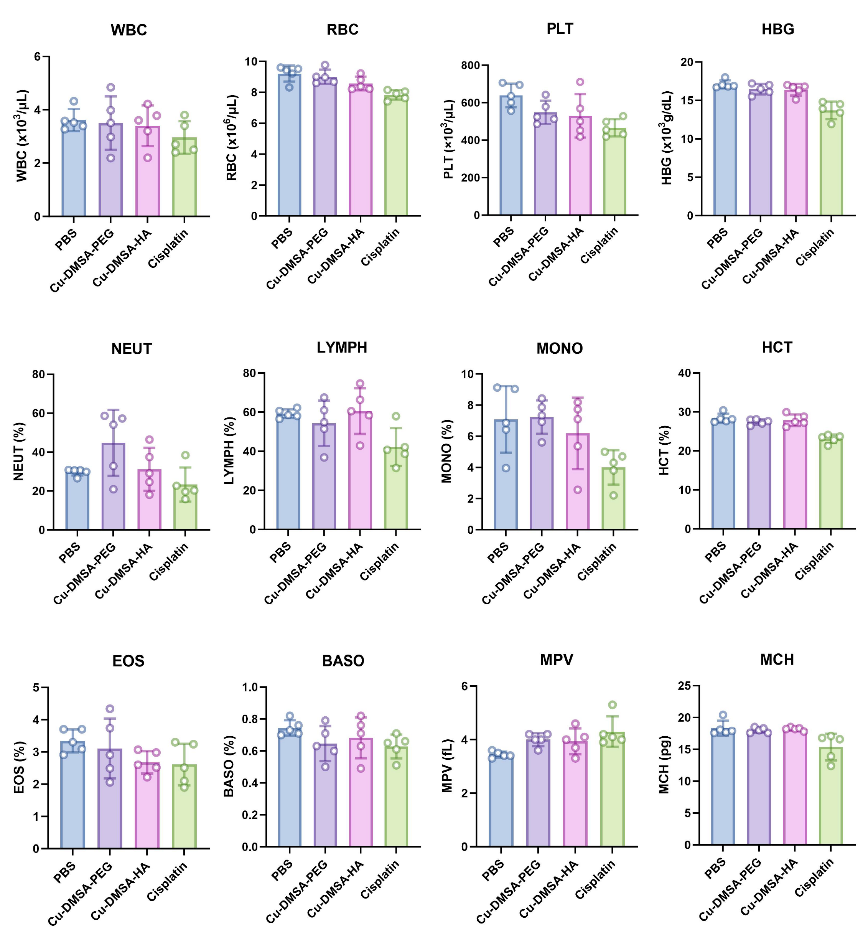


**Fig. S21** Complete blood count (CBC) analysis of mice after treatment with PBS, Cu-DMSA-PEG, Cu-DMSA-HA or cisplatin. WBC, white blood cell count; RBC, red blood cell count; PLT, platelet count; HBG, hemoglobin; NEUT, neutrophils; LYMPH, lymphocytes; MONO, monocytes; HCT, hematocrit; EOS, eosinophils; BASO, basophils; MPV, mean platelet volume; MCH, mean corpuscular hemoglobin


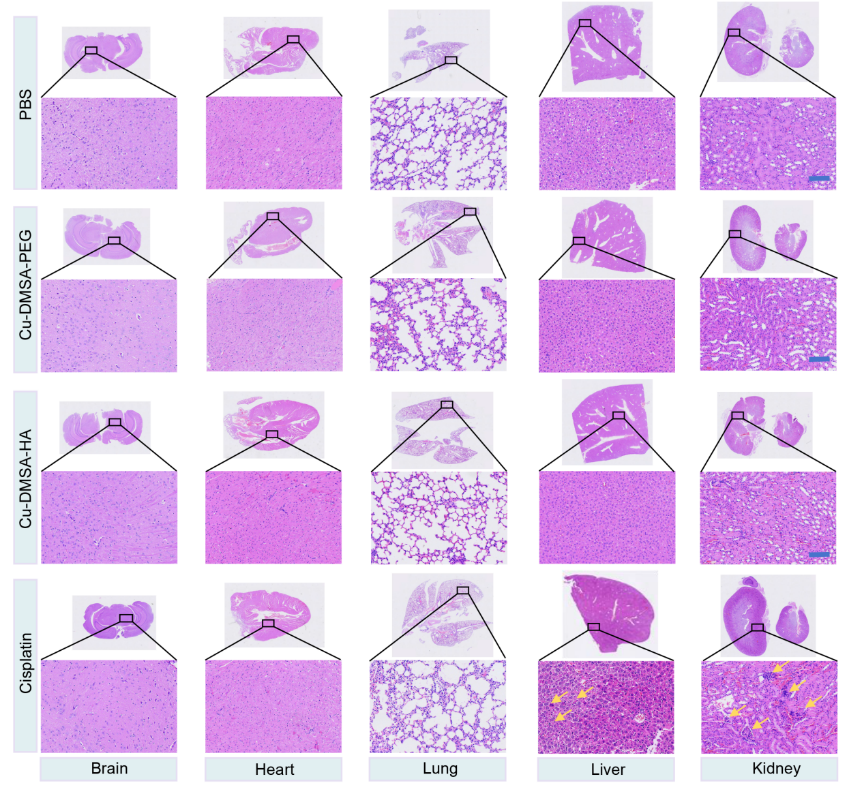


**Fig. S22** Histological evaluation of major organs by H&E staining after treatment with PBS, Cu-DMSA-PEG, Cu-DMSA-HA or cisplatin.

**S3 Supplementary Table**

**Table S1** Cu element was quantified by inductively coupled plasma optical emission spectrometry (ICP-OES)

| Sample | Cu |
| --- | --- |
| Cu-DMSA-HA | 29.82% |
| Cu-DMSA-PEG | 38.70% |

**Table S2** Transcriptomic RNA sequencing (RNA-seq) differentially expressed genes results

| ID | log2FoldChange | pval | padj | gene_name |
| --- | --- | --- | --- | --- |
| ENSG00000000460 | -1.176 | 3.711e-18 | 1.963e-17 | C1orf112 |
| ENSG00000001497 | -1.079 | 2.373e-23 | 1.595e-22 | LAS1L |
| ENSG00000001561 | -1.371 | 1.608e-10 | 5.589e-10 | ENPP4 |
| ENSG00000001617 | -1.346 | 6.4e-06 | 1.574e-05 | SEMA3F |
| ENSG00000002016 | -1.26 | 5.469e-11 | 1.963e-10 | RAD52 |
| ENSG00000002587 | -3.398 | 3.535e-16 | 1.693e-15 | HS3ST1 |
| ENSG00000002746 | 3.09 | 0.00522 | 0.009431 | HECW1 |
| ENSG00000002822 | -1.279 | 6.233e-24 | 4.313e-23 | MAD1L1 |
| ENSG00000003249 | -1.171 | 2.352e-11 | 8.648e-11 | DBNDD1 |
| ENSG00000003400 | -2.379 | 3.519e-30 | 3.159e-29 | CASP10 |
| ENSG00000003756 | 1.103 | 3.56e-31 | 3.342e-30 | RBM5 |
| ENSG00000003989 | -2.701 | 7.723e-21 | 4.627e-20 | SLC7A2 |
| ENSG00000004478 | 1.428 | 4.42e-45 | 6.883e-44 | FKBP4 |
| ENSG00000004660 | -1.499 | 3.842e-17 | 1.93e-16 | CAMKK1 |
| ENSG00000004776 | 3.736 | 1.052e-06 | 2.775e-06 | HSPB6 |
| ENSG00000004777 | -1.579 | 6.377e-13 | 2.571e-12 | ARHGAP33 |
| ENSG00000004799 | -1.596 | 9.662e-06 | 2.339e-05 | PDK4 |
| ENSG00000004975 | -1.307 | 2.604e-28 | 2.168e-27 | DVL2 |
| ENSG00000005001 | 2.116 | 8.506e-28 | 6.955e-27 | PRSS22 |
| ENSG00000005073 | -3.527 | 7.196e-08 | 2.078e-07 | HOXA11 |
| ENSG00000005108 | -1.805 | 1.025e-11 | 3.845e-11 | THSD7A |
| ENSG00000005189 | -1.225 | 4.189e-10 | 1.417e-09 | AC004381.6 |
| ENSG00000005243 | 1.641 | 0.01601 | 0.02684 | COPZ2 |
| ENSG00000005339 | 1.022 | 2.741e-11 | 1.003e-10 | CREBBP |
| ENSG00000005469 | -2.487 | 3.667e-59 | 8.632e-58 | CROT |
| ENSG00000005801 | 1.561 | 5.096e-20 | 2.94e-19 | ZNF195 |
| ENSG00000005844 | 3.688 | 4.65e-05 | 0.0001061 | ITGAL |
| ENSG00000005882 | -1.34 | 4.521e-16 | 2.152e-15 | PDK2 |
| ENSG00000005884 | -1.35 | 3.413e-35 | 3.814e-34 | ITGA3 |
| ENSG00000006015 | -1.463 | 2.147e-13 | 8.92e-13 | C19orf60 |
| ENSG00000006016 | 1.408 | 1.338e-06 | 3.493e-06 | CRLF1 |
| ENSG00000006025 | -3.798 | 1.237e-79 | 4.991e-78 | OSBPL7 |
| ENSG00000006282 | -1.038 | 3.056e-17 | 1.541e-16 | SPATA20 |
| ENSG00000006283 | 6.239 | 3.009e-17 | 1.519e-16 | CACNA1G |
| ENSG00000006459 | 2.36 | 5.51e-86 | 2.643e-84 | JHDM1D |
| ENSG00000006468 | -2.986 | 3.937e-05 | 9.037e-05 | ETV1 |
| ENSG00000006530 | -1.273 | 4.903e-30 | 4.376e-29 | AGK |
| ENSG00000006534 | -1.519 | 2.065e-21 | 1.276e-20 | ALDH3B1 |
| ENSG00000006555 | -2.601 | 1.287e-50 | 2.352e-49 | TTC22 |
| ENSG00000006606 | 4.204 | 1.338e-26 | 1.041e-25 | CCL26 |
| ENSG00000006607 | -1.134 | 5.737e-20 | 3.303e-19 | FARP2 |
| ENSG00000006634 | -2.749 | 5.538e-77 | 2.122e-75 | DBF4 |
| ENSG00000006652 | 1.647 | 2.926e-77 | 1.134e-75 | IFRD1 |
| ENSG00000006744 | -2.385 | 3.859e-99 | 2.444e-97 | ELAC2 |
| ENSG00000006756 | -1.273 | 6.927e-16 | 3.263e-15 | ARSD |
| ENSG00000006837 | 1.731 | 8.279e-13 | 3.316e-12 | CDKL3 |
| ENSG00000007202 | -1.149 | 9.904e-38 | 1.221e-36 | KIAA0100 |
| ENSG00000007237 | 1.699 | 0.0001225 | 0.0002679 | GAS7 |
| ENSG00000007314 | 3.657 | 1.087e-05 | 2.62e-05 | SCN4A |
| ENSG00000007350 | 3.523 | 0.003049 | 0.005691 | TKTL1 |
| ENSG00000007372 | -1.331 | 1.926e-09 | 6.233e-09 | PAX6 |
| ENSG00000007376 | -1.535 | 4.814e-25 | 3.497e-24 | RPUSD1 |
| ENSG00000007402 | 2.094 | 6.552e-11 | 2.34e-10 | CACNA2D2 |
| ENSG00000007520 | -1.022 | 9.778e-09 | 3.005e-08 | TSR3 |
| ENSG00000007541 | -1.77 | 8.459e-36 | 9.62e-35 | PIGQ |
| ENSG00000007866 | -1.323 | 3.864e-32 | 3.796e-31 | TEAD3 |
| ENSG00000007952 | 2.963 | 1.077e-08 | 3.297e-08 | NOX1 |
| ENSG00000007968 | -3.879 | 1.056e-69 | 3.369e-68 | E2F2 |
| ENSG00000008056 | 3.482 | 0.00273 | 0.005132 | SYN1 |
| ENSG00000008118 | 5.661 | 2.207e-09 | 7.103e-09 | CAMK1G |
| ENSG00000008128 | 1.4 | 2.702e-41 | 3.745e-40 | CDK11A |
| ENSG00000008130 | -1.441 | 3.565e-16 | 1.706e-15 | NADK |
| ENSG00000008277 | -2.375 | 1.271e-37 | 1.559e-36 | ADAM22 |
| ENSG00000008283 | -1.599 | 2.397e-17 | 1.215e-16 | CYB561 |
| ENSG00000008294 | 1.593 | 1.809e-81 | 7.687e-80 | SPAG9 |
| ENSG00000008311 | 3.198 | 7.284e-06 | 1.784e-05 | AASS |
| ENSG00000008323 | -2.65 | 2.044e-34 | 2.219e-33 | PLEKHG6 |
| ENSG00000008324 | -1.435 | 4.865e-09 | 1.531e-08 | SS18L2 |
| ENSG00000008516 | 4.694 | 2.188e-113 | 1.846e-111 | MMP25 |
| ENSG00000008517 | 4.634 | 7.762e-33 | 7.84e-32 | IL32 |
| ENSG00000008710 | -1.56 | 1.257e-23 | 8.559e-23 | PKD1 |
| ENSG00000008838 | -1.953 | 2.473e-59 | 5.905e-58 | MED24 |
| ENSG00000008853 | -2.511 | 6.009e-59 | 1.395e-57 | RHOBTB2 |
| ENSG00000009413 | -1.681 | 6.352e-57 | 1.381e-55 | REV3L |
| ENSG00000009724 | -2.563 | 4.143e-05 | 9.492e-05 | MASP2 |
| ENSG00000009780 | -2.283 | 3.04e-10 | 1.04e-09 | FAM76A |
| ENSG00000009950 | -1.595 | 1.862e-05 | 4.393e-05 | MLXIPL |
| ENSG00000009954 | -1.277 | 1.434e-39 | 1.876e-38 | BAZ1B |
| ENSG00000010072 | 1.362 | 2.907e-48 | 4.964e-47 | SPRTN |
| ENSG00000010165 | -1.265 | 4.658e-11 | 1.679e-10 | METTL13 |
| ENSG00000010219 | -2.187 | 4.33e-23 | 2.863e-22 | DYRK4 |
| ENSG00000010292 | -1.923 | 3.788e-77 | 1.462e-75 | NCAPD2 |
| ENSG00000010295 | 1.612 | 0.01771 | 0.02948 | IFFO1 |
| ENSG00000010310 | 2.282 | 2.764e-37 | 3.338e-36 | GIPR |
| ENSG00000010322 | -1.22 | 4.767e-20 | 2.755e-19 | NISCH |
| ENSG00000010379 | 4.367 | 1.731e-11 | 6.412e-11 | SLC6A13 |
| ENSG00000010671 | 3.195 | 7.39e-08 | 2.132e-07 | BTK |
| ENSG00000010704 | -2.078 | 2.677e-30 | 2.419e-29 | HFE |
| ENSG00000010803 | -1.055 | 2.849e-11 | 1.041e-10 | SCMH1 |
| ENSG00000010818 | 1.175 | 3.634e-20 | 2.112e-19 | HIVEP2 |
| ENSG00000011007 | -2.148 | 7.622e-70 | 2.437e-68 | TCEB3 |
| ENSG00000011021 | 2.856 | 2.074e-196 | 6.695e-194 | CLCN6 |
| ENSG00000011143 | -2.166 | 1.949e-22 | 1.255e-21 | MKS1 |
| ENSG00000011198 | 1.397 | 5.419e-25 | 3.926e-24 | ABHD5 |
| ENSG00000011332 | -1.57 | 0.0001278 | 0.000279 | DPF1 |
| ENSG00000011376 | -1.295 | 2.407e-19 | 1.345e-18 | LARS2 |
| ENSG00000011422 | 2.564 | 1.249e-199 | 4.369e-197 | PLAUR |
| ENSG00000011426 | -1.79 | 4.137e-25 | 3.012e-24 | ANLN |
| ENSG00000011478 | -1.473 | 1.105e-15 | 5.155e-15 | QPCTL |
| ENSG00000011523 | -2.421 | 2.701e-24 | 1.898e-23 | CEP68 |
| ENSG00000011638 | -1.59 | 8.163e-05 | 0.0001818 | TMEM159 |
| ENSG00000012048 | -2.183 | 2.556e-80 | 1.059e-78 | BRCA1 |
| ENSG00000012174 | -1.03 | 1.112e-27 | 9.056e-27 | MBTPS2 |
| ENSG00000012232 | -1.557 | 5.082e-20 | 2.933e-19 | EXTL3 |
| ENSG00000012660 | 1.524 | 9.002e-81 | 3.797e-79 | ELOVL5 |
| ENSG00000012779 | -1.1 | 2.609e-27 | 2.088e-26 | ALOX5 |
| ENSG00000013016 | -2.891 | 1.153e-18 | 6.25e-18 | EHD3 |
| ENSG00000013275 | 1.475 | 4.03e-59 | 9.448e-58 | PSMC4 |
| ENSG00000013375 | 1.204 | 3.735e-35 | 4.16e-34 | PGM3 |
| ENSG00000013441 | 3.705 | 0 | 0 | CLK1 |
| ENSG00000013503 | -2.121 | 1.292e-56 | 2.791e-55 | POLR3B |
| ENSG00000013523 | -2.695 | 1.02e-71 | 3.411e-70 | ANGEL1 |
| ENSG00000013573 | -2.67 | 2.096e-180 | 5.173e-178 | DDX11 |
| ENSG00000013810 | -1.9 | 3.528e-66 | 1.031e-64 | TACC3 |
| ENSG00000014123 | -1.78 | 1.818e-48 | 3.129e-47 | UFL1 |
| ENSG00000014138 | -2.388 | 1.035e-58 | 2.393e-57 | POLA2 |
| ENSG00000014919 | -1.26 | 1.283e-29 | 1.132e-28 | COX15 |
| ENSG00000015133 | -2.115 | 1.147e-31 | 1.106e-30 | CCDC88C |
| ENSG00000015520 | 5.052 | 1.752e-15 | 8.07e-15 | NPC1L1 |
| ENSG00000015532 | -2.451 | 2.638e-67 | 7.978e-66 | XYLT2 |
| ENSG00000015568 | 1.163 | 3.438e-24 | 2.403e-23 | RGPD5 |
| ENSG00000015676 | -1.256 | 1.027e-25 | 7.731e-25 | NUDCD3 |
| ENSG00000016082 | -2.243 | 0.006905 | 0.01227 | ISL1 |
| ENSG00000016402 | -3.651 | 1.248e-26 | 9.718e-26 | IL20RA |
| ENSG00000016864 | -2.728 | 2.181e-71 | 7.234e-70 | GLT8D1 |
| ENSG00000017260 | 1.135 | 4.642e-29 | 4.014e-28 | ATP2C1 |
| ENSG00000017373 | -1.802 | 1.554e-20 | 9.177e-20 | SRCIN1 |
| ENSG00000018408 | -1.383 | 6.815e-24 | 4.704e-23 | WWTR1 |
| ENSG00000018625 | 1.919 | 0.009226 | 0.01607 | ATP1A2 |
| ENSG00000019485 | -1.313 | 2.077e-11 | 7.668e-11 | PRDM11 |
| ENSG00000019549 | -3.13 | 1.018e-25 | 7.672e-25 | SNAI2 |
| ENSG00000020129 | -2.277 | 3.361e-53 | 6.584e-52 | NCDN |
| ENSG00000020256 | -1.499 | 1.168e-32 | 1.171e-31 | ZFP64 |
| ENSG00000020577 | 1.21 | 2.101e-46 | 3.411e-45 | SAMD4A |
| ENSG00000021762 | -1.81 | 4.977e-11 | 1.791e-10 | OSBPL5 |
| ENSG00000022267 | -1.543 | 0.01315 | 0.02236 | FHL1 |
| ENSG00000022976 | -2.27 | 4.433e-34 | 4.713e-33 | ZNF839 |
| ENSG00000023287 | 1.924 | 2.155e-49 | 3.815e-48 | RB1CC1 |
| ENSG00000023516 | -1.507 | 2.994e-32 | 2.955e-31 | AKAP11 |
| ENSG00000023697 | -1.103 | 3.566e-18 | 1.888e-17 | DERA |
| ENSG00000023892 | -2.408 | 4.422e-11 | 1.595e-10 | DEF6 |
| ENSG00000023909 | 2.024 | 9.272e-124 | 9.977e-122 | GCLM |
| ENSG00000024422 | -1.992 | 1.909e-24 | 1.351e-23 | EHD2 |
| ENSG00000024526 | -2.301 | 4.877e-50 | 8.775e-49 | DEPDC1 |
| ENSG00000025039 | -1.189 | 0.001149 | 0.002263 | RRAGD |
| ENSG00000025156 | -1.353 | 1.353e-15 | 6.273e-15 | HSF2 |
| ENSG00000025293 | -1.555 | 4.325e-45 | 6.741e-44 | PHF20 |
| ENSG00000025434 | -1.562 | 8.813e-17 | 4.343e-16 | NR1H3 |
| ENSG00000025770 | -1.029 | 7.576e-07 | 2.021e-06 | NCAPH2 |
| ENSG00000026559 | -2.601 | 5.522e-06 | 1.365e-05 | KCNG1 |
| ENSG00000026652 | 1.424 | 2.675e-14 | 1.164e-13 | AGPAT4 |
| ENSG00000026751 | 3.04 | 2.87e-14 | 1.247e-13 | SLAMF7 |
| ENSG00000026950 | -1.547 | 1.527e-14 | 6.714e-14 | BTN3A1 |
| ENSG00000027001 | -1.187 | 2.463e-17 | 1.247e-16 | MIPEP |
| ENSG00000028277 | 4.431 | 1.017e-163 | 2.031e-161 | POU2F2 |
| ENSG00000028528 | -1.51 | 3.509e-45 | 5.494e-44 | SNX1 |
| ENSG00000029534 | -1.372 | 0.0001423 | 0.0003091 | ANK1 |
| ENSG00000029639 | -1.581 | 5.695e-13 | 2.302e-12 | TFB1M |
| ENSG00000029993 | -1.444 | 2.777e-26 | 2.135e-25 | HMGB3 |
| ENSG00000030110 | -2.947 | 5.689e-58 | 1.284e-56 | BAK1 |
| ENSG00000030419 | -4.147 | 3.735e-82 | 1.62e-80 | IKZF2 |
| ENSG00000031081 | 1.807 | 1.947e-12 | 7.625e-12 | ARHGAP31 |
| ENSG00000031698 | 1.293 | 6.016e-37 | 7.152e-36 | SARS |
| ENSG00000032219 | 1.534 | 6.868e-16 | 3.236e-15 | ARID4A |
| ENSG00000033011 | -1.011 | 1.038e-05 | 2.506e-05 | ALG1 |
| ENSG00000033122 | 1.116 | 0.003752 | 0.006912 | LRRC7 |
| ENSG00000033170 | -1.392 | 1.11e-33 | 1.159e-32 | FUT8 |
| ENSG00000033627 | 1.539 | 2.297e-77 | 8.945e-76 | ATP6V0A1 |
| ENSG00000034533 | -1.201 | 2.041e-12 | 7.98e-12 | ASTE1 |
| ENSG00000034677 | 1.733 | 1.601e-50 | 2.921e-49 | RNF19A |
| ENSG00000034693 | -1.242 | 5.356e-21 | 3.245e-20 | PEX3 |
| ENSG00000035141 | -2.243 | 2.606e-91 | 1.43e-89 | FAM136A |
| ENSG00000035499 | -2.431 | 2.69e-55 | 5.568e-54 | DEPDC1B |
| ENSG00000035862 | -1.702 | 9.411e-91 | 5.031e-89 | TIMP2 |
| ENSG00000037042 | -1.295 | 1.374e-10 | 4.802e-10 | TUBG2 |
| ENSG00000037749 | -1.471 | 6.375e-08 | 1.847e-07 | MFAP3 |
| ENSG00000037757 | -2.154 | 2.968e-63 | 7.947e-62 | MRI1 |
| ENSG00000037897 | -1.419 | 5.295e-15 | 2.386e-14 | METTL1 |
| ENSG00000037965 | -2.653 | 5.815e-10 | 1.948e-09 | HOXC8 |
| ENSG00000038210 | -1.082 | 2.993e-09 | 9.552e-09 | PI4K2B |
| ENSG00000038219 | 1.783 | 2.049e-22 | 1.318e-21 | BOD1L1 |
| ENSG00000038358 | -1.383 | 2.927e-37 | 3.53e-36 | EDC4 |
| ENSG00000038382 | 1.433 | 8.238e-76 | 3.073e-74 | TRIO |
| ENSG00000038427 | 5.386 | 8.893e-07 | 2.36e-06 | VCAN |
| ENSG00000039523 | -1.694 | 1.485e-19 | 8.394e-19 | FAM65A |
| ENSG00000039987 | 4.272 | 4.022e-07 | 1.098e-06 | BEST2 |
| ENSG00000040531 | -1.028 | 1.055e-10 | 3.716e-10 | CTNS |
| ENSG00000040608 | -3.071 | 1.399e-09 | 4.577e-09 | RTN4R |
| ENSG00000040933 | -1.253 | 4.166e-23 | 2.761e-22 | INPP4A |
| ENSG00000041353 | -2.481 | 1.047e-114 | 8.966e-113 | RAB27B |
| ENSG00000041515 | 3.354 | 1.505e-13 | 6.309e-13 | MYO16 |
| ENSG00000041988 | -1.668 | 9.987e-08 | 2.856e-07 | THAP3 |
| ENSG00000042062 | 1.842 | 1.064e-05 | 2.568e-05 | FAM65C |
| ENSG00000042088 | -2.513 | 2.45e-46 | 3.969e-45 | TDP1 |
| ENSG00000042317 | -1.594 | 2.892e-05 | 6.707e-05 | SPATA7 |
| ENSG00000042429 | 1.37 | 1.163e-55 | 2.43e-54 | MED17 |
| ENSG00000043039 | -2.399 | 3.129e-12 | 1.21e-11 | BARX2 |
| ENSG00000043143 | -4.151 | 7.298e-172 | 1.612e-169 | PHF15 |
| ENSG00000043355 | -3.713 | 4.229e-33 | 4.321e-32 | ZIC2 |
| ENSG00000043591 | -3.304 | 0.002193 | 0.004178 | ADRB1 |
| ENSG00000044090 | -1.545 | 3.777e-26 | 2.895e-25 | CUL7 |
| ENSG00000044115 | 2.412 | 5.181e-174 | 1.16e-171 | CTNNA1 |
| ENSG00000044459 | -1.535 | 4.094e-12 | 1.572e-11 | CNTLN |
| ENSG00000044574 | 2.65 | 1.346e-123 | 1.43e-121 | HSPA5 |
| ENSG00000047230 | -2.29 | 6.261e-41 | 8.552e-40 | CTPS2 |
| ENSG00000047410 | -1.018 | 1.844e-07 | 5.168e-07 | TPR |
| ENSG00000047579 | -1.09 | 4.576e-06 | 1.14e-05 | DTNBP1 |
| ENSG00000047597 | -2.939 | 2.653e-19 | 1.481e-18 | XK |
| ENSG00000047634 | 1.488 | 6.774e-32 | 6.572e-31 | SCML1 |
| ENSG00000047648 | 2.416 | 0.01043 | 0.01803 | ARHGAP6 |
| ENSG00000047662 | 2.159 | 0.003113 | 0.005801 | FAM184B |
| ENSG00000047932 | -1.17 | 2.433e-17 | 1.233e-16 | GOPC |
| ENSG00000048028 | -1.545 | 2.006e-29 | 1.759e-28 | USP28 |
| ENSG00000048052 | 2.578 | 3.968e-92 | 2.213e-90 | HDAC9 |
| ENSG00000048140 | -1.107 | 1.193e-10 | 4.191e-10 | TSPAN17 |
| ENSG00000048342 | -1.841 | 4.938e-07 | 1.338e-06 | CC2D2A |
| ENSG00000048405 | 1.315 | 1.864e-22 | 1.201e-21 | ZNF800 |
| ENSG00000048462 | 4.133 | 0.0002991 | 0.0006292 | TNFRSF17 |
| ENSG00000048707 | -2.074 | 2.546e-35 | 2.857e-34 | VPS13D |
| ENSG00000048740 | 2.118 | 0.004287 | 0.007834 | CELF2 |
| ENSG00000049130 | -3.059 | 2.096e-135 | 2.665e-133 | KITLG |
| ENSG00000049192 | 1.451 | 0.0005749 | 0.001173 | ADAMTS6 |
| ENSG00000049239 | -2.89 | 6.447e-140 | 8.658e-138 | H6PD |
| ENSG00000049249 | 1.614 | 0.003076 | 0.005738 | TNFRSF9 |
| ENSG00000049283 | -4.249 | 1.065e-46 | 1.755e-45 | EPN3 |
| ENSG00000049541 | -1.387 | 1.615e-26 | 1.253e-25 | RFC2 |
| ENSG00000049759 | 2.616 | 1.205e-121 | 1.241e-119 | NEDD4L |
| ENSG00000049883 | -1.593 | 5.769e-17 | 2.869e-16 | PTCD2 |
| ENSG00000050344 | -2.15 | 3.939e-91 | 2.133e-89 | NFE2L3 |
| ENSG00000050438 | -2.013 | 5.152e-05 | 0.0001171 | SLC4A8 |
| ENSG00000050628 | 2.994 | 0.003406 | 0.006307 | PTGER3 |
| ENSG00000050730 | 2.305 | 0.01616 | 0.02708 | TNIP3 |
| ENSG00000051108 | 4.399 | 1.322e-283 | 8.533e-281 | HERPUD1 |
| ENSG00000051180 | -2.098 | 1.073e-14 | 4.762e-14 | RAD51 |
| ENSG00000051341 | -1.304 | 8.507e-37 | 1.006e-35 | POLQ |
| ENSG00000051382 | -1.08 | 1.991e-24 | 1.408e-23 | PIK3CB |
| ENSG00000051825 | -2.58 | 1.359e-88 | 6.914e-87 | MPHOSPH9 |
| ENSG00000053108 | -1.236 | 0.004665 | 0.008472 | FSTL4 |
| ENSG00000053438 | 2.24 | 7.12e-14 | 3.03e-13 | NNAT |
| ENSG00000053770 | -1.879 | 3.216e-59 | 7.613e-58 | AP5M1 |
| ENSG00000053900 | -1.292 | 1.706e-16 | 8.269e-16 | ANAPC4 |
| ENSG00000054179 | -1.569 | 6.521e-06 | 1.601e-05 | ENTPD2 |
| ENSG00000054267 | 1.757 | 1.384e-54 | 2.813e-53 | ARID4B |
| ENSG00000054277 | -1.069 | 1.641e-14 | 7.209e-14 | OPN3 |
| ENSG00000054282 | -1.107 | 3.808e-13 | 1.557e-12 | SDCCAG8 |
| ENSG00000054523 | 1.155 | 1.18e-36 | 1.381e-35 | KIF1B |
| ENSG00000054598 | -3.21 | 1.556e-70 | 5.051e-69 | FOXC1 |
| ENSG00000054690 | -1.613 | 4.301e-23 | 2.846e-22 | PLEKHH1 |
| ENSG00000054965 | -1.27 | 1.045e-27 | 8.522e-27 | FAM168A |
| ENSG00000055044 | 1.597 | 7.581e-37 | 8.974e-36 | NOP58 |
| ENSG00000055332 | -1.349 | 1.595e-24 | 1.133e-23 | EIF2AK2 |
| ENSG00000055483 | 1.334 | 1.812e-51 | 3.384e-50 | USP36 |
| ENSG00000055609 | 1.121 | 9.924e-20 | 5.655e-19 | KMT2C |
| ENSG00000056050 | -1.786 | 1.489e-20 | 8.803e-20 | C4orf27 |
| ENSG00000056558 | 1.58 | 5.733e-06 | 1.417e-05 | TRAF1 |
| ENSG00000056661 | -1.006 | 1.764e-07 | 4.949e-07 | PCGF2 |
| ENSG00000056998 | -1.485 | 1.568e-09 | 5.112e-09 | GYG2 |
| ENSG00000057019 | -1.167 | 2.504e-38 | 3.139e-37 | DCBLD2 |
| ENSG00000057593 | 5.158 | 2.931e-07 | 8.096e-07 | F7 |
| ENSG00000057657 | 2.686 | 3.666e-16 | 1.753e-15 | PRDM1 |
| ENSG00000057704 | -2.338 | 1.568e-18 | 8.456e-18 | TMCC3 |
| ENSG00000057935 | -1.009 | 2.627e-18 | 1.401e-17 | MTA3 |
| ENSG00000058056 | -2.846 | 7.156e-78 | 2.813e-76 | USP13 |
| ENSG00000058063 | 1.174 | 2.536e-22 | 1.626e-21 | ATP11B |
| ENSG00000058085 | 1.64 | 1.159e-71 | 3.869e-70 | LAMC2 |
| ENSG00000058453 | -2.331 | 1.925e-24 | 1.362e-23 | CROCC |
| ENSG00000058668 | -1.146 | 3.184e-40 | 4.245e-39 | ATP2B4 |
| ENSG00000058673 | 1.402 | 2.767e-89 | 1.425e-87 | ZC3H11A |
| ENSG00000058729 | -1.099 | 1.171e-20 | 6.954e-20 | RIOK2 |
| ENSG00000058866 | 1.931 | 2.937e-52 | 5.616e-51 | DGKG |
| ENSG00000059377 | 2.603 | 0.00646 | 0.01152 | TBXAS1 |
| ENSG00000059378 | -1.387 | 1.712e-31 | 1.636e-30 | PARP12 |
| ENSG00000059588 | -1.333 | 1.053e-34 | 1.154e-33 | TARBP1 |
| ENSG00000059728 | 2.17 | 2.329e-56 | 4.988e-55 | MXD1 |
| ENSG00000059758 | 1.255 | 5.638e-36 | 6.456e-35 | CDK17 |
| ENSG00000059769 | 1.182 | 2.935e-13 | 1.207e-12 | DNAJC25 |
| ENSG00000060138 | 1.15 | 3.247e-10 | 1.108e-09 | YBX3 |
| ENSG00000060140 | 1.301 | 2.6e-14 | 1.132e-13 | STYK1 |
| ENSG00000060491 | -1.71 | 2.257e-20 | 1.325e-19 | OGFR |
| ENSG00000060642 | -2.114 | 3.802e-08 | 1.121e-07 | PIGV |
| ENSG00000060656 | -1.803 | 5.764e-41 | 7.912e-40 | PTPRU |
| ENSG00000060688 | -1.08 | 1.228e-15 | 5.714e-15 | SNRNP40 |
| ENSG00000060718 | 3.758 | 0.001212 | 0.002383 | COL11A1 |
| ENSG00000060749 | -1.315 | 8.998e-17 | 4.432e-16 | QSER1 |
| ENSG00000061273 | -1.187 | 4.391e-40 | 5.84e-39 | HDAC7 |
| ENSG00000061938 | -1.556 | 3.267e-48 | 5.568e-47 | TNK2 |
| ENSG00000062194 | 1.417 | 4.304e-56 | 9.122e-55 | GPBP1 |
| ENSG00000062282 | -1.779 | 2.378e-22 | 1.526e-21 | DGAT2 |
| ENSG00000062524 | 3.062 | 0.01106 | 0.01904 | LTK |
| ENSG00000062716 | 1.018 | 4.595e-40 | 6.102e-39 | VMP1 |
| ENSG00000062822 | -1.61 | 3.424e-34 | 3.673e-33 | POLD1 |
| ENSG00000063127 | -1.797 | 0.0143 | 0.02419 | SLC6A16 |
| ENSG00000063169 | 1.065 | 3.672e-10 | 1.247e-09 | GLTSCR1 |
| ENSG00000063438 | 2.919 | 1.477e-45 | 2.339e-44 | AHRR |
| ENSG00000063761 | -1.202 | 8.813e-15 | 3.934e-14 | ADCK1 |
| ENSG00000064042 | -1.033 | 1.208e-42 | 1.745e-41 | LIMCH1 |
| ENSG00000064195 | -3.337 | 1.037e-20 | 6.179e-20 | DLX3 |
| ENSG00000064300 | 3.211 | 0.01124 | 0.01933 | NGFR |
| ENSG00000064309 | -1.634 | 2.042e-10 | 7.052e-10 | CDON |
| ENSG00000064607 | -1.198 | 1.253e-27 | 1.016e-26 | SUGP2 |
| ENSG00000064703 | -2.068 | 2.555e-43 | 3.792e-42 | DDX20 |
| ENSG00000064835 | 3.384 | 0.01041 | 0.01799 | POU1F1 |
| ENSG00000064933 | -2.742 | 1.324e-31 | 1.271e-30 | PMS1 |
| ENSG00000065054 | -1.56 | 5.083e-25 | 3.687e-24 | SLC9A3R2 |
| ENSG00000065057 | -1.708 | 4.825e-18 | 2.539e-17 | NTHL1 |
| ENSG00000065308 | -2.43 | 2.259e-117 | 2.06e-115 | TRAM2 |
| ENSG00000065320 | -1.206 | 1.437e-25 | 1.074e-24 | NTN1 |
| ENSG00000065328 | -3.453 | 3.352e-125 | 3.702e-123 | MCM10 |
| ENSG00000065357 | -1.021 | 1.918e-14 | 8.386e-14 | DGKA |
| ENSG00000065361 | -2.535 | 9.121e-119 | 8.799e-117 | ERBB3 |
| ENSG00000065413 | -1.209 | 0.01479 | 0.02495 | ANKRD44 |
| ENSG00000065457 | -1.541 | 1.424e-33 | 1.477e-32 | ADAT1 |
| ENSG00000065615 | 1.455 | 7.064e-37 | 8.38e-36 | CYB5R4 |
| ENSG00000065621 | -1.253 | 2.573e-07 | 7.134e-07 | GSTO2 |
| ENSG00000065675 | -3.036 | 8.355e-14 | 3.543e-13 | PRKCQ |
| ENSG00000065833 | 1.678 | 1.201e-112 | 9.98e-111 | ME1 |
| ENSG00000065911 | 1.369 | 3.884e-61 | 9.834e-60 | MTHFD2 |
| ENSG00000065970 | -3.13 | 1.663e-59 | 3.998e-58 | FOXJ2 |
| ENSG00000065989 | -3.296 | 2.582e-28 | 2.152e-27 | PDE4A |
| ENSG00000066056 | 5.547 | 7.846e-08 | 2.261e-07 | TIE1 |
| ENSG00000066248 | -1.125 | 1.225e-06 | 3.209e-06 | NGEF |
| ENSG00000066279 | -2.533 | 1.666e-46 | 2.716e-45 | ASPM |
| ENSG00000066336 | 3.047 | 0.00227 | 0.004317 | SPI1 |
| ENSG00000066422 | 1.526 | 3.364e-52 | 6.424e-51 | ZBTB11 |
| ENSG00000066583 | -1.871 | 1.037e-29 | 9.19e-29 | ISOC1 |
| ENSG00000066629 | -1.837 | 2.326e-09 | 7.476e-09 | EML1 |
| ENSG00000066735 | -2.107 | 0.01522 | 0.02562 | KIF26A |
| ENSG00000066923 | -1.661 | 1.069e-11 | 4.007e-11 | STAG3 |
| ENSG00000067082 | 2.423 | 1.059e-174 | 2.436e-172 | KLF6 |
| ENSG00000067113 | -1.361 | 6.659e-10 | 2.223e-09 | PPAP2A |
| ENSG00000067141 | -1.017 | 2.102e-06 | 5.377e-06 | NEO1 |
| ENSG00000067191 | -1.221 | 7.819e-10 | 2.601e-09 | CACNB1 |
| ENSG00000067221 | -2.741 | 3.551e-09 | 1.128e-08 | STOML1 |
| ENSG00000067334 | 1.822 | 9.17e-34 | 9.602e-33 | DNTTIP2 |
| ENSG00000067369 | -1.001 | 3.88e-22 | 2.469e-21 | TP53BP1 |
| ENSG00000067715 | 1.637 | 5.582e-09 | 1.748e-08 | SYT1 |
| ENSG00000067798 | 2.611 | 1.303e-66 | 3.852e-65 | NAV3 |
| ENSG00000067992 | -1.076 | 2.46e-08 | 7.349e-08 | PDK3 |
| ENSG00000068001 | -4.405 | 3.941e-77 | 1.517e-75 | HYAL2 |
| ENSG00000068078 | -2.737 | 6.475e-27 | 5.095e-26 | FGFR3 |
| ENSG00000068097 | -2.21 | 3.456e-33 | 3.539e-32 | HEATR6 |
| ENSG00000068120 | -1.099 | 4.999e-16 | 2.373e-15 | COASY |
| ENSG00000068137 | -1.669 | 1.829e-12 | 7.175e-12 | PLEKHH3 |
| ENSG00000068308 | 1.124 | 1.051e-12 | 4.191e-12 | OTUD5 |
| ENSG00000068366 | 1.351 | 9.186e-54 | 1.827e-52 | ACSL4 |
| ENSG00000068654 | -1.533 | 5.378e-71 | 1.763e-69 | POLR1A |
| ENSG00000068724 | -1.978 | 4.882e-48 | 8.303e-47 | TTC7A |
| ENSG00000068878 | 1.251 | 4.54e-44 | 6.884e-43 | PSME4 |
| ENSG00000068885 | -1.086 | 9.34e-27 | 7.309e-26 | IFT80 |
| ENSG00000068976 | 2.451 | 1.658e-07 | 4.665e-07 | PYGM |
| ENSG00000069011 | -2.048 | 1.353e-49 | 2.402e-48 | PITX1 |
| ENSG00000069020 | 1.88 | 9.159e-55 | 1.877e-53 | MAST4 |
| ENSG00000069122 | -1.405 | 1.144e-20 | 6.8e-20 | GPR116 |
| ENSG00000069248 | -1.049 | 6.279e-18 | 3.284e-17 | NUP133 |
| ENSG00000069275 | -1.642 | 6.612e-129 | 7.654e-127 | NUCKS1 |
| ENSG00000069399 | -1.556 | 1.185e-11 | 4.433e-11 | BCL3 |
| ENSG00000069431 | 4.021 | 8.162e-13 | 3.272e-12 | ABCC9 |
| ENSG00000069493 | -3.026 | 2.941e-31 | 2.772e-30 | CLEC2D |
| ENSG00000069667 | 1.687 | 1.471e-09 | 4.804e-09 | RORA |
| ENSG00000069696 | 2.032 | 0.001131 | 0.00223 | DRD4 |
| ENSG00000069702 | -4.059 | 4.359e-47 | 7.274e-46 | TGFBR3 |
| ENSG00000069712 | -1.103 | 0.0006576 | 0.001332 | KIAA1107 |
| ENSG00000069812 | -3.804 | 4.145e-15 | 1.879e-14 | HES2 |
| ENSG00000069956 | 1.105 | 1.879e-40 | 2.527e-39 | MAPK6 |
| ENSG00000069974 | -1.433 | 1.037e-09 | 3.425e-09 | RAB27A |
| ENSG00000069998 | -1.881 | 2.591e-18 | 1.382e-17 | CECR5 |
| ENSG00000070010 | 1.67 | 2.651e-59 | 6.303e-58 | UFD1L |
| ENSG00000070018 | -1.213 | 1.801e-16 | 8.724e-16 | LRP6 |
| ENSG00000070159 | -1.689 | 7.219e-49 | 1.262e-47 | PTPN3 |
| ENSG00000070190 | 2.194 | 1.747e-37 | 2.129e-36 | DAPP1 |
| ENSG00000070388 | 1.645 | 0.01065 | 0.01838 | FGF22 |
| ENSG00000070413 | -1.969 | 5.029e-32 | 4.92e-31 | DGCR2 |
| ENSG00000070476 | -1.339 | 5.514e-24 | 3.822e-23 | ZXDC |
| ENSG00000070610 | -1.088 | 3.034e-21 | 1.858e-20 | GBA2 |
| ENSG00000070614 | -2.079 | 1.244e-148 | 1.951e-146 | NDST1 |
| ENSG00000070669 | 1.55 | 5.448e-60 | 1.335e-58 | ASNS |
| ENSG00000070718 | -2.241 | 2.657e-61 | 6.757e-60 | AP3M2 |
| ENSG00000070729 | 1.666 | 9.558e-16 | 4.465e-15 | CNGB1 |
| ENSG00000070731 | -1.467 | 3.994e-07 | 1.091e-06 | ST6GALNAC2 |
| ENSG00000070886 | 3.921 | 0.002322 | 0.004405 | EPHA8 |
| ENSG00000070915 | -3.186 | 0.003477 | 0.00643 | SLC12A3 |
| ENSG00000071051 | -1.378 | 7.643e-20 | 4.376e-19 | NCK2 |
| ENSG00000071073 | -1.558 | 3.913e-53 | 7.646e-52 | MGAT4A |
| ENSG00000071246 | -2.21 | 6.776e-10 | 2.261e-09 | VASH1 |
| ENSG00000071537 | 1.472 | 6.801e-62 | 1.761e-60 | SEL1L |
| ENSG00000071539 | -1.37 | 2.899e-30 | 2.614e-29 | TRIP13 |
| ENSG00000071564 | -1.279 | 9.874e-32 | 9.537e-31 | TCF3 |
| ENSG00000071575 | -2.621 | 5.768e-57 | 1.259e-55 | TRIB2 |
| ENSG00000071655 | -1.403 | 4.071e-23 | 2.699e-22 | MBD3 |
| ENSG00000071909 | -2.229 | 3.202e-05 | 7.403e-05 | MYO3B |
| ENSG00000072134 | -1.54 | 6.35e-50 | 1.139e-48 | EPN2 |
| ENSG00000072135 | -1.425 | 1.207e-10 | 4.237e-10 | PTPN18 |
| ENSG00000072163 | 1.849 | 0.0006892 | 0.001391 | LIMS2 |
| ENSG00000072182 | 2.251 | 0.01179 | 0.02021 | ASIC4 |
| ENSG00000072195 | -1.542 | 9.625e-25 | 6.89e-24 | SPEG |
| ENSG00000072210 | -1.504 | 6.902e-23 | 4.526e-22 | ALDH3A2 |
| ENSG00000072310 | -1.281 | 2.251e-25 | 1.663e-24 | SREBF1 |
| ENSG00000072315 | 2.838 | 0.01491 | 0.02514 | TRPC5 |
| ENSG00000072401 | 1.687 | 3.946e-23 | 2.619e-22 | UBE2D1 |
| ENSG00000072415 | -1.664 | 1.685e-48 | 2.91e-47 | MPP5 |
| ENSG00000072422 | -3.583 | 2.189e-43 | 3.258e-42 | RHOBTB1 |
| ENSG00000072571 | -2.149 | 1.688e-50 | 3.076e-49 | HMMR |
| ENSG00000072609 | -1.049 | 1.041e-17 | 5.378e-17 | CHFR |
| ENSG00000072682 | 1.261 | 2.948e-16 | 1.416e-15 | P4HA2 |
| ENSG00000072694 | 2.908 | 0.008971 | 0.01565 | FCGR2B |
| ENSG00000072736 | -1.4 | 1.672e-28 | 1.408e-27 | NFATC3 |
| ENSG00000072818 | 3.256 | 8.105e-17 | 4.004e-16 | ACAP1 |
| ENSG00000072832 | -2.004 | 3.226e-05 | 7.457e-05 | CRMP1 |
| ENSG00000072840 | -2.049 | 1.034e-14 | 4.592e-14 | EVC |
| ENSG00000072858 | -1.405 | 2.307e-05 | 5.402e-05 | SIDT1 |
| ENSG00000072864 | -1.691 | 5.687e-42 | 8.069e-41 | NDE1 |
| ENSG00000072958 | -2.281 | 4.022e-34 | 4.295e-33 | AP1M1 |
| ENSG00000073050 | -1.632 | 1.578e-24 | 1.121e-23 | XRCC1 |
| ENSG00000073060 | -1.038 | 3.227e-19 | 1.796e-18 | SCARB1 |
| ENSG00000073067 | 2.292 | 7.214e-05 | 0.0001615 | CYP2W1 |
| ENSG00000073111 | -2.122 | 6.935e-74 | 2.466e-72 | MCM2 |
| ENSG00000073169 | -1.011 | 1.094e-06 | 2.881e-06 | SELO |
| ENSG00000073282 | 2.88 | 2.352e-37 | 2.844e-36 | TP63 |
| ENSG00000073464 | -1.246 | 7.645e-15 | 3.424e-14 | CLCN4 |
| ENSG00000073536 | -1.22 | 1.277e-10 | 4.472e-10 | NLE1 |
| ENSG00000073584 | -1.104 | 1.253e-20 | 7.433e-20 | SMARCE1 |
| ENSG00000073605 | -1.111 | 0.000178 | 0.0003829 | GSDMB |
| ENSG00000073614 | 1.38 | 1.019e-54 | 2.084e-53 | KDM5A |
| ENSG00000073711 | -1.822 | 4.299e-37 | 5.147e-36 | PPP2R3A |
| ENSG00000073712 | 1.481 | 1.257e-35 | 1.425e-34 | FERMT2 |
| ENSG00000073792 | 1.466 | 1.209e-16 | 5.91e-16 | IGF2BP2 |
| ENSG00000073849 | -2.862 | 1.525e-79 | 6.139e-78 | ST6GAL1 |
| ENSG00000073910 | -1.87 | 3.491e-16 | 1.672e-15 | FRY |
| ENSG00000073921 | 1.586 | 3.347e-42 | 4.785e-41 | PICALM |
| ENSG00000073969 | -1.052 | 3.439e-25 | 2.512e-24 | NSF |
| ENSG00000074071 | -1.629 | 1.031e-33 | 1.078e-32 | MRPS34 |
| ENSG00000074181 | -1.195 | 9.065e-50 | 1.621e-48 | NOTCH3 |
| ENSG00000074219 | -1.191 | 2.623e-20 | 1.534e-19 | TEAD2 |
| ENSG00000074266 | -1.453 | 3.57e-22 | 2.276e-21 | EED |
| ENSG00000074276 | 1.82 | 6.188e-06 | 1.524e-05 | CDHR2 |
| ENSG00000074410 | -1.831 | 0.002507 | 0.004739 | CA12 |
| ENSG00000074527 | -1.087 | 2.98e-22 | 1.905e-21 | NTN4 |
| ENSG00000074582 | -1.473 | 2.219e-18 | 1.187e-17 | BCS1L |
| ENSG00000074590 | -1.146 | 3.649e-16 | 1.746e-15 | NUAK1 |
| ENSG00000074603 | -1.466 | 2.875e-35 | 3.222e-34 | DPP8 |
| ENSG00000074621 | -2.084 | 1.583e-26 | 1.229e-25 | SLC24A1 |
| ENSG00000074855 | -1.752 | 8.654e-20 | 4.943e-19 | ANO8 |
| ENSG00000074964 | -1.611 | 1.347e-17 | 6.906e-17 | ARHGEF10L |
| ENSG00000075043 | 1.622 | 0.007691 | 0.01356 | KCNQ2 |
| ENSG00000075073 | 1.21 | 0.001248 | 0.002451 | TACR2 |
| ENSG00000075131 | -1.608 | 6.063e-28 | 4.989e-27 | TIPIN |
| ENSG00000075188 | -1.37 | 7.108e-22 | 4.47e-21 | NUP37 |
| ENSG00000075213 | -2.6 | 2.095e-07 | 5.843e-07 | SEMA3A |
| ENSG00000075218 | -3.739 | 8.915e-86 | 4.263e-84 | GTSE1 |
| ENSG00000075234 | -1.02 | 4.468e-09 | 1.408e-08 | TTC38 |
| ENSG00000075240 | -1.908 | 8.299e-29 | 7.096e-28 | GRAMD4 |
| ENSG00000075303 | -1.555 | 9.677e-31 | 8.925e-30 | SLC25A40 |
| ENSG00000075336 | -1.088 | 3.832e-10 | 1.299e-09 | TIMM21 |
| ENSG00000075413 | 1.222 | 1.397e-51 | 2.619e-50 | MARK3 |
| ENSG00000075461 | -3.078 | 1.71e-90 | 9.11e-89 | CACNG4 |
| ENSG00000075618 | -1.319 | 6.641e-35 | 7.32e-34 | FSCN1 |
| ENSG00000075651 | -1.748 | 1.777e-18 | 9.553e-18 | PLD1 |
| ENSG00000075702 | -1.327 | 6.831e-17 | 3.386e-16 | WDR62 |
| ENSG00000075785 | 2.08 | 7.414e-17 | 3.668e-16 | RAB7A |
| ENSG00000075826 | -1.612 | 0.0001118 | 0.000246 | SEC31B |
| ENSG00000075856 | 1.175 | 1.629e-30 | 1.485e-29 | SART3 |
| ENSG00000076003 | -2.328 | 8.93e-101 | 5.925e-99 | MCM6 |
| ENSG00000076053 | 1.099 | 3.035e-17 | 1.531e-16 | RBM7 |
| ENSG00000076248 | -2.413 | 1.301e-30 | 1.191e-29 | UNG |
| ENSG00000076351 | -1.884 | 5.397e-11 | 1.938e-10 | SLC46A1 |
| ENSG00000076382 | -1.498 | 4.237e-45 | 6.61e-44 | SPAG5 |
| ENSG00000076555 | -2.629 | 3.356e-40 | 4.471e-39 | ACACB |
| ENSG00000076604 | -1.773 | 8.074e-39 | 1.032e-37 | TRAF4 |
| ENSG00000076650 | -2.417 | 2.714e-26 | 2.089e-25 | GPATCH1 |
| ENSG00000076706 | -1.011 | 1.431e-23 | 9.703e-23 | MCAM |
| ENSG00000076716 | -1.076 | 0.002838 | 0.005317 | GPC4 |
| ENSG00000076770 | -1.877 | 0.02384 | 0.03874 | MBNL3 |
| ENSG00000076826 | -1.436 | 5.527e-14 | 2.365e-13 | CAMSAP3 |
| ENSG00000077009 | 4.218 | 0.0002045 | 0.0004374 | NMRK2 |
| ENSG00000077152 | -2.266 | 2.029e-87 | 1.011e-85 | UBE2T |
| ENSG00000077420 | 3.541 | 0.007854 | 0.01384 | APBB1IP |
| ENSG00000077454 | -2.58 | 9.894e-84 | 4.525e-82 | LRCH4 |
| ENSG00000077463 | 1.13 | 1.145e-15 | 5.336e-15 | SIRT6 |
| ENSG00000077514 | -2.416 | 6.283e-93 | 3.612e-91 | POLD3 |
| ENSG00000077616 | 3.393 | 6.264e-51 | 1.156e-49 | NAALAD2 |
| ENSG00000077684 | -2.349 | 3.857e-47 | 6.449e-46 | PHF17 |
| ENSG00000077809 | -1.875 | 8.273e-118 | 7.715e-116 | GTF2I |
| ENSG00000078142 | 1.041 | 6.897e-28 | 5.664e-27 | PIK3C3 |
| ENSG00000078177 | -1.243 | 2.956e-18 | 1.572e-17 | N4BP2 |
| ENSG00000078269 | 1.017 | 1.899e-36 | 2.202e-35 | SYNJ2 |
| ENSG00000078399 | -3.07 | 5.004e-19 | 2.755e-18 | HOXA9 |
| ENSG00000078401 | 1.175 | 5.674e-15 | 2.551e-14 | EDN1 |
| ENSG00000078795 | 2.026 | 0.0133 | 0.02261 | PKD2L2 |
| ENSG00000078814 | 1.066 | 0.02462 | 0.03992 | MYH7B |
| ENSG00000078900 | -2.83 | 5.526e-15 | 2.487e-14 | TP73 |
| ENSG00000078967 | -1.501 | 1.034e-13 | 4.368e-13 | UBE2D4 |
| ENSG00000079112 | 3.232 | 0.002556 | 0.004826 | CDH17 |
| ENSG00000079215 | -1.534 | 1.196e-38 | 1.521e-37 | SLC1A3 |
| ENSG00000079263 | 2.691 | 4.411e-19 | 2.436e-18 | SP140 |
| ENSG00000079308 | -1.15 | 6.33e-07 | 1.698e-06 | TNS1 |
| ENSG00000079435 | 1.317 | 2.67e-26 | 2.056e-25 | LIPE |
| ENSG00000079616 | -2.703 | 3.396e-56 | 7.244e-55 | KIF22 |
| ENSG00000079974 | -1.291 | 2.656e-34 | 2.862e-33 | RABL2B |
| ENSG00000080007 | 1.212 | 1.041e-13 | 4.399e-13 | DDX43 |
| ENSG00000080200 | -2.345 | 7.149e-48 | 1.211e-46 | CRYBG3 |
| ENSG00000080298 | -1.038 | 2.696e-06 | 6.834e-06 | RFX3 |
| ENSG00000080371 | 1.051 | 8.005e-27 | 6.288e-26 | RAB21 |
| ENSG00000080493 | 2.919 | 0.003336 | 0.006185 | SLC4A4 |
| ENSG00000080503 | -1.687 | 1.657e-28 | 1.396e-27 | SMARCA2 |
| ENSG00000080546 | -2.007 | 5.085e-13 | 2.062e-12 | SESN1 |
| ENSG00000080802 | 1.777 | 4.54e-13 | 1.847e-12 | CNOT4 |
| ENSG00000080819 | -2.217 | 2.353e-59 | 5.626e-58 | CPOX |
| ENSG00000080824 | 2.411 | 5.279e-218 | 2.332e-215 | HSP90AA1 |
| ENSG00000080839 | -2.561 | 3.919e-60 | 9.647e-59 | RBL1 |
| ENSG00000080845 | 1.144 | 1.209e-12 | 4.793e-12 | DLGAP4 |
| ENSG00000080986 | -2.713 | 2.54e-90 | 1.345e-88 | NDC80 |
| ENSG00000081041 | 2.003 | 2.289e-12 | 8.926e-12 | CXCL2 |
| ENSG00000081052 | -1.439 | 0.0004489 | 0.000925 | COL4A4 |
| ENSG00000081138 | -2.551 | 0.002112 | 0.004034 | CDH7 |
| ENSG00000081177 | -1.101 | 3.072e-13 | 1.262e-12 | EXD2 |
| ENSG00000081181 | 1.989 | 4.979e-13 | 2.021e-12 | ARG2 |
| ENSG00000081189 | -1.943 | 0.0004754 | 0.0009773 | MEF2C |
| ENSG00000081248 | 4.357 | 3.939e-07 | 1.076e-06 | CACNA1S |
| ENSG00000081692 | -2.249 | 7.344e-30 | 6.537e-29 | JMJD4 |
| ENSG00000081721 | -1.216 | 7.442e-12 | 2.811e-11 | DUSP12 |
| ENSG00000081803 | -1.708 | 0.003361 | 0.006229 | CADPS2 |
| ENSG00000081923 | -1.796 | 6.917e-72 | 2.327e-70 | ATP8B1 |
| ENSG00000081985 | 3.641 | 3.186e-10 | 1.088e-09 | IL12RB2 |
| ENSG00000082068 | -1.534 | 3.754e-34 | 4.018e-33 | WDR70 |
| ENSG00000082146 | -2.637 | 9.522e-56 | 2e-54 | STRADB |
| ENSG00000082153 | 1.077 | 1.442e-16 | 7.026e-16 | BZW1 |
| ENSG00000082175 | 4.074 | 9.03e-10 | 2.993e-09 | PGR |
| ENSG00000082196 | -3.436 | 2.561e-23 | 1.717e-22 | C1QTNF3 |
| ENSG00000082438 | -1.544 | 2.479e-07 | 6.885e-07 | COBLL1 |
| ENSG00000082458 | -2.774 | 2.378e-144 | 3.299e-142 | DLG3 |
| ENSG00000082482 | -1.953 | 7.97e-05 | 0.0001777 | KCNK2 |
| ENSG00000082497 | -1.205 | 3.891e-23 | 2.583e-22 | SERTAD4 |
| ENSG00000082512 | -1.445 | 1.945e-06 | 4.996e-06 | TRAF5 |
| ENSG00000082516 | -3.554 | 1.785e-132 | 2.171e-130 | GEMIN5 |
| ENSG00000082701 | 1.088 | 7.672e-29 | 6.567e-28 | GSK3B |
| ENSG00000082781 | -1.088 | 1.67e-16 | 8.101e-16 | ITGB5 |
| ENSG00000082898 | -1.001 | 3.673e-24 | 2.562e-23 | XPO1 |
| ENSG00000082996 | -1.163 | 4.429e-26 | 3.381e-25 | RNF13 |
| ENSG00000083093 | -1.651 | 4.098e-33 | 4.19e-32 | PALB2 |
| ENSG00000083223 | 2.111 | 3.503e-58 | 7.968e-57 | ZCCHC6 |
| ENSG00000083290 | -1.431 | 1.323e-17 | 6.789e-17 | ULK2 |
| ENSG00000083307 | -2.035 | 2.889e-78 | 1.144e-76 | GRHL2 |
| ENSG00000083312 | 1.484 | 1.647e-76 | 6.255e-75 | TNPO1 |
| ENSG00000083454 | -2.463 | 7.279e-21 | 4.37e-20 | P2RX5 |
| ENSG00000083799 | 1.205 | 4.928e-37 | 5.888e-36 | CYLD |
| ENSG00000083896 | 1.778 | 6.933e-35 | 7.631e-34 | YTHDC1 |
| ENSG00000083937 | 1.287 | 4.831e-27 | 3.823e-26 | CHMP2B |
| ENSG00000084093 | -1.14 | 2.898e-24 | 2.033e-23 | REST |
| ENSG00000084444 | -1.224 | 1.663e-09 | 5.408e-09 | KIAA1467 |
| ENSG00000084652 | -1.08 | 1.231e-33 | 1.28e-32 | TXLNA |
| ENSG00000084710 | -1.625 | 2.274e-11 | 8.369e-11 | EFR3B |
| ENSG00000084734 | 4.033 | 4.26e-06 | 1.065e-05 | GCKR |
| ENSG00000084764 | 1.071 | 1.019e-12 | 4.067e-12 | MAPRE3 |
| ENSG00000084774 | -2.544 | 4.502e-45 | 7.003e-44 | CAD |
| ENSG00000085185 | -1.553 | 1.428e-09 | 4.671e-09 | BCORL1 |
| ENSG00000085276 | -2.224 | 3.008e-42 | 4.309e-41 | MECOM |
| ENSG00000085511 | -1.8 | 2.128e-43 | 3.172e-42 | MAP3K4 |
| ENSG00000085514 | 1.239 | 0.02019 | 0.03328 | PILRA |
| ENSG00000085552 | -3.677 | 6.28e-57 | 1.367e-55 | IGSF9 |
| ENSG00000085563 | 4.174 | 0.001652 | 0.003199 | ABCB1 |
| ENSG00000085741 | -1.736 | 0.02384 | 0.03874 | WNT11 |
| ENSG00000085760 | -1.345 | 6.271e-30 | 5.588e-29 | MTIF2 |
| ENSG00000085788 | -1.399 | 6.602e-27 | 5.193e-26 | DDHD2 |
| ENSG00000085840 | -2.083 | 7.426e-91 | 3.983e-89 | ORC1 |
| ENSG00000085982 | -1.045 | 1.63e-39 | 2.129e-38 | USP40 |
| ENSG00000085999 | -1.099 | 3.515e-27 | 2.799e-26 | RAD54L |
| ENSG00000086015 | -1.613 | 4.981e-61 | 1.255e-59 | MAST2 |
| ENSG00000086061 | 2.389 | 2.917e-156 | 5.266e-154 | DNAJA1 |
| ENSG00000086189 | -1.317 | 2.923e-44 | 4.452e-43 | DIMT1 |
| ENSG00000086289 | -2.494 | 1.932e-09 | 6.25e-09 | EPDR1 |
| ENSG00000086475 | -1.249 | 5.162e-40 | 6.845e-39 | SEPHS1 |
| ENSG00000086544 | 1.454 | 1.104e-16 | 5.404e-16 | ITPKC |
| ENSG00000086570 | 1.86 | 3.222e-10 | 1.1e-09 | FAT2 |
| ENSG00000086619 | 2.375 | 6.349e-58 | 1.427e-56 | ERO1LB |
| ENSG00000087074 | 5.104 | 0 | 0 | PPP1R15A |
| ENSG00000087085 | 1.32 | 0.02612 | 0.04219 | ACHE |
| ENSG00000087111 | -1.282 | 8.074e-25 | 5.795e-24 | PIGS |
| ENSG00000087237 | 3.387 | 0.004289 | 0.007838 | CETP |
| ENSG00000087258 | -2.156 | 5.632e-15 | 2.533e-14 | GNAO1 |
| ENSG00000087299 | -1.466 | 1.389e-17 | 7.117e-17 | L2HGDH |
| ENSG00000087303 | 1.409 | 0.001674 | 0.003237 | NID2 |
| ENSG00000087448 | -2.703 | 7.042e-102 | 4.805e-100 | KLHL42 |
| ENSG00000087495 | 1.559 | 0.002303 | 0.004373 | PHACTR3 |
| ENSG00000087510 | -2.702 | 9.826e-124 | 1.051e-121 | TFAP2C |
| ENSG00000087586 | -3.302 | 3.864e-242 | 1.966e-239 | AURKA |
| ENSG00000087903 | -1.468 | 4.102e-11 | 1.483e-10 | RFX2 |
| ENSG00000088002 | -1.074 | 3.558e-07 | 9.763e-07 | SULT2B1 |
| ENSG00000088035 | -1.416 | 1.873e-18 | 1.005e-17 | ALG6 |
| ENSG00000088038 | 1.21 | 1.231e-09 | 4.049e-09 | CNOT3 |
| ENSG00000088053 | 3.076 | 1.461e-16 | 7.115e-16 | GP6 |
| ENSG00000088280 | -1.396 | 2.654e-07 | 7.35e-07 | ASAP3 |
| ENSG00000088305 | -1.678 | 9.17e-17 | 4.509e-16 | DNMT3B |
| ENSG00000088325 | -1.993 | 1.946e-40 | 2.611e-39 | TPX2 |
| ENSG00000088367 | -1.386 | 4.499e-26 | 3.433e-25 | EPB41L1 |
| ENSG00000088386 | -2.278 | 0.0003189 | 0.0006688 | SLC15A1 |
| ENSG00000088543 | -1.989 | 1.456e-05 | 3.468e-05 | C3orf18 |
| ENSG00000088682 | -2.152 | 2.043e-63 | 5.514e-62 | COQ9 |
| ENSG00000088726 | 1.02 | 7.34e-12 | 2.775e-11 | TMEM40 |
| ENSG00000088756 | -4.501 | 3.578e-09 | 1.136e-08 | ARHGAP28 |
| ENSG00000088808 | -1.408 | 1.917e-10 | 6.632e-10 | PPP1R13B |
| ENSG00000088826 | 1.136 | 8.144e-13 | 3.266e-12 | SMOX |
| ENSG00000088836 | -1.861 | 1.321e-55 | 2.755e-54 | SLC4A11 |
| ENSG00000088888 | -1.754 | 1.404e-56 | 3.026e-55 | MAVS |
| ENSG00000088986 | -1.648 | 2.13e-24 | 1.502e-23 | DYNLL1 |
| ENSG00000089050 | -1.292 | 9.71e-31 | 8.95e-30 | RBBP9 |
| ENSG00000089053 | -1.665 | 1.677e-30 | 1.527e-29 | ANAPC5 |
| ENSG00000089091 | -1.254 | 3.442e-08 | 1.017e-07 | DZANK1 |
| ENSG00000089094 | -1.825 | 1.428e-28 | 1.206e-27 | KDM2B |
| ENSG00000089116 | -1.264 | 0.009546 | 0.01659 | LHX5 |
| ENSG00000089127 | -1.339 | 2.674e-06 | 6.781e-06 | OAS1 |
| ENSG00000089154 | -1.428 | 4.017e-43 | 5.925e-42 | GCN1L1 |
| ENSG00000089335 | -2.35 | 2.356e-52 | 4.52e-51 | ZNF302 |
| ENSG00000089505 | -1.707 | 7.332e-05 | 0.0001641 | CMTM1 |
| ENSG00000089685 | -1.48 | 2.242e-15 | 1.029e-14 | BIRC5 |
| ENSG00000089723 | -1.576 | 7.133e-06 | 1.748e-05 | OTUB2 |
| ENSG00000089775 | -2.529 | 3.243e-39 | 4.19e-38 | ZBTB25 |
| ENSG00000089820 | 2.125 | 1.452e-09 | 4.742e-09 | ARHGAP4 |
| ENSG00000089876 | -1.957 | 1.752e-40 | 2.362e-39 | DHX32 |
| ENSG00000089916 | 1.525 | 1.002e-68 | 3.115e-67 | GPATCH2L |
| ENSG00000090060 | 1.315 | 1.074e-17 | 5.542e-17 | PAPOLA |
| ENSG00000090061 | 1.578 | 2.86e-13 | 1.178e-12 | CCNK |
| ENSG00000090097 | -1.372 | 1.139e-14 | 5.041e-14 | PCBP4 |
| ENSG00000090376 | 3.837 | 1.724e-07 | 4.842e-07 | IRAK3 |
| ENSG00000090470 | -1.883 | 4.804e-43 | 7.036e-42 | PDCD7 |
| ENSG00000090520 | 2.227 | 5.99e-170 | 1.289e-167 | DNAJB11 |
| ENSG00000090554 | -1.074 | 0.001492 | 0.002904 | FLT3LG |
| ENSG00000090565 | -1.515 | 3.493e-26 | 2.678e-25 | RAB11FIP3 |
| ENSG00000090661 | -1.887 | 1.972e-20 | 1.161e-19 | CERS4 |
| ENSG00000090776 | -1.218 | 5.403e-09 | 1.694e-08 | EFNB1 |
| ENSG00000090857 | -1.148 | 1.296e-43 | 1.942e-42 | PDPR |
| ENSG00000090889 | -2.768 | 2.019e-117 | 1.852e-115 | KIF4A |
| ENSG00000090924 | 1.877 | 5.358e-15 | 2.412e-14 | PLEKHG2 |
| ENSG00000090971 | -1.15 | 1.388e-16 | 6.769e-16 | NAT14 |
| ENSG00000091039 | 1.158 | 2.143e-26 | 1.653e-25 | OSBPL8 |
| ENSG00000091106 | 2.047 | 7.88e-09 | 2.444e-08 | NLRC4 |
| ENSG00000091127 | -2.399 | 6.397e-66 | 1.858e-64 | PUS7 |
| ENSG00000091128 | 2.755 | 0.001367 | 0.002673 | LAMB4 |
| ENSG00000091136 | -1.485 | 9.529e-82 | 4.07e-80 | LAMB1 |
| ENSG00000091138 | 4.672 | 4.18e-06 | 1.045e-05 | SLC26A3 |
| ENSG00000091157 | -1.385 | 7.127e-22 | 4.481e-21 | WDR7 |
| ENSG00000091164 | 1.54 | 1.137e-47 | 1.916e-46 | TXNL1 |
| ENSG00000091542 | -1.393 | 2.132e-19 | 1.193e-18 | ALKBH5 |
| ENSG00000091592 | 2.127 | 3.415e-44 | 5.192e-43 | NLRP1 |
| ENSG00000091879 | 2.355 | 0.0003012 | 0.0006334 | ANGPT2 |
| ENSG00000091947 | -1.292 | 1.64e-09 | 5.339e-09 | TMEM101 |
| ENSG00000091972 | -2.124 | 6.214e-26 | 4.718e-25 | CD200 |
| ENSG00000091986 | -1.723 | 9.86e-40 | 1.296e-38 | CCDC80 |
| ENSG00000092094 | -1.352 | 1.725e-16 | 8.357e-16 | OSGEP |
| ENSG00000092098 | -1.822 | 5.75e-57 | 1.257e-55 | RNF31 |
| ENSG00000092200 | -1.194 | 0.00448 | 0.008155 | RPGRIP1 |
| ENSG00000092203 | 1.167 | 1.475e-36 | 1.719e-35 | TOX4 |
| ENSG00000092208 | -2.073 | 2.35e-20 | 1.378e-19 | GEMIN2 |
| ENSG00000092345 | 5.052 | 1.027e-10 | 3.617e-10 | DAZL |
| ENSG00000092470 | -2.041 | 4.554e-56 | 9.64e-55 | WDR76 |
| ENSG00000092531 | 1.686 | 2.494e-70 | 8.05e-69 | SNAP23 |
| ENSG00000092758 | -2.238 | 1.675e-10 | 5.817e-10 | COL9A3 |
| ENSG00000092847 | -1.938 | 9.795e-74 | 3.461e-72 | AGO1 |
| ENSG00000092853 | -3.735 | 1.169e-99 | 7.461e-98 | CLSPN |
| ENSG00000092929 | -1.334 | 2.989e-36 | 3.458e-35 | UNC13D |
| ENSG00000092964 | -1.237 | 8.855e-27 | 6.936e-26 | DPYSL2 |
| ENSG00000093183 | -2.123 | 3.317e-57 | 7.298e-56 | SEC22C |
| ENSG00000093217 | -2.251 | 5.997e-09 | 1.875e-08 | XYLB |
| ENSG00000094804 | -1.321 | 3.565e-36 | 4.116e-35 | CDC6 |
| ENSG00000094880 | -2.128 | 1.218e-65 | 3.52e-64 | CDC23 |
| ENSG00000094914 | -1.273 | 2.142e-28 | 1.792e-27 | AAAS |
| ENSG00000094916 | -1.575 | 1.811e-89 | 9.352e-88 | CBX5 |
| ENSG00000095002 | -2.119 | 1.63e-58 | 3.743e-57 | MSH2 |
| ENSG00000095015 | -1.814 | 9.786e-55 | 2.003e-53 | MAP3K1 |
| ENSG00000095203 | -1.616 | 2.004e-29 | 1.759e-28 | EPB41L4B |
| ENSG00000095261 | -1.498 | 2.211e-38 | 2.776e-37 | PSMD5 |
| ENSG00000095397 | 1.024 | 1.928e-14 | 8.426e-14 | DFNB31 |
| ENSG00000095585 | 1.247 | 0.008023 | 0.01412 | BLNK |
| ENSG00000095596 | -3.924 | 2.207e-14 | 9.635e-14 | CYP26A1 |
| ENSG00000095637 | 1.35 | 2.714e-14 | 1.18e-13 | SORBS1 |
| ENSG00000095713 | 3.35 | 0.0007541 | 0.001515 | CRTAC1 |
| ENSG00000095752 | 5.389 | 2.066e-148 | 3.212e-146 | IL11 |
| ENSG00000095906 | -1.364 | 2.067e-06 | 5.294e-06 | NUBP2 |
| ENSG00000095932 | 3.251 | 0.02029 | 0.03342 | C19orf77 |
| ENSG00000095951 | 1.464 | 1.4e-15 | 6.485e-15 | HIVEP1 |
| ENSG00000096060 | -2.221 | 2.043e-69 | 6.433e-68 | FKBP5 |
| ENSG00000096070 | -1.382 | 9.572e-17 | 4.702e-16 | BRPF3 |
| ENSG00000096092 | -1.237 | 1.066e-06 | 2.81e-06 | TMEM14A |
| ENSG00000096093 | -1.165 | 4.238e-07 | 1.154e-06 | EFHC1 |
| ENSG00000096654 | -2.513 | 5.349e-24 | 3.713e-23 | ZNF184 |
| ENSG00000096968 | -1.181 | 0.0003812 | 0.0007924 | JAK2 |
| ENSG00000097033 | -1.241 | 3.548e-37 | 4.267e-36 | SH3GLB1 |
| ENSG00000097046 | -3.327 | 1.072e-111 | 8.649e-110 | CDC7 |
| ENSG00000097096 | -1.444 | 9.3e-23 | 6.07e-22 | SYDE2 |
| ENSG00000099203 | -2.048 | 2.116e-13 | 8.79e-13 | TMED1 |
| ENSG00000099250 | -3.391 | 1.459e-156 | 2.662e-154 | NRP1 |
| ENSG00000099260 | -2.283 | 0.005729 | 0.01029 | PALMD |
| ENSG00000099282 | -1.765 | 2.907e-33 | 2.986e-32 | TSPAN15 |
| ENSG00000099284 | -1.886 | 0.0001097 | 0.0002418 | H2AFY2 |
| ENSG00000099330 | -1.101 | 1.08e-05 | 2.604e-05 | OCEL1 |
| ENSG00000099337 | 3.045 | 1.018e-17 | 5.264e-17 | KCNK6 |
| ENSG00000099338 | 2.125 | 2.219e-12 | 8.659e-12 | CATSPERG |
| ENSG00000099377 | -1.544 | 2.709e-12 | 1.051e-11 | HSD3B7 |
| ENSG00000099381 | -1.056 | 1.319e-10 | 4.618e-10 | SETD1A |
| ENSG00000099797 | 1.396 | 1.412e-38 | 1.786e-37 | TECR |
| ENSG00000099812 | -1.204 | 1.377e-17 | 7.06e-17 | MISP |
| ENSG00000099821 | -1.756 | 1.233e-27 | 1.001e-26 | POLRMT |
| ENSG00000099822 | 1.391 | 0.0005024 | 0.001031 | HCN2 |
| ENSG00000099834 | 2.368 | 7.484e-07 | 1.998e-06 | CDHR5 |
| ENSG00000099849 | -1.807 | 1.998e-17 | 1.016e-16 | RASSF7 |
| ENSG00000099860 | 4.11 | 3.136e-198 | 1.032e-195 | GADD45B |
| ENSG00000099864 | 4.361 | 3.28e-05 | 7.576e-05 | PALM |
| ENSG00000099889 | -1.668 | 7.504e-06 | 1.835e-05 | ARVCF |
| ENSG00000099904 | -2.607 | 1.554e-37 | 1.897e-36 | ZDHHC8 |
| ENSG00000099910 | -2.486 | 4.022e-35 | 4.465e-34 | KLHL22 |
| ENSG00000099949 | -1.145 | 4.124e-07 | 1.124e-06 | LZTR1 |
| ENSG00000099954 | 3.029 | 0.02799 | 0.04494 | CECR2 |
| ENSG00000099985 | 4.601 | 2.164e-08 | 6.487e-08 | OSM |
| ENSG00000099991 | -1.972 | 2.583e-61 | 6.579e-60 | CABIN1 |
| ENSG00000100003 | -1.154 | 9.435e-11 | 3.329e-10 | SEC14L2 |
| ENSG00000100014 | -1.569 | 9.42e-61 | 2.356e-59 | SPECC1L |
| ENSG00000100023 | -1.501 | 1.082e-54 | 2.207e-53 | PPIL2 |
| ENSG00000100027 | 2.603 | 4.331e-06 | 1.081e-05 | YPEL1 |
| ENSG00000100034 | -2.654 | 3.538e-107 | 2.627e-105 | PPM1F |
| ENSG00000100036 | -2.867 | 3.802e-14 | 1.64e-13 | SLC35E4 |
| ENSG00000100065 | -1.65 | 1.659e-18 | 8.935e-18 | CARD10 |
| ENSG00000100068 | -1.296 | 6.36e-06 | 1.565e-05 | LRP5L |
| ENSG00000100075 | -1.224 | 5.436e-09 | 1.703e-08 | SLC25A1 |
| ENSG00000100079 | 3.049 | 8.269e-11 | 2.929e-10 | LGALS2 |
| ENSG00000100083 | -1.111 | 4.834e-25 | 3.51e-24 | GGA1 |
| ENSG00000100092 | -1.988 | 5.995e-38 | 7.443e-37 | SH3BP1 |
| ENSG00000100099 | -1.281 | 3.794e-26 | 2.907e-25 | HPS4 |
| ENSG00000100105 | -4.273 | 6.741e-115 | 5.803e-113 | PATZ1 |
| ENSG00000100106 | -1.114 | 2.245e-25 | 1.66e-24 | TRIOBP |
| ENSG00000100109 | 1.661 | 3.116e-64 | 8.63e-63 | TFIP11 |
| ENSG00000100124 | -2.407 | 4.407e-47 | 7.345e-46 | ANKRD54 |
| ENSG00000100147 | -2.017 | 6.731e-13 | 2.71e-12 | CCDC134 |
| ENSG00000100162 | -1.367 | 2.639e-06 | 6.697e-06 | CENPM |
| ENSG00000100191 | 3.366 | 0.006472 | 0.01154 | SLC5A4 |
| ENSG00000100196 | -1.294 | 3.596e-23 | 2.393e-22 | KDELR3 |
| ENSG00000100211 | -2.085 | 9.52e-74 | 3.371e-72 | CBY1 |
| ENSG00000100219 | 1.684 | 1.088e-67 | 3.327e-66 | XBP1 |
| ENSG00000100226 | 1.064 | 1.748e-24 | 1.24e-23 | GTPBP1 |
| ENSG00000100228 | -1.298 | 7.881e-11 | 2.795e-10 | RAB36 |
| ENSG00000100253 | 4.048 | 0.0002087 | 0.000446 | MIOX |
| ENSG00000100271 | -1.627 | 0.001295 | 0.002537 | TTLL1 |
| ENSG00000100285 | -1.066 | 2.403e-06 | 6.12e-06 | NEFH |
| ENSG00000100288 | -1.27 | 1.672e-09 | 5.436e-09 | CHKB |
| ENSG00000100292 | 8.788 | 0 | 0 | HMOX1 |
| ENSG00000100294 | -2.294 | 5.799e-24 | 4.014e-23 | MCAT |
| ENSG00000100297 | -2.261 | 6.128e-130 | 7.193e-128 | MCM5 |
| ENSG00000100299 | -1.135 | 4.652e-06 | 1.157e-05 | ARSA |
| ENSG00000100302 | -1.714 | 9.825e-09 | 3.018e-08 | RASD2 |
| ENSG00000100304 | -1.408 | 1.274e-66 | 3.773e-65 | TTLL12 |
| ENSG00000100307 | -1.953 | 5.151e-14 | 2.207e-13 | CBX7 |
| ENSG00000100311 | -2.603 | 6.292e-77 | 2.406e-75 | PDGFB |
| ENSG00000100314 | 3.442 | 2.53e-13 | 1.046e-12 | CABP7 |
| ENSG00000100324 | -3.11 | 1.578e-51 | 2.949e-50 | TAB1 |
| ENSG00000100330 | 1.159 | 2.67e-20 | 1.56e-19 | MTMR3 |
| ENSG00000100336 | 2.98 | 9.906e-05 | 0.0002192 | APOL4 |
| ENSG00000100344 | -2.553 | 3.174e-30 | 2.857e-29 | PNPLA3 |
| ENSG00000100346 | -2.5 | 1.158e-09 | 3.813e-09 | CACNA1I |
| ENSG00000100350 | -3.241 | 9.99e-110 | 7.799e-108 | FOXRED2 |
| ENSG00000100364 | -1.003 | 2.481e-20 | 1.454e-19 | KIAA0930 |
| ENSG00000100368 | 1.307 | 0.02862 | 0.04588 | CSF2RB |
| ENSG00000100376 | -1.678 | 1.837e-16 | 8.896e-16 | FAM118A |
| ENSG00000100379 | -1.355 | 2.16e-15 | 9.916e-15 | KCTD17 |
| ENSG00000100380 | 1.449 | 3.847e-50 | 6.951e-49 | ST13 |
| ENSG00000100416 | -1.428 | 3.181e-14 | 1.377e-13 | TRMU |
| ENSG00000100422 | -1.042 | 1.168e-14 | 5.166e-14 | CERK |
| ENSG00000100426 | -1.134 | 1.403e-24 | 9.987e-24 | ZBED4 |
| ENSG00000100427 | 6.454 | 8.22e-13 | 3.294e-12 | MLC1 |
| ENSG00000100429 | -1.279 | 1.047e-13 | 4.423e-13 | HDAC10 |
| ENSG00000100479 | -2.695 | 1.737e-20 | 1.025e-19 | POLE2 |
| ENSG00000100503 | -1.674 | 1.97e-33 | 2.03e-32 | NIN |
| ENSG00000100505 | -2.312 | 1.632e-18 | 8.792e-18 | TRIM9 |
| ENSG00000100575 | -1.463 | 2.221e-25 | 1.644e-24 | TIMM9 |
| ENSG00000100578 | -1.935 | 3.174e-23 | 2.117e-22 | KIAA0586 |
| ENSG00000100583 | -2.231 | 5.03e-17 | 2.513e-16 | SAMD15 |
| ENSG00000100591 | 1.859 | 2.111e-92 | 1.185e-90 | AHSA1 |
| ENSG00000100599 | -1.921 | 1.63e-43 | 2.437e-42 | RIN3 |
| ENSG00000100604 | 4.015 | 1.707e-10 | 5.926e-10 | CHGA |
| ENSG00000100626 | -2.008 | 4.717e-11 | 1.699e-10 | GALNT16 |
| ENSG00000100628 | 3.174 | 0.009059 | 0.0158 | ASB2 |
| ENSG00000100664 | 1.352 | 2.54e-46 | 4.112e-45 | EIF5 |
| ENSG00000100697 | -1.564 | 4.688e-31 | 4.369e-30 | DICER1 |
| ENSG00000100711 | -1.104 | 7.774e-13 | 3.12e-12 | ZFYVE21 |
| ENSG00000100722 | -1.144 | 3.866e-11 | 1.402e-10 | ZC3H14 |
| ENSG00000100726 | -2.002 | 5.798e-21 | 3.505e-20 | TELO2 |
| ENSG00000100802 | -1.167 | 1.469e-06 | 3.821e-06 | C14orf93 |
| ENSG00000100842 | -2.608 | 2.009e-05 | 4.728e-05 | EFS |
| ENSG00000100884 | 3.492 | 0.0007399 | 0.001488 | CPNE6 |
| ENSG00000100890 | -1.109 | 0.0002014 | 0.0004311 | KIAA0391 |
| ENSG00000100902 | 1.194 | 4.992e-12 | 1.909e-11 | PSMA6 |
| ENSG00000100916 | -1.152 | 1.45e-20 | 8.574e-20 | BRMS1L |
| ENSG00000100918 | -1.002 | 0.009661 | 0.01678 | REC8 |
| ENSG00000100968 | -1.774 | 0.0001327 | 0.0002892 | NFATC4 |
| ENSG00000100982 | -1.147 | 1.725e-17 | 8.808e-17 | PCIF1 |
| ENSG00000100987 | -4.445 | 8.767e-09 | 2.704e-08 | VSX1 |
| ENSG00000101003 | -4.065 | 7.107e-159 | 1.326e-156 | GINS1 |
| ENSG00000101004 | -2.043 | 3.364e-27 | 2.681e-26 | NINL |
| ENSG00000101098 | -2.671 | 0.02469 | 0.04003 | RIMS4 |
| ENSG00000101109 | 1.778 | 2.405e-72 | 8.171e-71 | STK4 |
| ENSG00000101138 | -1.265 | 1.588e-15 | 7.327e-15 | CSTF1 |
| ENSG00000101166 | 1.144 | 7.604e-33 | 7.684e-32 | SLMO2 |
| ENSG00000101187 | -1.219 | 3.167e-14 | 1.372e-13 | SLCO4A1 |
| ENSG00000101188 | -3.522 | 2.384e-11 | 8.765e-11 | NTSR1 |
| ENSG00000101189 | -1.033 | 4.858e-10 | 1.636e-09 | MRGBP |
| ENSG00000101190 | -1.964 | 2.724e-31 | 2.576e-30 | TCFL5 |
| ENSG00000101197 | -2.926 | 0.01307 | 0.02224 | BIRC7 |
| ENSG00000101203 | 6.732 | 6.842e-18 | 3.569e-17 | COL20A1 |
| ENSG00000101213 | -1.794 | 8.85e-12 | 3.328e-11 | PTK6 |
| ENSG00000101220 | -1.159 | 2.076e-07 | 5.794e-07 | C20orf27 |
| ENSG00000101222 | -2.974 | 4.819e-06 | 1.196e-05 | SPEF1 |
| ENSG00000101224 | -3.53 | 1.071e-111 | 8.649e-110 | CDC25B |
| ENSG00000101246 | -2.242 | 8.586e-28 | 7.017e-27 | ARFRP1 |
| ENSG00000101255 | 2.025 | 5.489e-50 | 9.866e-49 | TRIB3 |
| ENSG00000101276 | -2.997 | 1.282e-37 | 1.57e-36 | SLC52A3 |
| ENSG00000101280 | 2.044 | 0.03122 | 0.04973 | ANGPT4 |
| ENSG00000101290 | -1.661 | 9.719e-67 | 2.893e-65 | CDS2 |
| ENSG00000101298 | -2.338 | 1.775e-09 | 5.756e-09 | SNPH |
| ENSG00000101346 | -2.447 | 3.241e-66 | 9.494e-65 | POFUT1 |
| ENSG00000101347 | 3.024 | 9.712e-164 | 1.964e-161 | SAMHD1 |
| ENSG00000101361 | -1.558 | 1.686e-36 | 1.958e-35 | NOP56 |
| ENSG00000101407 | -1.748 | 3.019e-61 | 7.667e-60 | TTI1 |
| ENSG00000101412 | -1.923 | 1.684e-25 | 1.256e-24 | E2F1 |
| ENSG00000101417 | -3.182 | 7.758e-29 | 6.638e-28 | PXMP4 |
| ENSG00000101442 | -1.531 | 5.384e-11 | 1.934e-10 | ACTR5 |
| ENSG00000101447 | -3.319 | 4.573e-153 | 7.914e-151 | FAM83D |
| ENSG00000101448 | 2.376 | 0.02855 | 0.04579 | EPPIN |
| ENSG00000101470 | 1.845 | 0.02766 | 0.04444 | TNNC2 |
| ENSG00000101544 | 1.868 | 2.314e-126 | 2.572e-124 | ADNP2 |
| ENSG00000101546 | -1.113 | 6.264e-15 | 2.813e-14 | RBFA |
| ENSG00000101577 | -1.253 | 3.189e-14 | 1.38e-13 | LPIN2 |
| ENSG00000101639 | -1.418 | 1.115e-36 | 1.306e-35 | CEP192 |
| ENSG00000101654 | 1.525 | 1.092e-67 | 3.334e-66 | RNMT |
| ENSG00000101665 | 1.169 | 1.112e-12 | 4.422e-12 | SMAD7 |
| ENSG00000101670 | 1.571 | 2.195e-48 | 3.756e-47 | LIPG |
| ENSG00000101680 | 4.994 | 4.347e-15 | 1.969e-14 | LAMA1 |
| ENSG00000101745 | 1.865 | 4.924e-18 | 2.59e-17 | ANKRD12 |
| ENSG00000101751 | -1.992 | 1.936e-23 | 1.306e-22 | POLI |
| ENSG00000101773 | -1.204 | 5.639e-26 | 4.293e-25 | RBBP8 |
| ENSG00000101782 | 2.029 | 1.463e-151 | 2.431e-149 | RIOK3 |
| ENSG00000101842 | 3.234 | 0.001857 | 0.003576 | VSIG1 |
| ENSG00000101846 | -1.137 | 3.196e-12 | 1.235e-11 | STS |
| ENSG00000101871 | -1.963 | 1.967e-80 | 8.215e-79 | MID1 |
| ENSG00000101882 | -1.013 | 2.442e-21 | 1.502e-20 | NKAP |
| ENSG00000101888 | 1.762 | 7.331e-102 | 4.982e-100 | NXT2 |
| ENSG00000101911 | -1.155 | 1.577e-37 | 1.924e-36 | PRPS2 |
| ENSG00000101945 | -2.766 | 2.008e-45 | 3.169e-44 | SUV39H1 |
| ENSG00000102043 | -1.912 | 6.567e-05 | 0.0001477 | MTMR8 |
| ENSG00000102057 | 2.761 | 5.109e-09 | 1.605e-08 | KCND1 |
| ENSG00000102096 | 1.212 | 5.212e-09 | 1.637e-08 | PIM2 |
| ENSG00000102098 | -1.477 | 1.106e-13 | 4.665e-13 | SCML2 |
| ENSG00000102125 | -1.358 | 4.373e-22 | 2.774e-21 | TAZ |
| ENSG00000102178 | -3.055 | 4.655e-71 | 1.529e-69 | UBL4A |
| ENSG00000102181 | -1.755 | 8.116e-47 | 1.342e-45 | CD99L2 |
| ENSG00000102221 | -1.771 | 2.241e-19 | 1.253e-18 | PHF16 |
| ENSG00000102239 | 3.029 | 0.0276 | 0.04436 | BRS3 |
| ENSG00000102302 | -2.164 | 3.073e-13 | 1.262e-12 | FGD1 |
| ENSG00000102349 | -2.85 | 2.043e-31 | 1.947e-30 | KLF8 |
| ENSG00000102384 | -2.108 | 2.045e-34 | 2.219e-33 | CENPI |
| ENSG00000102385 | 1.421 | 1.995e-06 | 5.119e-06 | DRP2 |
| ENSG00000102393 | 1.75 | 5.047e-41 | 6.944e-40 | GLA |
| ENSG00000102401 | -1.067 | 4.277e-08 | 1.254e-07 | ARMCX3 |
| ENSG00000102409 | 3.879 | 3.961e-06 | 9.917e-06 | BEX4 |
| ENSG00000102445 | 2.404 | 0.02374 | 0.03859 | KIAA0226L |
| ENSG00000102466 | -1.292 | 2.109e-06 | 5.394e-06 | FGF14 |
| ENSG00000102543 | -1.292 | 0.0001066 | 0.0002352 | CDADC1 |
| ENSG00000102580 | 2.253 | 9.354e-93 | 5.322e-91 | DNAJC3 |
| ENSG00000102699 | -2.654 | 5.891e-128 | 6.727e-126 | PARP4 |
| ENSG00000102743 | -2.859 | 8.808e-38 | 1.088e-36 | SLC25A15 |
| ENSG00000102786 | 1.196 | 2.419e-22 | 1.551e-21 | INTS6 |
| ENSG00000102804 | -1.204 | 7.832e-33 | 7.901e-32 | TSC22D1 |
| ENSG00000102805 | -1.623 | 7.341e-24 | 5.057e-23 | CLN5 |
| ENSG00000102870 | -2.49 | 1.179e-48 | 2.049e-47 | ZNF629 |
| ENSG00000102871 | -2.285 | 3.294e-24 | 2.305e-23 | TRADD |
| ENSG00000102890 | -1.061 | 4.189e-08 | 1.23e-07 | ELMO3 |
| ENSG00000102900 | -1.481 | 7.343e-37 | 8.705e-36 | NUP93 |
| ENSG00000102908 | 1.398 | 1.306e-15 | 6.066e-15 | NFAT5 |
| ENSG00000102910 | -1.367 | 4.47e-43 | 6.565e-42 | LONP2 |
| ENSG00000102921 | -1.371 | 9.021e-26 | 6.802e-25 | N4BP1 |
| ENSG00000102934 | -3.659 | 6.435e-36 | 7.338e-35 | PLLP |
| ENSG00000102967 | -2.149 | 3.852e-21 | 2.348e-20 | DHODH |
| ENSG00000102981 | -2.465 | 3.411e-09 | 1.085e-08 | PARD6A |
| ENSG00000102996 | -2.501 | 2.704e-82 | 1.182e-80 | MMP15 |
| ENSG00000103021 | -1.777 | 3.694e-43 | 5.454e-42 | CCDC113 |
| ENSG00000103024 | -1.959 | 2.072e-08 | 6.219e-08 | NME3 |
| ENSG00000103034 | -1.318 | 2.359e-09 | 7.58e-09 | NDRG4 |
| ENSG00000103037 | -2.305 | 5.715e-34 | 6.053e-33 | SETD6 |
| ENSG00000103044 | -3.741 | 1.164e-45 | 1.85e-44 | HAS3 |
| ENSG00000103047 | -2.042 | 3.293e-21 | 2.012e-20 | TANGO6 |
| ENSG00000103051 | -1.152 | 1.591e-09 | 5.185e-09 | COG4 |
| ENSG00000103067 | -2.008 | 5.794e-92 | 3.21e-90 | ESRP2 |
| ENSG00000103145 | 1.443 | 1.544e-18 | 8.33e-18 | HCFC1R1 |
| ENSG00000103148 | -1.041 | 2.302e-13 | 9.546e-13 | NPRL3 |
| ENSG00000103150 | -1.607 | 4.507e-08 | 1.32e-07 | MLYCD |
| ENSG00000103154 | 5.745 | 1.267e-27 | 1.027e-26 | NECAB2 |
| ENSG00000103160 | -3.21 | 4.639e-62 | 1.209e-60 | HSDL1 |
| ENSG00000103199 | -2.123 | 8.171e-26 | 6.172e-25 | ZNF500 |
| ENSG00000103245 | -1.445 | 7.538e-20 | 4.318e-19 | NARFL |
| ENSG00000103253 | -1.354 | 9.229e-09 | 2.84e-08 | HAGHL |
| ENSG00000103254 | -1.616 | 4.796e-07 | 1.301e-06 | FAM173A |
| ENSG00000103257 | 1.772 | 2.084e-62 | 5.508e-61 | SLC7A5 |
| ENSG00000103264 | -1.186 | 3.909e-15 | 1.774e-14 | FBXO31 |
| ENSG00000103266 | -1.189 | 4.213e-18 | 2.224e-17 | STUB1 |
| ENSG00000103313 | 3.161 | 0.000153 | 0.0003312 | MEFV |
| ENSG00000103316 | 4.411 | 4.103e-08 | 1.205e-07 | CRYM |
| ENSG00000103319 | -3.108 | 1.079e-99 | 6.916e-98 | EEF2K |
| ENSG00000103351 | -1.331 | 1.473e-22 | 9.539e-22 | CLUAP1 |
| ENSG00000103365 | -1.22 | 1.087e-15 | 5.073e-15 | GGA2 |
| ENSG00000103381 | -1.282 | 4.259e-23 | 2.819e-22 | CPPED1 |
| ENSG00000103404 | -1.756 | 8.797e-54 | 1.752e-52 | USP31 |
| ENSG00000103415 | -1.781 | 2.946e-34 | 3.17e-33 | HMOX2 |
| ENSG00000103426 | 6.841 | 0.0002442 | 0.000518 | CORO7-PAM16 |
| ENSG00000103494 | -1.672 | 1.165e-27 | 9.472e-27 | RPGRIP1L |
| ENSG00000103495 | -1.853 | 9.919e-75 | 3.612e-73 | MAZ |
| ENSG00000103502 | -1.275 | 1.285e-11 | 4.791e-11 | CDIPT |
| ENSG00000103507 | -1.417 | 3.772e-27 | 2.998e-26 | BCKDK |
| ENSG00000103528 | -1.636 | 4.922e-15 | 2.22e-14 | SYT17 |
| ENSG00000103534 | -3.027 | 4.002e-64 | 1.105e-62 | TMC5 |
| ENSG00000103540 | -2.169 | 1.071e-94 | 6.31e-93 | CCP110 |
| ENSG00000103550 | -1.647 | 5.69e-50 | 1.021e-48 | KNOP1 |
| ENSG00000103647 | 1.461 | 6.793e-06 | 1.667e-05 | CORO2B |
| ENSG00000103653 | -1.295 | 2.936e-14 | 1.273e-13 | CSK |
| ENSG00000103657 | -1.308 | 4.312e-34 | 4.589e-33 | HERC1 |
| ENSG00000103707 | -2.103 | 9.426e-21 | 5.623e-20 | MTFMT |
| ENSG00000103742 | 2.176 | 0.002631 | 0.00496 | IGDCC4 |
| ENSG00000103852 | -1.778 | 6.468e-24 | 4.466e-23 | TTC23 |
| ENSG00000103995 | -1.368 | 9.469e-20 | 5.399e-19 | CEP152 |
| ENSG00000104043 | -2.417 | 0.001992 | 0.003821 | ATP8B4 |
| ENSG00000104047 | -1.933 | 1.782e-18 | 9.576e-18 | DTWD1 |
| ENSG00000104055 | -1.887 | 0.005988 | 0.01073 | TGM5 |
| ENSG00000104067 | 1.068 | 5.505e-09 | 1.724e-08 | TJP1 |
| ENSG00000104081 | -4.258 | 2.944e-30 | 2.653e-29 | BMF |
| ENSG00000104093 | -1.496 | 4.512e-22 | 2.86e-21 | DMXL2 |
| ENSG00000104140 | -2.754 | 1.634e-12 | 6.427e-12 | RHOV |
| ENSG00000104142 | -1.495 | 1.146e-10 | 4.028e-10 | VPS18 |
| ENSG00000104147 | -2.411 | 2.327e-16 | 1.124e-15 | OIP5 |
| ENSG00000104154 | -1.722 | 1.318e-06 | 3.442e-06 | SLC30A4 |
| ENSG00000104164 | 1.109 | 6.371e-24 | 4.402e-23 | BLOC1S6 |
| ENSG00000104205 | -1.587 | 1.586e-13 | 6.645e-13 | SGK3 |
| ENSG00000104213 | -1.925 | 0.0007267 | 0.001462 | PDGFRL |
| ENSG00000104218 | -1.627 | 1.495e-16 | 7.272e-16 | CSPP1 |
| ENSG00000104221 | 1.207 | 1.445e-06 | 3.761e-06 | BRF2 |
| ENSG00000104228 | -1.902 | 3.864e-14 | 1.665e-13 | TRIM35 |
| ENSG00000104267 | 1.035 | 2.608e-08 | 7.777e-08 | CA2 |
| ENSG00000104290 | -2.313 | 2.668e-43 | 3.956e-42 | FZD3 |
| ENSG00000104313 | -2.88 | 2.299e-10 | 7.926e-10 | EYA1 |
| ENSG00000104320 | -1.041 | 4.158e-20 | 2.411e-19 | NBN |
| ENSG00000104332 | -1.51 | 1.294e-34 | 1.413e-33 | SFRP1 |
| ENSG00000104365 | -1.301 | 1.863e-28 | 1.564e-27 | IKBKB |
| ENSG00000104368 | -2.617 | 3.723e-59 | 8.753e-58 | PLAT |
| ENSG00000104369 | -3.619 | 4.221e-31 | 3.949e-30 | JPH1 |
| ENSG00000104371 | 3.447 | 0.003902 | 0.007169 | DKK4 |
| ENSG00000104413 | -1.098 | 3.86e-26 | 2.956e-25 | ESRP1 |
| ENSG00000104415 | 3.524 | 0.008035 | 0.01414 | WISP1 |
| ENSG00000104419 | 2.093 | 6.8e-60 | 1.657e-58 | NDRG1 |
| ENSG00000104427 | -1.522 | 2.333e-07 | 6.489e-07 | ZC2HC1A |
| ENSG00000104442 | -2.099 | 3.355e-50 | 6.081e-49 | ARMC1 |
| ENSG00000104447 | -2.203 | 1.426e-15 | 6.597e-15 | TRPS1 |
| ENSG00000104518 | -1.523 | 8.086e-10 | 2.688e-09 | GSDMD |
| ENSG00000104524 | -3.918 | 1.041e-63 | 2.818e-62 | PYCRL |
| ENSG00000104549 | -1.643 | 7.422e-82 | 3.186e-80 | SQLE |
| ENSG00000104611 | -2.163 | 2.61e-53 | 5.136e-52 | SH2D4A |
| ENSG00000104613 | -1.9 | 4.614e-32 | 4.524e-31 | INTS10 |
| ENSG00000104635 | 1.067 | 2.37e-09 | 7.613e-09 | SLC39A14 |
| ENSG00000104679 | -1.853 | 5.712e-25 | 4.133e-24 | R3HCC1 |
| ENSG00000104687 | 1.212 | 1.945e-39 | 2.526e-38 | GSR |
| ENSG00000104695 | 1.345 | 2.108e-39 | 2.73e-38 | PPP2CB |
| ENSG00000104714 | 1.142 | 2.529e-21 | 1.553e-20 | ERICH1 |
| ENSG00000104738 | -2.11 | 2.061e-127 | 2.322e-125 | MCM4 |
| ENSG00000104765 | 1.229 | 1.101e-33 | 1.15e-32 | BNIP3L |
| ENSG00000104783 | -2.426 | 1.333e-38 | 1.69e-37 | KCNN4 |
| ENSG00000104804 | 5.056 | 4.8e-07 | 1.302e-06 | TULP2 |
| ENSG00000104808 | 3.318 | 5.458e-10 | 1.832e-09 | DHDH |
| ENSG00000104825 | 1.04 | 7.144e-17 | 3.538e-16 | NFKBIB |
| ENSG00000104826 | 2.832 | 3.996e-05 | 9.171e-05 | LHB |
| ENSG00000104848 | 2.461 | 2.404e-06 | 6.121e-06 | KCNA7 |
| ENSG00000104856 | 1.774 | 4.845e-21 | 2.938e-20 | RELB |
| ENSG00000104863 | -2.601 | 1.648e-07 | 4.64e-07 | LIN7B |
| ENSG00000104866 | -1.74 | 2.36e-37 | 2.852e-36 | PPP1R37 |
| ENSG00000104879 | 4.264 | 5.155e-10 | 1.733e-09 | CKM |
| ENSG00000104883 | -1.164 | 0.007664 | 0.01352 | PEX11G |
| ENSG00000104886 | -1.762 | 1.134e-14 | 5.02e-14 | PLEKHJ1 |
| ENSG00000104967 | -1.654 | 2.338e-05 | 5.472e-05 | NOVA2 |
| ENSG00000104976 | -1.551 | 5.754e-13 | 2.325e-12 | SNAPC2 |
| ENSG00000104983 | -1.585 | 4.976e-10 | 1.675e-09 | CCDC61 |
| ENSG00000104998 | -1.543 | 5.428e-11 | 1.948e-10 | IL27RA |
| ENSG00000105011 | -2.916 | 5.687e-135 | 7.123e-133 | ASF1B |
| ENSG00000105053 | 1.053 | 2.633e-32 | 2.607e-31 | VRK3 |
| ENSG00000105058 | 1.451 | 8.3e-42 | 1.175e-40 | FAM32A |
| ENSG00000105137 | -2.966 | 1.252e-22 | 8.13e-22 | SYDE1 |
| ENSG00000105143 | -1.523 | 3.598e-08 | 1.062e-07 | SLC1A6 |
| ENSG00000105173 | -1.271 | 4.31e-14 | 1.855e-13 | CCNE1 |
| ENSG00000105219 | 3.005 | 7.953e-09 | 2.464e-08 | CNTD2 |
| ENSG00000105245 | 1.517 | 4.746e-12 | 1.816e-11 | NUMBL |
| ENSG00000105248 | 1.241 | 5.679e-18 | 2.978e-17 | CCDC94 |
| ENSG00000105251 | 4.51 | 1.204e-05 | 2.889e-05 | SHD |
| ENSG00000105289 | -1.474 | 7.944e-11 | 2.816e-10 | TJP3 |
| ENSG00000105327 | 1.397 | 3.386e-05 | 7.814e-05 | BBC3 |
| ENSG00000105376 | -2.043 | 4.424e-18 | 2.333e-17 | ICAM5 |
| ENSG00000105397 | -1.095 | 3.241e-11 | 1.18e-10 | TYK2 |
| ENSG00000105426 | -1.887 | 1.579e-86 | 7.727e-85 | PTPRS |
| ENSG00000105429 | -1.536 | 9.341e-43 | 1.356e-41 | MEGF8 |
| ENSG00000105464 | -1.905 | 8.306e-23 | 5.429e-22 | GRIN2D |
| ENSG00000105483 | -1.504 | 3.404e-18 | 1.805e-17 | CARD8 |
| ENSG00000105486 | -1.275 | 3.564e-23 | 2.372e-22 | LIG1 |
| ENSG00000105497 | 2.427 | 1.204e-51 | 2.268e-50 | ZNF175 |
| ENSG00000105499 | 3.415 | 3.413e-10 | 1.162e-09 | PLA2G4C |
| ENSG00000105514 | -3.435 | 2.472e-47 | 4.141e-46 | RAB3D |
| ENSG00000105523 | -2.227 | 2.508e-05 | 5.851e-05 | FAM83E |
| ENSG00000105538 | 2.184 | 0.0001228 | 0.0002684 | RASIP1 |
| ENSG00000105550 | 5.174 | 4.693e-07 | 1.274e-06 | FGF21 |
| ENSG00000105552 | -1.448 | 4.656e-24 | 3.237e-23 | BCAT2 |
| ENSG00000105559 | -1.457 | 2.788e-07 | 7.71e-07 | PLEKHA4 |
| ENSG00000105607 | -1.143 | 2.37e-05 | 5.544e-05 | GCDH |
| ENSG00000105619 | 1.249 | 3.642e-18 | 1.927e-17 | TFPT |
| ENSG00000105639 | -1.013 | 0.0117 | 0.02006 | JAK3 |
| ENSG00000105641 | 2.849 | 5.694e-05 | 0.0001289 | SLC5A5 |
| ENSG00000105642 | 4.911 | 4.263e-24 | 2.967e-23 | KCNN1 |
| ENSG00000105647 | -1.398 | 2.353e-47 | 3.946e-46 | PIK3R2 |
| ENSG00000105656 | 2.165 | 1.006e-94 | 5.947e-93 | ELL |
| ENSG00000105662 | -1.216 | 1.486e-15 | 6.862e-15 | CRTC1 |
| ENSG00000105676 | -2.208 | 9.398e-54 | 1.867e-52 | ARMC6 |
| ENSG00000105717 | 2.033 | 3.874e-26 | 2.965e-25 | PBX4 |
| ENSG00000105722 | -1.166 | 4.683e-24 | 3.255e-23 | ERF |
| ENSG00000105737 | 1.486 | 0.0147 | 0.02481 | GRIK5 |
| ENSG00000105738 | -1.483 | 2.112e-40 | 2.832e-39 | SIPA1L3 |
| ENSG00000105810 | -1.531 | 4.192e-27 | 3.324e-26 | CDK6 |
| ENSG00000105821 | 1.682 | 1.187e-15 | 5.527e-15 | DNAJC2 |
| ENSG00000105825 | 1.939 | 3.331e-92 | 1.864e-90 | TFPI2 |
| ENSG00000105849 | -1.187 | 8.41e-17 | 4.151e-16 | TWISTNB |
| ENSG00000105856 | 2.624 | 2.77e-130 | 3.275e-128 | HBP1 |
| ENSG00000105879 | 1.629 | 4.928e-13 | 2.002e-12 | CBLL1 |
| ENSG00000105926 | -1.277 | 8.762e-27 | 6.87e-26 | MPP6 |
| ENSG00000105948 | -2.076 | 9.677e-27 | 7.563e-26 | TTC26 |
| ENSG00000105967 | 2.815 | 0.0005979 | 0.001217 | TFEC |
| ENSG00000105971 | -1.242 | 1.829e-40 | 2.462e-39 | CAV2 |
| ENSG00000105974 | -1.195 | 1.67e-48 | 2.887e-47 | CAV1 |
| ENSG00000105991 | -2.843 | 0.02042 | 0.03363 | HOXA1 |
| ENSG00000105993 | 2.806 | 3.914e-110 | 3.084e-108 | DNAJB6 |
| ENSG00000105996 | -3.541 | 5.037e-07 | 1.363e-06 | HOXA2 |
| ENSG00000105997 | -3.181 | 3.833e-25 | 2.792e-24 | HOXA3 |
| ENSG00000106003 | -3.095 | 9.213e-23 | 6.015e-22 | LFNG |
| ENSG00000106004 | -3.143 | 1.497e-10 | 5.221e-10 | HOXA5 |
| ENSG00000106006 | -2.617 | 2.343e-08 | 7.006e-08 | HOXA6 |
| ENSG00000106009 | -1.729 | 4.357e-34 | 4.635e-33 | BRAT1 |
| ENSG00000106012 | -1.793 | 8.945e-67 | 2.672e-65 | IQCE |
| ENSG00000106025 | -2.529 | 7.675e-17 | 3.795e-16 | TSPAN12 |
| ENSG00000106052 | 1.553 | 1.138e-20 | 6.766e-20 | TAX1BP1 |
| ENSG00000106069 | -2.238 | 1.17e-32 | 1.172e-31 | CHN2 |
| ENSG00000106070 | 2.031 | 3.869e-68 | 1.194e-66 | GRB10 |
| ENSG00000106089 | 1.756 | 8.546e-33 | 8.605e-32 | STX1A |
| ENSG00000106100 | -3.519 | 5.07e-65 | 1.438e-63 | NOD1 |
| ENSG00000106105 | 2.235 | 6.088e-148 | 9.375e-146 | GARS |
| ENSG00000106113 | 2.827 | 0.003904 | 0.007171 | CRHR2 |
| ENSG00000106123 | -1.434 | 7.037e-24 | 4.851e-23 | EPHB6 |
| ENSG00000106125 | -2.552 | 2.4e-55 | 4.98e-54 | FAM188B |
| ENSG00000106144 | -1.61 | 3.484e-57 | 7.656e-56 | CASP2 |
| ENSG00000106178 | 6.03 | 3.598e-17 | 1.81e-16 | CCL24 |
| ENSG00000106211 | 1.8 | 1.56e-55 | 3.248e-54 | HSPB1 |
| ENSG00000106245 | 1.287 | 1.351e-21 | 8.412e-21 | BUD31 |
| ENSG00000106246 | -1.079 | 2.066e-06 | 5.291e-06 | PTCD1 |
| ENSG00000106258 | 1.442 | 0.003255 | 0.006047 | CYP3A5 |
| ENSG00000106290 | -1.479 | 5.665e-19 | 3.115e-18 | TAF6 |
| ENSG00000106305 | -1.886 | 3.37e-39 | 4.352e-38 | AIMP2 |
| ENSG00000106327 | -1.53 | 6.466e-06 | 1.589e-05 | TFR2 |
| ENSG00000106351 | -3.707 | 4.348e-35 | 4.817e-34 | AGFG2 |
| ENSG00000106366 | 1.395 | 1.668e-54 | 3.377e-53 | SERPINE1 |
| ENSG00000106392 | -1.315 | 1.094e-30 | 1.006e-29 | C1GALT1 |
| ENSG00000106404 | 1.667 | 1.042e-18 | 5.661e-18 | CLDN15 |
| ENSG00000106459 | -1.123 | 8.378e-09 | 2.591e-08 | NRF1 |
| ENSG00000106460 | -1.206 | 1.969e-35 | 2.217e-34 | TMEM106B |
| ENSG00000106462 | -1.131 | 3.545e-13 | 1.452e-12 | EZH2 |
| ENSG00000106477 | -1.599 | 2.126e-38 | 2.671e-37 | CEP41 |
| ENSG00000106484 | -1.512 | 1.245e-28 | 1.056e-27 | MEST |
| ENSG00000106546 | 1.388 | 7.734e-28 | 6.336e-27 | AHR |
| ENSG00000106603 | -1.072 | 2.024e-21 | 1.252e-20 | COA1 |
| ENSG00000106624 | 3.079 | 0.0005311 | 0.001087 | AEBP1 |
| ENSG00000106683 | -1.472 | 8.28e-31 | 7.658e-30 | LIMK1 |
| ENSG00000106688 | -3.852 | 3.601e-39 | 4.646e-38 | SLC1A1 |
| ENSG00000106689 | -3.468 | 6.84e-12 | 2.591e-11 | LHX2 |
| ENSG00000106701 | -2.123 | 6.664e-14 | 2.839e-13 | FSD1L |
| ENSG00000106714 | -2.218 | 2.127e-11 | 7.851e-11 | CNTNAP3 |
| ENSG00000106771 | -1.541 | 1.303e-55 | 2.72e-54 | TMEM245 |
| ENSG00000106780 | -1.336 | 1.951e-41 | 2.724e-40 | MEGF9 |
| ENSG00000106785 | -2.089 | 7.336e-55 | 1.505e-53 | TRIM14 |
| ENSG00000106803 | 1.005 | 1.014e-11 | 3.805e-11 | SEC61B |
| ENSG00000106804 | -1.003 | 4.722e-06 | 1.174e-05 | C5 |
| ENSG00000106852 | -2.679 | 0.002422 | 0.004587 | LHX6 |
| ENSG00000106991 | -2.549 | 7.054e-65 | 1.993e-63 | ENG |
| ENSG00000107014 | -1.694 | 0.01493 | 0.02518 | RLN2 |
| ENSG00000107021 | -2.124 | 7.091e-38 | 8.765e-37 | TBC1D13 |
| ENSG00000107099 | -1.382 | 1.368e-10 | 4.783e-10 | DOCK8 |
| ENSG00000107104 | -2.34 | 4.127e-49 | 7.27e-48 | KANK1 |
| ENSG00000107165 | 4.397 | 2.53e-05 | 5.9e-05 | TYRP1 |
| ENSG00000107187 | 3.436 | 0.004445 | 0.008097 | LHX3 |
| ENSG00000107281 | -1.397 | 1.6e-11 | 5.935e-11 | NPDC1 |
| ENSG00000107372 | 2.086 | 3.35e-131 | 3.988e-129 | ZFAND5 |
| ENSG00000107443 | -1.462 | 2.979e-18 | 1.583e-17 | CCNJ |
| ENSG00000107554 | -1.676 | 4.108e-43 | 6.044e-42 | DNMBP |
| ENSG00000107566 | -1.71 | 1.022e-54 | 2.088e-53 | ERLIN1 |
| ENSG00000107593 | 3.438 | 0.004719 | 0.008568 | PKD2L1 |
| ENSG00000107618 | -2.305 | 0.006062 | 0.01085 | RBP3 |
| ENSG00000107672 | -2.624 | 3.149e-49 | 5.559e-48 | NSMCE4A |
| ENSG00000107679 | -1.177 | 1.69e-18 | 9.096e-18 | PLEKHA1 |
| ENSG00000107719 | -2.362 | 2.45e-34 | 2.643e-33 | PALD1 |
| ENSG00000107738 | -1.367 | 2.245e-12 | 8.758e-12 | C10orf54 |
| ENSG00000107779 | -1.672 | 1.84e-30 | 1.673e-29 | BMPR1A |
| ENSG00000107789 | -2.465 | 2.817e-25 | 2.07e-24 | MINPP1 |
| ENSG00000107816 | -2.706 | 8.283e-68 | 2.537e-66 | LZTS2 |
| ENSG00000107819 | -1.515 | 5.032e-34 | 5.333e-33 | SFXN3 |
| ENSG00000107821 | -1.413 | 7.92e-08 | 2.281e-07 | KAZALD1 |
| ENSG00000107829 | -1.136 | 9.217e-12 | 3.465e-11 | FBXW4 |
| ENSG00000107863 | 1.355 | 5.714e-15 | 2.568e-14 | ARHGAP21 |
| ENSG00000107864 | 2.712 | 1.501e-36 | 1.747e-35 | CPEB3 |
| ENSG00000107872 | -3.085 | 5.534e-20 | 3.19e-19 | FBXL15 |
| ENSG00000107882 | -1.724 | 7.005e-25 | 5.044e-24 | SUFU |
| ENSG00000107890 | -1.456 | 7.738e-14 | 3.287e-13 | ANKRD26 |
| ENSG00000107897 | -1.258 | 4.071e-27 | 3.232e-26 | ACBD5 |
| ENSG00000107929 | 1.143 | 1.424e-30 | 1.302e-29 | LARP4B |
| ENSG00000107951 | -1.8 | 5.112e-37 | 6.103e-36 | MTPAP |
| ENSG00000107968 | -1.144 | 4.661e-05 | 0.0001064 | MAP3K8 |
| ENSG00000107984 | -2.549 | 8.167e-160 | 1.54e-157 | DKK1 |
| ENSG00000108001 | 2.602 | 0.01961 | 0.0324 | EBF3 |
| ENSG00000108091 | -1.146 | 2.855e-15 | 1.304e-14 | CCDC6 |
| ENSG00000108219 | -1.356 | 3.688e-65 | 1.051e-63 | TSPAN14 |
| ENSG00000108239 | -1.05 | 4.618e-14 | 1.986e-13 | TBC1D12 |
| ENSG00000108256 | -1.084 | 4.809e-30 | 4.294e-29 | NUFIP2 |
| ENSG00000108264 | -1.952 | 2.009e-21 | 1.244e-20 | TADA2A |
| ENSG00000108272 | -1.916 | 5.304e-16 | 2.512e-15 | DHRS11 |
| ENSG00000108292 | -1.321 | 4.609e-59 | 1.076e-57 | MLLT6 |
| ENSG00000108296 | 2.281 | 4.901e-102 | 3.358e-100 | CWC25 |
| ENSG00000108312 | -3.687 | 5.948e-209 | 2.496e-206 | UBTF |
| ENSG00000108349 | -1.168 | 2.465e-19 | 1.377e-18 | CASC3 |
| ENSG00000108352 | -1.404 | 2.556e-10 | 8.776e-10 | RAPGEFL1 |
| ENSG00000108384 | -1.043 | 2.453e-17 | 1.243e-16 | RAD51C |
| ENSG00000108395 | -1.398 | 2.097e-22 | 1.349e-21 | TRIM37 |
| ENSG00000108433 | 1.905 | 3.685e-95 | 2.193e-93 | GOSR2 |
| ENSG00000108439 | -2.052 | 6.041e-23 | 3.971e-22 | PNPO |
| ENSG00000108465 | -1.104 | 1.269e-11 | 4.734e-11 | CDK5RAP3 |
| ENSG00000108468 | -1.009 | 4.133e-27 | 3.28e-26 | CBX1 |
| ENSG00000108511 | -3.678 | 6.838e-49 | 1.197e-47 | HOXB6 |
| ENSG00000108528 | -1.466 | 2.842e-11 | 1.039e-10 | SLC25A11 |
| ENSG00000108551 | 4.154 | 1.255e-17 | 6.457e-17 | RASD1 |
| ENSG00000108556 | 2.453 | 0.009507 | 0.01653 | CHRNE |
| ENSG00000108557 | -1.103 | 1.324e-25 | 9.912e-25 | RAI1 |
| ENSG00000108578 | -1.12 | 6.058e-11 | 2.168e-10 | BLMH |
| ENSG00000108604 | -2.204 | 9.631e-38 | 1.189e-36 | SMARCD2 |
| ENSG00000108641 | -1.027 | 9.566e-09 | 2.943e-08 | B9D1 |
| ENSG00000108669 | 1.324 | 3.987e-35 | 4.43e-34 | CYTH1 |
| ENSG00000108771 | -1.914 | 1.241e-08 | 3.781e-08 | DHX58 |
| ENSG00000108773 | -1.371 | 1.281e-30 | 1.173e-29 | KAT2A |
| ENSG00000108784 | -2.743 | 2.058e-31 | 1.959e-30 | NAGLU |
| ENSG00000108797 | -1.429 | 0.000492 | 0.00101 | CNTNAP1 |
| ENSG00000108813 | -2.227 | 3.7e-06 | 9.281e-06 | DLX4 |
| ENSG00000108819 | -2.395 | 1.002e-88 | 5.114e-87 | PPP1R9B |
| ENSG00000108852 | -2.902 | 5.146e-16 | 2.442e-15 | MPP2 |
| ENSG00000108861 | -1.012 | 5.555e-24 | 3.848e-23 | DUSP3 |
| ENSG00000108932 | -3.02 | 6.466e-21 | 3.896e-20 | SLC16A6 |
| ENSG00000108960 | -1.715 | 2.219e-18 | 1.187e-17 | MMD |
| ENSG00000108961 | -1.727 | 2.225e-17 | 1.129e-16 | RANGRF |
| ENSG00000108963 | -2.008 | 8.334e-31 | 7.703e-30 | DPH1 |
| ENSG00000109062 | -1.761 | 2.474e-24 | 1.74e-23 | SLC9A3R1 |
| ENSG00000109063 | 1.874 | 1.472e-08 | 4.457e-08 | MYH3 |
| ENSG00000109066 | -1.983 | 1.26e-27 | 1.021e-26 | TMEM104 |
| ENSG00000109084 | -1.04 | 3.091e-23 | 2.066e-22 | TMEM97 |
| ENSG00000109089 | -2.48 | 2.277e-30 | 2.061e-29 | CDR2L |
| ENSG00000109099 | -1.218 | 8.326e-05 | 0.0001852 | PMP22 |
| ENSG00000109103 | -2.06 | 3.218e-30 | 2.894e-29 | UNC119 |
| ENSG00000109111 | 1.294 | 2.373e-45 | 3.733e-44 | SUPT6H |
| ENSG00000109113 | -1.275 | 3.279e-14 | 1.418e-13 | RAB34 |
| ENSG00000109118 | -1.101 | 3.847e-10 | 1.304e-09 | PHF12 |
| ENSG00000109184 | -2.146 | 2.81e-37 | 3.391e-36 | DCUN1D4 |
| ENSG00000109189 | -1.327 | 1.021e-20 | 6.084e-20 | USP46 |
| ENSG00000109220 | 2.723 | 1.3e-80 | 5.457e-79 | CHIC2 |
| ENSG00000109270 | 2.071 | 5.23e-39 | 6.733e-38 | LAMTOR3 |
| ENSG00000109321 | 3.948 | 1.953e-51 | 3.642e-50 | AREG |
| ENSG00000109323 | -1.133 | 1.385e-16 | 6.758e-16 | MANBA |
| ENSG00000109332 | 1.998 | 2.422e-49 | 4.279e-48 | UBE2D3 |
| ENSG00000109381 | -1.244 | 1.143e-13 | 4.82e-13 | ELF2 |
| ENSG00000109458 | -2.131 | 1.127e-32 | 1.13e-31 | GAB1 |
| ENSG00000109466 | -1.075 | 1.931e-12 | 7.565e-12 | KLHL2 |
| ENSG00000109501 | -2.724 | 9.856e-104 | 6.951e-102 | WFS1 |
| ENSG00000109511 | 1.911 | 2.103e-16 | 1.017e-15 | ANXA10 |
| ENSG00000109572 | -1.243 | 2.465e-43 | 3.661e-42 | CLCN3 |
| ENSG00000109576 | -1.921 | 7.454e-15 | 3.34e-14 | AADAT |
| ENSG00000109586 | -1.519 | 7.021e-41 | 9.573e-40 | GALNT7 |
| ENSG00000109618 | -2.17 | 1.256e-24 | 8.957e-24 | SEPSECS |
| ENSG00000109654 | -1.325 | 8.683e-45 | 1.342e-43 | TRIM2 |
| ENSG00000109667 | 1.261 | 0.02484 | 0.04026 | SLC2A9 |
| ENSG00000109670 | 1.728 | 1.48e-59 | 3.574e-58 | FBXW7 |
| ENSG00000109674 | -3.582 | 1.678e-61 | 4.288e-60 | NEIL3 |
| ENSG00000109685 | -1.304 | 4.388e-59 | 1.026e-57 | WHSC1 |
| ENSG00000109686 | -1.212 | 7.587e-15 | 3.399e-14 | SH3D19 |
| ENSG00000109689 | -1.013 | 6.931e-11 | 2.469e-10 | STIM2 |
| ENSG00000109738 | -2.487 | 2.959e-11 | 1.079e-10 | GLRB |
| ENSG00000109756 | 2.231 | 1.324e-139 | 1.764e-137 | RAPGEF2 |
| ENSG00000109771 | 1.502 | 1.072e-12 | 4.271e-12 | LRP2BP |
| ENSG00000109805 | -2.277 | 3.281e-57 | 7.228e-56 | NCAPG |
| ENSG00000109846 | 4.712 | 3.659e-35 | 4.084e-34 | CRYAB |
| ENSG00000109943 | 4.921 | 7.363e-07 | 1.967e-06 | CRTAM |
| ENSG00000109944 | -1.035 | 0.0006787 | 0.001371 | C11orf63 |
| ENSG00000110042 | -3.038 | 3.028e-122 | 3.157e-120 | DTX4 |
| ENSG00000110057 | -1.139 | 1.546e-17 | 7.905e-17 | UNC93B1 |
| ENSG00000110074 | -1.096 | 1.235e-09 | 4.06e-09 | FOXRED1 |
| ENSG00000110090 | -1.545 | 1.722e-56 | 3.705e-55 | CPT1A |
| ENSG00000110092 | -2.677 | 2.018e-150 | 3.258e-148 | CCND1 |
| ENSG00000110108 | -1.551 | 1.428e-54 | 2.899e-53 | TMEM109 |
| ENSG00000110169 | 3.285 | 1.45e-16 | 7.061e-16 | HPX |
| ENSG00000110171 | 1.474 | 1.791e-23 | 1.209e-22 | TRIM3 |
| ENSG00000110172 | 3.97 | 0 | 0 | CHORDC1 |
| ENSG00000110237 | -2.371 | 4.464e-101 | 2.974e-99 | ARHGEF17 |
| ENSG00000110274 | -2.074 | 7.977e-32 | 7.731e-31 | CEP164 |
| ENSG00000110315 | -1.248 | 1.365e-31 | 1.31e-30 | RNF141 |
| ENSG00000110318 | -1.832 | 4.329e-12 | 1.66e-11 | KIAA1377 |
| ENSG00000110324 | 3.128 | 0.0002284 | 0.000486 | IL10RA |
| ENSG00000110328 | -1.872 | 0.004799 | 0.008705 | GALNT18 |
| ENSG00000110400 | -3.466 | 1.311e-61 | 3.366e-60 | PVRL1 |
| ENSG00000110455 | -2.93 | 8.766e-28 | 7.157e-27 | ACCS |
| ENSG00000110497 | -1.935 | 1.625e-29 | 1.429e-28 | AMBRA1 |
| ENSG00000110583 | -2.29 | 2.467e-41 | 3.426e-40 | NAA40 |
| ENSG00000110619 | 2.176 | 1.507e-86 | 7.396e-85 | CARS |
| ENSG00000110660 | -1.182 | 2.602e-25 | 1.916e-24 | SLC35F2 |
| ENSG00000110786 | 3.303 | 0.01299 | 0.02211 | PTPN5 |
| ENSG00000110841 | -1.102 | 1.033e-25 | 7.776e-25 | PPFIBP1 |
| ENSG00000110844 | -1.14 | 1.45e-07 | 4.104e-07 | PRPF40B |
| ENSG00000110852 | 2.007 | 0.02233 | 0.03648 | CLEC2B |
| ENSG00000110871 | -1.783 | 3.834e-28 | 3.17e-27 | COQ5 |
| ENSG00000110876 | 3.623 | 7.34e-20 | 4.209e-19 | SELPLG |
| ENSG00000110880 | 1.225 | 1.034e-48 | 1.798e-47 | CORO1C |
| ENSG00000110881 | -1.395 | 1.71e-08 | 5.16e-08 | ASIC1 |
| ENSG00000110900 | -1.62 | 7.315e-05 | 0.0001637 | TSPAN11 |
| ENSG00000110906 | 1.007 | 2.455e-28 | 2.049e-27 | KCTD10 |
| ENSG00000110911 | -2.236 | 7.676e-74 | 2.724e-72 | SLC11A2 |
| ENSG00000110917 | -1.382 | 3.689e-32 | 3.628e-31 | MLEC |
| ENSG00000110921 | -2.283 | 1.498e-35 | 1.695e-34 | MVK |
| ENSG00000110931 | -1.252 | 1.761e-33 | 1.818e-32 | CAMKK2 |
| ENSG00000110944 | 2.465 | 0.0003122 | 0.0006554 | IL23A |
| ENSG00000110958 | 1.711 | 8.085e-76 | 3.023e-74 | PTGES3 |
| ENSG00000110987 | -2.323 | 9.065e-39 | 1.156e-37 | BCL7A |
| ENSG00000111011 | 3.036 | 5.043e-146 | 7.362e-144 | RSRC2 |
| ENSG00000111012 | 2.518 | 1.093e-08 | 3.342e-08 | CYP27B1 |
| ENSG00000111052 | 2.12 | 0.03131 | 0.04985 | LIN7A |
| ENSG00000111077 | -1.378 | 2.35e-09 | 7.55e-09 | TENC1 |
| ENSG00000111110 | -4.302 | 1.029e-124 | 1.122e-122 | PPM1H |
| ENSG00000111181 | 5.268 | 8.155e-08 | 2.346e-07 | SLC6A12 |
| ENSG00000111186 | -2.68 | 1.28e-17 | 6.578e-17 | WNT5B |
| ENSG00000111206 | -1.751 | 1.312e-66 | 3.87e-65 | FOXM1 |
| ENSG00000111247 | -1.371 | 5.753e-27 | 4.538e-26 | RAD51AP1 |
| ENSG00000111261 | -2.08 | 1.166e-15 | 5.43e-15 | MANSC1 |
| ENSG00000111266 | 1.381 | 4.247e-36 | 4.886e-35 | DUSP16 |
| ENSG00000111271 | -1.72 | 7.106e-26 | 5.385e-25 | ACAD10 |
| ENSG00000111276 | -3.332 | 4.466e-139 | 5.857e-137 | CDKN1B |
| ENSG00000111291 | 1.415 | 1.316e-05 | 3.147e-05 | GPRC5D |
| ENSG00000111319 | -1.825 | 1.498e-61 | 3.838e-60 | SCNN1A |
| ENSG00000111325 | -1.145 | 0.0004505 | 0.0009281 | OGFOD2 |
| ENSG00000111331 | -1.875 | 1.86e-33 | 1.919e-32 | OAS3 |
| ENSG00000111371 | 1.373 | 1.566e-65 | 4.516e-64 | SLC38A1 |
| ENSG00000111405 | 2.659 | 0.0001091 | 0.0002405 | ENDOU |
| ENSG00000111412 | 1.412 | 5.167e-64 | 1.419e-62 | C12orf49 |
| ENSG00000111424 | -1.91 | 2.731e-41 | 3.782e-40 | VDR |
| ENSG00000111432 | -3 | 0.0004897 | 0.001006 | FZD10 |
| ENSG00000111445 | -1.577 | 7.607e-25 | 5.466e-24 | RFC5 |
| ENSG00000111452 | 3.324 | 0.0001956 | 0.0004188 | GPR133 |
| ENSG00000111490 | -2.223 | 1.528e-30 | 1.394e-29 | TBC1D30 |
| ENSG00000111530 | -1.251 | 5.084e-33 | 5.185e-32 | CAND1 |
| ENSG00000111540 | -1.069 | 7.775e-18 | 4.038e-17 | RAB5B |
| ENSG00000111554 | -1.997 | 3.055e-18 | 1.623e-17 | MDM1 |
| ENSG00000111602 | -2.329 | 3.987e-112 | 3.281e-110 | TIMELESS |
| ENSG00000111641 | -1.016 | 8.267e-27 | 6.484e-26 | NOP2 |
| ENSG00000111642 | -1.385 | 9.952e-33 | 1e-31 | CHD4 |
| ENSG00000111644 | 2.42 | 0.0002302 | 0.0004898 | ACRBP |
| ENSG00000111665 | -2.579 | 8.493e-99 | 5.34e-97 | CDCA3 |
| ENSG00000111666 | -1.089 | 1.349e-06 | 3.521e-06 | CHPT1 |
| ENSG00000111670 | -2.617 | 2.355e-90 | 1.251e-88 | GNPTAB |
| ENSG00000111671 | -2.282 | 8.691e-16 | 4.069e-15 | SPSB2 |
| ENSG00000111674 | -1.169 | 1.315e-09 | 4.31e-09 | ENO2 |
| ENSG00000111679 | -2.375 | 1.746e-61 | 4.454e-60 | PTPN6 |
| ENSG00000111696 | 1.536 | 1.591e-33 | 1.647e-32 | NT5DC3 |
| ENSG00000111700 | 1.09 | 9.176e-16 | 4.292e-15 | SLCO1B3 |
| ENSG00000111731 | -2.412 | 8.052e-73 | 2.781e-71 | C2CD5 |
| ENSG00000111785 | -1.767 | 8.44e-26 | 6.367e-25 | RIC8B |
| ENSG00000111816 | -2.278 | 8.425e-48 | 1.423e-46 | FRK |
| ENSG00000111837 | -1.506 | 8.24e-05 | 0.0001834 | MAK |
| ENSG00000111850 | -2.134 | 2.88e-08 | 8.56e-08 | SMIM8 |
| ENSG00000111863 | 1.528 | 3.673e-21 | 2.241e-20 | ADTRP |
| ENSG00000111877 | -1.619 | 1.814e-39 | 2.366e-38 | MCM9 |
| ENSG00000111885 | -1.069 | 4.092e-11 | 1.48e-10 | MAN1A1 |
| ENSG00000111886 | 1.907 | 1.849e-08 | 5.566e-08 | GABRR2 |
| ENSG00000111961 | -1.098 | 3.771e-16 | 1.803e-15 | SASH1 |
| ENSG00000111981 | 1.393 | 1.347e-06 | 3.516e-06 | ULBP1 |
| ENSG00000112029 | -2.882 | 9.954e-41 | 1.349e-39 | FBXO5 |
| ENSG00000112039 | -2.878 | 1.952e-32 | 1.94e-31 | FANCE |
| ENSG00000112041 | -1.766 | 0.02214 | 0.03622 | TULP1 |
| ENSG00000112053 | 1.744 | 0.01932 | 0.03193 | SLC26A8 |
| ENSG00000112078 | -1.833 | 2.22e-49 | 3.927e-48 | KCTD20 |
| ENSG00000112079 | -1.105 | 9.582e-33 | 9.637e-32 | STK38 |
| ENSG00000112096 | 1.589 | 1.042e-40 | 1.411e-39 | SOD2 |
| ENSG00000112118 | -3.387 | 2.613e-158 | 4.82e-156 | MCM3 |
| ENSG00000112130 | -1.693 | 3.076e-31 | 2.892e-30 | RNF8 |
| ENSG00000112137 | 2.226 | 0.0002179 | 0.0004651 | PHACTR1 |
| ENSG00000112139 | -1.047 | 5.512e-07 | 1.485e-06 | MDGA1 |
| ENSG00000112144 | -1.83 | 2.604e-23 | 1.745e-22 | ICK |
| ENSG00000112167 | -1.931 | 6.173e-19 | 3.387e-18 | SAYSD1 |
| ENSG00000112183 | 2.114 | 1.168e-26 | 9.097e-26 | RBM24 |
| ENSG00000112195 | 2.898 | 0.003104 | 0.005787 | TREML2 |
| ENSG00000112208 | -1.769 | 3.04e-25 | 2.232e-24 | BAG2 |
| ENSG00000112218 | -3.224 | 1.855e-06 | 4.777e-06 | GPR63 |
| ENSG00000112234 | -2.75 | 1.886e-50 | 3.423e-49 | FBXL4 |
| ENSG00000112245 | 1.525 | 1.35e-45 | 2.142e-44 | PTP4A1 |
| ENSG00000112246 | 3.547 | 0.003434 | 0.006355 | SIM1 |
| ENSG00000112276 | -1.333 | 0.009791 | 0.01699 | BVES |
| ENSG00000112293 | -1.479 | 1.749e-06 | 4.517e-06 | GPLD1 |
| ENSG00000112294 | -1.505 | 7.69e-39 | 9.853e-38 | ALDH5A1 |
| ENSG00000112297 | -2.814 | 3.156e-185 | 8.279e-183 | AIM1 |
| ENSG00000112299 | 1.707 | 0.0003838 | 0.0007975 | VNN1 |
| ENSG00000112309 | 1.134 | 5.417e-05 | 0.0001229 | B3GAT2 |
| ENSG00000112312 | -2.609 | 1.168e-89 | 6.089e-88 | GMNN |
| ENSG00000112335 | 1.22 | 1.692e-16 | 8.203e-16 | SNX3 |
| ENSG00000112343 | -1.152 | 5.639e-23 | 3.709e-22 | TRIM38 |
| ENSG00000112365 | -2.293 | 2.18e-33 | 2.245e-32 | ZBTB24 |
| ENSG00000112394 | -1.513 | 0.004451 | 0.008105 | SLC16A10 |
| ENSG00000112419 | -1.286 | 1.67e-27 | 1.346e-26 | PHACTR2 |
| ENSG00000112511 | 1.128 | 1.676e-17 | 8.56e-17 | PHF1 |
| ENSG00000112531 | 1.94 | 1.34e-89 | 6.963e-88 | QKI |
| ENSG00000112541 | 3.434 | 5.406e-06 | 1.338e-05 | PDE10A |
| ENSG00000112562 | 7.067 | 1.524e-15 | 7.04e-15 | SMOC2 |
| ENSG00000112599 | -2.046 | 0.0118 | 0.02022 | GUCA1B |
| ENSG00000112624 | -1.45 | 1.277e-12 | 5.057e-12 | GLTSCR1L |
| ENSG00000112640 | -1.288 | 6.035e-36 | 6.891e-35 | PPP2R5D |
| ENSG00000112651 | -1.191 | 5.359e-17 | 2.673e-16 | MRPL2 |
| ENSG00000112655 | -2.346 | 9.145e-53 | 1.771e-51 | PTK7 |
| ENSG00000112659 | -1.646 | 5.995e-40 | 7.917e-39 | CUL9 |
| ENSG00000112679 | -1.121 | 1.19e-13 | 5.01e-13 | DUSP22 |
| ENSG00000112685 | -1.145 | 6.15e-25 | 4.44e-24 | EXOC2 |
| ENSG00000112715 | 1.852 | 1.461e-106 | 1.076e-104 | VEGFA |
| ENSG00000112742 | -1.045 | 2.286e-12 | 8.915e-12 | TTK |
| ENSG00000112759 | -1.98 | 8.756e-65 | 2.466e-63 | SLC29A1 |
| ENSG00000112769 | -2.266 | 1.463e-72 | 5.022e-71 | LAMA4 |
| ENSG00000112796 | -3.351 | 9.825e-24 | 6.735e-23 | ENPP5 |
| ENSG00000112812 | -1.058 | 1.078e-06 | 2.84e-06 | PRSS16 |
| ENSG00000112855 | -1.663 | 9.13e-19 | 4.969e-18 | HARS2 |
| ENSG00000112874 | -1.484 | 4.628e-25 | 3.364e-24 | NUDT12 |
| ENSG00000112902 | -1.421 | 1.487e-24 | 1.057e-23 | SEMA5A |
| ENSG00000112984 | -4.801 | 1.709e-281 | 1.063e-278 | KIF20A |
| ENSG00000112992 | -1.213 | 6.336e-19 | 3.473e-18 | NNT |
| ENSG00000113013 | 1.089 | 5.69e-32 | 5.544e-31 | HSPA9 |
| ENSG00000113070 | 4.032 | 8.381e-148 | 1.279e-145 | HBEGF |
| ENSG00000113119 | -1.175 | 6.134e-09 | 1.916e-08 | TMCO6 |
| ENSG00000113194 | 1.374 | 4.284e-80 | 1.763e-78 | FAF2 |
| ENSG00000113231 | -1.391 | 0.0007665 | 0.001539 | PDE8B |
| ENSG00000113263 | 3.932 | 0.0004467 | 0.0009207 | ITK |
| ENSG00000113273 | -2.048 | 8.111e-33 | 8.177e-32 | ARSB |
| ENSG00000113356 | -1.172 | 3.511e-11 | 1.276e-10 | POLR3G |
| ENSG00000113360 | -1.628 | 5.689e-32 | 5.544e-31 | DROSHA |
| ENSG00000113368 | -3.583 | 2.156e-307 | 1.645e-304 | LMNB1 |
| ENSG00000113441 | -1.276 | 8.035e-26 | 6.072e-25 | LNPEP |
| ENSG00000113456 | -1.659 | 1.831e-32 | 1.823e-31 | RAD1 |
| ENSG00000113494 | 5.413 | 1.863e-08 | 5.606e-08 | PRLR |
| ENSG00000113555 | 1.464 | 8.734e-07 | 2.32e-06 | PCDH12 |
| ENSG00000113578 | 1.509 | 3.262e-05 | 7.536e-05 | FGF1 |
| ENSG00000113583 | -1.362 | 6.116e-38 | 7.582e-37 | C5orf15 |
| ENSG00000113600 | 3.049 | 0.02328 | 0.03793 | C9 |
| ENSG00000113615 | 2.009 | 1.833e-32 | 1.824e-31 | SEC24A |
| ENSG00000113621 | -2.278 | 2.76e-43 | 4.09e-42 | TXNDC15 |
| ENSG00000113657 | 3.622 | 1.611e-05 | 3.822e-05 | DPYSL3 |
| ENSG00000113658 | -1.542 | 2.076e-39 | 2.691e-38 | SMAD5 |
| ENSG00000113742 | 1.745 | 2.088e-86 | 1.019e-84 | CPEB4 |
| ENSG00000113758 | -1.133 | 1.838e-27 | 1.478e-26 | DBN1 |
| ENSG00000113763 | -1.114 | 1.12e-08 | 3.424e-08 | UNC5A |
| ENSG00000113790 | -3.006 | 1.218e-21 | 7.595e-21 | EHHADH |
| ENSG00000113811 | 2.141 | 3.863e-24 | 2.691e-23 | SELK |
| ENSG00000113812 | -1.411 | 6.012e-22 | 3.79e-21 | ACTR8 |
| ENSG00000113838 | -1.617 | 1.815e-16 | 8.791e-16 | TBCCD1 |
| ENSG00000113905 | 7.173 | 4.352e-44 | 6.604e-43 | HRG |
| ENSG00000113916 | 1.466 | 8.275e-19 | 4.511e-18 | BCL6 |
| ENSG00000113966 | -1.153 | 2.401e-11 | 8.825e-11 | ARL6 |
| ENSG00000114026 | -1.662 | 2.248e-25 | 1.661e-24 | OGG1 |
| ENSG00000114098 | -2.168 | 9.988e-38 | 1.23e-36 | ARMC8 |
| ENSG00000114120 | -1.398 | 8.579e-49 | 1.494e-47 | SLC25A36 |
| ENSG00000114124 | 2.964 | 0.0005091 | 0.001044 | GRK7 |
| ENSG00000114126 | -1.426 | 4.289e-16 | 2.045e-15 | TFDP2 |
| ENSG00000114279 | -1.003 | 0.003503 | 0.006475 | FGF12 |
| ENSG00000114346 | -1 | 1.11e-21 | 6.94e-21 | ECT2 |
| ENSG00000114388 | -1.216 | 1.291e-14 | 5.691e-14 | NPRL2 |
| ENSG00000114416 | 1.279 | 1.555e-72 | 5.317e-71 | FXR1 |
| ENSG00000114423 | 1.238 | 1.65e-12 | 6.484e-12 | CBLB |
| ENSG00000114439 | -1.346 | 7.938e-35 | 8.72e-34 | BBX |
| ENSG00000114450 | -1.457 | 8.083e-08 | 2.327e-07 | GNB4 |
| ENSG00000114480 | 1.1 | 8.201e-15 | 3.665e-14 | GBE1 |
| ENSG00000114491 | -2.184 | 1.933e-28 | 1.621e-27 | UMPS |
| ENSG00000114554 | -1.576 | 1.081e-27 | 8.812e-27 | PLXNA1 |
| ENSG00000114631 | -2.158 | 2.353e-56 | 5.032e-55 | PODXL2 |
| ENSG00000114648 | 1.687 | 1.946e-45 | 3.073e-44 | KLHL18 |
| ENSG00000114650 | -1.008 | 1.915e-14 | 8.375e-14 | SCAP |
| ENSG00000114698 | -1.733 | 0.02552 | 0.04129 | PLSCR4 |
| ENSG00000114735 | -2.549 | 1.465e-34 | 1.594e-33 | HEMK1 |
| ENSG00000114737 | -2.359 | 3.985e-09 | 1.26e-08 | CISH |
| ENSG00000114739 | -1.993 | 8.375e-25 | 6.003e-24 | ACVR2B |
| ENSG00000114744 | -1.042 | 3.385e-23 | 2.255e-22 | COMMD2 |
| ENSG00000114745 | -2.174 | 2.3e-42 | 3.305e-41 | GORASP1 |
| ENSG00000114757 | -1.773 | 0.006393 | 0.01141 | PEX5L |
| ENSG00000114770 | -1.082 | 2.216e-40 | 2.964e-39 | ABCC5 |
| ENSG00000114771 | 2.31 | 2.594e-30 | 2.345e-29 | AADAC |
| ENSG00000114790 | -1.589 | 2.987e-11 | 1.089e-10 | ARHGEF26 |
| ENSG00000114796 | 1.597 | 2.535e-31 | 2.401e-30 | KLHL24 |
| ENSG00000114812 | -1.931 | 2.132e-11 | 7.865e-11 | VIPR1 |
| ENSG00000114841 | 1.009 | 3.278e-13 | 1.344e-12 | DNAH1 |
| ENSG00000114853 | -2.6 | 3.167e-12 | 1.225e-11 | ZBTB47 |
| ENSG00000114859 | -1.058 | 3.595e-09 | 1.141e-08 | CLCN2 |
| ENSG00000114904 | -1.057 | 6.656e-21 | 4.007e-20 | NEK4 |
| ENSG00000114923 | -1.378 | 3.603e-10 | 1.224e-09 | SLC4A3 |
| ENSG00000114948 | -1.288 | 2.613e-05 | 6.086e-05 | ADAM23 |
| ENSG00000114993 | -1.847 | 8.179e-67 | 2.447e-65 | RTKN |
| ENSG00000114999 | -1.352 | 9.038e-32 | 8.744e-31 | TTL |
| ENSG00000115008 | 3.178 | 4.786e-16 | 2.275e-15 | IL1A |
| ENSG00000115009 | 5.036 | 2.508e-34 | 2.704e-33 | CCL20 |
| ENSG00000115073 | -1.587 | 4.237e-12 | 1.626e-11 | ACTR1B |
| ENSG00000115091 | 1.313 | 3.763e-83 | 1.684e-81 | ACTR3 |
| ENSG00000115107 | -2.495 | 2.579e-82 | 1.13e-80 | STEAP3 |
| ENSG00000115112 | -3.154 | 2.811e-45 | 4.409e-44 | TFCP2L1 |
| ENSG00000115129 | -1.749 | 1.177e-15 | 5.48e-15 | TP53I3 |
| ENSG00000115137 | 1.152 | 0.0006839 | 0.001381 | DNAJC27 |
| ENSG00000115163 | -1.902 | 5.612e-21 | 3.395e-20 | CENPA |
| ENSG00000115183 | -2.365 | 1.351e-69 | 4.304e-68 | TANC1 |
| ENSG00000115194 | -1.263 | 0.000874 | 0.001744 | SLC30A3 |
| ENSG00000115207 | -1.469 | 7.52e-26 | 5.689e-25 | GTF3C2 |
| ENSG00000115233 | 1.407 | 2.561e-52 | 4.902e-51 | PSMD14 |
| ENSG00000115234 | -1.657 | 1.109e-21 | 6.94e-21 | SNX17 |
| ENSG00000115266 | 1.614 | 5.069e-13 | 2.056e-12 | APC2 |
| ENSG00000115271 | -1.39 | 4.903e-09 | 1.542e-08 | GCA |
| ENSG00000115275 | -2.026 | 5.803e-41 | 7.958e-40 | MOGS |
| ENSG00000115282 | -1.385 | 1.063e-20 | 6.331e-20 | TTC31 |
| ENSG00000115306 | -1.358 | 7.075e-48 | 1.2e-46 | SPTBN1 |
| ENSG00000115310 | 1.637 | 1.771e-96 | 1.077e-94 | RTN4 |
| ENSG00000115318 | -1.656 | 6.247e-13 | 2.52e-12 | LOXL3 |
| ENSG00000115325 | -2.152 | 7.763e-09 | 2.408e-08 | DOK1 |
| ENSG00000115363 | -2.102 | 2.889e-12 | 1.119e-11 | EVA1A |
| ENSG00000115365 | -1.266 | 6.98e-24 | 4.816e-23 | LANCL1 |
| ENSG00000115368 | -1.701 | 9.763e-30 | 8.653e-29 | WDR75 |
| ENSG00000115392 | -1.832 | 7.107e-35 | 7.817e-34 | FANCL |
| ENSG00000115419 | 1.549 | 7.604e-34 | 8.002e-33 | GLS |
| ENSG00000115446 | -1.205 | 5.517e-12 | 2.103e-11 | UNC50 |
| ENSG00000115461 | -1.916 | 3.262e-30 | 2.933e-29 | IGFBP5 |
| ENSG00000115474 | 2.075 | 0.001538 | 0.002989 | KCNJ13 |
| ENSG00000115507 | -2.184 | 1.756e-13 | 7.331e-13 | OTX1 |
| ENSG00000115520 | 1.307 | 1.659e-33 | 1.714e-32 | COQ10B |
| ENSG00000115526 | -4.213 | 6.828e-31 | 6.329e-30 | CHST10 |
| ENSG00000115568 | -1.298 | 3.707e-20 | 2.153e-19 | ZNF142 |
| ENSG00000115592 | 3.031 | 0.02445 | 0.03966 | PRKAG3 |
| ENSG00000115594 | -2.02 | 4.493e-18 | 2.367e-17 | IL1R1 |
| ENSG00000115596 | -2.869 | 2.335e-16 | 1.127e-15 | WNT6 |
| ENSG00000115602 | 3.001 | 2.465e-199 | 8.444e-197 | IL1RL1 |
| ENSG00000115641 | 1.794 | 5.52e-67 | 1.657e-65 | FHL2 |
| ENSG00000115648 | -1.116 | 2.896e-17 | 1.463e-16 | MLPH |
| ENSG00000115687 | -2.199 | 3.092e-32 | 3.049e-31 | PASK |
| ENSG00000115738 | 4.312 | 3.94e-46 | 6.347e-45 | ID2 |
| ENSG00000115756 | 1.565 | 6.273e-34 | 6.631e-33 | HPCAL1 |
| ENSG00000115825 | -1.009 | 5.438e-24 | 3.772e-23 | PRKD3 |
| ENSG00000115827 | -1.646 | 1.825e-40 | 2.459e-39 | DCAF17 |
| ENSG00000115840 | -2.934 | 4.199e-96 | 2.545e-94 | SLC25A12 |
| ENSG00000115841 | -1.87 | 6.3e-08 | 1.826e-07 | RMDN2 |
| ENSG00000115844 | 3.843 | 3.5e-20 | 2.037e-19 | DLX2 |
| ENSG00000115884 | -3.865 | 5.831e-83 | 2.576e-81 | SDC1 |
| ENSG00000115902 | 1.262 | 1.622e-07 | 4.569e-07 | SLC1A4 |
| ENSG00000115942 | -1.941 | 7.416e-58 | 1.664e-56 | ORC2 |
| ENSG00000115963 | 2.524 | 1.624e-150 | 2.672e-148 | RND3 |
| ENSG00000115970 | -1.068 | 3.99e-13 | 1.627e-12 | THADA |
| ENSG00000115998 | 1.432 | 1.727e-12 | 6.777e-12 | C2orf42 |
| ENSG00000116005 | -1.816 | 5.78e-58 | 1.302e-56 | PCYOX1 |
| ENSG00000116017 | -1.926 | 7.318e-50 | 1.311e-48 | ARID3A |
| ENSG00000116035 | -3.4 | 0.002637 | 0.00497 | VAX2 |
| ENSG00000116044 | 2.264 | 5.076e-58 | 1.148e-56 | NFE2L2 |
| ENSG00000116062 | -2.422 | 1.9e-101 | 1.276e-99 | MSH6 |
| ENSG00000116096 | -1.817 | 5.584e-29 | 4.802e-28 | SPR |
| ENSG00000116133 | -1.804 | 2.778e-82 | 1.208e-80 | DHCR24 |
| ENSG00000116138 | -2.06 | 9.595e-29 | 8.184e-28 | DNAJC16 |
| ENSG00000116141 | -1.995 | 4.023e-07 | 1.098e-06 | MARK1 |
| ENSG00000116151 | -1.876 | 7.796e-20 | 4.459e-19 | MORN1 |
| ENSG00000116161 | 1.928 | 2.644e-110 | 2.094e-108 | CACYBP |
| ENSG00000116183 | 5.269 | 8.316e-08 | 2.392e-07 | PAPPA2 |
| ENSG00000116213 | -1.011 | 7.685e-07 | 2.049e-06 | WRAP73 |
| ENSG00000116237 | -2.52 | 8.664e-107 | 6.407e-105 | ICMT |
| ENSG00000116285 | 2.36 | 2.323e-163 | 4.587e-161 | ERRFI1 |
| ENSG00000116299 | 2.691 | 2.486e-16 | 1.198e-15 | KIAA1324 |
| ENSG00000116353 | -1.117 | 7.113e-06 | 1.743e-05 | MECR |
| ENSG00000116396 | -1.729 | 4.446e-07 | 1.209e-06 | KCNC4 |
| ENSG00000116406 | -1.069 | 1.947e-19 | 1.094e-18 | EDEM3 |
| ENSG00000116497 | -1.545 | 6.62e-33 | 6.718e-32 | S100PBP |
| ENSG00000116525 | -1.238 | 1.725e-12 | 6.771e-12 | TRIM62 |
| ENSG00000116560 | 1.023 | 3.419e-23 | 2.276e-22 | SFPQ |
| ENSG00000116604 | 1.208 | 3.83e-14 | 1.651e-13 | MEF2D |
| ENSG00000116663 | -1.398 | 3.321e-07 | 9.134e-07 | FBXO6 |
| ENSG00000116667 | -1.648 | 0.00536 | 0.009673 | C1orf21 |
| ENSG00000116668 | 2.52 | 2.952e-20 | 1.721e-19 | SWT1 |
| ENSG00000116685 | -1.992 | 5.964e-45 | 9.243e-44 | KIAA2013 |
| ENSG00000116691 | -1.918 | 2.441e-16 | 1.177e-15 | MIIP |
| ENSG00000116701 | 1.988 | 4.237e-77 | 1.628e-75 | NCF2 |
| ENSG00000116703 | 1.813 | 0.02133 | 0.03499 | PDC |
| ENSG00000116717 | 3.171 | 1.687e-198 | 5.665e-196 | GADD45A |
| ENSG00000116741 | 3.631 | 1.611e-84 | 7.514e-83 | RGS2 |
| ENSG00000116745 | 1.608 | 1.006e-08 | 3.088e-08 | RPE65 |
| ENSG00000116761 | 1.549 | 4.675e-09 | 1.472e-08 | CTH |
| ENSG00000116771 | -1.743 | 0.002283 | 0.004338 | AGMAT |
| ENSG00000116809 | 1.017 | 3.581e-17 | 1.801e-16 | ZBTB17 |
| ENSG00000116833 | -3.725 | 3.142e-27 | 2.508e-26 | NR5A2 |
| ENSG00000116857 | -1.127 | 2.91e-11 | 1.063e-10 | TMEM9 |
| ENSG00000116863 | -2.153 | 4.68e-14 | 2.011e-13 | ADPRHL2 |
| ENSG00000116885 | -1.072 | 9.775e-06 | 2.366e-05 | OSCP1 |
| ENSG00000116903 | 1.508 | 6.766e-40 | 8.929e-39 | EXOC8 |
| ENSG00000116962 | 3.669 | 4.083e-05 | 9.362e-05 | NID1 |
| ENSG00000116984 | -2.355 | 1.47e-86 | 7.237e-85 | MTR |
| ENSG00000116990 | -2.681 | 0.0007632 | 0.001533 | MYCL |
| ENSG00000117000 | 2.658 | 3.439e-100 | 2.246e-98 | RLF |
| ENSG00000117013 | -1.879 | 0.0001324 | 0.0002885 | KCNQ4 |
| ENSG00000117016 | -1.291 | 6.269e-11 | 2.242e-10 | RIMS3 |
| ENSG00000117036 | 1.656 | 2.686e-64 | 7.452e-63 | ETV3 |
| ENSG00000117114 | 1.409 | 1.337e-22 | 8.662e-22 | LPHN2 |
| ENSG00000117115 | -1.187 | 5.85e-11 | 2.096e-10 | PADI2 |
| ENSG00000117143 | 1.149 | 8.904e-46 | 1.419e-44 | UAP1 |
| ENSG00000117152 | 5.071 | 3.986e-07 | 1.089e-06 | RGS4 |
| ENSG00000117155 | -1.925 | 1.33e-76 | 5.064e-75 | SSX2IP |
| ENSG00000117222 | 1.493 | 2.314e-54 | 4.669e-53 | RBBP5 |
| ENSG00000117262 | 1.415 | 5.715e-15 | 2.568e-14 | GPR89A |
| ENSG00000117280 | -1.91 | 1.14e-19 | 6.48e-19 | RAB7L1 |
| ENSG00000117281 | -1.712 | 0.0009707 | 0.001929 | CD160 |
| ENSG00000117289 | -1.676 | 1.25e-31 | 1.202e-30 | TXNIP |
| ENSG00000117305 | -1.63 | 1.439e-15 | 6.654e-15 | HMGCL |
| ENSG00000117308 | -1.094 | 2.885e-08 | 8.573e-08 | GALE |
| ENSG00000117318 | -2.703 | 2.653e-60 | 6.558e-59 | ID3 |
| ENSG00000117385 | -1.154 | 1.273e-14 | 5.615e-14 | LEPRE1 |
| ENSG00000117399 | -2.837 | 4.382e-74 | 1.568e-72 | CDC20 |
| ENSG00000117411 | -1.467 | 1.899e-39 | 2.472e-38 | B4GALT2 |
| ENSG00000117461 | -2.262 | 2.982e-90 | 1.574e-88 | PIK3R3 |
| ENSG00000117475 | 1.107 | 1.168e-28 | 9.918e-28 | BLZF1 |
| ENSG00000117477 | 3.774 | 2.535e-08 | 7.565e-08 | CCDC181 |
| ENSG00000117481 | -2.243 | 1.365e-21 | 8.498e-21 | NSUN4 |
| ENSG00000117519 | 1.156 | 7.563e-60 | 1.84e-58 | CNN3 |
| ENSG00000117593 | -1.674 | 1.012e-42 | 1.467e-41 | DARS2 |
| ENSG00000117602 | -2.286 | 6.308e-36 | 7.199e-35 | RCAN3 |
| ENSG00000117614 | 1.612 | 3.215e-36 | 3.717e-35 | SYF2 |
| ENSG00000117620 | -1.49 | 6.661e-28 | 5.473e-27 | SLC35A3 |
| ENSG00000117625 | -1.784 | 2.772e-32 | 2.744e-31 | RCOR3 |
| ENSG00000117643 | -2.009 | 0.006939 | 0.01232 | MAN1C1 |
| ENSG00000117650 | -2.588 | 2.78e-95 | 1.66e-93 | NEK2 |
| ENSG00000117676 | -1.265 | 5.998e-29 | 5.15e-28 | RPS6KA1 |
| ENSG00000117697 | -1.374 | 2.83e-22 | 1.81e-21 | NSL1 |
| ENSG00000117724 | -3.446 | 9.057e-145 | 1.299e-142 | CENPF |
| ENSG00000117751 | -1.676 | 1.034e-37 | 1.273e-36 | PPP1R8 |
| ENSG00000117791 | -1.77 | 7.87e-18 | 4.085e-17 | MARC2 |
| ENSG00000117877 | -2.103 | 5.967e-35 | 6.589e-34 | CD3EAP |
| ENSG00000117899 | -1.172 | 2.853e-23 | 1.909e-22 | MESDC2 |
| ENSG00000118007 | -1.044 | 3.397e-21 | 2.075e-20 | STAG1 |
| ENSG00000118046 | -1.548 | 2.315e-21 | 1.425e-20 | STK11 |
| ENSG00000118058 | -1.57 | 1.936e-27 | 1.554e-26 | KMT2A |
| ENSG00000118096 | -2.436 | 2.681e-17 | 1.357e-16 | IFT46 |
| ENSG00000118160 | 3.635 | 1.399e-12 | 5.527e-12 | SLC8A2 |
| ENSG00000118194 | 5.039 | 2.345e-55 | 4.871e-54 | TNNT2 |
| ENSG00000118197 | -1.312 | 2.493e-23 | 1.673e-22 | DDX59 |
| ENSG00000118242 | 1.023 | 9.784e-08 | 2.801e-07 | MREG |
| ENSG00000118246 | -3.067 | 9.4e-105 | 6.772e-103 | FASTKD2 |
| ENSG00000118257 | -3.958 | 1.477e-105 | 1.068e-103 | NRP2 |
| ENSG00000118276 | -1.621 | 5.147e-24 | 3.574e-23 | B4GALT6 |
| ENSG00000118407 | 3.03 | 1.412e-10 | 4.931e-10 | FILIP1 |
| ENSG00000118432 | 5.083 | 3.576e-07 | 9.806e-07 | CNR1 |
| ENSG00000118496 | 2.744 | 1.143e-124 | 1.238e-122 | FBXO30 |
| ENSG00000118503 | 3.344 | 3.491e-152 | 5.92e-150 | TNFAIP3 |
| ENSG00000118507 | -1.25 | 0.001023 | 0.002029 | AKAP7 |
| ENSG00000118513 | -4.639 | 2.164e-20 | 1.274e-19 | MYB |
| ENSG00000118515 | 2.707 | 9.008e-121 | 9.164e-119 | SGK1 |
| ENSG00000118523 | 1.061 | 1.165e-21 | 7.275e-21 | CTGF |
| ENSG00000118557 | 1.381 | 0.009262 | 0.01613 | PMFBP1 |
| ENSG00000118564 | -1.186 | 1.009e-14 | 4.485e-14 | FBXL5 |
| ENSG00000118600 | -1.588 | 1.194e-28 | 1.013e-27 | TMEM5 |
| ENSG00000118620 | 2.125 | 1.385e-14 | 6.101e-14 | ZNF430 |
| ENSG00000118655 | -2.528 | 2.62e-53 | 5.15e-52 | DCLRE1B |
| ENSG00000118690 | -1.186 | 0.001128 | 0.002225 | ARMC2 |
| ENSG00000118707 | -1.477 | 1.312e-20 | 7.778e-20 | TGIF2 |
| ENSG00000118762 | -1.859 | 5.144e-25 | 3.73e-24 | PKD2 |
| ENSG00000118777 | -1.015 | 3.363e-29 | 2.921e-28 | ABCG2 |
| ENSG00000118804 | -2.084 | 0.0001118 | 0.0002461 | FAM47E-STBD1 |
| ENSG00000118894 | -2.946 | 2.702e-42 | 3.873e-41 | FAM86A |
| ENSG00000118898 | -1.89 | 2.303e-114 | 1.963e-112 | PPL |
| ENSG00000118900 | -1.038 | 2.977e-23 | 1.991e-22 | UBN1 |
| ENSG00000118960 | -1.715 | 6.242e-14 | 2.664e-13 | HS1BP3 |
| ENSG00000118965 | -1.485 | 3.961e-16 | 1.891e-15 | WDR35 |
| ENSG00000118985 | 2.684 | 8.919e-77 | 3.403e-75 | ELL2 |
| ENSG00000118997 | -1.714 | 0.0007833 | 0.001571 | DNAH7 |
| ENSG00000119004 | -1.812 | 2.384e-18 | 1.274e-17 | CYP20A1 |
| ENSG00000119048 | 1.553 | 1.383e-64 | 3.882e-63 | UBE2B |
| ENSG00000119203 | -1.18 | 2.374e-34 | 2.569e-33 | CPSF3 |
| ENSG00000119227 | -1.215 | 0.007294 | 0.01291 | PIGZ |
| ENSG00000119280 | -1.038 | 4.585e-16 | 2.182e-15 | C1orf198 |
| ENSG00000119285 | -1.075 | 4.858e-46 | 7.789e-45 | HEATR1 |
| ENSG00000119333 | -1.002 | 4.956e-07 | 1.342e-06 | WDR34 |
| ENSG00000119383 | -1.062 | 1.729e-07 | 4.854e-07 | PPP2R4 |
| ENSG00000119401 | -1.342 | 7.317e-29 | 6.273e-28 | TRIM32 |
| ENSG00000119402 | -1.226 | 2.162e-31 | 2.056e-30 | FBXW2 |
| ENSG00000119403 | -2.064 | 2.423e-46 | 3.929e-45 | PHF19 |
| ENSG00000119408 | -2.209 | 1.243e-50 | 2.275e-49 | NEK6 |
| ENSG00000119411 | -1.127 | 2.161e-12 | 8.436e-12 | BSPRY |
| ENSG00000119414 | 1.137 | 2.491e-31 | 2.361e-30 | PPP6C |
| ENSG00000119431 | -2.526 | 5.948e-22 | 3.751e-21 | HDHD3 |
| ENSG00000119508 | 3.485 | 3.316e-16 | 1.59e-15 | NR4A3 |
| ENSG00000119514 | -1.428 | 3.467e-34 | 3.717e-33 | GALNT12 |
| ENSG00000119522 | -1.048 | 3.654e-14 | 1.576e-13 | DENND1A |
| ENSG00000119541 | 1.645 | 9.859e-64 | 2.678e-62 | VPS4B |
| ENSG00000119547 | -2.819 | 2.753e-54 | 5.548e-53 | ONECUT2 |
| ENSG00000119574 | -2.704 | 1.574e-41 | 2.206e-40 | ZBTB45 |
| ENSG00000119596 | -1.522 | 7.36e-14 | 3.13e-13 | YLPM1 |
| ENSG00000119608 | -2.206 | 5.052e-20 | 2.916e-19 | PROX2 |
| ENSG00000119630 | 3.809 | 6.954e-25 | 5.01e-24 | PGF |
| ENSG00000119638 | -1.042 | 5.699e-17 | 2.835e-16 | NEK9 |
| ENSG00000119661 | -1.172 | 2.291e-10 | 7.899e-10 | DNAL1 |
| ENSG00000119669 | -4.266 | 2.42e-147 | 3.628e-145 | IRF2BPL |
| ENSG00000119673 | -2.469 | 3.087e-25 | 2.263e-24 | ACOT2 |
| ENSG00000119684 | -1.599 | 4.104e-61 | 1.036e-59 | MLH3 |
| ENSG00000119685 | -1.017 | 1.964e-08 | 5.903e-08 | TTLL5 |
| ENSG00000119686 | -1.546 | 5.41e-09 | 1.696e-08 | FLVCR2 |
| ENSG00000119688 | -1.181 | 2.867e-12 | 1.112e-11 | ABCD4 |
| ENSG00000119699 | -1.568 | 0.003233 | 0.006009 | TGFB3 |
| ENSG00000119703 | -1.331 | 1.846e-10 | 6.392e-10 | ZC2HC1C |
| ENSG00000119707 | 1.123 | 2.427e-15 | 1.111e-14 | RBM25 |
| ENSG00000119715 | 1.749 | 0.02661 | 0.04293 | ESRRB |
| ENSG00000119737 | 1.907 | 8.046e-10 | 2.675e-09 | GPR75 |
| ENSG00000119772 | -1.162 | 3.783e-29 | 3.283e-28 | DNMT3A |
| ENSG00000119787 | -1.516 | 4.822e-47 | 8.03e-46 | ATL2 |
| ENSG00000119801 | 2.032 | 9.495e-63 | 2.53e-61 | YPEL5 |
| ENSG00000119865 | 3.789 | 6.46e-09 | 2.014e-08 | CNRIP1 |
| ENSG00000119900 | -1.993 | 3.304e-37 | 3.978e-36 | OGFRL1 |
| ENSG00000119906 | -3.063 | 5.487e-153 | 9.399e-151 | FAM178A |
| ENSG00000119915 | -2.928 | 5.508e-10 | 1.848e-09 | ELOVL3 |
| ENSG00000119917 | -2.252 | 1.294e-08 | 3.936e-08 | IFIT3 |
| ENSG00000119922 | -2.875 | 1.839e-26 | 1.422e-25 | IFIT2 |
| ENSG00000119927 | -2.025 | 1.423e-34 | 1.55e-33 | GPAM |
| ENSG00000119938 | 4.791 | 6.307e-27 | 4.966e-26 | PPP1R3C |
| ENSG00000119946 | -3.158 | 1.055e-24 | 7.541e-24 | CNNM1 |
| ENSG00000119953 | 1.405 | 3.084e-58 | 7.023e-57 | SMNDC1 |
| ENSG00000119969 | -1.187 | 9.031e-17 | 4.447e-16 | HELLS |
| ENSG00000119977 | -1.816 | 9.759e-38 | 1.204e-36 | TCTN3 |
| ENSG00000119979 | -1.869 | 5.871e-47 | 9.737e-46 | FAM45A |
| ENSG00000120049 | -1.764 | 0.0005437 | 0.001111 | KCNIP2 |
| ENSG00000120051 | 2.876 | 1.823e-05 | 4.305e-05 | CCDC147 |
| ENSG00000120055 | -1.66 | 0.0001597 | 0.0003449 | C10orf95 |
| ENSG00000120071 | 1.275 | 2.84e-28 | 2.36e-27 | KANSL1 |
| ENSG00000120075 | -3.633 | 2.929e-19 | 1.631e-18 | HOXB5 |
| ENSG00000120088 | 3.174 | 0.009059 | 0.0158 | CRHR1 |
| ENSG00000120093 | -4.073 | 1.036e-34 | 1.137e-33 | HOXB3 |
| ENSG00000120129 | 5.477 | 0 | 0 | DUSP1 |
| ENSG00000120162 | -2.652 | 3.027e-29 | 2.632e-28 | MOB3B |
| ENSG00000120211 | 2.702 | 6.014e-13 | 2.428e-12 | INSL4 |
| ENSG00000120215 | 1.506 | 0.01891 | 0.0313 | MLANA |
| ENSG00000120217 | 1.552 | 1.006e-17 | 5.204e-17 | CD274 |
| ENSG00000120253 | -1.477 | 2.148e-45 | 3.382e-44 | NUP43 |
| ENSG00000120256 | -1.386 | 1.468e-38 | 1.856e-37 | LRP11 |
| ENSG00000120278 | -1.903 | 2.881e-14 | 1.251e-13 | PLEKHG1 |
| ENSG00000120279 | 6.372 | 4.717e-12 | 1.806e-11 | MYCT1 |
| ENSG00000120314 | -1.086 | 1.459e-28 | 1.232e-27 | WDR55 |
| ENSG00000120318 | -2.154 | 4.59e-38 | 5.72e-37 | ARAP3 |
| ENSG00000120337 | 4.956 | 3.552e-14 | 1.533e-13 | TNFSF18 |
| ENSG00000120438 | 1.242 | 6.485e-41 | 8.85e-40 | TCP1 |
| ENSG00000120471 | 2.776 | 0.002903 | 0.00543 | TP53AIP1 |
| ENSG00000120549 | 2.472 | 7.569e-37 | 8.967e-36 | KIAA1217 |
| ENSG00000120586 | 3.555 | 3.011e-16 | 1.446e-15 | MRC1 |
| ENSG00000120645 | 4.761 | 3.292e-06 | 8.303e-06 | IQSEC3 |
| ENSG00000120647 | 1.943 | 1.13e-58 | 2.607e-57 | CCDC77 |
| ENSG00000120658 | -1.039 | 5.449e-06 | 1.348e-05 | ENOX1 |
| ENSG00000120662 | 1.249 | 4.994e-26 | 3.809e-25 | MTRF1 |
| ENSG00000120686 | 2.02 | 1.719e-107 | 1.294e-105 | UFM1 |
| ENSG00000120688 | -2.291 | 1.099e-41 | 1.547e-40 | WBP4 |
| ENSG00000120690 | -1.616 | 1.117e-55 | 2.338e-54 | ELF1 |
| ENSG00000120693 | -2.988 | 2.707e-06 | 6.86e-06 | SMAD9 |
| ENSG00000120694 | 4.078 | 0 | 0 | HSPH1 |
| ENSG00000120696 | -3.109 | 4.468e-33 | 4.562e-32 | KBTBD7 |
| ENSG00000120705 | 1.437 | 1.696e-57 | 3.781e-56 | ETF1 |
| ENSG00000120733 | -1.296 | 1.195e-34 | 1.306e-33 | KDM3B |
| ENSG00000120738 | 1.388 | 2.852e-08 | 8.48e-08 | EGR1 |
| ENSG00000120742 | 1.042 | 3.49e-25 | 2.548e-24 | SERP1 |
| ENSG00000120784 | -2.754 | 4.842e-05 | 0.0001103 | ZFP30 |
| ENSG00000120802 | -2.854 | 2.654e-252 | 1.437e-249 | TMPO |
| ENSG00000120805 | -1.006 | 3.15e-16 | 1.512e-15 | ARL1 |
| ENSG00000120832 | -2.419 | 8.051e-25 | 5.781e-24 | MTERFD3 |
| ENSG00000120833 | -1.295 | 7.261e-17 | 3.593e-16 | SOCS2 |
| ENSG00000120868 | -2.276 | 2.343e-29 | 2.048e-28 | APAF1 |
| ENSG00000120875 | -2.358 | 2.504e-187 | 6.78e-185 | DUSP4 |
| ENSG00000120885 | 1.515 | 1.66e-28 | 1.398e-27 | CLU |
| ENSG00000120889 | 1.031 | 1.713e-13 | 7.158e-13 | TNFRSF10B |
| ENSG00000120896 | -1.343 | 3.154e-17 | 1.59e-16 | SORBS3 |
| ENSG00000120903 | 3.149 | 0.01045 | 0.01805 | CHRNA2 |
| ENSG00000120913 | -1.053 | 4.983e-08 | 1.455e-07 | PDLIM2 |
| ENSG00000120925 | -1.974 | 7.689e-21 | 4.608e-20 | RNF170 |
| ENSG00000120937 | 3.093 | 0.01211 | 0.02071 | NPPB |
| ENSG00000120942 | -1.468 | 3.705e-19 | 2.054e-18 | UBIAD1 |
| ENSG00000120949 | 2.791 | 0.0002693 | 0.0005688 | TNFRSF8 |
| ENSG00000121057 | -3.235 | 5.178e-188 | 1.449e-185 | AKAP1 |
| ENSG00000121058 | -1.089 | 1.74e-20 | 1.026e-19 | COIL |
| ENSG00000121068 | -1.62 | 0.0001264 | 0.0002761 | TBX2 |
| ENSG00000121075 | 2.962 | 0.02073 | 0.03408 | TBX4 |
| ENSG00000121152 | -2.666 | 2.526e-129 | 2.945e-127 | NCAPH |
| ENSG00000121210 | -2.073 | 7.719e-34 | 8.113e-33 | KIAA0922 |
| ENSG00000121211 | -1.412 | 2.414e-08 | 7.212e-08 | MND1 |
| ENSG00000121236 | -2.802 | 3.005e-26 | 2.306e-25 | TRIM6 |
| ENSG00000121270 | 2.803 | 1.37e-13 | 5.75e-13 | ABCC11 |
| ENSG00000121289 | -1.027 | 1.463e-12 | 5.772e-12 | CEP89 |
| ENSG00000121310 | -2.046 | 4.513e-27 | 3.577e-26 | ECHDC2 |
| ENSG00000121318 | -1.443 | 0.002055 | 0.003934 | TAS2R10 |
| ENSG00000121350 | 1.418 | 5.544e-42 | 7.88e-41 | PYROXD1 |
| ENSG00000121413 | -1.433 | 0.0007696 | 0.001545 | ZSCAN18 |
| ENSG00000121440 | 2.987 | 0.02765 | 0.04444 | PDZRN3 |
| ENSG00000121454 | -1.445 | 2.196e-22 | 1.411e-21 | LHX4 |
| ENSG00000121486 | -1.5 | 9.968e-18 | 5.159e-17 | TRMT1L |
| ENSG00000121542 | -1.746 | 1.645e-31 | 1.574e-30 | SEC22A |
| ENSG00000121578 | -1.796 | 6.489e-51 | 1.194e-49 | B4GALT4 |
| ENSG00000121621 | -1.634 | 3.812e-23 | 2.532e-22 | KIF18A |
| ENSG00000121671 | 1.388 | 4.824e-14 | 2.071e-13 | CRY2 |
| ENSG00000121690 | -1.949 | 2.449e-06 | 6.234e-06 | DEPDC7 |
| ENSG00000121741 | -1.096 | 4.956e-29 | 4.273e-28 | ZMYM2 |
| ENSG00000121749 | 1.645 | 4.313e-46 | 6.941e-45 | TBC1D15 |
| ENSG00000121753 | -2.039 | 2.966e-32 | 2.932e-31 | BAI2 |
| ENSG00000121764 | 3.78 | 1.717e-09 | 5.575e-09 | HCRTR1 |
| ENSG00000121775 | -1.814 | 3.37e-27 | 2.685e-26 | TMEM39B |
| ENSG00000121851 | -1.477 | 5.959e-09 | 1.863e-08 | POLR3GL |
| ENSG00000121853 | 3.303 | 0.01299 | 0.02211 | GHSR |
| ENSG00000121864 | -1.378 | 2.56e-18 | 1.366e-17 | ZNF639 |
| ENSG00000121931 | 1.466 | 2.526e-32 | 2.505e-31 | LRIF1 |
| ENSG00000121957 | -2.51 | 7.219e-109 | 5.584e-107 | GPSM2 |
| ENSG00000122033 | -1.218 | 2.963e-12 | 1.147e-11 | MTIF3 |
| ENSG00000122035 | -1.329 | 4.3e-05 | 9.838e-05 | RASL11A |
| ENSG00000122042 | 2.319 | 7.202e-100 | 4.668e-98 | UBL3 |
| ENSG00000122122 | 3.422 | 5.692e-05 | 0.0001289 | SASH3 |
| ENSG00000122126 | -1.18 | 6.177e-34 | 6.537e-33 | OCRL |
| ENSG00000122140 | -1.849 | 1.47e-33 | 1.525e-32 | MRPS2 |
| ENSG00000122218 | 1.339 | 6.962e-72 | 2.337e-70 | COPA |
| ENSG00000122223 | 2.603 | 0.02059 | 0.03387 | CD244 |
| ENSG00000122386 | -1.095 | 3.799e-07 | 1.04e-06 | ZNF205 |
| ENSG00000122417 | -1.678 | 1.882e-27 | 1.511e-26 | ODF2L |
| ENSG00000122481 | -1.536 | 4.488e-13 | 1.827e-12 | RWDD3 |
| ENSG00000122484 | -1.52 | 6.254e-14 | 2.668e-13 | RPAP2 |
| ENSG00000122512 | -1.357 | 8.51e-30 | 7.546e-29 | PMS2 |
| ENSG00000122547 | -3.178 | 5.121e-32 | 5.007e-31 | EEPD1 |
| ENSG00000122557 | -2.444 | 7.231e-52 | 1.367e-50 | HERPUD2 |
| ENSG00000122565 | -1.006 | 1.713e-11 | 6.347e-11 | CBX3 |
| ENSG00000122591 | -1.094 | 9.024e-23 | 5.894e-22 | FAM126A |
| ENSG00000122641 | 1.934 | 2.699e-134 | 3.331e-132 | INHBA |
| ENSG00000122644 | -1.232 | 3.165e-10 | 1.081e-09 | ARL4A |
| ENSG00000122678 | -1.623 | 1.141e-26 | 8.891e-26 | POLM |
| ENSG00000122687 | -1.177 | 1.65e-16 | 8.007e-16 | FTSJ2 |
| ENSG00000122694 | -1.272 | 4.775e-05 | 0.0001088 | GLIPR2 |
| ENSG00000122711 | 2.567 | 0.0279 | 0.04481 | SPINK4 |
| ENSG00000122733 | 7.814 | 3.423e-16 | 1.641e-15 | KIAA1045 |
| ENSG00000122741 | -1.155 | 3.403e-22 | 2.172e-21 | DCAF10 |
| ENSG00000122778 | -1.654 | 1.326e-27 | 1.073e-26 | KIAA1549 |
| ENSG00000122779 | -1.396 | 2.551e-09 | 8.183e-09 | TRIM24 |
| ENSG00000122783 | -1.861 | 8.692e-47 | 1.436e-45 | C7orf49 |
| ENSG00000122786 | 1.093 | 1.543e-12 | 6.084e-12 | CALD1 |
| ENSG00000122787 | 1.332 | 0.019 | 0.03144 | AKR1D1 |
| ENSG00000122862 | 1.209 | 1.507e-46 | 2.463e-45 | SRGN |
| ENSG00000122863 | -2.64 | 5.646e-62 | 1.465e-60 | CHST3 |
| ENSG00000122877 | 2.906 | 5.324e-08 | 1.552e-07 | EGR2 |
| ENSG00000122952 | -1.135 | 1.938e-37 | 2.354e-36 | ZWINT |
| ENSG00000122958 | 1.053 | 1.818e-35 | 2.052e-34 | VPS26A |
| ENSG00000122966 | -2.826 | 3.5e-91 | 1.902e-89 | CIT |
| ENSG00000122970 | -1.289 | 1.787e-10 | 6.193e-10 | IFT81 |
| ENSG00000122971 | -1.555 | 1.806e-08 | 5.44e-08 | ACADS |
| ENSG00000123064 | -1.142 | 9.738e-31 | 8.971e-30 | DDX54 |
| ENSG00000123080 | -3.69 | 4.036e-45 | 6.309e-44 | CDKN2C |
| ENSG00000123095 | -3.465 | 6.371e-116 | 5.599e-114 | BHLHE41 |
| ENSG00000123146 | -2.314 | 1.098e-83 | 4.997e-82 | CD97 |
| ENSG00000123154 | 1.238 | 1.622e-12 | 6.383e-12 | WDR83 |
| ENSG00000123219 | -1.06 | 1.426e-20 | 8.438e-20 | CENPK |
| ENSG00000123297 | -2.484 | 5.036e-21 | 3.053e-20 | TSFM |
| ENSG00000123329 | 2.755 | 0.02869 | 0.04598 | ARHGAP9 |
| ENSG00000123342 | 2.402 | 3.436e-08 | 1.016e-07 | MMP19 |
| ENSG00000123358 | 6.017 | 0 | 0 | NR4A1 |
| ENSG00000123364 | -2.885 | 6.213e-85 | 2.93e-83 | HOXC13 |
| ENSG00000123374 | -2.384 | 3.79e-67 | 1.14e-65 | CDK2 |
| ENSG00000123388 | -3.028 | 2.809e-34 | 3.024e-33 | HOXC11 |
| ENSG00000123405 | -1.375 | 0.01136 | 0.01952 | NFE2 |
| ENSG00000123411 | -1.468 | 1.521e-06 | 3.947e-06 | IKZF4 |
| ENSG00000123427 | -2.844 | 4.692e-23 | 3.096e-22 | METTL21B |
| ENSG00000123444 | 1.71 | 5.725e-12 | 2.18e-11 | KBTBD4 |
| ENSG00000123472 | -1.032 | 9.425e-16 | 4.406e-15 | ATPAF1 |
| ENSG00000123473 | -1.833 | 7.213e-44 | 1.088e-42 | STIL |
| ENSG00000123485 | -3.018 | 2.376e-168 | 4.923e-166 | HJURP |
| ENSG00000123496 | 3.325 | 5.76e-07 | 1.55e-06 | IL13RA2 |
| ENSG00000123505 | -1.221 | 1.861e-46 | 3.028e-45 | AMD1 |
| ENSG00000123545 | -1.648 | 3.834e-27 | 3.046e-26 | NDUFAF4 |
| ENSG00000123560 | 3.118 | 0.01628 | 0.02727 | PLP1 |
| ENSG00000123570 | 2.401 | 0.0009572 | 0.001903 | RAB9B |
| ENSG00000123572 | -1.228 | 7.062e-07 | 1.888e-06 | NRK |
| ENSG00000123575 | -1.849 | 5.748e-43 | 8.383e-42 | FAM199X |
| ENSG00000123600 | -2.52 | 1.392e-80 | 5.829e-79 | METTL8 |
| ENSG00000123610 | 3.109 | 0.01368 | 0.02321 | TNFAIP6 |
| ENSG00000123810 | -1.473 | 0.000733 | 0.001474 | B9D2 |
| ENSG00000123843 | -1.409 | 0.01651 | 0.02761 | C4BPB |
| ENSG00000123892 | -1.561 | 6.275e-12 | 2.382e-11 | RAB38 |
| ENSG00000123908 | 1.909 | 7.117e-97 | 4.36e-95 | AGO2 |
| ENSG00000123989 | -2.103 | 2.297e-80 | 9.544e-79 | CHPF |
| ENSG00000124006 | -2.841 | 7.147e-33 | 7.235e-32 | OBSL1 |
| ENSG00000124091 | 4.609 | 2.286e-52 | 4.391e-51 | GCNT7 |
| ENSG00000124092 | 3.176 | 0.009296 | 0.01619 | CTCFL |
| ENSG00000124102 | 1.425 | 3.197e-49 | 5.637e-48 | PI3 |
| ENSG00000124103 | 2.681 | 1.037e-06 | 2.736e-06 | FAM209A |
| ENSG00000124104 | -2.414 | 4.969e-22 | 3.144e-21 | SNX21 |
| ENSG00000124160 | -1.182 | 1.802e-35 | 2.035e-34 | NCOA5 |
| ENSG00000124171 | 3.008 | 2.764e-101 | 1.848e-99 | PARD6B |
| ENSG00000124181 | -1.35 | 2.232e-27 | 1.79e-26 | PLCG1 |
| ENSG00000124194 | 2.882 | 4.685e-05 | 0.0001069 | GDAP1L1 |
| ENSG00000124201 | -1.056 | 1.757e-35 | 1.986e-34 | ZNFX1 |
| ENSG00000124208 | 9.877 | 1.718e-26 | 1.331e-25 | TMEM189-UBE2V1 |
| ENSG00000124209 | 1.003 | 4.969e-34 | 5.269e-33 | RAB22A |
| ENSG00000124212 | -3.234 | 2.193e-30 | 1.988e-29 | PTGIS |
| ENSG00000124215 | -2.528 | 2.593e-05 | 6.043e-05 | CDH26 |
| ENSG00000124216 | 3.59 | 1.095e-66 | 3.248e-65 | SNAI1 |
| ENSG00000124224 | 2.197 | 9.081e-61 | 2.275e-59 | PPP4R1L |
| ENSG00000124233 | 3.681 | 6.465e-05 | 0.0001455 | SEMG1 |
| ENSG00000124253 | 4.425 | 2.657e-05 | 6.181e-05 | PCK1 |
| ENSG00000124256 | 5.145 | 3.031e-07 | 8.362e-07 | ZBP1 |
| ENSG00000124279 | -1.275 | 7.364e-28 | 6.038e-27 | FASTKD3 |
| ENSG00000124313 | -1.931 | 7.908e-21 | 4.736e-20 | IQSEC2 |
| ENSG00000124370 | -2.072 | 1.964e-12 | 7.687e-12 | MCEE |
| ENSG00000124391 | 4.001 | 0.0007723 | 0.001551 | IL17C |
| ENSG00000124422 | -1.77 | 1.631e-79 | 6.551e-78 | USP22 |
| ENSG00000124466 | 1.68 | 5.514e-37 | 6.569e-36 | LYPD3 |
| ENSG00000124496 | -2.569 | 2.409e-73 | 8.408e-72 | TRERF1 |
| ENSG00000124523 | -1.39 | 8.253e-19 | 4.501e-18 | SIRT5 |
| ENSG00000124532 | -1.153 | 6.195e-28 | 5.095e-27 | MRS2 |
| ENSG00000124541 | -1.131 | 1.443e-24 | 1.026e-23 | RRP36 |
| ENSG00000124574 | -1.822 | 1.417e-34 | 1.544e-33 | ABCC10 |
| ENSG00000124593 | -1.777 | 0.006772 | 0.01205 | PRICKLE4 |
| ENSG00000124608 | -3.176 | 8.896e-42 | 1.256e-40 | AARS2 |
| ENSG00000124641 | -1.347 | 3.297e-14 | 1.425e-13 | MED20 |
| ENSG00000124659 | -1.041 | 2.894e-09 | 9.247e-09 | TBCC |
| ENSG00000124702 | -1.746 | 2.106e-25 | 1.562e-24 | KLHDC3 |
| ENSG00000124713 | 2.02 | 0.01852 | 0.03072 | GNMT |
| ENSG00000124731 | 1.686 | 2.389e-06 | 6.086e-06 | TREM1 |
| ENSG00000124762 | 4.387 | 1.676e-174 | 3.801e-172 | CDKN1A |
| ENSG00000124772 | 3.764 | 1.699e-08 | 5.13e-08 | CPNE5 |
| ENSG00000124782 | -1.278 | 2.309e-29 | 2.02e-28 | RREB1 |
| ENSG00000124788 | 2.674 | 5.269e-168 | 1.079e-165 | ATXN1 |
| ENSG00000124813 | -1.982 | 2.087e-19 | 1.17e-18 | RUNX2 |
| ENSG00000124831 | 2.036 | 3.374e-63 | 9.017e-62 | LRRFIP1 |
| ENSG00000124882 | 2.334 | 3.868e-64 | 1.07e-62 | EREG |
| ENSG00000124942 | -1.484 | 2.764e-16 | 1.33e-15 | AHNAK |
| ENSG00000125046 | 2.563 | 2.572e-27 | 2.059e-26 | SSUH2 |
| ENSG00000125124 | -1.001 | 1.136e-12 | 4.516e-12 | BBS2 |
| ENSG00000125148 | 3.195 | 1.023e-84 | 4.783e-83 | MT2A |
| ENSG00000125247 | -1.702 | 2.545e-17 | 1.289e-16 | TMTC4 |
| ENSG00000125347 | -1.296 | 1.786e-06 | 4.607e-06 | IRF1 |
| ENSG00000125352 | -1.053 | 6.66e-15 | 2.986e-14 | RNF113A |
| ENSG00000125355 | 1.324 | 0.01997 | 0.03294 | TMEM255A |
| ENSG00000125378 | -1.861 | 9.674e-17 | 4.749e-16 | BMP4 |
| ENSG00000125430 | 4.217 | 1.264e-06 | 3.307e-06 | HS3ST3B1 |
| ENSG00000125434 | -1.63 | 1.641e-09 | 5.343e-09 | SLC25A35 |
| ENSG00000125450 | -1.105 | 5.045e-12 | 1.928e-11 | NUP85 |
| ENSG00000125454 | -2.539 | 4.32e-35 | 4.79e-34 | SLC25A19 |
| ENSG00000125458 | -1.302 | 4.117e-13 | 1.678e-12 | NT5C |
| ENSG00000125482 | 1.139 | 5.293e-38 | 6.587e-37 | TTF1 |
| ENSG00000125508 | -1.686 | 2.65e-05 | 6.168e-05 | SRMS |
| ENSG00000125510 | 3.069 | 0.0005848 | 0.001192 | OPRL1 |
| ENSG00000125538 | 2.837 | 1.783e-28 | 1.498e-27 | IL1B |
| ENSG00000125551 | 2.715 | 1.579e-22 | 1.021e-21 | PLGLB2 |
| ENSG00000125618 | 1.268 | 1.496e-26 | 1.163e-25 | PAX8 |
| ENSG00000125630 | -1.158 | 1.603e-36 | 1.864e-35 | POLR1B |
| ENSG00000125637 | -2.255 | 7.122e-33 | 7.214e-32 | PSD4 |
| ENSG00000125657 | 2.107 | 1.201e-25 | 9.011e-25 | TNFSF9 |
| ENSG00000125703 | -1.292 | 5.129e-14 | 2.198e-13 | ATG4C |
| ENSG00000125726 | 4.1 | 2.193e-05 | 5.145e-05 | CD70 |
| ENSG00000125735 | 5.695 | 5.209e-29 | 4.484e-28 | TNFSF14 |
| ENSG00000125740 | 7.437 | 1.579e-296 | 1.104e-293 | FOSB |
| ENSG00000125775 | 1.63 | 5.689e-10 | 1.907e-09 | SDCBP2 |
| ENSG00000125817 | -3.194 | 1.242e-199 | 4.369e-197 | CENPB |
| ENSG00000125827 | -1.1 | 1.388e-08 | 4.211e-08 | TMX4 |
| ENSG00000125841 | -1.342 | 0.0001797 | 0.0003864 | NRSN2 |
| ENSG00000125843 | -2.833 | 7.684e-51 | 1.413e-49 | AP5S1 |
| ENSG00000125845 | 1.892 | 9.093e-14 | 3.854e-13 | BMP2 |
| ENSG00000125846 | 1.606 | 2.473e-23 | 1.661e-22 | ZNF133 |
| ENSG00000125848 | -1.192 | 0.029 | 0.04641 | FLRT3 |
| ENSG00000125871 | -2.087 | 4.517e-64 | 1.243e-62 | MGME1 |
| ENSG00000125895 | 1.434 | 0.006499 | 0.01158 | TMEM74B |
| ENSG00000125898 | -1.422 | 2.741e-23 | 1.836e-22 | FAM110A |
| ENSG00000125901 | -3.842 | 2.351e-144 | 3.288e-142 | MRPS26 |
| ENSG00000125945 | 2.494 | 1.432e-111 | 1.15e-109 | ZNF436 |
| ENSG00000125954 | 3.657 | 0.005599 | 0.01007 | CHURC1-FNTB |
| ENSG00000125966 | 2.86 | 2.649e-18 | 1.411e-17 | MMP24 |
| ENSG00000125998 | 2.778 | 0.03074 | 0.049 | FAM83C |
| ENSG00000126001 | -1.845 | 9.211e-48 | 1.554e-46 | CEP250 |
| ENSG00000126003 | -1.096 | 2.941e-18 | 1.564e-17 | PLAGL2 |
| ENSG00000126016 | -2.419 | 7.958e-62 | 2.049e-60 | AMOT |
| ENSG00000126062 | -1.067 | 8.643e-13 | 3.459e-12 | TMEM115 |
| ENSG00000126106 | -1.065 | 0.0005631 | 0.001149 | TMEM53 |
| ENSG00000126215 | -1.165 | 1.33e-37 | 1.626e-36 | XRCC3 |
| ENSG00000126216 | -2.074 | 7.583e-100 | 4.896e-98 | TUBGCP3 |
| ENSG00000126218 | 2.033 | 0.003644 | 0.006724 | F10 |
| ENSG00000126243 | -2.938 | 1.294e-19 | 7.334e-19 | LRFN3 |
| ENSG00000126249 | -1.256 | 3.115e-08 | 9.234e-08 | PDCD2L |
| ENSG00000126351 | -1.267 | 9.866e-06 | 2.387e-05 | THRA |
| ENSG00000126391 | -1.158 | 0.02487 | 0.0403 | FRMD8 |
| ENSG00000126464 | -1.966 | 3.235e-23 | 2.156e-22 | PRR12 |
| ENSG00000126500 | 1.534 | 1.236e-08 | 3.767e-08 | FLRT1 |
| ENSG00000126524 | 1.258 | 1.77e-27 | 1.425e-26 | SBDS |
| ENSG00000126698 | 1.144 | 8.188e-14 | 3.474e-13 | DNAJC8 |
| ENSG00000126705 | -1.271 | 6.463e-20 | 3.713e-19 | AHDC1 |
| ENSG00000126746 | 1.121 | 1.042e-10 | 3.671e-10 | ZNF384 |
| ENSG00000126752 | 3.615 | 0.001979 | 0.003797 | SSX1 |
| ENSG00000126759 | 5.033 | 2.966e-33 | 3.045e-32 | CFP |
| ENSG00000126773 | -1.828 | 3.579e-62 | 9.372e-61 | PCNXL4 |
| ENSG00000126778 | -4.374 | 1.43e-46 | 2.339e-45 | SIX1 |
| ENSG00000126787 | -2.797 | 4.001e-163 | 7.808e-161 | DLGAP5 |
| ENSG00000126790 | -1.114 | 1.134e-17 | 5.846e-17 | L3HYPDH |
| ENSG00000126803 | -1.59 | 5.169e-18 | 2.715e-17 | HSPA2 |
| ENSG00000126804 | -1.233 | 2.275e-29 | 1.991e-28 | ZBTB1 |
| ENSG00000126814 | -2.21 | 1.387e-69 | 4.409e-68 | TRMT5 |
| ENSG00000126821 | -1.377 | 1.491e-18 | 8.054e-18 | SGPP1 |
| ENSG00000126822 | -2.439 | 9.349e-80 | 3.809e-78 | PLEKHG3 |
| ENSG00000126856 | 4.327 | 9.78e-09 | 3.005e-08 | PRDM7 |
| ENSG00000126858 | -1.897 | 1.063e-53 | 2.109e-52 | RHOT1 |
| ENSG00000126860 | 2.7 | 8.883e-07 | 2.358e-06 | EVI2A |
| ENSG00000126861 | 1.495 | 0.0163 | 0.02729 | OMG |
| ENSG00000126870 | -1.484 | 5.12e-10 | 1.722e-09 | WDR60 |
| ENSG00000126882 | -3.528 | 6.787e-14 | 2.89e-13 | FAM78A |
| ENSG00000126895 | 2.815 | 0.0002956 | 0.0006224 | AVPR2 |
| ENSG00000126903 | -2.958 | 2.161e-40 | 2.893e-39 | SLC10A3 |
| ENSG00000126945 | 1.022 | 3.552e-15 | 1.616e-14 | HNRNPH2 |
| ENSG00000126953 | -1.405 | 3.075e-10 | 1.052e-09 | TIMM8A |
| ENSG00000127081 | 1.034 | 1.804e-10 | 6.248e-10 | ZNF484 |
| ENSG00000127084 | -4.373 | 8.011e-85 | 3.756e-83 | FGD3 |
| ENSG00000127125 | -1.8 | 9.773e-37 | 1.15e-35 | PPCS |
| ENSG00000127129 | 3.574 | 4.819e-05 | 0.0001098 | EDN2 |
| ENSG00000127220 | -1.271 | 6.353e-16 | 2.997e-15 | ABHD8 |
| ENSG00000127311 | 1.636 | 3.948e-13 | 1.612e-12 | HELB |
| ENSG00000127329 | -1.712 | 8.055e-12 | 3.036e-11 | PTPRB |
| ENSG00000127334 | -2.214 | 7.76e-93 | 4.43e-91 | DYRK2 |
| ENSG00000127337 | -1.356 | 7.274e-12 | 2.751e-11 | YEATS4 |
| ENSG00000127419 | -1.473 | 3.93e-08 | 1.157e-07 | TMEM175 |
| ENSG00000127423 | -1.899 | 4.657e-14 | 2.002e-13 | AUNIP |
| ENSG00000127554 | -1.384 | 4.232e-24 | 2.946e-23 | GFER |
| ENSG00000127561 | -1.816 | 0.001105 | 0.002182 | SYNGR3 |
| ENSG00000127564 | -2.955 | 7.256e-88 | 3.625e-86 | PKMYT1 |
| ENSG00000127585 | -4.166 | 2.978e-29 | 2.593e-28 | FBXL16 |
| ENSG00000127586 | -1.448 | 1.651e-23 | 1.116e-22 | CHTF18 |
| ENSG00000127663 | -1.544 | 2.02e-10 | 6.979e-10 | KDM4B |
| ENSG00000127666 | 1.26 | 2.035e-06 | 5.216e-06 | TICAM1 |
| ENSG00000127720 | -1.293 | 3.623e-05 | 8.339e-05 | METTL25 |
| ENSG00000127774 | -1.659 | 2.105e-08 | 6.314e-08 | EMC6 |
| ENSG00000127947 | 1.345 | 8.824e-17 | 4.348e-16 | PTPN12 |
| ENSG00000127980 | -1.638 | 5.352e-35 | 5.918e-34 | PEX1 |
| ENSG00000127993 | 2.197 | 8.421e-76 | 3.134e-74 | RBM48 |
| ENSG00000127995 | -2.058 | 7.977e-46 | 1.273e-44 | CASD1 |
| ENSG00000128011 | -3.661 | 1.407e-20 | 8.334e-20 | LRFN1 |
| ENSG00000128016 | 1.861 | 1.332e-19 | 7.547e-19 | ZFP36 |
| ENSG00000128059 | -1.878 | 2.931e-57 | 6.481e-56 | PPAT |
| ENSG00000128159 | -1.076 | 3.893e-11 | 1.411e-10 | TUBGCP6 |
| ENSG00000128165 | 1.709 | 7.438e-09 | 2.31e-08 | ADM2 |
| ENSG00000128191 | -1.95 | 4.764e-70 | 1.532e-68 | DGCR8 |
| ENSG00000128242 | 3.312 | 0.01791 | 0.02979 | GAL3ST1 |
| ENSG00000128245 | -2.697 | 3.375e-94 | 1.974e-92 | YWHAH |
| ENSG00000128253 | 4.549 | 1.092e-07 | 3.115e-07 | RFPL2 |
| ENSG00000128266 | -2.843 | 2.557e-20 | 1.496e-19 | GNAZ |
| ENSG00000128274 | -2.655 | 9.001e-16 | 4.212e-15 | A4GALT |
| ENSG00000128283 | -1.01 | 5.394e-07 | 1.456e-06 | CDC42EP1 |
| ENSG00000128284 | -2.31 | 1.179e-07 | 3.359e-07 | APOL3 |
| ENSG00000128309 | -1.194 | 8.809e-09 | 2.716e-08 | MPST |
| ENSG00000128311 | -1.848 | 2.578e-11 | 9.457e-11 | TST |
| ENSG00000128342 | 1.887 | 1.784e-49 | 3.166e-48 | LIF |
| ENSG00000128346 | -1.142 | 0.003001 | 0.005607 | C22orf23 |
| ENSG00000128383 | 1.266 | 0.001546 | 0.003003 | APOBEC3A |
| ENSG00000128408 | -3.228 | 1.694e-25 | 1.262e-24 | RIBC2 |
| ENSG00000128422 | 3.036 | 4.802e-90 | 2.527e-88 | KRT17 |
| ENSG00000128487 | -1.074 | 1.993e-19 | 1.118e-18 | SPECC1 |
| ENSG00000128510 | -1.474 | 5.439e-33 | 5.53e-32 | CPA4 |
| ENSG00000128512 | 1.455 | 2.163e-11 | 7.975e-11 | DOCK4 |
| ENSG00000128513 | -1.01 | 1.189e-21 | 7.422e-21 | POT1 |
| ENSG00000128536 | -1.413 | 0.007323 | 0.01296 | CDHR3 |
| ENSG00000128564 | 1.15 | 3.98e-07 | 1.087e-06 | VGF |
| ENSG00000128567 | -1.217 | 6.817e-25 | 4.913e-24 | PODXL |
| ENSG00000128590 | 3.279 | 5.345e-123 | 5.608e-121 | DNAJB9 |
| ENSG00000128591 | 1.593 | 1.129e-34 | 1.237e-33 | FLNC |
| ENSG00000128595 | 1.006 | 9.873e-31 | 9.086e-30 | CALU |
| ENSG00000128596 | -1.734 | 9.39e-14 | 3.977e-13 | CCDC136 |
| ENSG00000128606 | 3.445 | 7.801e-08 | 2.248e-07 | LRRC17 |
| ENSG00000128607 | -1.036 | 1.241e-25 | 9.306e-25 | KLHDC10 |
| ENSG00000128626 | -1.604 | 7.837e-11 | 2.781e-10 | MRPS12 |
| ENSG00000128683 | -1.764 | 3.39e-12 | 1.306e-11 | GAD1 |
| ENSG00000128694 | -1.683 | 2.814e-05 | 6.533e-05 | OSGEPL1 |
| ENSG00000128731 | -2.15 | 8.596e-73 | 2.963e-71 | HERC2 |
| ENSG00000128829 | -2.096 | 5.733e-87 | 2.839e-85 | EIF2AK4 |
| ENSG00000128886 | -1.056 | 0.002179 | 0.004155 | ELL3 |
| ENSG00000128891 | 1.14 | 3.228e-11 | 1.176e-10 | C15orf57 |
| ENSG00000128908 | 1.124 | 9.831e-29 | 8.381e-28 | INO80 |
| ENSG00000128928 | -2.008 | 2.318e-38 | 2.908e-37 | IVD |
| ENSG00000128944 | -1.231 | 1.791e-24 | 1.27e-23 | KNSTRN |
| ENSG00000128951 | -1.323 | 8.685e-11 | 3.071e-10 | DUT |
| ENSG00000128965 | 2.919 | 1.572e-12 | 6.19e-12 | CHAC1 |
| ENSG00000129003 | -1.91 | 1.177e-49 | 2.099e-48 | VPS13C |
| ENSG00000129038 | -1.931 | 1.724e-25 | 1.284e-24 | LOXL1 |
| ENSG00000129116 | 1.275 | 1.12e-25 | 8.414e-25 | PALLD |
| ENSG00000129158 | -1.001 | 5.507e-08 | 1.603e-07 | SERGEF |
| ENSG00000129167 | -2.213 | 1.931e-06 | 4.965e-06 | TPH1 |
| ENSG00000129173 | -4.888 | 6.52e-135 | 8.107e-133 | E2F8 |
| ENSG00000129194 | -2.985 | 4.214e-125 | 4.623e-123 | SOX15 |
| ENSG00000129195 | -3.034 | 3.115e-27 | 2.488e-26 | FAM64A |
| ENSG00000129204 | -1.673 | 0.02822 | 0.0453 | USP6 |
| ENSG00000129282 | -3.258 | 1.959e-12 | 7.671e-12 | MRM1 |
| ENSG00000129295 | -1.257 | 0.001131 | 0.002231 | LRRC6 |
| ENSG00000129347 | -2.254 | 5.955e-66 | 1.733e-64 | KRI1 |
| ENSG00000129353 | -1.138 | 1.417e-47 | 2.386e-46 | SLC44A2 |
| ENSG00000129437 | 4.795 | 8.947e-51 | 1.643e-49 | KLK14 |
| ENSG00000129450 | 3.639 | 0.002027 | 0.003885 | SIGLEC9 |
| ENSG00000129460 | 1.784 | 2.942e-60 | 7.261e-59 | NGDN |
| ENSG00000129474 | -1.617 | 1.02e-22 | 6.644e-22 | AJUBA |
| ENSG00000129480 | -2.376 | 2.3e-57 | 5.119e-56 | DTD2 |
| ENSG00000129484 | -1.024 | 2.816e-16 | 1.354e-15 | PARP2 |
| ENSG00000129493 | -1.694 | 5.165e-37 | 6.163e-36 | HEATR5A |
| ENSG00000129514 | -1.542 | 7.436e-18 | 3.873e-17 | FOXA1 |
| ENSG00000129521 | -1.32 | 5.438e-08 | 1.584e-07 | EGLN3 |
| ENSG00000129534 | -1.314 | 7.123e-18 | 3.713e-17 | MIS18BP1 |
| ENSG00000129646 | 1.15 | 3.335e-05 | 7.699e-05 | QRICH2 |
| ENSG00000129675 | 2.455 | 0.004359 | 0.007957 | ARHGEF6 |
| ENSG00000129680 | -1.143 | 3.262e-09 | 1.039e-08 | MAP7D3 |
| ENSG00000129691 | -1.065 | 3.035e-22 | 1.939e-21 | ASH2L |
| ENSG00000129757 | 2.96 | 3.234e-10 | 1.104e-09 | CDKN1C |
| ENSG00000129911 | -1.415 | 1.271e-11 | 4.743e-11 | KLF16 |
| ENSG00000129946 | 1.486 | 0.01014 | 0.01757 | SHC2 |
| ENSG00000129993 | -2.607 | 6.447e-11 | 2.303e-10 | CBFA2T3 |
| ENSG00000130021 | -1.295 | 1.05e-21 | 6.576e-21 | HDHD1 |
| ENSG00000130023 | -1.088 | 1.65e-11 | 6.117e-11 | ERMARD |
| ENSG00000130032 | 4.092 | 0.0005686 | 0.00116 | PRRG3 |
| ENSG00000130052 | 1.935 | 0.01023 | 0.0177 | STARD8 |
| ENSG00000130066 | 3.962 | 0 | 0 | SAT1 |
| ENSG00000130176 | 1.93 | 0.02112 | 0.03467 | CNN1 |
| ENSG00000130177 | -1.076 | 2.132e-25 | 1.58e-24 | CDC16 |
| ENSG00000130182 | 3.512 | 0.003076 | 0.005738 | ZSCAN10 |
| ENSG00000130193 | -2.772 | 2.547e-57 | 5.648e-56 | THEM6 |
| ENSG00000130203 | 4.103 | 5.954e-19 | 3.269e-18 | APOE |
| ENSG00000130222 | 3.04 | 1.813e-19 | 1.02e-18 | GADD45G |
| ENSG00000130287 | 4.55 | 4.058e-05 | 9.308e-05 | NCAN |
| ENSG00000130294 | 2.66 | 0.003263 | 0.00606 | KIF1A |
| ENSG00000130299 | -2.628 | 3.487e-77 | 1.349e-75 | GTPBP3 |
| ENSG00000130300 | 4.16 | 0.0001056 | 0.000233 | PLVAP |
| ENSG00000130305 | -1.152 | 1.501e-09 | 4.898e-09 | NSUN5 |
| ENSG00000130309 | -1.562 | 8.498e-53 | 1.649e-51 | COLGALT1 |
| ENSG00000130311 | 1.198 | 6.984e-11 | 2.487e-10 | DDA1 |
| ENSG00000130312 | -2.57 | 1.35e-20 | 8.001e-20 | MRPL34 |
| ENSG00000130340 | 1.152 | 2.392e-42 | 3.435e-41 | SNX9 |
| ENSG00000130347 | -2.806 | 9.496e-23 | 6.192e-22 | RTN4IP1 |
| ENSG00000130349 | -3.101 | 9.131e-32 | 8.829e-31 | C6orf203 |
| ENSG00000130413 | -1.285 | 1.271e-06 | 3.326e-06 | STK33 |
| ENSG00000130433 | -1.567 | 7.334e-29 | 6.284e-28 | CACNG6 |
| ENSG00000130449 | 4.364 | 1.27e-168 | 2.666e-166 | ZSWIM6 |
| ENSG00000130475 | -1.295 | 2.642e-15 | 1.207e-14 | FCHO1 |
| ENSG00000130489 | -2.857 | 3.609e-23 | 2.4e-22 | SCO2 |
| ENSG00000130508 | -1.375 | 6.607e-39 | 8.485e-38 | PXDN |
| ENSG00000130511 | -1.852 | 4.338e-31 | 4.05e-30 | SSBP4 |
| ENSG00000130513 | 2.853 | 1.472e-43 | 2.205e-42 | GDF15 |
| ENSG00000130517 | -1.802 | 1.469e-23 | 9.956e-23 | PGPEP1 |
| ENSG00000130518 | 1.543 | 0.00197 | 0.00378 | KIAA1683 |
| ENSG00000130522 | 1.095 | 1.28e-25 | 9.59e-25 | JUND |
| ENSG00000130529 | -1.159 | 3.011e-14 | 1.305e-13 | TRPM4 |
| ENSG00000130544 | 1.565 | 9.043e-17 | 4.452e-16 | ZNF557 |
| ENSG00000130560 | -1.804 | 5.684e-19 | 3.124e-18 | UBAC1 |
| ENSG00000130590 | -2.023 | 2.922e-28 | 2.427e-27 | SAMD10 |
| ENSG00000130649 | 3.885 | 3.11e-10 | 1.062e-09 | CYP2E1 |
| ENSG00000130653 | 1.097 | 0.0254 | 0.0411 | PNPLA7 |
| ENSG00000130675 | -2.448 | 8.175e-12 | 3.08e-11 | MNX1 |
| ENSG00000130684 | 1.194 | 8.257e-10 | 2.744e-09 | ZNF337 |
| ENSG00000130701 | -3.793 | 2.145e-25 | 1.588e-24 | RBBP8NL |
| ENSG00000130702 | -1.871 | 5.817e-47 | 9.657e-46 | LAMA5 |
| ENSG00000130703 | 1.261 | 2.697e-44 | 4.116e-43 | OSBPL2 |
| ENSG00000130713 | -1.41 | 4.55e-14 | 1.957e-13 | EXOSC2 |
| ENSG00000130723 | -1.106 | 3.882e-54 | 7.785e-53 | PRRC2B |
| ENSG00000130726 | -1.506 | 3.275e-27 | 2.613e-26 | TRIM28 |
| ENSG00000130762 | -2.683 | 1.921e-40 | 2.582e-39 | ARHGEF16 |
| ENSG00000130764 | -1.822 | 2.714e-09 | 8.686e-09 | LRRC47 |
| ENSG00000130766 | 4.86 | 1.731e-191 | 5.189e-189 | SESN2 |
| ENSG00000130783 | 2.753 | 9.566e-06 | 2.318e-05 | CCDC62 |
| ENSG00000130813 | -1.542 | 3.108e-18 | 1.651e-17 | C19orf66 |
| ENSG00000130816 | -1.14 | 6.061e-25 | 4.381e-24 | DNMT1 |
| ENSG00000130822 | 6.5 | 1.551e-12 | 6.113e-12 | PNCK |
| ENSG00000130856 | 1.12 | 8.828e-27 | 6.918e-26 | ZNF236 |
| ENSG00000130881 | -1.521 | 9.954e-06 | 2.407e-05 | LRP3 |
| ENSG00000131013 | 1.139 | 1.896e-12 | 7.431e-12 | PPIL4 |
| ENSG00000131016 | 2.195 | 2.241e-74 | 8.09e-73 | AKAP12 |
| ENSG00000131018 | 3.924 | 1.428e-92 | 8.068e-91 | SYNE1 |
| ENSG00000131019 | -1.464 | 0.0001216 | 0.0002661 | ULBP3 |
| ENSG00000131037 | 1.179 | 3.271e-17 | 1.647e-16 | EPS8L1 |
| ENSG00000131051 | 1.217 | 1.997e-59 | 4.794e-58 | RBM39 |
| ENSG00000131061 | -1.372 | 2.645e-11 | 9.697e-11 | ZNF341 |
| ENSG00000131089 | -1.406 | 9.573e-40 | 1.26e-38 | ARHGEF9 |
| ENSG00000131095 | 2.638 | 0.0146 | 0.02465 | GFAP |
| ENSG00000131096 | 3.247 | 0.01871 | 0.03101 | PYY |
| ENSG00000131100 | 1.07 | 1.083e-21 | 6.781e-21 | ATP6V1E1 |
| ENSG00000131116 | -1.028 | 2.688e-09 | 8.606e-09 | ZNF428 |
| ENSG00000131149 | -1.643 | 3.774e-32 | 3.709e-31 | GSE1 |
| ENSG00000131153 | -1.693 | 1.965e-19 | 1.103e-18 | GINS2 |
| ENSG00000131187 | -1.511 | 6.239e-09 | 1.947e-08 | F12 |
| ENSG00000131188 | -1.24 | 2.018e-09 | 6.52e-09 | PRR7 |
| ENSG00000131236 | 1.037 | 9.006e-44 | 1.355e-42 | CAP1 |
| ENSG00000131242 | -1.577 | 3.193e-32 | 3.145e-31 | RAB11FIP4 |
| ENSG00000131368 | -1.238 | 1.008e-18 | 5.476e-18 | MRPS25 |
| ENSG00000131375 | -1.246 | 3.15e-20 | 1.835e-19 | CAPN7 |
| ENSG00000131409 | 2.469 | 0.001517 | 0.002951 | LRRC4B |
| ENSG00000131435 | 1.188 | 6.486e-05 | 0.0001459 | PDLIM4 |
| ENSG00000131437 | -1.596 | 1.665e-38 | 2.101e-37 | KIF3A |
| ENSG00000131446 | -1.025 | 3.924e-06 | 9.825e-06 | MGAT1 |
| ENSG00000131462 | -1.131 | 3.535e-12 | 1.361e-11 | TUBG1 |
| ENSG00000131471 | 3.897 | 1.47e-16 | 7.154e-16 | AOC3 |
| ENSG00000131475 | -1.026 | 2.348e-20 | 1.377e-19 | VPS25 |
| ENSG00000131480 | 3.318 | 1.91e-33 | 1.969e-32 | AOC2 |
| ENSG00000131650 | -2.274 | 1.045e-25 | 7.858e-25 | KREMEN2 |
| ENSG00000131669 | -1.677 | 4.821e-42 | 6.863e-41 | NINJ1 |
| ENSG00000131697 | -1.034 | 2.476e-13 | 1.025e-12 | NPHP4 |
| ENSG00000131711 | 2.39 | 1.409e-51 | 2.64e-50 | MAP1B |
| ENSG00000131730 | 2.878 | 9.383e-20 | 5.352e-19 | CKMT2 |
| ENSG00000131737 | 2.529 | 5.564e-36 | 6.376e-35 | KRT34 |
| ENSG00000131746 | -1.448 | 7.505e-16 | 3.525e-15 | TNS4 |
| ENSG00000131747 | -3.801 | 0 | 0 | TOP2A |
| ENSG00000131779 | -4.876 | 1.778e-52 | 3.426e-51 | PEX11B |
| ENSG00000131788 | -2.746 | 5.627e-62 | 1.462e-60 | PIAS3 |
| ENSG00000131791 | -1.321 | 4.501e-29 | 3.895e-28 | PRKAB2 |
| ENSG00000131795 | -1.076 | 3.587e-18 | 1.899e-17 | RBM8A |
| ENSG00000131848 | -1.306 | 7.834e-18 | 4.067e-17 | ZSCAN5A |
| ENSG00000131849 | -1.116 | 0.001146 | 0.002258 | ZNF132 |
| ENSG00000131871 | 1.323 | 1.181e-27 | 9.598e-27 | VIMP |
| ENSG00000131873 | -2.011 | 2.437e-48 | 4.165e-47 | CHSY1 |
| ENSG00000131931 | 1.38 | 8.013e-20 | 4.581e-19 | THAP1 |
| ENSG00000131941 | -1.475 | 2.755e-28 | 2.292e-27 | RHPN2 |
| ENSG00000131943 | -1.347 | 9.725e-18 | 5.038e-17 | C19orf12 |
| ENSG00000131944 | -1.844 | 6.552e-09 | 2.04e-08 | C19orf40 |
| ENSG00000132002 | 5.146 | 0 | 0 | DNAJB1 |
| ENSG00000132003 | 3.021 | 2.2e-136 | 2.84e-134 | ZSWIM4 |
| ENSG00000132016 | -2.513 | 4.77e-15 | 2.157e-14 | C19orf57 |
| ENSG00000132130 | -2.851 | 1.098e-30 | 1.008e-29 | LHX1 |
| ENSG00000132142 | -1.197 | 1.477e-30 | 1.348e-29 | ACACA |
| ENSG00000132153 | -2.073 | 1.818e-50 | 3.302e-49 | DHX30 |
| ENSG00000132182 | -1.427 | 8.414e-41 | 1.144e-39 | NUP210 |
| ENSG00000132185 | 3.511 | 0.0003805 | 0.0007911 | FCRLA |
| ENSG00000132196 | 1.319 | 3.824e-20 | 2.22e-19 | HSD17B7 |
| ENSG00000132199 | -2.096 | 1.881e-25 | 1.398e-24 | ENOSF1 |
| ENSG00000132274 | 1.891 | 0.02596 | 0.04195 | TRIM22 |
| ENSG00000132305 | -1.717 | 2.98e-91 | 1.629e-89 | IMMT |
| ENSG00000132313 | -2.422 | 2.757e-86 | 1.341e-84 | MRPL35 |
| ENSG00000132330 | -1.247 | 1.059e-15 | 4.945e-15 | SCLY |
| ENSG00000132361 | -1.668 | 4.379e-83 | 1.94e-81 | CLUH |
| ENSG00000132382 | -1.3 | 3.68e-46 | 5.933e-45 | MYBBP1A |
| ENSG00000132383 | -1.262 | 1.122e-54 | 2.285e-53 | RPA1 |
| ENSG00000132386 | -2.433 | 2.909e-05 | 6.746e-05 | SERPINF1 |
| ENSG00000132394 | -1.956 | 7.544e-27 | 5.928e-26 | EEFSEC |
| ENSG00000132405 | -1.867 | 1.364e-30 | 1.248e-29 | TBC1D14 |
| ENSG00000132423 | -1.406 | 9.43e-08 | 2.702e-07 | COQ3 |
| ENSG00000132429 | 1.182 | 1.101e-05 | 2.651e-05 | POPDC3 |
| ENSG00000132432 | 1.261 | 1.567e-32 | 1.564e-31 | SEC61G |
| ENSG00000132434 | -1.162 | 8.762e-37 | 1.035e-35 | LANCL2 |
| ENSG00000132436 | -2.684 | 6.549e-108 | 4.952e-106 | FIGNL1 |
| ENSG00000132437 | 2.487 | 0.0008884 | 0.001771 | DDC |
| ENSG00000132471 | 2.09 | 9.703e-42 | 1.368e-40 | WBP2 |
| ENSG00000132478 | -1.652 | 2.972e-26 | 2.283e-25 | UNK |
| ENSG00000132481 | -3.681 | 4.999e-51 | 9.242e-50 | TRIM47 |
| ENSG00000132485 | 1.478 | 1.742e-33 | 1.8e-32 | ZRANB2 |
| ENSG00000132510 | 3.757 | 1.142e-73 | 4.027e-72 | KDM6B |
| ENSG00000132570 | -2.573 | 4.037e-17 | 2.026e-16 | PCBD2 |
| ENSG00000132591 | -1.934 | 1.244e-51 | 2.336e-50 | ERAL1 |
| ENSG00000132600 | -1.102 | 1.408e-23 | 9.559e-23 | PRMT7 |
| ENSG00000132623 | -1.512 | 5.339e-31 | 4.965e-30 | ANKEF1 |
| ENSG00000132635 | -2.023 | 6.782e-31 | 6.293e-30 | PCED1A |
| ENSG00000132640 | -1.581 | 1.462e-26 | 1.136e-25 | BTBD3 |
| ENSG00000132646 | -2.992 | 2.528e-155 | 4.514e-153 | PCNA |
| ENSG00000132661 | -2.066 | 2.018e-22 | 1.299e-21 | NXT1 |
| ENSG00000132669 | -2.869 | 7e-71 | 2.291e-69 | RIN2 |
| ENSG00000132670 | -2.311 | 3.513e-54 | 7.063e-53 | PTPRA |
| ENSG00000132677 | -1.438 | 0.0004744 | 0.0009757 | RHBG |
| ENSG00000132681 | 2.98 | 0.01713 | 0.02858 | ATP1A4 |
| ENSG00000132688 | -1.22 | 2.985e-06 | 7.547e-06 | NES |
| ENSG00000132692 | 1.949 | 4.746e-25 | 3.449e-24 | BCAN |
| ENSG00000132694 | -1.808 | 4.389e-79 | 1.75e-77 | ARHGEF11 |
| ENSG00000132698 | -1.032 | 1.575e-11 | 5.844e-11 | RAB25 |
| ENSG00000132740 | 1.862 | 3.263e-32 | 3.213e-31 | IGHMBP2 |
| ENSG00000132749 | -2.009 | 1.245e-11 | 4.652e-11 | MTL5 |
| ENSG00000132763 | -2.967 | 3.927e-31 | 3.678e-30 | MMACHC |
| ENSG00000132768 | -1.184 | 4.775e-29 | 4.125e-28 | DPH2 |
| ENSG00000132773 | -1.729 | 2.849e-27 | 2.279e-26 | TOE1 |
| ENSG00000132781 | -1.504 | 3.225e-12 | 1.246e-11 | MUTYH |
| ENSG00000132793 | -2.746 | 1.837e-11 | 6.796e-11 | LPIN3 |
| ENSG00000132819 | -2.021 | 4.889e-32 | 4.787e-31 | RBM38 |
| ENSG00000132823 | 2.305 | 4.327e-96 | 2.613e-94 | OSER1 |
| ENSG00000132825 | -3.072 | 1.098e-36 | 1.286e-35 | PPP1R3D |
| ENSG00000132854 | -3.313 | 7.702e-10 | 2.563e-09 | KANK4 |
| ENSG00000132855 | 2.126 | 0.01224 | 0.02092 | ANGPTL3 |
| ENSG00000132879 | -1.395 | 1.454e-12 | 5.738e-12 | FBXO44 |
| ENSG00000132881 | -1.474 | 0.001557 | 0.003024 | RSG1 |
| ENSG00000132906 | -1.089 | 2.054e-05 | 4.831e-05 | CASP9 |
| ENSG00000132938 | 6.48 | 2.483e-20 | 1.455e-19 | MTUS2 |
| ENSG00000132952 | 1.794 | 2.37e-75 | 8.744e-74 | USPL1 |
| ENSG00000132970 | -1.721 | 2.487e-06 | 6.324e-06 | WASF3 |
| ENSG00000133019 | -3.282 | 0.002435 | 0.004609 | CHRM3 |
| ENSG00000133026 | -1.242 | 8.33e-42 | 1.178e-40 | MYH10 |
| ENSG00000133056 | -4.014 | 2.245e-181 | 5.625e-179 | PIK3C2B |
| ENSG00000133059 | -2.219 | 8.059e-49 | 1.406e-47 | DSTYK |
| ENSG00000133069 | -1.238 | 0.000682 | 0.001377 | TMCC2 |
| ENSG00000133083 | 1.83 | 1.453e-15 | 6.713e-15 | DCLK1 |
| ENSG00000133101 | 4.242 | 2.095e-09 | 6.76e-09 | CCNA1 |
| ENSG00000133107 | -1.528 | 2.917e-10 | 9.987e-10 | TRPC4 |
| ENSG00000133110 | 2.863 | 0.0001178 | 0.0002583 | POSTN |
| ENSG00000133111 | -2.654 | 1.294e-17 | 6.648e-17 | RFXAP |
| ENSG00000133112 | 1.077 | 1.095e-23 | 7.477e-23 | TPT1 |
| ENSG00000133119 | -1.321 | 3.615e-34 | 3.872e-33 | RFC3 |
| ENSG00000133134 | 2.008 | 2.333e-05 | 5.462e-05 | BEX2 |
| ENSG00000133169 | 3.059 | 0.02355 | 0.03831 | BEX1 |
| ENSG00000133243 | -1.211 | 3.125e-23 | 2.088e-22 | BTBD2 |
| ENSG00000133250 | -1.78 | 7.106e-10 | 2.369e-09 | ZNF414 |
| ENSG00000133302 | -1.664 | 4.355e-35 | 4.822e-34 | ANKRD32 |
| ENSG00000133398 | 1.444 | 2.178e-34 | 2.361e-33 | MED10 |
| ENSG00000133460 | -1.772 | 2.209e-17 | 1.122e-16 | SLC2A11 |
| ENSG00000133466 | -4.134 | 1.639e-234 | 7.862e-232 | C1QTNF6 |
| ENSG00000133477 | -2.659 | 0.0004241 | 0.0008773 | FAM83F |
| ENSG00000133597 | -2.453 | 2.555e-20 | 1.495e-19 | ADCK2 |
| ENSG00000133612 | -1.796 | 5.44e-55 | 1.118e-53 | AGAP3 |
| ENSG00000133627 | -1.313 | 9.564e-14 | 4.049e-13 | ACTR3B |
| ENSG00000133639 | 1.035 | 4.986e-19 | 2.746e-18 | BTG1 |
| ENSG00000133641 | -1.315 | 2.951e-19 | 1.642e-18 | C12orf29 |
| ENSG00000133657 | 1.366 | 9.993e-64 | 2.71e-62 | ATP13A3 |
| ENSG00000133678 | -1.157 | 6.647e-21 | 4.004e-20 | TMEM254 |
| ENSG00000133703 | -1.178 | 1.784e-27 | 1.435e-26 | KRAS |
| ENSG00000133739 | -1.416 | 2.185e-08 | 6.548e-08 | LRRCC1 |
| ENSG00000133773 | 1.199 | 3.996e-11 | 1.447e-10 | CCDC59 |
| ENSG00000133812 | 1.558 | 1.186e-45 | 1.884e-44 | SBF2 |
| ENSG00000133818 | 1.507 | 7.762e-60 | 1.883e-58 | RRAS2 |
| ENSG00000133863 | -1.393 | 2.398e-18 | 1.281e-17 | TEX15 |
| ENSG00000133895 | -2.797 | 1.777e-111 | 1.42e-109 | MEN1 |
| ENSG00000133997 | 1.73 | 5.683e-39 | 7.31e-38 | MED6 |
| ENSG00000134049 | -1.079 | 3.536e-19 | 1.964e-18 | IER3IP1 |
| ENSG00000134057 | -3.163 | 5.164e-91 | 2.787e-89 | CCNB1 |
| ENSG00000134138 | -2.33 | 1.206e-33 | 1.254e-32 | MEIS2 |
| ENSG00000134152 | 1.742 | 4.369e-38 | 5.448e-37 | KATNBL1 |
| ENSG00000134183 | 1.191 | 0.0003197 | 0.0006703 | GNAT2 |
| ENSG00000134186 | 1.609 | 4.356e-31 | 4.065e-30 | PRPF38B |
| ENSG00000134207 | 1.14 | 5.041e-05 | 0.0001147 | SYT6 |
| ENSG00000134215 | -1.871 | 5.997e-17 | 2.98e-16 | VAV3 |
| ENSG00000134222 | -4.679 | 1.601e-85 | 7.594e-84 | PSRC1 |
| ENSG00000134243 | -1.431 | 1.192e-31 | 1.146e-30 | SORT1 |
| ENSG00000134245 | 1.059 | 4.155e-07 | 1.132e-06 | WNT2B |
| ENSG00000134247 | -2.117 | 7.231e-52 | 1.367e-50 | PTGFRN |
| ENSG00000134248 | -1.115 | 1.53e-06 | 3.968e-06 | LAMTOR5 |
| ENSG00000134250 | -1.02 | 5.03e-35 | 5.566e-34 | NOTCH2 |
| ENSG00000134253 | -1.822 | 5.531e-29 | 4.759e-28 | TRIM45 |
| ENSG00000134256 | -1.06 | 4.717e-06 | 1.173e-05 | CD101 |
| ENSG00000134259 | -2.388 | 1.828e-20 | 1.077e-19 | NGF |
| ENSG00000134278 | 1.256 | 1.086e-26 | 8.468e-26 | SPIRE1 |
| ENSG00000134294 | 1.676 | 1.634e-135 | 2.093e-133 | SLC38A2 |
| ENSG00000134321 | 4.382 | 3.592e-05 | 8.27e-05 | RSAD2 |
| ENSG00000134369 | -2.199 | 1.045e-96 | 6.376e-95 | NAV1 |
| ENSG00000134398 | 3.841 | 4.872e-05 | 0.0001109 | ERN2 |
| ENSG00000134461 | -1.995 | 1.449e-09 | 4.736e-09 | ANKRD16 |
| ENSG00000134470 | -1.525 | 2.004e-06 | 5.141e-06 | IL15RA |
| ENSG00000134489 | -2.865 | 0.01639 | 0.02744 | HRH4 |
| ENSG00000134504 | -1.463 | 1.842e-21 | 1.142e-20 | KCTD1 |
| ENSG00000134508 | -1.814 | 3.353e-28 | 2.778e-27 | CABLES1 |
| ENSG00000134548 | 3.664 | 3.154e-06 | 7.965e-06 | C12orf39 |
| ENSG00000134569 | -1.394 | 3.622e-06 | 9.098e-06 | LRP4 |
| ENSG00000134574 | -1.256 | 1.567e-14 | 6.89e-14 | DDB2 |
| ENSG00000134575 | -2.188 | 2.364e-40 | 3.159e-39 | ACP2 |
| ENSG00000134590 | -1.674 | 1.482e-18 | 8.008e-18 | FAM127A |
| ENSG00000134684 | 1.047 | 6.568e-37 | 7.803e-36 | YARS |
| ENSG00000134690 | -1.906 | 7.176e-76 | 2.689e-74 | CDCA8 |
| ENSG00000134716 | -1.08 | 1.105e-08 | 3.379e-08 | CYP2J2 |
| ENSG00000134748 | 1.061 | 3.904e-15 | 1.772e-14 | PRPF38A |
| ENSG00000134765 | 2.819 | 0.0001138 | 0.0002502 | DSC1 |
| ENSG00000134769 | 1.821 | 0.000646 | 0.001311 | DTNA |
| ENSG00000134775 | -1.221 | 2.819e-13 | 1.162e-12 | FHOD3 |
| ENSG00000134780 | -1.71 | 4.669e-27 | 3.697e-26 | DAGLA |
| ENSG00000134809 | -1.136 | 1.849e-13 | 7.71e-13 | TIMM10 |
| ENSG00000134852 | -1.167 | 7.451e-18 | 3.879e-17 | CLOCK |
| ENSG00000134864 | -1.217 | 0.002914 | 0.005448 | GGACT |
| ENSG00000134874 | -1.946 | 0.002573 | 0.004858 | DZIP1 |
| ENSG00000134884 | -1.007 | 2.883e-31 | 2.719e-30 | ARGLU1 |
| ENSG00000134897 | -3.059 | 7.433e-29 | 6.366e-28 | BIVM |
| ENSG00000134901 | -3.926 | 2.967e-31 | 2.795e-30 | KDELC1 |
| ENSG00000134954 | 1.331 | 6.64e-34 | 7.014e-33 | ETS1 |
| ENSG00000134962 | 1.541 | 0.00545 | 0.009824 | KLB |
| ENSG00000135018 | 1.49 | 3.703e-74 | 1.328e-72 | UBQLN1 |
| ENSG00000135045 | -2.633 | 2.113e-22 | 1.358e-21 | C9orf40 |
| ENSG00000135046 | 1.654 | 2.129e-120 | 2.153e-118 | ANXA1 |
| ENSG00000135074 | -3.048 | 1.003e-57 | 2.248e-56 | ADAM19 |
| ENSG00000135077 | 3.327 | 0.006048 | 0.01083 | HAVCR2 |
| ENSG00000135083 | -2.272 | 3.378e-10 | 1.151e-09 | CCNJL |
| ENSG00000135093 | 1.262 | 1.492e-19 | 8.428e-19 | USP30 |
| ENSG00000135094 | 1.561 | 0.02214 | 0.03622 | SDS |
| ENSG00000135114 | -2.021 | 0.0001156 | 0.0002539 | OASL |
| ENSG00000135119 | -1.83 | 1.32e-09 | 4.325e-09 | RNFT2 |
| ENSG00000135124 | -1.036 | 2.023e-12 | 7.912e-12 | P2RX4 |
| ENSG00000135127 | -1.615 | 7.146e-21 | 4.296e-20 | CCDC64 |
| ENSG00000135148 | -1.048 | 7.68e-18 | 3.995e-17 | TRAFD1 |
| ENSG00000135185 | -1.936 | 5.247e-14 | 2.246e-13 | TMEM243 |
| ENSG00000135211 | -3.121 | 1.111e-16 | 5.439e-16 | TMEM60 |
| ENSG00000135241 | 2.213 | 8.272e-100 | 5.32e-98 | PNPLA8 |
| ENSG00000135245 | -4.563 | 1.172e-39 | 1.539e-38 | HILPDA |
| ENSG00000135248 | 2.116 | 0.005769 | 0.01036 | FAM71F1 |
| ENSG00000135269 | 2.291 | 7.139e-117 | 6.443e-115 | TES |
| ENSG00000135272 | -1.944 | 1.126e-51 | 2.124e-50 | MDFIC |
| ENSG00000135314 | -2.086 | 6.222e-12 | 2.363e-11 | KHDC1 |
| ENSG00000135318 | -1.016 | 1.833e-17 | 9.346e-17 | NT5E |
| ENSG00000135324 | 3.683 | 0.001563 | 0.003035 | MRAP2 |
| ENSG00000135334 | 1.347 | 1.002e-21 | 6.278e-21 | AKIRIN2 |
| ENSG00000135336 | -1.103 | 2.354e-14 | 1.026e-13 | ORC3 |
| ENSG00000135346 | 3.77 | 4.765e-05 | 0.0001086 | CGA |
| ENSG00000135362 | -1.358 | 0.005392 | 0.009727 | PRR5L |
| ENSG00000135365 | -1.15 | 2.525e-07 | 7.002e-07 | PHF21A |
| ENSG00000135372 | -1.193 | 5.169e-33 | 5.269e-32 | NAT10 |
| ENSG00000135373 | 1.968 | 1.176e-103 | 8.261e-102 | EHF |
| ENSG00000135374 | -1.72 | 0.0004973 | 0.001021 | ELF5 |
| ENSG00000135414 | -1.608 | 1.132e-10 | 3.98e-10 | GDF11 |
| ENSG00000135439 | -3.671 | 0.0008282 | 0.001657 | AGAP2 |
| ENSG00000135451 | -2.575 | 1.296e-78 | 5.155e-77 | TROAP |
| ENSG00000135469 | -2.394 | 3.967e-13 | 1.619e-12 | COQ10A |
| ENSG00000135473 | -1.418 | 7.357e-16 | 3.457e-15 | PAN2 |
| ENSG00000135476 | -2.254 | 5.635e-64 | 1.543e-62 | ESPL1 |
| ENSG00000135503 | -1.787 | 5.519e-32 | 5.389e-31 | ACVR1B |
| ENSG00000135604 | 3.242 | 6.193e-05 | 0.0001397 | STX11 |
| ENSG00000135617 | -1.58 | 6.598e-13 | 2.658e-12 | PRADC1 |
| ENSG00000135622 | -2.006 | 1.834e-19 | 1.031e-18 | SEMA4F |
| ENSG00000135631 | -1.366 | 1.28e-19 | 7.254e-19 | RAB11FIP5 |
| ENSG00000135632 | -1.472 | 1.097e-16 | 5.375e-16 | SMYD5 |
| ENSG00000135638 | 3.209 | 0.01902 | 0.03147 | EMX1 |
| ENSG00000135655 | 1.246 | 9.748e-23 | 6.352e-22 | USP15 |
| ENSG00000135686 | -1.19 | 4.594e-11 | 1.657e-10 | KLHL36 |
| ENSG00000135723 | -1.96 | 6.817e-52 | 1.292e-50 | FHOD1 |
| ENSG00000135736 | -2.608 | 7.211e-16 | 3.395e-15 | CCDC102A |
| ENSG00000135740 | 1.189 | 5.149e-12 | 1.965e-11 | SLC9A5 |
| ENSG00000135763 | -1.833 | 4.105e-61 | 1.036e-59 | URB2 |
| ENSG00000135766 | -1.26 | 4.434e-28 | 3.663e-27 | EGLN1 |
| ENSG00000135775 | -2.823 | 3.305e-116 | 2.967e-114 | COG2 |
| ENSG00000135776 | -2.485 | 2.277e-35 | 2.56e-34 | ABCB10 |
| ENSG00000135801 | -1.548 | 2.847e-63 | 7.635e-62 | TAF5L |
| ENSG00000135828 | -1.512 | 1.798e-10 | 6.228e-10 | RNASEL |
| ENSG00000135845 | -2.735 | 6.382e-48 | 1.084e-46 | PIGC |
| ENSG00000135870 | 1.788 | 1.253e-34 | 1.368e-33 | RC3H1 |
| ENSG00000135900 | -1.104 | 3.826e-13 | 1.565e-12 | MRPL44 |
| ENSG00000135914 | 5.232 | 3.109e-37 | 3.747e-36 | HTR2B |
| ENSG00000135917 | -2.485 | 8.934e-09 | 2.752e-08 | SLC19A3 |
| ENSG00000135919 | 1.99 | 1.835e-84 | 8.531e-83 | SERPINE2 |
| ENSG00000135924 | 1.113 | 3.038e-11 | 1.108e-10 | DNAJB2 |
| ENSG00000135925 | -1.332 | 6.479e-05 | 0.0001458 | WNT10A |
| ENSG00000135930 | -1.353 | 2.556e-20 | 1.496e-19 | EIF4E2 |
| ENSG00000135931 | -1.673 | 6.263e-27 | 4.933e-26 | ARMC9 |
| ENSG00000135966 | -2.498 | 4.873e-76 | 1.83e-74 | TGFBRAP1 |
| ENSG00000135976 | -2.493 | 3.739e-22 | 2.381e-21 | ANKRD36 |
| ENSG00000136010 | -1.348 | 0.02199 | 0.03601 | ALDH1L2 |
| ENSG00000136014 | -2.865 | 8.325e-14 | 3.532e-13 | USP44 |
| ENSG00000136026 | -2.071 | 3.439e-62 | 9.021e-61 | CKAP4 |
| ENSG00000136048 | -1.722 | 3.96e-12 | 1.522e-11 | DRAM1 |
| ENSG00000136098 | -2.092 | 0.02522 | 0.04084 | NEK3 |
| ENSG00000136100 | -1.083 | 6.679e-16 | 3.149e-15 | VPS36 |
| ENSG00000136108 | -3.654 | 3.749e-119 | 3.659e-117 | CKAP2 |
| ENSG00000136111 | -2.725 | 8.497e-44 | 1.279e-42 | TBC1D4 |
| ENSG00000136122 | -1.814 | 9.992e-27 | 7.801e-26 | BORA |
| ENSG00000136144 | -1.387 | 8.371e-21 | 5.008e-20 | RCBTB1 |
| ENSG00000136152 | 1.018 | 1.425e-28 | 1.204e-27 | COG3 |
| ENSG00000136158 | -1.057 | 2.142e-08 | 6.425e-08 | SPRY2 |
| ENSG00000136161 | -2.38 | 2.34e-10 | 8.06e-10 | RCBTB2 |
| ENSG00000136167 | 1.407 | 2.007e-84 | 9.306e-83 | LCP1 |
| ENSG00000136169 | -2.578 | 1.014e-23 | 6.942e-23 | SETDB2 |
| ENSG00000136193 | -1.339 | 1.997e-46 | 3.245e-45 | SCRN1 |
| ENSG00000136197 | -3.051 | 0.0001146 | 0.0002519 | C7orf25 |
| ENSG00000136205 | -3.211 | 2.694e-253 | 1.507e-250 | TNS3 |
| ENSG00000136213 | -1.68 | 3.313e-10 | 1.13e-09 | CHST12 |
| ENSG00000136237 | -1.345 | 2.649e-25 | 1.949e-24 | RAPGEF5 |
| ENSG00000136243 | -1.405 | 2.837e-23 | 1.899e-22 | NUPL2 |
| ENSG00000136244 | 2.384 | 1.733e-13 | 7.239e-13 | IL6 |
| ENSG00000136280 | -1.455 | 1.266e-22 | 8.218e-22 | CCM2 |
| ENSG00000136319 | -2.247 | 2.978e-32 | 2.942e-31 | TTC5 |
| ENSG00000136371 | -1.931 | 1.44e-19 | 8.152e-19 | MTHFS |
| ENSG00000136378 | -1.128 | 0.02759 | 0.04435 | ADAMTS7 |
| ENSG00000136379 | -2.254 | 1.295e-60 | 3.226e-59 | ABHD17C |
| ENSG00000136383 | -1.108 | 1.188e-05 | 2.853e-05 | ALPK3 |
| ENSG00000136436 | 1.655 | 1.615e-44 | 2.479e-43 | CALCOCO2 |
| ENSG00000136444 | -3.582 | 4.833e-24 | 3.358e-23 | RSAD1 |
| ENSG00000136457 | -4.021 | 2.931e-08 | 8.706e-08 | CHAD |
| ENSG00000136463 | -1.77 | 1.672e-25 | 1.247e-24 | TACO1 |
| ENSG00000136492 | -1.234 | 2.044e-21 | 1.263e-20 | BRIP1 |
| ENSG00000136527 | 1.537 | 2.924e-62 | 7.682e-61 | TRA2B |
| ENSG00000136535 | 5.514 | 2.107e-16 | 1.018e-15 | TBR1 |
| ENSG00000136603 | 1.17 | 3.58e-38 | 4.475e-37 | SKIL |
| ENSG00000136630 | -1.267 | 0.001966 | 0.003774 | HLX |
| ENSG00000136631 | -1.231 | 2.467e-25 | 1.819e-24 | VPS45 |
| ENSG00000136636 | -3.532 | 1.489e-185 | 3.968e-183 | KCTD3 |
| ENSG00000136653 | -1.046 | 4.586e-07 | 1.246e-06 | RASSF5 |
| ENSG00000136688 | 2.306 | 0.0234 | 0.0381 | IL36G |
| ENSG00000136689 | -1.385 | 3.562e-07 | 9.774e-07 | IL1RN |
| ENSG00000136696 | 5.048 | 1.375e-06 | 3.584e-06 | IL36B |
| ENSG00000136710 | -1.094 | 1.255e-10 | 4.401e-10 | CCDC115 |
| ENSG00000136717 | -1.536 | 5.167e-36 | 5.924e-35 | BIN1 |
| ENSG00000136720 | -2.355 | 3.182e-100 | 2.095e-98 | HS6ST1 |
| ENSG00000136738 | 1.321 | 3.402e-13 | 1.394e-12 | STAM |
| ENSG00000136802 | 1.241 | 1.155e-28 | 9.822e-28 | LRRC8A |
| ENSG00000136827 | -1.041 | 6.12e-25 | 4.42e-24 | TOR1A |
| ENSG00000136828 | -1.412 | 1.148e-18 | 6.228e-18 | RALGPS1 |
| ENSG00000136848 | -1.681 | 1.76e-17 | 8.982e-17 | DAB2IP |
| ENSG00000136856 | -1.595 | 2.959e-09 | 9.448e-09 | SLC2A8 |
| ENSG00000136866 | 1.621 | 2.776e-07 | 7.679e-07 | ZFP37 |
| ENSG00000136869 | -1.16 | 4.645e-06 | 1.156e-05 | TLR4 |
| ENSG00000136874 | -1.191 | 2.411e-12 | 9.385e-12 | STX17 |
| ENSG00000136881 | 3.196 | 0.01078 | 0.01859 | BAAT |
| ENSG00000136888 | 1.506 | 7.256e-48 | 1.228e-46 | ATP6V1G1 |
| ENSG00000136895 | -2.048 | 8.38e-11 | 2.966e-10 | GARNL3 |
| ENSG00000136932 | 1.198 | 5.399e-25 | 3.913e-24 | C9orf156 |
| ENSG00000136944 | -1.617 | 0.000126 | 0.0002753 | LMX1B |
| ENSG00000136960 | 6.279 | 5.008e-12 | 1.914e-11 | ENPP2 |
| ENSG00000136982 | -1.59 | 2.682e-29 | 2.34e-28 | DSCC1 |
| ENSG00000136997 | -2.311 | 1.918e-83 | 8.655e-82 | MYC |
| ENSG00000137038 | -1.055 | 0.0005971 | 0.001216 | TMEM261 |
| ENSG00000137040 | -1.917 | 4.887e-29 | 4.215e-28 | RANBP6 |
| ENSG00000137055 | 1.036 | 7.183e-22 | 4.514e-21 | PLAA |
| ENSG00000137090 | 3.231 | 0.01545 | 0.02598 | DMRT1 |
| ENSG00000137094 | 3.515 | 2.97e-30 | 2.675e-29 | DNAJB5 |
| ENSG00000137103 | -1.222 | 0.01994 | 0.03289 | TMEM8B |
| ENSG00000137124 | -3.679 | 9.861e-82 | 4.201e-80 | ALDH1B1 |
| ENSG00000137135 | -3.103 | 2.596e-21 | 1.593e-20 | ARHGEF39 |
| ENSG00000137177 | -1.447 | 1.122e-24 | 8.014e-24 | KIF13A |
| ENSG00000137200 | -1.463 | 3.634e-19 | 2.016e-18 | CMTR1 |
| ENSG00000137203 | -1.626 | 2.777e-13 | 1.146e-12 | TFAP2A |
| ENSG00000137218 | -1.689 | 2.397e-06 | 6.105e-06 | FRS3 |
| ENSG00000137269 | -1.857 | 1.677e-30 | 1.527e-29 | LRRC1 |
| ENSG00000137270 | 3.237 | 2.239e-09 | 7.204e-09 | GCM1 |
| ENSG00000137273 | -2.687 | 1.498e-32 | 1.497e-31 | FOXF2 |
| ENSG00000137275 | -3.477 | 9.551e-104 | 6.765e-102 | RIPK1 |
| ENSG00000137310 | -3.129 | 2.991e-34 | 3.216e-33 | TCF19 |
| ENSG00000137331 | 1.534 | 5.12e-52 | 9.733e-51 | IER3 |
| ENSG00000137337 | -1.008 | 2.292e-06 | 5.846e-06 | MDC1 |
| ENSG00000137338 | -2.52 | 4.661e-12 | 1.786e-11 | PGBD1 |
| ENSG00000137411 | -1.048 | 1.403e-06 | 3.653e-06 | VARS2 |
| ENSG00000137449 | 1.553 | 7.993e-39 | 1.023e-37 | CPEB2 |
| ENSG00000137460 | -1.409 | 3.444e-14 | 1.487e-13 | FHDC1 |
| ENSG00000137486 | -3.108 | 3.227e-44 | 4.912e-43 | ARRB1 |
| ENSG00000137492 | -1.08 | 1.914e-29 | 1.681e-28 | PRKRIR |
| ENSG00000137497 | -1.2 | 3.798e-55 | 7.822e-54 | NUMA1 |
| ENSG00000137501 | -2.76 | 8.158e-61 | 2.047e-59 | SYTL2 |
| ENSG00000137507 | 2.263 | 0.01098 | 0.0189 | LRRC32 |
| ENSG00000137513 | -1.455 | 7.078e-19 | 3.87e-18 | NARS2 |
| ENSG00000137547 | -2.101 | 1.192e-38 | 1.518e-37 | MRPL15 |
| ENSG00000137571 | 3.661 | 0.005432 | 0.009794 | SLCO5A1 |
| ENSG00000137574 | -1.29 | 6.716e-19 | 3.677e-18 | TGS1 |
| ENSG00000137575 | 1.377 | 2.426e-34 | 2.621e-33 | SDCBP |
| ENSG00000137601 | -1.267 | 2.11e-18 | 1.13e-17 | NEK1 |
| ENSG00000137628 | -1.52 | 2.098e-21 | 1.296e-20 | DDX60 |
| ENSG00000137656 | -1.417 | 4.077e-16 | 1.946e-15 | BUD13 |
| ENSG00000137700 | -1.256 | 7.752e-20 | 4.435e-19 | SLC37A4 |
| ENSG00000137745 | 2.816 | 1.547e-17 | 7.91e-17 | MMP13 |
| ENSG00000137764 | -1.192 | 2.158e-10 | 7.446e-10 | MAP2K5 |
| ENSG00000137801 | 1.144 | 7.417e-41 | 1.011e-39 | THBS1 |
| ENSG00000137804 | -1.982 | 1.912e-28 | 1.604e-27 | NUSAP1 |
| ENSG00000137807 | -1.395 | 6.934e-19 | 3.794e-18 | KIF23 |
| ENSG00000137809 | 2.436 | 0.0009713 | 0.00193 | ITGA11 |
| ENSG00000137812 | -1.967 | 5.08e-43 | 7.434e-42 | CASC5 |
| ENSG00000137825 | -3.657 | 0.000445 | 0.0009177 | ITPKA |
| ENSG00000137831 | -1.914 | 1.274e-38 | 1.617e-37 | UACA |
| ENSG00000137834 | -2.573 | 9.106e-18 | 4.72e-17 | SMAD6 |
| ENSG00000137841 | -5.814 | 9.276e-11 | 3.274e-10 | PLCB2 |
| ENSG00000137843 | -4.833 | 8.131e-89 | 4.174e-87 | PAK6 |
| ENSG00000137857 | -1.967 | 0.01241 | 0.0212 | DUOX1 |
| ENSG00000137876 | 2.328 | 3.377e-116 | 3.015e-114 | RSL24D1 |
| ENSG00000137877 | 1.259 | 0.0002784 | 0.0005874 | SPTBN5 |
| ENSG00000137936 | -1.466 | 2.477e-59 | 5.906e-58 | BCAR3 |
| ENSG00000137960 | 2.247 | 0.03096 | 0.04933 | GIPC2 |
| ENSG00000137975 | 2.076 | 0.02207 | 0.03612 | CLCA2 |
| ENSG00000138002 | -1.54 | 1.671e-21 | 1.039e-20 | IFT172 |
| ENSG00000138030 | -2.55 | 1.337e-15 | 6.204e-15 | KHK |
| ENSG00000138031 | -1.181 | 2.906e-19 | 1.618e-18 | ADCY3 |
| ENSG00000138035 | -1.001 | 1.024e-28 | 8.718e-28 | PNPT1 |
| ENSG00000138061 | -2.776 | 1.767e-160 | 3.37e-158 | CYP1B1 |
| ENSG00000138069 | 1.069 | 2.115e-37 | 2.564e-36 | RAB1A |
| ENSG00000138073 | -1.327 | 3.042e-25 | 2.232e-24 | PREB |
| ENSG00000138074 | -1.953 | 3.161e-23 | 2.111e-22 | SLC5A6 |
| ENSG00000138081 | 2.03 | 8.858e-62 | 2.277e-60 | FBXO11 |
| ENSG00000138083 | -2.202 | 0.00217 | 0.00414 | SIX3 |
| ENSG00000138100 | 1.867 | 3.184e-12 | 1.231e-11 | TRIM54 |
| ENSG00000138109 | 2.914 | 0.009505 | 0.01653 | CYP2C9 |
| ENSG00000138111 | -2.057 | 1.856e-27 | 1.491e-26 | TMEM180 |
| ENSG00000138131 | -1.381 | 0.001254 | 0.002461 | LOXL4 |
| ENSG00000138134 | -1.587 | 4.071e-11 | 1.472e-10 | STAMBPL1 |
| ENSG00000138160 | -3.003 | 3.321e-118 | 3.132e-116 | KIF11 |
| ENSG00000138166 | 2.269 | 4.907e-74 | 1.749e-72 | DUSP5 |
| ENSG00000138172 | -3.377 | 3.895e-83 | 1.739e-81 | CALHM2 |
| ENSG00000138180 | -1.502 | 1.121e-16 | 5.486e-16 | CEP55 |
| ENSG00000138182 | -1.828 | 6.188e-25 | 4.465e-24 | KIF20B |
| ENSG00000138185 | -2.736 | 1.117e-64 | 3.14e-63 | ENTPD1 |
| ENSG00000138193 | -1.958 | 2.16e-53 | 4.256e-52 | PLCE1 |
| ENSG00000138231 | -1.446 | 6.023e-36 | 6.883e-35 | DBR1 |
| ENSG00000138271 | -2.793 | 7.77e-33 | 7.843e-32 | GPR87 |
| ENSG00000138311 | -2.069 | 2.45e-19 | 1.369e-18 | ZNF365 |
| ENSG00000138316 | -2.858 | 4.275e-15 | 1.937e-14 | ADAMTS14 |
| ENSG00000138336 | -3.531 | 5.679e-10 | 1.904e-09 | TET1 |
| ENSG00000138346 | -1.729 | 5.593e-42 | 7.942e-41 | DNA2 |
| ENSG00000138375 | -1.362 | 3.596e-20 | 2.091e-19 | SMARCAL1 |
| ENSG00000138376 | -3.263 | 5.831e-127 | 6.525e-125 | BARD1 |
| ENSG00000138380 | -1.883 | 5.462e-10 | 1.833e-09 | CARF |
| ENSG00000138399 | -1.574 | 4.534e-40 | 6.026e-39 | FASTKD1 |
| ENSG00000138433 | 1.783 | 5.844e-17 | 2.906e-16 | CIR1 |
| ENSG00000138439 | -1.8 | 6.5e-14 | 2.771e-13 | FAM117B |
| ENSG00000138442 | -1.306 | 1.755e-29 | 1.542e-28 | WDR12 |
| ENSG00000138449 | -6.488 | 5.591e-32 | 5.453e-31 | SLC40A1 |
| ENSG00000138459 | -2.366 | 6.323e-58 | 1.423e-56 | SLC35A5 |
| ENSG00000138496 | -1.879 | 1.325e-36 | 1.548e-35 | PARP9 |
| ENSG00000138593 | -1.033 | 1.208e-12 | 4.793e-12 | SECISBP2L |
| ENSG00000138604 | -2.544 | 8.94e-57 | 1.939e-55 | GLCE |
| ENSG00000138613 | -1.719 | 1.854e-08 | 5.58e-08 | APH1B |
| ENSG00000138614 | -1.1 | 3.275e-13 | 1.343e-12 | VWA9 |
| ENSG00000138617 | -2.571 | 2.219e-22 | 1.425e-21 | PARP16 |
| ENSG00000138639 | -2.041 | 6.642e-05 | 0.0001493 | ARHGAP24 |
| ENSG00000138642 | -1.352 | 1.19e-12 | 4.724e-12 | HERC6 |
| ENSG00000138646 | -1.693 | 1.347e-09 | 4.412e-09 | HERC5 |
| ENSG00000138670 | 3.628 | 0.0001499 | 0.000325 | RASGEF1B |
| ENSG00000138678 | 1.525 | 1.849e-38 | 2.33e-37 | AGPAT9 |
| ENSG00000138688 | -1.073 | 5.582e-32 | 5.448e-31 | KIAA1109 |
| ENSG00000138735 | -1.349 | 2.017e-09 | 6.519e-09 | PDE5A |
| ENSG00000138744 | -1.172 | 4.105e-05 | 9.41e-05 | NAAA |
| ENSG00000138756 | 1.418 | 7.895e-09 | 2.448e-08 | BMP2K |
| ENSG00000138764 | 1.702 | 1.843e-36 | 2.139e-35 | CCNG2 |
| ENSG00000138767 | -1.055 | 1.418e-15 | 6.561e-15 | CNOT6L |
| ENSG00000138771 | -1.354 | 3.194e-48 | 5.449e-47 | SHROOM3 |
| ENSG00000138778 | -1.693 | 2.554e-16 | 1.23e-15 | CENPE |
| ENSG00000138780 | -1.094 | 2.832e-09 | 9.059e-09 | GSTCD |
| ENSG00000138794 | -1.237 | 1.69e-12 | 6.638e-12 | CASP6 |
| ENSG00000138795 | -1.476 | 0.002021 | 0.003873 | LEF1 |
| ENSG00000138798 | -1.068 | 0.002949 | 0.005512 | EGF |
| ENSG00000138829 | 2.475 | 0.001086 | 0.002147 | FBN2 |
| ENSG00000138834 | -1.115 | 3.698e-35 | 4.124e-34 | MAPK8IP3 |
| ENSG00000138835 | -4.405 | 2.702e-73 | 9.412e-72 | RGS3 |
| ENSG00000138892 | 3.031 | 0.02445 | 0.03966 | TTLL8 |
| ENSG00000138942 | 1.292 | 2.147e-11 | 7.918e-11 | RNF185 |
| ENSG00000139044 | -1.343 | 7.53e-08 | 2.171e-07 | B4GALNT3 |
| ENSG00000139112 | 2.474 | 2.123e-68 | 6.563e-67 | GABARAPL1 |
| ENSG00000139133 | -1.483 | 3.683e-13 | 1.507e-12 | ALG10 |
| ENSG00000139155 | 2.506 | 0.02515 | 0.04073 | SLCO1C1 |
| ENSG00000139160 | 1.851 | 2.282e-15 | 1.046e-14 | METTL20 |
| ENSG00000139174 | -1.779 | 5.611e-16 | 2.654e-15 | PRICKLE1 |
| ENSG00000139178 | -1.612 | 5.967e-19 | 3.276e-18 | C1RL |
| ENSG00000139194 | -1.371 | 6.866e-06 | 1.684e-05 | RBP5 |
| ENSG00000139197 | -1.292 | 6.228e-14 | 2.659e-13 | PEX5 |
| ENSG00000139200 | 3.446 | 9.304e-15 | 4.142e-14 | PIANP |
| ENSG00000139209 | 3.12 | 0.002434 | 0.004607 | SLC38A4 |
| ENSG00000139211 | -2.107 | 9.767e-43 | 1.417e-41 | AMIGO2 |
| ENSG00000139219 | 5.416 | 3.823e-15 | 1.737e-14 | COL2A1 |
| ENSG00000139263 | -3.435 | 2.351e-36 | 2.723e-35 | LRIG3 |
| ENSG00000139266 | -1.229 | 3.479e-08 | 1.028e-07 | 9-Mar |
| ENSG00000139269 | 3.296 | 5.454e-05 | 0.0001237 | INHBE |
| ENSG00000139278 | 2.129 | 1.447e-29 | 1.275e-28 | GLIPR1 |
| ENSG00000139289 | -1.136 | 5.65e-07 | 1.522e-06 | PHLDA1 |
| ENSG00000139291 | -1.909 | 4.955e-13 | 2.012e-12 | TMEM19 |
| ENSG00000139292 | 3.593 | 0.00591 | 0.01059 | LGR5 |
| ENSG00000139370 | -1.942 | 4.036e-37 | 4.843e-36 | SLC15A4 |
| ENSG00000139405 | -1.93 | 1.589e-37 | 1.937e-36 | C12orf52 |
| ENSG00000139410 | -1.474 | 0.0002025 | 0.0004332 | SDSL |
| ENSG00000139436 | -1.042 | 1.226e-10 | 4.301e-10 | GIT2 |
| ENSG00000139437 | -3.56 | 2.976e-74 | 1.07e-72 | TCHP |
| ENSG00000139514 | 1.589 | 2.129e-93 | 1.228e-91 | SLC7A1 |
| ENSG00000139531 | -3.416 | 6.229e-34 | 6.589e-33 | SUOX |
| ENSG00000139546 | -1.827 | 8.446e-08 | 2.427e-07 | TARBP2 |
| ENSG00000139547 | -2.512 | 0.007946 | 0.01399 | RDH16 |
| ENSG00000139618 | -1.563 | 2.724e-26 | 2.095e-25 | BRCA2 |
| ENSG00000139620 | -1.165 | 1.875e-11 | 6.934e-11 | KANSL2 |
| ENSG00000139629 | -3.423 | 3.547e-128 | 4.079e-126 | GALNT6 |
| ENSG00000139645 | -1.823 | 8.627e-89 | 4.415e-87 | ANKRD52 |
| ENSG00000139651 | -1.373 | 2.031e-15 | 9.343e-15 | ZNF740 |
| ENSG00000139675 | -1.315 | 5.173e-19 | 2.847e-18 | HNRNPA1L2 |
| ENSG00000139719 | -1.457 | 6.34e-26 | 4.809e-25 | VPS33A |
| ENSG00000139722 | 1.469 | 7.275e-49 | 1.271e-47 | VPS37B |
| ENSG00000139725 | 1.109 | 3.135e-07 | 8.634e-07 | RHOF |
| ENSG00000139737 | -2.178 | 3.474e-05 | 8.007e-05 | SLAIN1 |
| ENSG00000139767 | 3.098 | 0.01278 | 0.02178 | SRRM4 |
| ENSG00000139826 | -1.439 | 5.708e-27 | 4.507e-26 | ABHD13 |
| ENSG00000139832 | -3.873 | 4.877e-62 | 1.269e-60 | RAB20 |
| ENSG00000139835 | -1.157 | 2.291e-14 | 9.987e-14 | GRTP1 |
| ENSG00000139890 | 2.128 | 1.89e-07 | 5.292e-07 | REM2 |
| ENSG00000139899 | -1.326 | 0.009358 | 0.01629 | CBLN3 |
| ENSG00000139910 | -2.163 | 8.269e-10 | 2.747e-09 | NOVA1 |
| ENSG00000139988 | 2.206 | 0.003442 | 0.00637 | RDH12 |
| ENSG00000139990 | -1.101 | 1.045e-13 | 4.414e-13 | DCAF5 |
| ENSG00000139998 | -1.33 | 3.394e-59 | 8.001e-58 | RAB15 |
| ENSG00000140006 | -2.489 | 1.055e-57 | 2.361e-56 | WDR89 |
| ENSG00000140009 | -1.734 | 0.002104 | 0.004022 | ESR2 |
| ENSG00000140022 | 1.561 | 1.762e-13 | 7.354e-13 | STON2 |
| ENSG00000140104 | -3.407 | 1.649e-33 | 1.706e-32 | C14orf79 |
| ENSG00000140105 | 1.254 | 2.284e-31 | 2.17e-30 | WARS |
| ENSG00000140153 | 1.021 | 1.289e-23 | 8.777e-23 | WDR20 |
| ENSG00000140280 | -2.149 | 4.642e-13 | 1.887e-12 | LYSMD2 |
| ENSG00000140299 | 1.286 | 2.05e-38 | 2.578e-37 | BNIP2 |
| ENSG00000140323 | -3.209 | 4.671e-05 | 0.0001066 | DISP2 |
| ENSG00000140326 | -2.101 | 4.189e-26 | 3.201e-25 | CDAN1 |
| ENSG00000140368 | 1.744 | 0.001592 | 0.00309 | PSTPIP1 |
| ENSG00000140379 | 4.103 | 0.0002245 | 0.0004785 | BCL2A1 |
| ENSG00000140382 | -1.409 | 2.331e-24 | 1.642e-23 | HMG20A |
| ENSG00000140396 | -1.568 | 1.727e-26 | 1.337e-25 | NCOA2 |
| ENSG00000140398 | -1.007 | 0.0003928 | 0.0008156 | NEIL1 |
| ENSG00000140406 | -1.587 | 1.554e-17 | 7.944e-17 | MESDC1 |
| ENSG00000140450 | 1.74 | 3.414e-33 | 3.499e-32 | ARRDC4 |
| ENSG00000140451 | -6.459 | 4.314e-40 | 5.743e-39 | PIF1 |
| ENSG00000140464 | -1.347 | 3.75e-09 | 1.189e-08 | PML |
| ENSG00000140474 | -1.612 | 4.595e-16 | 2.186e-15 | ULK3 |
| ENSG00000140478 | 5.166 | 1.941e-07 | 5.431e-07 | GOLGA6D |
| ENSG00000140488 | 4.486 | 0.0005725 | 0.001168 | CELF6 |
| ENSG00000140511 | 1.006 | 0.01556 | 0.02615 | HAPLN3 |
| ENSG00000140519 | 1.573 | 5.69e-07 | 1.532e-06 | RHCG |
| ENSG00000140525 | -1.077 | 3.317e-20 | 1.931e-19 | FANCI |
| ENSG00000140534 | -1.615 | 1.042e-45 | 1.657e-44 | TICRR |
| ENSG00000140538 | 3.166 | 0.02381 | 0.03869 | NTRK3 |
| ENSG00000140548 | -1.837 | 4.336e-31 | 4.05e-30 | ZNF710 |
| ENSG00000140557 | 4.743 | 6.071e-06 | 1.496e-05 | ST8SIA2 |
| ENSG00000140563 | -3.81 | 0.0003535 | 0.0007379 | MCTP2 |
| ENSG00000140577 | -1.426 | 3.2e-34 | 3.436e-33 | CRTC3 |
| ENSG00000140598 | -1.018 | 1.597e-24 | 1.133e-23 | EFTUD1 |
| ENSG00000140600 | 2.093 | 0.01424 | 0.02409 | SH3GL3 |
| ENSG00000140650 | 1.205 | 1.395e-33 | 1.449e-32 | PMM2 |
| ENSG00000140678 | 2.831 | 7.983e-06 | 1.947e-05 | ITGAX |
| ENSG00000140682 | 2.163 | 0.02426 | 0.03938 | TGFB1I1 |
| ENSG00000140694 | -1.158 | 1.727e-19 | 9.732e-19 | PARN |
| ENSG00000140749 | 1.667 | 0.003222 | 0.005991 | IGSF6 |
| ENSG00000140798 | 4.168 | 0.000248 | 0.0005257 | ABCC12 |
| ENSG00000140832 | -1.756 | 8.201e-49 | 1.43e-47 | MARVELD3 |
| ENSG00000140835 | -4.934 | 8.986e-07 | 2.383e-06 | CHST4 |
| ENSG00000140853 | -1.191 | 2.368e-12 | 9.228e-12 | NLRC5 |
| ENSG00000140854 | -1.109 | 5.441e-14 | 2.329e-13 | KATNB1 |
| ENSG00000140873 | 4.388 | 3.467e-05 | 7.993e-05 | ADAMTS18 |
| ENSG00000140876 | -1.714 | 0.01564 | 0.02628 | NUDT7 |
| ENSG00000140941 | 3.892 | 0 | 0 | MAP1LC3B |
| ENSG00000140943 | -1.002 | 7.242e-21 | 4.351e-20 | MBTPS1 |
| ENSG00000140950 | -1.528 | 2.182e-12 | 8.514e-12 | TLDC1 |
| ENSG00000140961 | 2.575 | 1.112e-113 | 9.424e-112 | OSGIN1 |
| ENSG00000140995 | -1.499 | 1.019e-14 | 4.529e-14 | DEF8 |
| ENSG00000141068 | -1.265 | 2.124e-12 | 8.292e-12 | KSR1 |
| ENSG00000141127 | -1.199 | 8.883e-15 | 3.965e-14 | PRPSAP2 |
| ENSG00000141140 | -1.032 | 4.881e-22 | 3.091e-21 | MYO19 |
| ENSG00000141179 | -1.271 | 1.146e-25 | 8.602e-25 | PCTP |
| ENSG00000141219 | -1.513 | 2.27e-21 | 1.399e-20 | C17orf80 |
| ENSG00000141258 | -1.481 | 2.05e-41 | 2.86e-40 | SGSM2 |
| ENSG00000141295 | -1.402 | 1.192e-07 | 3.395e-07 | SCRN2 |
| ENSG00000141337 | -1.539 | 8.459e-09 | 2.615e-08 | ARSG |
| ENSG00000141380 | -1.298 | 6.806e-12 | 2.578e-11 | SS18 |
| ENSG00000141385 | -1.486 | 6.505e-45 | 1.007e-43 | AFG3L2 |
| ENSG00000141401 | -1.15 | 2.814e-13 | 1.16e-12 | IMPA2 |
| ENSG00000141428 | 1.274 | 6.86e-18 | 3.577e-17 | C18orf21 |
| ENSG00000141441 | -1.502 | 1.991e-13 | 8.284e-13 | GAREM |
| ENSG00000141449 | -1.732 | 2.998e-24 | 2.101e-23 | GREB1L |
| ENSG00000141456 | -1.529 | 2.7e-40 | 3.606e-39 | PELP1 |
| ENSG00000141458 | 2.387 | 4.63e-163 | 8.934e-161 | NPC1 |
| ENSG00000141485 | 2.921 | 1.124e-06 | 2.955e-06 | SLC13A5 |
| ENSG00000141499 | -1.19 | 3.217e-13 | 1.32e-12 | WRAP53 |
| ENSG00000141503 | -2.216 | 3.134e-104 | 2.229e-102 | MINK1 |
| ENSG00000141505 | 3.082 | 6.353e-21 | 3.83e-20 | ASGR1 |
| ENSG00000141510 | -1.506 | 4.391e-71 | 1.445e-69 | TP53 |
| ENSG00000141522 | -1.338 | 3.917e-59 | 9.195e-58 | ARHGDIA |
| ENSG00000141526 | -1.738 | 3.896e-14 | 1.678e-13 | SLC16A3 |
| ENSG00000141527 | -1.556 | 1.582e-10 | 5.502e-10 | CARD14 |
| ENSG00000141540 | -2.088 | 0.0005523 | 0.001128 | TTYH2 |
| ENSG00000141542 | -1.348 | 4.084e-07 | 1.113e-06 | RAB40B |
| ENSG00000141556 | -1.232 | 2.676e-22 | 1.715e-21 | TBCD |
| ENSG00000141560 | -2.239 | 2.317e-24 | 1.633e-23 | FN3KRP |
| ENSG00000141569 | -3.149 | 3.197e-67 | 9.633e-66 | TRIM65 |
| ENSG00000141570 | -2.154 | 1.482e-17 | 7.588e-17 | CBX8 |
| ENSG00000141574 | -1.102 | 5.964e-07 | 1.603e-06 | SECTM1 |
| ENSG00000141577 | -2.383 | 1.498e-39 | 1.959e-38 | AZI1 |
| ENSG00000141579 | -1.952 | 9.388e-06 | 2.276e-05 | ZNF750 |
| ENSG00000141622 | -2.496 | 2.197e-17 | 1.116e-16 | RNF165 |
| ENSG00000141642 | -3.619 | 1.554e-25 | 1.161e-24 | ELAC1 |
| ENSG00000141644 | 1.785 | 4.887e-58 | 1.107e-56 | MBD1 |
| ENSG00000141655 | -1.256 | 2.972e-17 | 1.501e-16 | TNFRSF11A |
| ENSG00000141664 | 1.055 | 8.36e-20 | 4.778e-19 | ZCCHC2 |
| ENSG00000141682 | 3.211 | 8.176e-109 | 6.267e-107 | PMAIP1 |
| ENSG00000141696 | -3.143 | 5.866e-106 | 4.263e-104 | LEPREL4 |
| ENSG00000141720 | -1.815 | 1.92e-28 | 1.61e-27 | PIP4K2B |
| ENSG00000141736 | -2.797 | 1.163e-104 | 8.345e-103 | ERBB2 |
| ENSG00000141753 | -1.447 | 1.257e-17 | 6.465e-17 | IGFBP4 |
| ENSG00000141837 | -1.924 | 0.005808 | 0.01042 | CACNA1A |
| ENSG00000141854 | -3.659 | 1.711e-10 | 5.935e-10 | hsa-mir-1199 |
| ENSG00000141858 | -1.845 | 5.592e-25 | 4.048e-24 | SAMD1 |
| ENSG00000141867 | 1.755 | 1.097e-16 | 5.375e-16 | BRD4 |
| ENSG00000141905 | -2.064 | 3.053e-31 | 2.872e-30 | NFIC |
| ENSG00000141956 | -1.491 | 6.101e-23 | 4.006e-22 | PRDM15 |
| ENSG00000142046 | -1.193 | 0.0006895 | 0.001392 | TMEM91 |
| ENSG00000142065 | -2.149 | 2.449e-10 | 8.421e-10 | ZFP14 |
| ENSG00000142082 | -2.134 | 1.944e-31 | 1.854e-30 | SIRT3 |
| ENSG00000142149 | -2.916 | 1.823e-06 | 4.696e-06 | HUNK |
| ENSG00000142178 | 1.362 | 1.313e-23 | 8.93e-23 | SIK1 |
| ENSG00000142182 | 2.947 | 0.001872 | 0.003602 | DNMT3L |
| ENSG00000142188 | -1.641 | 1.884e-24 | 1.334e-23 | TMEM50B |
| ENSG00000142252 | -3.131 | 4.051e-37 | 4.857e-36 | GEMIN7 |
| ENSG00000142327 | -1.506 | 1.058e-31 | 1.021e-30 | RNPEPL1 |
| ENSG00000142405 | 2.986 | 0.0115 | 0.01974 | NLRP12 |
| ENSG00000142408 | -1.48 | 1.024e-16 | 5.021e-16 | CACNG8 |
| ENSG00000142409 | -1.05 | 2.802e-15 | 1.28e-14 | ZNF787 |
| ENSG00000142444 | -1.166 | 1.45e-05 | 3.456e-05 | C19orf52 |
| ENSG00000142459 | -1.384 | 7.214e-13 | 2.899e-12 | EVI5L |
| ENSG00000142511 | 3.163 | 0.004894 | 0.008871 | GPR32 |
| ENSG00000142513 | 2.977 | 1.624e-06 | 4.203e-06 | ACPT |
| ENSG00000142549 | -1.051 | 0.006614 | 0.01178 | IGLON5 |
| ENSG00000142611 | 4.191 | 1.667e-11 | 6.18e-11 | PRDM16 |
| ENSG00000142619 | -1.376 | 1.221e-06 | 3.199e-06 | PADI3 |
| ENSG00000142621 | 1.023 | 1.584e-05 | 3.761e-05 | FHAD1 |
| ENSG00000142623 | -1.246 | 9.755e-09 | 2.999e-08 | PADI1 |
| ENSG00000142632 | -3.773 | 1.215e-46 | 1.996e-45 | ARHGEF19 |
| ENSG00000142655 | -1.358 | 3.511e-17 | 1.767e-16 | PEX14 |
| ENSG00000142661 | 3.057 | 0.02362 | 0.03841 | MYOM3 |
| ENSG00000142677 | -2.648 | 3.825e-06 | 9.586e-06 | IL22RA1 |
| ENSG00000142686 | -3.088 | 2.836e-14 | 1.232e-13 | C1orf216 |
| ENSG00000142687 | -1.257 | 1.378e-35 | 1.56e-34 | KIAA0319L |
| ENSG00000142694 | -1.189 | 0.01235 | 0.0211 | EVA1B |
| ENSG00000142733 | -2.699 | 2.246e-41 | 3.129e-40 | MAP3K6 |
| ENSG00000142765 | -1.205 | 0.003262 | 0.006058 | SYTL1 |
| ENSG00000142794 | -1.092 | 2.199e-20 | 1.291e-19 | NBPF3 |
| ENSG00000142798 | -1.263 | 9.232e-42 | 1.302e-40 | HSPG2 |
| ENSG00000142945 | -2.87 | 1.133e-82 | 4.98e-81 | KIF2C |
| ENSG00000142959 | 2.654 | 0.0002577 | 0.0005451 | BEST4 |
| ENSG00000143061 | -2.851 | 1.458e-151 | 2.431e-149 | IGSF3 |
| ENSG00000143079 | 1.143 | 1.261e-15 | 5.859e-15 | CTTNBP2NL |
| ENSG00000143107 | 2.874 | 0.01563 | 0.02626 | FNDC7 |
| ENSG00000143119 | 4.311 | 1.562e-12 | 6.155e-12 | CD53 |
| ENSG00000143126 | -2.051 | 4.219e-39 | 5.439e-38 | CELSR2 |
| ENSG00000143140 | -4.901 | 9.567e-84 | 4.388e-82 | GJA5 |
| ENSG00000143147 | -2.548 | 3.911e-11 | 1.417e-10 | GPR161 |
| ENSG00000143149 | -1.398 | 1.069e-23 | 7.31e-23 | ALDH9A1 |
| ENSG00000143157 | -3.652 | 8.054e-200 | 2.939e-197 | POGK |
| ENSG00000143178 | -1.445 | 5.353e-07 | 1.445e-06 | TBX19 |
| ENSG00000143179 | -1.443 | 1.335e-38 | 1.691e-37 | UCK2 |
| ENSG00000143217 | -1.147 | 1.644e-07 | 4.627e-07 | PVRL4 |
| ENSG00000143228 | -2.258 | 4.039e-43 | 5.953e-42 | NUF2 |
| ENSG00000143256 | 1.16 | 9.325e-18 | 4.833e-17 | PFDN2 |
| ENSG00000143258 | -1.194 | 1.133e-19 | 6.44e-19 | USP21 |
| ENSG00000143303 | -1.477 | 1.531e-10 | 5.334e-10 | RRNAD1 |
| ENSG00000143315 | -1.814 | 2.832e-31 | 2.675e-30 | PIGM |
| ENSG00000143318 | 2.057 | 0.02092 | 0.03436 | CASQ1 |
| ENSG00000143322 | 3.301 | 0 | 0 | ABL2 |
| ENSG00000143355 | -2.696 | 0.003185 | 0.005927 | LHX9 |
| ENSG00000143363 | -1.638 | 4.499e-34 | 4.779e-33 | PRUNE |
| ENSG00000143367 | 1.399 | 3.814e-52 | 7.276e-51 | TUFT1 |
| ENSG00000143374 | -2.854 | 6.224e-70 | 1.998e-68 | TARS2 |
| ENSG00000143375 | -4.212 | 4.457e-169 | 9.47e-167 | CGN |
| ENSG00000143376 | -1.114 | 2.281e-32 | 2.263e-31 | SNX27 |
| ENSG00000143398 | 1.148 | 3.154e-46 | 5.101e-45 | PIP5K1A |
| ENSG00000143401 | -1.382 | 3.917e-53 | 7.646e-52 | ANP32E |
| ENSG00000143409 | -2.783 | 3.575e-24 | 2.495e-23 | FAM63A |
| ENSG00000143443 | -1.425 | 0.001348 | 0.002637 | C1orf56 |
| ENSG00000143457 | -1.502 | 8.451e-34 | 8.866e-33 | GOLPH3L |
| ENSG00000143466 | -1.349 | 3.486e-12 | 1.342e-11 | IKBKE |
| ENSG00000143476 | -2.303 | 3.182e-91 | 1.734e-89 | DTL |
| ENSG00000143493 | -1.223 | 6.184e-32 | 6.01e-31 | INTS7 |
| ENSG00000143499 | -1.006 | 3.77e-09 | 1.195e-08 | SMYD2 |
| ENSG00000143502 | 1.514 | 1.304e-07 | 3.703e-07 | SUSD4 |
| ENSG00000143507 | 1.407 | 1.414e-15 | 6.548e-15 | DUSP10 |
| ENSG00000143515 | 2.642 | 2.057e-05 | 4.836e-05 | ATP8B2 |
| ENSG00000143553 | -1.157 | 2.093e-16 | 1.012e-15 | SNAPIN |
| ENSG00000143578 | -1.165 | 2.844e-08 | 8.459e-08 | CREB3L4 |
| ENSG00000143590 | -2.157 | 1.524e-08 | 4.612e-08 | EFNA3 |
| ENSG00000143603 | 3.189 | 0.00114 | 0.002248 | KCNN3 |
| ENSG00000143622 | 2.228 | 1.86e-79 | 7.434e-78 | RIT1 |
| ENSG00000143627 | 3.474 | 0.002904 | 0.005431 | PKLR |
| ENSG00000143630 | -2.925 | 5.846e-32 | 5.688e-31 | HCN3 |
| ENSG00000143631 | 1.792 | 0.001805 | 0.003479 | FLG |
| ENSG00000143632 | 2.969 | 8.135e-05 | 0.0001812 | ACTA1 |
| ENSG00000143643 | -1.374 | 4.027e-31 | 3.77e-30 | TTC13 |
| ENSG00000143674 | -1.702 | 1.768e-38 | 2.229e-37 | MLK4 |
| ENSG00000143702 | -1.578 | 1.03e-26 | 8.04e-26 | CEP170 |
| ENSG00000143740 | -1.803 | 9.414e-36 | 1.07e-34 | SNAP47 |
| ENSG00000143751 | 2.014 | 3.348e-100 | 2.195e-98 | SDE2 |
| ENSG00000143753 | -2.002 | 9.588e-50 | 1.712e-48 | DEGS1 |
| ENSG00000143761 | 1.007 | 1.916e-45 | 3.028e-44 | ARF1 |
| ENSG00000143772 | -3.078 | 1.398e-73 | 4.921e-72 | ITPKB |
| ENSG00000143776 | 1.042 | 5.452e-22 | 3.444e-21 | CDC42BPA |
| ENSG00000143786 | 1.157 | 0.002541 | 0.004799 | CNIH3 |
| ENSG00000143793 | -1.819 | 7.604e-20 | 4.355e-19 | C1orf35 |
| ENSG00000143799 | -1.046 | 3.077e-31 | 2.892e-30 | PARP1 |
| ENSG00000143801 | -2.442 | 3.636e-27 | 2.894e-26 | PSEN2 |
| ENSG00000143811 | -1.086 | 1.401e-14 | 6.168e-14 | PYCR2 |
| ENSG00000143816 | -2.618 | 9.764e-24 | 6.695e-23 | WNT9A |
| ENSG00000143842 | -2.588 | 6.503e-80 | 2.656e-78 | SOX13 |
| ENSG00000143858 | -1.166 | 0.01625 | 0.02722 | SYT2 |
| ENSG00000143870 | 1.035 | 1.643e-41 | 2.3e-40 | PDIA6 |
| ENSG00000143882 | 1.031 | 0.000148 | 0.0003209 | ATP6V1C2 |
| ENSG00000143942 | -2.119 | 4.721e-28 | 3.898e-27 | CHAC2 |
| ENSG00000144026 | -2.332 | 1.642e-31 | 1.572e-30 | ZNF514 |
| ENSG00000144040 | -1.201 | 1.262e-09 | 4.148e-09 | SFXN5 |
| ENSG00000144043 | -1.001 | 8.686e-28 | 7.095e-27 | TEX261 |
| ENSG00000144045 | 1.789 | 0.0007881 | 0.00158 | DQX1 |
| ENSG00000144115 | -1.865 | 8.862e-19 | 4.827e-18 | THNSL2 |
| ENSG00000144120 | -4.124 | 2.515e-59 | 5.989e-58 | TMEM177 |
| ENSG00000144130 | 1.706 | 3.412e-06 | 8.591e-06 | NT5DC4 |
| ENSG00000144136 | 1.737 | 9.311e-45 | 1.438e-43 | SLC20A1 |
| ENSG00000144182 | -2.138 | 5.1e-18 | 2.681e-17 | LIPT1 |
| ENSG00000144191 | 3.5 | 0.003117 | 0.005809 | CNGA3 |
| ENSG00000144218 | 4.802 | 1.739e-06 | 4.492e-06 | AFF3 |
| ENSG00000144224 | 1.036 | 6.243e-39 | 8.024e-38 | UBXN4 |
| ENSG00000144230 | 3.344 | 8.926e-06 | 2.168e-05 | GPR17 |
| ENSG00000144231 | -2.233 | 9.546e-61 | 2.384e-59 | POLR2D |
| ENSG00000144283 | -1.3 | 2.22e-21 | 1.368e-20 | PKP4 |
| ENSG00000144306 | -1.497 | 1.032e-23 | 7.068e-23 | SCRN3 |
| ENSG00000144320 | -1.145 | 5.781e-12 | 2.201e-11 | KIAA1715 |
| ENSG00000144331 | -1.34 | 0.01205 | 0.02062 | ZNF385B |
| ENSG00000144354 | -3.055 | 5.364e-206 | 2.094e-203 | CDCA7 |
| ENSG00000144362 | -2.481 | 3.53e-10 | 1.201e-09 | PHOSPHO2 |
| ENSG00000144401 | -1.15 | 1.437e-09 | 4.699e-09 | METTL21A |
| ENSG00000144455 | -1.12 | 4.979e-09 | 1.566e-08 | SUMF1 |
| ENSG00000144488 | -1.735 | 0.001099 | 0.002171 | ESPNL |
| ENSG00000144504 | -1.969 | 3.294e-42 | 4.714e-41 | ANKMY1 |
| ENSG00000144524 | -1.11 | 2.054e-23 | 1.385e-22 | COPS7B |
| ENSG00000144550 | 4.145 | 0.0001418 | 0.000308 | CPNE9 |
| ENSG00000144554 | -1.052 | 3.734e-18 | 1.975e-17 | FANCD2 |
| ENSG00000144559 | -3.003 | 1.811e-20 | 1.068e-19 | TAMM41 |
| ENSG00000144566 | 1.253 | 5.058e-27 | 3.996e-26 | RAB5A |
| ENSG00000144579 | -2.436 | 9.665e-41 | 1.312e-39 | CTDSP1 |
| ENSG00000144580 | -1.475 | 5.464e-42 | 7.773e-41 | RQCD1 |
| ENSG00000144597 | 2.376 | 2.554e-142 | 3.514e-140 | EAF1 |
| ENSG00000144645 | -1.09 | 9.242e-14 | 3.916e-13 | OSBPL10 |
| ENSG00000144647 | -2.453 | 1.228e-24 | 8.765e-24 | POMGNT2 |
| ENSG00000144655 | 1.844 | 1.394e-13 | 5.85e-13 | CSRNP1 |
| ENSG00000144659 | -1.467 | 1.177e-16 | 5.758e-16 | SLC25A38 |
| ENSG00000144671 | 3.589 | 1.039e-08 | 3.185e-08 | SLC22A14 |
| ENSG00000144677 | -1.614 | 4.203e-52 | 8.008e-51 | CTDSPL |
| ENSG00000144681 | -1.478 | 1.874e-21 | 1.161e-20 | STAC |
| ENSG00000144711 | -2.065 | 2.71e-26 | 2.087e-25 | IQSEC1 |
| ENSG00000144712 | 1.424 | 0.02387 | 0.03878 | CAND2 |
| ENSG00000144744 | -1.01 | 2.263e-11 | 8.33e-11 | UBA3 |
| ENSG00000144746 | -2.154 | 5.257e-57 | 1.152e-55 | ARL6IP5 |
| ENSG00000144785 | 2.147 | 5.656e-07 | 1.523e-06 | RP11-977G19.10 |
| ENSG00000144791 | -1.563 | 4.35e-26 | 3.322e-25 | LIMD1 |
| ENSG00000144810 | -2.222 | 1.866e-21 | 1.156e-20 | COL8A1 |
| ENSG00000144815 | -1.407 | 3.639e-19 | 2.019e-18 | NXPE3 |
| ENSG00000144827 | -2.297 | 2.18e-48 | 3.733e-47 | ABHD10 |
| ENSG00000144834 | 4.717 | 6.154e-18 | 3.221e-17 | TAGLN3 |
| ENSG00000144843 | -2.712 | 1.21e-07 | 3.446e-07 | ADPRH |
| ENSG00000144895 | 1.143 | 6.074e-18 | 3.18e-17 | EIF2A |
| ENSG00000144909 | -1.342 | 1.441e-19 | 8.152e-19 | OSBPL11 |
| ENSG00000144935 | -1.176 | 0.007078 | 0.01255 | TRPC1 |
| ENSG00000144959 | -1.224 | 5.989e-26 | 4.553e-25 | NCEH1 |
| ENSG00000145002 | -1.326 | 5.732e-05 | 0.0001297 | FAM86B2 |
| ENSG00000145014 | -1.906 | 4.996e-18 | 2.627e-17 | TMEM44 |
| ENSG00000145016 | 1.221 | 4.011e-33 | 4.103e-32 | KIAA0226 |
| ENSG00000145020 | -2.507 | 2.822e-10 | 9.669e-10 | AMT |
| ENSG00000145029 | -1.305 | 6.006e-06 | 1.481e-05 | NICN1 |
| ENSG00000145050 | 1.84 | 3.594e-35 | 4.014e-34 | MANF |
| ENSG00000145087 | -4.14 | 3.784e-05 | 8.696e-05 | STXBP5L |
| ENSG00000145103 | -3.797 | 4.619e-06 | 1.15e-05 | ILDR1 |
| ENSG00000145107 | 1.321 | 6.683e-13 | 2.691e-12 | TM4SF19 |
| ENSG00000145194 | -1.689 | 6.38e-23 | 4.188e-22 | ECE2 |
| ENSG00000145198 | 4.411 | 3.075e-11 | 1.121e-10 | VWA5B2 |
| ENSG00000145214 | -1.632 | 1.328e-10 | 4.647e-10 | DGKQ |
| ENSG00000145246 | -1.994 | 5.889e-49 | 1.032e-47 | ATP10D |
| ENSG00000145284 | -1.223 | 1.96e-30 | 1.779e-29 | SCD5 |
| ENSG00000145332 | -1.18 | 5.522e-13 | 2.233e-12 | KLHL8 |
| ENSG00000145337 | -1.184 | 1.713e-15 | 7.891e-15 | PYURF |
| ENSG00000145365 | -4.458 | 7.2e-59 | 1.669e-57 | TIFA |
| ENSG00000145386 | -4.037 | 8.052e-228 | 3.755e-225 | CCNA2 |
| ENSG00000145388 | 1.078 | 9.242e-15 | 4.119e-14 | METTL14 |
| ENSG00000145390 | 1.102 | 1.108e-18 | 6.013e-18 | USP53 |
| ENSG00000145416 | -2.722 | 6.269e-32 | 6.089e-31 | 1-Mar |
| ENSG00000145431 | -1.023 | 3.411e-07 | 9.372e-07 | PDGFC |
| ENSG00000145476 | -1.297 | 1.494e-18 | 8.068e-18 | CYP4V2 |
| ENSG00000145495 | 1.091 | 5.437e-26 | 4.143e-25 | 6-Mar |
| ENSG00000145555 | 2.149 | 1.309e-100 | 8.651e-99 | MYO10 |
| ENSG00000145569 | -1.454 | 1.116e-06 | 2.937e-06 | FAM105A |
| ENSG00000145604 | -3.437 | 3.567e-239 | 1.761e-236 | SKP2 |
| ENSG00000145623 | 1.557 | 2.171e-56 | 4.655e-55 | OSMR |
| ENSG00000145675 | -1.998 | 1.646e-12 | 6.472e-12 | PIK3R1 |
| ENSG00000145687 | -1.252 | 0.0002422 | 0.0005138 | SSBP2 |
| ENSG00000145692 | 5.66 | 3.349e-09 | 1.066e-08 | BHMT |
| ENSG00000145703 | 2.543 | 3.234e-05 | 7.473e-05 | IQGAP2 |
| ENSG00000145779 | -1.445 | 2.357e-33 | 2.424e-32 | TNFAIP8 |
| ENSG00000145780 | 1.285 | 7.752e-18 | 4.027e-17 | FEM1C |
| ENSG00000145860 | -1.083 | 2.177e-15 | 9.995e-15 | RNF145 |
| ENSG00000145868 | 1.265 | 2.678e-22 | 1.716e-21 | FBXO38 |
| ENSG00000145882 | -1.691 | 1.55e-16 | 7.535e-16 | PCYOX1L |
| ENSG00000145911 | -1.84 | 1.828e-07 | 5.121e-07 | N4BP3 |
| ENSG00000145920 | 2.634 | 7.372e-05 | 0.0001649 | CPLX2 |
| ENSG00000145945 | -4.593 | 7.224e-13 | 2.902e-12 | FAM50B |
| ENSG00000145965 | -2.301 | 0.006123 | 0.01095 | C6ORF50 |
| ENSG00000145975 | 2.619 | 3.363e-07 | 9.243e-07 | FAM217A |
| ENSG00000145990 | -2.204 | 3.736e-54 | 7.501e-53 | GFOD1 |
| ENSG00000146006 | 2.496 | 3.355e-12 | 1.293e-11 | LRRTM2 |
| ENSG00000146038 | -1.835 | 0.01877 | 0.03109 | DCDC2 |
| ENSG00000146054 | -2.738 | 2.537e-18 | 1.355e-17 | TRIM7 |
| ENSG00000146063 | -1.132 | 5.241e-22 | 3.313e-21 | TRIM41 |
| ENSG00000146072 | -2.419 | 1.521e-103 | 1.064e-101 | TNFRSF21 |
| ENSG00000146083 | 1.118 | 2.876e-22 | 1.839e-21 | RNF44 |
| ENSG00000146094 | 1.556 | 5.405e-07 | 1.458e-06 | DOK3 |
| ENSG00000146166 | 5.357 | 2.093e-07 | 5.84e-07 | LGSN |
| ENSG00000146233 | 1.213 | 0.002219 | 0.004223 | CYP39A1 |
| ENSG00000146242 | -1.081 | 1.265e-14 | 5.587e-14 | TPBG |
| ENSG00000146243 | -1.904 | 1.263e-12 | 5.004e-12 | IRAK1BP1 |
| ENSG00000146263 | -1.829 | 2.082e-76 | 7.871e-75 | MMS22L |
| ENSG00000146267 | -3.475 | 1.079e-14 | 4.784e-14 | FAXC |
| ENSG00000146278 | 1.767 | 4.031e-29 | 3.495e-28 | PNRC1 |
| ENSG00000146281 | -2.213 | 8.112e-103 | 5.627e-101 | PM20D2 |
| ENSG00000146350 | -1.429 | 1.588e-12 | 6.252e-12 | TBC1D32 |
| ENSG00000146373 | -1.079 | 2.881e-24 | 2.022e-23 | RNF217 |
| ENSG00000146374 | 1.848 | 0.02519 | 0.04079 | RSPO3 |
| ENSG00000146376 | -1.311 | 1.536e-23 | 1.04e-22 | ARHGAP18 |
| ENSG00000146399 | 3.219 | 0.01705 | 0.02846 | TAAR1 |
| ENSG00000146409 | -1.172 | 4.347e-19 | 2.402e-18 | SLC18B1 |
| ENSG00000146410 | -1.089 | 4.829e-10 | 1.627e-09 | MTFR2 |
| ENSG00000146411 | -1.771 | 5.817e-05 | 0.0001315 | SLC2A12 |
| ENSG00000146414 | -1.34 | 1.068e-30 | 9.821e-30 | SHPRH |
| ENSG00000146425 | -1.075 | 5.939e-10 | 1.989e-09 | DYNLT1 |
| ENSG00000146453 | 4.863 | 1.052e-06 | 2.775e-06 | PNLDC1 |
| ENSG00000146469 | 5.897 | 1.884e-10 | 6.521e-10 | VIP |
| ENSG00000146530 | -2.225 | 1.1e-22 | 7.154e-22 | VWDE |
| ENSG00000146540 | -1.135 | 8.613e-18 | 4.468e-17 | C7orf50 |
| ENSG00000146576 | -1.399 | 2.193e-23 | 1.476e-22 | C7orf26 |
| ENSG00000146592 | 4.251 | 3.805e-86 | 1.841e-84 | CREB5 |
| ENSG00000146670 | -1.009 | 2.111e-11 | 7.793e-11 | CDCA5 |
| ENSG00000146674 | -2.655 | 2.392e-132 | 2.868e-130 | IGFBP3 |
| ENSG00000146678 | 2.214 | 0.02017 | 0.03326 | IGFBP1 |
| ENSG00000146733 | -1.739 | 5.952e-38 | 7.396e-37 | PSPH |
| ENSG00000146757 | 1.687 | 3.229e-24 | 2.261e-23 | ZNF92 |
| ENSG00000146776 | -1.125 | 2.738e-07 | 7.579e-07 | ATXN7L1 |
| ENSG00000146802 | -1.584 | 7.524e-34 | 7.929e-33 | TMEM168 |
| ENSG00000146828 | -1.924 | 1.311e-18 | 7.101e-18 | SLC12A9 |
| ENSG00000146830 | -1.582 | 5.918e-41 | 8.099e-40 | GIGYF1 |
| ENSG00000146834 | -1.856 | 7.67e-46 | 1.225e-44 | MEPCE |
| ENSG00000146856 | -1.56 | 4.163e-05 | 9.537e-05 | AGBL3 |
| ENSG00000146858 | -3.051 | 3.706e-37 | 4.45e-36 | ZC3HAV1L |
| ENSG00000146904 | -1.48 | 3.8e-16 | 1.816e-15 | EPHA1 |
| ENSG00000146950 | -2.541 | 1.243e-37 | 1.525e-36 | SHROOM2 |
| ENSG00000146966 | -1.86 | 7.7e-13 | 3.09e-12 | DENND2A |
| ENSG00000147003 | 1.085 | 4.61e-13 | 1.874e-12 | TMEM27 |
| ENSG00000147010 | 2.069 | 4.141e-34 | 4.416e-33 | SH3KBP1 |
| ENSG00000147050 | 1.648 | 2.245e-31 | 2.134e-30 | KDM6A |
| ENSG00000147059 | -1.456 | 0.003451 | 0.006385 | SPIN2A |
| ENSG00000147065 | 1.349 | 9.545e-23 | 6.222e-22 | MSN |
| ENSG00000147082 | 1.031 | 0.01314 | 0.02235 | CCNB3 |
| ENSG00000147100 | -1.154 | 0.002816 | 0.00528 | SLC16A2 |
| ENSG00000147113 | 3.815 | 0.0007938 | 0.001591 | CXorf36 |
| ENSG00000147119 | -1.691 | 0.0003058 | 0.0006424 | CHST7 |
| ENSG00000147130 | -4.183 | 8.688e-172 | 1.894e-169 | ZMYM3 |
| ENSG00000147144 | -1.712 | 1.038e-26 | 8.094e-26 | CCDC120 |
| ENSG00000147162 | 1.132 | 1.61e-58 | 3.703e-57 | OGT |
| ENSG00000147168 | 4.591 | 8.398e-06 | 2.044e-05 | IL2RG |
| ENSG00000147174 | 3.083 | 4.299e-31 | 4.018e-30 | ACRC |
| ENSG00000147206 | 3.652 | 0.0004404 | 0.0009089 | NXF3 |
| ENSG00000147224 | -1.306 | 7.425e-17 | 3.672e-16 | PRPS1 |
| ENSG00000147231 | -1.53 | 1.408e-05 | 3.358e-05 | CXorf57 |
| ENSG00000147234 | -2.717 | 0.001503 | 0.002925 | FRMPD3 |
| ENSG00000147251 | -1.656 | 7.216e-26 | 5.464e-25 | DOCK11 |
| ENSG00000147382 | -1.416 | 6.416e-14 | 2.736e-13 | FAM58A |
| ENSG00000147421 | 1.206 | 3.725e-17 | 1.872e-16 | HMBOX1 |
| ENSG00000147576 | -1.422 | 0.0006596 | 0.001336 | ADHFE1 |
| ENSG00000147614 | 1.594 | 0.0005287 | 0.001083 | ATP6V0D2 |
| ENSG00000147642 | -2.445 | 3.454e-12 | 1.33e-11 | SYBU |
| ENSG00000147654 | -1.115 | 1.218e-07 | 3.467e-07 | EBAG9 |
| ENSG00000147697 | -1.966 | 0.0001743 | 0.0003751 | GSDMC |
| ENSG00000147862 | -1.59 | 1.594e-27 | 1.285e-26 | NFIB |
| ENSG00000147872 | -1.234 | 4.275e-08 | 1.254e-07 | PLIN2 |
| ENSG00000147883 | -1.403 | 3.918e-21 | 2.386e-20 | CDKN2B |
| ENSG00000147894 | 1.675 | 2.157e-21 | 1.331e-20 | C9orf72 |
| ENSG00000147905 | 1.093 | 3.419e-08 | 1.011e-07 | ZCCHC7 |
| ENSG00000147912 | -1.041 | 3.39e-06 | 8.54e-06 | FBXO10 |
| ENSG00000147955 | -1.475 | 1.605e-25 | 1.198e-24 | SIGMAR1 |
| ENSG00000148019 | -2.106 | 9.245e-86 | 4.409e-84 | CEP78 |
| ENSG00000148057 | -1.771 | 3.724e-09 | 1.181e-08 | IDNK |
| ENSG00000148082 | -2.372 | 5.859e-21 | 3.539e-20 | SHC3 |
| ENSG00000148110 | -1.057 | 1.695e-21 | 1.052e-20 | HIATL1 |
| ENSG00000148143 | -1.598 | 3.85e-13 | 1.574e-12 | ZNF462 |
| ENSG00000148175 | -1.256 | 1.105e-12 | 4.399e-12 | STOM |
| ENSG00000148204 | 2.198 | 0.000346 | 0.0007228 | CRB2 |
| ENSG00000148218 | -2.73 | 2.819e-62 | 7.427e-61 | ALAD |
| ENSG00000148219 | -1.137 | 0.01038 | 0.01795 | ASTN2 |
| ENSG00000148225 | -2.555 | 1.112e-12 | 4.422e-12 | WDR31 |
| ENSG00000148229 | -1.213 | 2.219e-15 | 1.018e-14 | POLE3 |
| ENSG00000148296 | -1.757 | 3.943e-26 | 3.017e-25 | SURF6 |
| ENSG00000148297 | -2.331 | 2.12e-93 | 1.227e-91 | MED22 |
| ENSG00000148334 | -1.508 | 1.056e-14 | 4.686e-14 | PTGES2 |
| ENSG00000148339 | 1.547 | 4.361e-21 | 2.651e-20 | SLC25A25 |
| ENSG00000148343 | -1.549 | 1.46e-08 | 4.424e-08 | FAM73B |
| ENSG00000148344 | -1.39 | 4.349e-17 | 2.18e-16 | PTGES |
| ENSG00000148356 | -1.278 | 6.384e-15 | 2.865e-14 | LRSAM1 |
| ENSG00000148362 | -1.291 | 3.322e-07 | 9.134e-07 | C9orf142 |
| ENSG00000148384 | -2.338 | 4.326e-23 | 2.861e-22 | INPP5E |
| ENSG00000148399 | -1.449 | 7.362e-12 | 2.781e-11 | DPH7 |
| ENSG00000148400 | 1.183 | 4.941e-28 | 4.076e-27 | NOTCH1 |
| ENSG00000148408 | 3.582 | 0.00209 | 0.003998 | CACNA1B |
| ENSG00000148411 | -3.128 | 3.511e-65 | 1.004e-63 | NACC2 |
| ENSG00000148444 | -1.15 | 5.335e-15 | 2.403e-14 | COMMD3 |
| ENSG00000148459 | -1.397 | 1.959e-07 | 5.478e-07 | PDSS1 |
| ENSG00000148482 | 2.491 | 0.01845 | 0.03062 | SLC39A12 |
| ENSG00000148483 | 2.76 | 2.252e-17 | 1.142e-16 | TMEM236 |
| ENSG00000148516 | 5.309 | 1.506e-70 | 4.899e-69 | ZEB1 |
| ENSG00000148606 | -1.015 | 5.079e-12 | 1.94e-11 | POLR3A |
| ENSG00000148660 | -2.019 | 6.879e-88 | 3.447e-86 | CAMK2G |
| ENSG00000148677 | 4.075 | 0 | 0 | ANKRD1 |
| ENSG00000148680 | -1.719 | 5.709e-16 | 2.698e-15 | HTR7 |
| ENSG00000148700 | -1.07 | 6.391e-21 | 3.852e-20 | ADD3 |
| ENSG00000148730 | -1.985 | 7.479e-109 | 5.759e-107 | EIF4EBP2 |
| ENSG00000148735 | -3.638 | 5.041e-09 | 1.584e-08 | PLEKHS1 |
| ENSG00000148773 | -3.452 | 1.52e-64 | 4.253e-63 | MKI67 |
| ENSG00000148814 | -2.159 | 2.183e-09 | 7.032e-09 | LRRC27 |
| ENSG00000148824 | -1.177 | 3.903e-10 | 1.323e-09 | MTG1 |
| ENSG00000148832 | -2.193 | 6.768e-06 | 1.661e-05 | PAOX |
| ENSG00000148926 | 1.833 | 1.793e-31 | 1.712e-30 | ADM |
| ENSG00000148935 | -3.068 | 0.0001517 | 0.0003286 | GAS2 |
| ENSG00000148943 | 1.486 | 4.069e-43 | 5.992e-42 | LIN7C |
| ENSG00000148985 | -1.847 | 1.617e-36 | 1.88e-35 | PGAP2 |
| ENSG00000149021 | 2.708 | 0.003611 | 0.006666 | SCGB1A1 |
| ENSG00000149043 | -2.28 | 8.689e-06 | 2.112e-05 | SYT8 |
| ENSG00000149050 | -1.467 | 0.02426 | 0.03938 | ZNF214 |
| ENSG00000149054 | -1.726 | 6.046e-13 | 2.441e-12 | ZNF215 |
| ENSG00000149115 | -1.462 | 1.529e-23 | 1.036e-22 | TNKS1BP1 |
| ENSG00000149150 | 1.15 | 0.02728 | 0.04391 | SLC43A1 |
| ENSG00000149177 | -1.804 | 1.389e-41 | 1.949e-40 | PTPRJ |
| ENSG00000149196 | 1.152 | 1.872e-14 | 8.196e-14 | C11orf73 |
| ENSG00000149201 | 2.353 | 8.333e-17 | 4.114e-16 | CCDC81 |
| ENSG00000149212 | 2.338 | 0.009094 | 0.01586 | SESN3 |
| ENSG00000149218 | -3.264 | 1.1e-65 | 3.183e-64 | ENDOD1 |
| ENSG00000149243 | -2.815 | 1.371e-07 | 3.887e-07 | KLHL35 |
| ENSG00000149257 | 1.359 | 2.952e-29 | 2.571e-28 | SERPINH1 |
| ENSG00000149260 | -1.236 | 0.0002381 | 0.0005058 | CAPN5 |
| ENSG00000149292 | -1.719 | 7.404e-24 | 5.097e-23 | TTC12 |
| ENSG00000149300 | -1.898 | 2.052e-07 | 5.732e-07 | C11orf52 |
| ENSG00000149313 | -2.37 | 1.018e-74 | 3.699e-73 | AASDHPPT |
| ENSG00000149418 | -2.188 | 3.362e-139 | 4.444e-137 | ST14 |
| ENSG00000149428 | 1.076 | 1.289e-37 | 1.578e-36 | HYOU1 |
| ENSG00000149452 | 3.229 | 0.008785 | 0.01535 | SLC22A8 |
| ENSG00000149474 | -4.588 | 2.907e-123 | 3.069e-121 | CSRP2BP |
| ENSG00000149476 | -1.122 | 7.606e-11 | 2.701e-10 | DAK |
| ENSG00000149483 | -1.164 | 3.936e-13 | 1.608e-12 | TMEM138 |
| ENSG00000149488 | 3.893 | 1.602e-06 | 4.15e-06 | TMC2 |
| ENSG00000149499 | -1.61 | 1.524e-14 | 6.702e-14 | EML3 |
| ENSG00000149503 | -1.438 | 3.386e-41 | 4.674e-40 | INCENP |
| ENSG00000149531 | -1.424 | 4.733e-15 | 2.14e-14 | FRG1B |
| ENSG00000149541 | -1.898 | 4.251e-34 | 4.528e-33 | B3GAT3 |
| ENSG00000149548 | -1.053 | 4.265e-06 | 1.065e-05 | CCDC15 |
| ENSG00000149554 | -1.512 | 1.148e-34 | 1.255e-33 | CHEK1 |
| ENSG00000149571 | -1.444 | 0.01347 | 0.02287 | KIRREL3 |
| ENSG00000149573 | -1.1 | 1.194e-17 | 6.151e-17 | MPZL2 |
| ENSG00000149577 | 1.491 | 1.179e-35 | 1.337e-34 | SIDT2 |
| ENSG00000149582 | -1.783 | 1.153e-12 | 4.579e-12 | TMEM25 |
| ENSG00000149591 | 2.415 | 7.081e-33 | 7.182e-32 | TAGLN |
| ENSG00000149596 | -2.115 | 2.223e-16 | 1.074e-15 | JPH2 |
| ENSG00000149599 | 3.08 | 0.01228 | 0.02099 | DUSP15 |
| ENSG00000149600 | -1.077 | 2.368e-06 | 6.034e-06 | COMMD7 |
| ENSG00000149609 | 1.846 | 0.007915 | 0.01394 | C20orf144 |
| ENSG00000149633 | 1.753 | 0.0113 | 0.01943 | KIAA1755 |
| ENSG00000149634 | -1.631 | 0.03035 | 0.04842 | SPATA25 |
| ENSG00000149636 | -2.248 | 1.749e-34 | 1.903e-33 | DSN1 |
| ENSG00000149639 | -2.317 | 4.04e-88 | 2.043e-86 | SOGA1 |
| ENSG00000149646 | 1.644 | 0.01118 | 0.01923 | CNBD2 |
| ENSG00000149657 | -2.238 | 1.644e-79 | 6.586e-78 | LSM14B |
| ENSG00000149761 | -1.904 | 2.845e-24 | 1.998e-23 | NUDT22 |
| ENSG00000149781 | 4.233 | 1.21e-48 | 2.1e-47 | FERMT3 |
| ENSG00000149782 | -1.638 | 1.642e-43 | 2.451e-42 | PLCB3 |
| ENSG00000149792 | -1.373 | 6.193e-29 | 5.315e-28 | MRPL49 |
| ENSG00000149927 | -2.135 | 0.00203 | 0.003889 | DOC2A |
| ENSG00000149929 | -1.14 | 1.269e-11 | 4.734e-11 | HIRIP3 |
| ENSG00000149968 | 8.761 | 3.94e-83 | 1.754e-81 | MMP3 |
| ENSG00000149972 | 3.093 | 0.01211 | 0.02071 | CNTN5 |
| ENSG00000150337 | 3.965 | 1.147e-41 | 1.611e-40 | FCGR1A |
| ENSG00000150433 | -1.085 | 1.644e-06 | 4.254e-06 | TMEM218 |
| ENSG00000150456 | -1.807 | 7.809e-12 | 2.946e-11 | N6AMT2 |
| ENSG00000150510 | -2.439 | 0.008857 | 0.01547 | FAM124A |
| ENSG00000150627 | -1.061 | 2.042e-09 | 6.595e-09 | WDR17 |
| ENSG00000150687 | -2.582 | 2.408e-200 | 8.981e-198 | PRSS23 |
| ENSG00000150712 | -1.113 | 9.517e-31 | 8.783e-30 | MTMR12 |
| ENSG00000150893 | -3.459 | 1.09e-75 | 4.046e-74 | FREM2 |
| ENSG00000150961 | 1.575 | 1.734e-30 | 1.577e-29 | SEC24D |
| ENSG00000150967 | -1.783 | 1.993e-10 | 6.888e-10 | ABCB9 |
| ENSG00000150977 | -1.435 | 0.0006947 | 0.001401 | RILPL2 |
| ENSG00000150991 | 3.156 | 2.039e-144 | 2.877e-142 | UBC |
| ENSG00000151006 | -2.5 | 2.192e-11 | 8.075e-11 | PRSS53 |
| ENSG00000151012 | 2.707 | 6.63e-268 | 3.837e-265 | SLC7A11 |
| ENSG00000151014 | 1.624 | 1.325e-23 | 9.007e-23 | CCRN4L |
| ENSG00000151062 | 2.328 | 1.027e-08 | 3.149e-08 | CACNA2D4 |
| ENSG00000151065 | -4.082 | 4.643e-15 | 2.101e-14 | DCP1B |
| ENSG00000151117 | -1.466 | 0.001376 | 0.002689 | TMEM86A |
| ENSG00000151150 | -1.274 | 3.741e-10 | 1.269e-09 | ANK3 |
| ENSG00000151208 | -1.106 | 5.287e-37 | 6.303e-36 | DLG5 |
| ENSG00000151224 | 2.724 | 0.008991 | 0.01569 | MAT1A |
| ENSG00000151229 | -1.119 | 0.004379 | 0.007988 | SLC2A13 |
| ENSG00000151233 | -1.581 | 3.425e-46 | 5.533e-45 | GXYLT1 |
| ENSG00000151287 | -1.577 | 3.159e-17 | 1.592e-16 | TEX30 |
| ENSG00000151353 | -1.014 | 1.685e-06 | 4.356e-06 | TMEM18 |
| ENSG00000151376 | -1.123 | 6.385e-17 | 3.17e-16 | ME3 |
| ENSG00000151388 | -1.293 | 1.966e-39 | 2.553e-38 | ADAMTS12 |
| ENSG00000151466 | -1.818 | 2.829e-22 | 1.81e-21 | SCLT1 |
| ENSG00000151475 | 3.171 | 0.02461 | 0.0399 | SLC25A31 |
| ENSG00000151498 | -1.916 | 4.014e-34 | 4.289e-33 | ACAD8 |
| ENSG00000151500 | -1.577 | 4.566e-18 | 2.405e-17 | THYN1 |
| ENSG00000151502 | -1.08 | 1.81e-19 | 1.019e-18 | VPS26B |
| ENSG00000151503 | -1.741 | 4.38e-64 | 1.207e-62 | NCAPD3 |
| ENSG00000151552 | -1.401 | 8.52e-12 | 3.207e-11 | QDPR |
| ENSG00000151611 | -1.843 | 1.479e-09 | 4.827e-09 | MMAA |
| ENSG00000151612 | -2.487 | 7.786e-41 | 1.06e-39 | ZNF827 |
| ENSG00000151692 | -1.116 | 0.0001275 | 0.0002783 | RNF144A |
| ENSG00000151693 | 1.162 | 3.603e-19 | 2e-18 | ASAP2 |
| ENSG00000151694 | 1.479 | 3.963e-83 | 1.76e-81 | ADAM17 |
| ENSG00000151726 | -1.448 | 6.646e-17 | 3.297e-16 | ACSL1 |
| ENSG00000151729 | -1.185 | 5.362e-21 | 3.247e-20 | SLC25A4 |
| ENSG00000151743 | 1.275 | 3.725e-11 | 1.352e-10 | AMN1 |
| ENSG00000151812 | 2.361 | 0.005125 | 0.00927 | SLC35F4 |
| ENSG00000151835 | -2.501 | 5.133e-65 | 1.453e-63 | SACS |
| ENSG00000151846 | 1.507 | 0.002059 | 0.003941 | PABPC3 |
| ENSG00000151876 | -1.692 | 4.198e-22 | 2.668e-21 | FBXO4 |
| ENSG00000151882 | -2.086 | 1.079e-14 | 4.785e-14 | CCL28 |
| ENSG00000151883 | -1.656 | 0.0001177 | 0.0002581 | PARP8 |
| ENSG00000151914 | 1.072 | 3.571e-22 | 2.276e-21 | DST |
| ENSG00000151929 | 3.409 | 6.816e-119 | 6.614e-117 | BAG3 |
| ENSG00000151967 | 1.513 | 1.003e-06 | 2.651e-06 | SCHIP1 |
| ENSG00000152049 | 4.137 | 1.957e-06 | 5.025e-06 | KCNE4 |
| ENSG00000152056 | -1.672 | 1.464e-36 | 1.707e-35 | AP1S3 |
| ENSG00000152127 | -1.461 | 4.099e-41 | 5.649e-40 | MGAT5 |
| ENSG00000152137 | 4.046 | 0 | 0 | HSPB8 |
| ENSG00000152147 | -1.788 | 1.913e-25 | 1.42e-24 | GEMIN6 |
| ENSG00000152193 | -1.339 | 1.466e-27 | 1.184e-26 | RNF219 |
| ENSG00000152213 | -2.1 | 3.212e-08 | 9.518e-08 | ARL11 |
| ENSG00000152217 | -1.975 | 7.045e-24 | 4.855e-23 | SETBP1 |
| ENSG00000152219 | -1.273 | 7.44e-26 | 5.631e-25 | ARL14EP |
| ENSG00000152253 | -1.499 | 1.798e-18 | 9.659e-18 | SPC25 |
| ENSG00000152270 | -3.189 | 0.0002919 | 0.0006145 | PDE3B |
| ENSG00000152284 | -2.434 | 4.34e-21 | 2.639e-20 | TCF7L1 |
| ENSG00000152348 | -1.489 | 1.641e-09 | 5.342e-09 | ATG10 |
| ENSG00000152359 | -2.083 | 4.17e-34 | 4.445e-33 | POC5 |
| ENSG00000152382 | -2.305 | 3.093e-27 | 2.471e-26 | TADA1 |
| ENSG00000152433 | 1.87 | 4.044e-10 | 1.369e-09 | ZNF547 |
| ENSG00000152439 | -1.153 | 3.777e-08 | 1.113e-07 | ZNF773 |
| ENSG00000152463 | 4.504 | 2.94e-08 | 8.733e-08 | OLAH |
| ENSG00000152503 | 1.179 | 0.01594 | 0.02674 | TRIM36 |
| ENSG00000152518 | -3.582 | 1.248e-149 | 1.996e-147 | ZFP36L2 |
| ENSG00000152582 | -2.393 | 1.695e-12 | 6.655e-12 | SPEF2 |
| ENSG00000152620 | -2.211 | 7.426e-61 | 1.866e-59 | NADK2 |
| ENSG00000152642 | -1.244 | 1.454e-19 | 8.222e-19 | GPD1L |
| ENSG00000152669 | -3.303 | 4.981e-20 | 2.876e-19 | CCNO |
| ENSG00000152689 | 1.36 | 0.004283 | 0.007829 | RASGRP3 |
| ENSG00000152749 | -2.718 | 3.171e-73 | 1.102e-71 | GPR180 |
| ENSG00000152766 | -1.965 | 3.177e-12 | 1.228e-11 | ANKRD22 |
| ENSG00000152778 | -1.927 | 1.558e-19 | 8.793e-19 | IFIT5 |
| ENSG00000152782 | -3.974 | 4.281e-87 | 2.126e-85 | PANK1 |
| ENSG00000152785 | -4.132 | 4.432e-07 | 1.205e-06 | BMP3 |
| ENSG00000152804 | -3.149 | 3.09e-29 | 2.686e-28 | HHEX |
| ENSG00000152822 | 2.274 | 0.005143 | 0.009301 | GRM1 |
| ENSG00000152894 | 1.215 | 1.897e-31 | 1.81e-30 | PTPRK |
| ENSG00000152939 | -2.557 | 4.847e-27 | 3.833e-26 | MARVELD2 |
| ENSG00000152952 | 2.399 | 4.587e-207 | 1.833e-204 | PLOD2 |
| ENSG00000152953 | 3.155 | 0.02047 | 0.03371 | STK32B |
| ENSG00000152990 | -1.749 | 2.932e-51 | 5.445e-50 | GPR125 |
| ENSG00000153006 | 1.829 | 5.144e-68 | 1.582e-66 | SREK1IP1 |
| ENSG00000153015 | 1.009 | 5.136e-08 | 1.498e-07 | CWC27 |
| ENSG00000153029 | -1.185 | 1.809e-14 | 7.932e-14 | MR1 |
| ENSG00000153048 | -1.293 | 1.315e-17 | 6.749e-17 | CARHSP1 |
| ENSG00000153064 | -1.078 | 0.00112 | 0.002209 | BANK1 |
| ENSG00000153071 | -1.459 | 7.157e-10 | 2.385e-09 | DAB2 |
| ENSG00000153086 | 1.759 | 0.01228 | 0.02099 | ACMSD |
| ENSG00000153093 | -1.071 | 2.697e-05 | 6.269e-05 | ACOXL |
| ENSG00000153094 | -2.439 | 9.597e-67 | 2.861e-65 | BCL2L11 |
| ENSG00000153107 | -1.544 | 1.257e-54 | 2.559e-53 | ANAPC1 |
| ENSG00000153140 | -1.344 | 8.894e-20 | 5.078e-19 | CETN3 |
| ENSG00000153162 | 2.119 | 4.568e-30 | 4.083e-29 | BMP6 |
| ENSG00000153165 | 1.425 | 9.636e-05 | 0.0002134 | RGPD3 |
| ENSG00000153179 | -1.533 | 3.134e-22 | 2.002e-21 | RASSF3 |
| ENSG00000153233 | -2.336 | 2.434e-44 | 3.718e-43 | PTPRR |
| ENSG00000153234 | 3.028 | 6.979e-23 | 4.574e-22 | NR4A2 |
| ENSG00000153292 | -2.356 | 2.273e-58 | 5.206e-57 | GPR110 |
| ENSG00000153294 | -1.239 | 6.517e-11 | 2.327e-10 | GPR115 |
| ENSG00000153303 | 3.193 | 0.003278 | 0.006084 | FRMD1 |
| ENSG00000153395 | -2.764 | 2.425e-201 | 9.25299999999999e-199 | LPCAT1 |
| ENSG00000153404 | 1.103 | 0.01941 | 0.03209 | PLEKHG4B |
| ENSG00000153406 | -1.728 | 1.218e-19 | 6.915e-19 | NMRAL1 |
| ENSG00000153551 | -1.635 | 2.586e-12 | 1.004e-11 | CMTM7 |
| ENSG00000153575 | -3.533 | 7.201e-72 | 2.413e-70 | TUBGCP5 |
| ENSG00000153714 | -1.25 | 0.01878 | 0.0311 | LURAP1L |
| ENSG00000153721 | -1.974 | 1.433e-17 | 7.339e-17 | CNKSR3 |
| ENSG00000153790 | -1.124 | 0.01387 | 0.02351 | C7orf31 |
| ENSG00000153815 | 1.412 | 6.669e-12 | 2.527e-11 | CMIP |
| ENSG00000153896 | -2.735 | 1.066e-11 | 3.997e-11 | ZNF599 |
| ENSG00000153898 | -1.426 | 2.259e-08 | 6.764e-08 | MCOLN2 |
| ENSG00000153902 | 1.758 | 3.675e-05 | 8.454e-05 | LGI4 |
| ENSG00000153904 | -1.047 | 3.683e-13 | 1.507e-12 | DDAH1 |
| ENSG00000153956 | -1.058 | 0.001369 | 0.002676 | CACNA2D1 |
| ENSG00000153975 | 1.119 | 1.785e-19 | 1.005e-18 | ZUFSP |
| ENSG00000153982 | -1.514 | 0.007892 | 0.01391 | GDPD1 |
| ENSG00000153993 | -2.543 | 3.712e-05 | 8.534e-05 | SEMA3D |
| ENSG00000154025 | 5.687 | 1.721e-09 | 5.586e-09 | SLC5A10 |
| ENSG00000154035 | 1.248 | 5.531e-08 | 1.61e-07 | C17orf103 |
| ENSG00000154059 | -1.266 | 2.469e-26 | 1.904e-25 | IMPACT |
| ENSG00000154065 | -1.335 | 0.0004616 | 0.00095 | ANKRD29 |
| ENSG00000154079 | -1.62 | 1.449e-06 | 3.771e-06 | C6orf57 |
| ENSG00000154099 | 1.736 | 0.0009366 | 0.001864 | DNAAF1 |
| ENSG00000154102 | -2.429 | 1.08e-11 | 4.047e-11 | C16orf74 |
| ENSG00000154118 | 3.679 | 4.024e-18 | 2.125e-17 | JPH3 |
| ENSG00000154133 | 3.384 | 0.01041 | 0.01799 | ROBO4 |
| ENSG00000154146 | -1.302 | 0.001202 | 0.002364 | NRGN |
| ENSG00000154175 | 1.131 | 0.01015 | 0.01758 | ABI3BP |
| ENSG00000154188 | -1.802 | 2.414e-05 | 5.644e-05 | ANGPT1 |
| ENSG00000154237 | -1.598 | 2.514e-10 | 8.639e-10 | LRRK1 |
| ENSG00000154263 | 2.177 | 0.002663 | 0.005016 | ABCA10 |
| ENSG00000154274 | -1.334 | 2.88e-13 | 1.186e-12 | C4orf19 |
| ENSG00000154309 | -1.015 | 4.712e-08 | 1.378e-07 | DISP1 |
| ENSG00000154328 | -1.776 | 2.878e-10 | 9.854e-10 | NEIL2 |
| ENSG00000154358 | -1.509 | 2.592e-25 | 1.909e-24 | OBSCN |
| ENSG00000154370 | 1.995 | 1.456e-32 | 1.456e-31 | TRIM11 |
| ENSG00000154429 | -2.112 | 2.555e-52 | 4.896e-51 | CCSAP |
| ENSG00000154479 | -1.801 | 0.0006235 | 0.001267 | CCDC173 |
| ENSG00000154493 | 1.86 | 0.00397 | 0.007284 | C10orf90 |
| ENSG00000154511 | -2.38 | 5.642e-24 | 3.907e-23 | FAM69A |
| ENSG00000154548 | 2.971 | 0.0183 | 0.03039 | SRSF12 |
| ENSG00000154589 | 1.719 | 0.005348 | 0.009652 | LY96 |
| ENSG00000154710 | 1.501 | 1.121e-33 | 1.169e-32 | RABGEF1 |
| ENSG00000154719 | -1.154 | 1.684e-13 | 7.045e-13 | MRPL39 |
| ENSG00000154734 | 3.616 | 0.002806 | 0.005263 | ADAMTS1 |
| ENSG00000154743 | -2.143 | 1.891e-39 | 2.463e-38 | TSEN2 |
| ENSG00000154760 | -3.282 | 1.448e-77 | 5.667e-76 | SLFN13 |
| ENSG00000154764 | -1.163 | 1.771e-09 | 5.743e-09 | WNT7A |
| ENSG00000154781 | 1.203 | 1.11e-12 | 4.415e-12 | CCDC174 |
| ENSG00000154813 | 1.075 | 1.933e-16 | 9.351e-16 | DPH3 |
| ENSG00000154822 | -2.342 | 4.391e-09 | 1.385e-08 | PLCL2 |
| ENSG00000154889 | -2.539 | 1.496e-19 | 8.452e-19 | MPPE1 |
| ENSG00000154917 | -1.388 | 1.48e-06 | 3.847e-06 | RAB6B |
| ENSG00000154920 | -1.506 | 2.917e-18 | 1.552e-17 | EME1 |
| ENSG00000154930 | -2.744 | 6.429e-12 | 2.438e-11 | ACSS1 |
| ENSG00000154945 | -1.307 | 2.954e-15 | 1.347e-14 | ANKRD40 |
| ENSG00000154957 | -1.254 | 2.177e-05 | 5.109e-05 | ZNF18 |
| ENSG00000154978 | -1.077 | 7.063e-11 | 2.514e-10 | VOPP1 |
| ENSG00000155016 | -1.013 | 0.002103 | 0.00402 | CYP2U1 |
| ENSG00000155026 | 2.323 | 0.0004317 | 0.0008922 | RSPH10B |
| ENSG00000155085 | 1.458 | 9.863e-07 | 2.608e-06 | AK9 |
| ENSG00000155096 | 1.7 | 1.513e-59 | 3.65e-58 | AZIN1 |
| ENSG00000155130 | -1.18 | 6.312e-38 | 7.814e-37 | MARCKS |
| ENSG00000155189 | -1.532 | 3.071e-13 | 1.262e-12 | AGPAT5 |
| ENSG00000155254 | -4.766 | 1.11e-184 | 2.866e-182 | MARVELD1 |
| ENSG00000155265 | -1.056 | 5.489e-07 | 1.48e-06 | GOLGA7B |
| ENSG00000155269 | -1.347 | 3.168e-23 | 2.114e-22 | GPR78 |
| ENSG00000155275 | 2.059 | 1.179e-40 | 1.593e-39 | TRMT44 |
| ENSG00000155287 | -1.318 | 2.492e-11 | 9.153e-11 | SLC25A28 |
| ENSG00000155304 | 1.557 | 4.475e-29 | 3.874e-28 | HSPA13 |
| ENSG00000155324 | -1.708 | 1.687e-39 | 2.202e-38 | GRAMD3 |
| ENSG00000155363 | -1.779 | 4.195e-65 | 1.192e-63 | MOV10 |
| ENSG00000155393 | -2.058 | 1.773e-72 | 6.035e-71 | HEATR3 |
| ENSG00000155629 | -2.069 | 3.479e-09 | 1.106e-08 | PIK3AP1 |
| ENSG00000155636 | -1.813 | 3.314e-25 | 2.426e-24 | RBM45 |
| ENSG00000155666 | -2.48 | 2.136e-09 | 6.891e-09 | KDM8 |
| ENSG00000155719 | 4.249 | 0.0001315 | 0.0002868 | OTOA |
| ENSG00000155729 | -1.336 | 9.316e-23 | 6.078e-22 | KCTD18 |
| ENSG00000155755 | -1.31 | 1.848e-25 | 1.375e-24 | TMEM237 |
| ENSG00000155760 | -1.605 | 1.428e-12 | 5.642e-12 | FZD7 |
| ENSG00000155761 | 1.694 | 8.108e-05 | 0.0001806 | SPAG17 |
| ENSG00000155816 | 2.897 | 0.001054 | 0.002086 | FMN2 |
| ENSG00000155846 | -4.612 | 5.923e-91 | 3.186e-89 | PPARGC1B |
| ENSG00000155850 | -2.819 | 1.021e-101 | 6.911e-100 | SLC26A2 |
| ENSG00000155858 | -1.815 | 1.001e-21 | 6.276e-21 | LSM11 |
| ENSG00000155876 | -1.988 | 1.318e-29 | 1.162e-28 | RRAGA |
| ENSG00000155893 | -3.572 | 1.734e-56 | 3.727e-55 | ACPL2 |
| ENSG00000155918 | 1.306 | 0.008472 | 0.01485 | RAET1L |
| ENSG00000155961 | 3.73 | 3.492e-08 | 1.031e-07 | RAB39B |
| ENSG00000155970 | -1.23 | 2.102e-06 | 5.377e-06 | MICU3 |
| ENSG00000155980 | 3.42 | 3.195e-13 | 1.311e-12 | KIF5A |
| ENSG00000155984 | -1.788 | 1.732e-18 | 9.32e-18 | TMEM185A |
| ENSG00000156017 | -1.209 | 2.739e-19 | 1.528e-18 | C9orf41 |
| ENSG00000156103 | -1.791 | 0.001856 | 0.003573 | MMP16 |
| ENSG00000156127 | -1.403 | 3.163e-23 | 2.111e-22 | BATF |
| ENSG00000156136 | -2.049 | 6.983e-34 | 7.373e-33 | DCK |
| ENSG00000156162 | -2.005 | 4.129e-62 | 1.08e-60 | DPY19L4 |
| ENSG00000156170 | -1.44 | 8.729e-16 | 4.086e-15 | NDUFAF6 |
| ENSG00000156172 | -1.165 | 8.578e-06 | 2.086e-05 | C8orf37 |
| ENSG00000156206 | 2.854 | 0.005309 | 0.009587 | C15orf26 |
| ENSG00000156253 | -1.795 | 1.852e-25 | 1.377e-24 | RWDD2B |
| ENSG00000156265 | 2.999 | 0.003923 | 0.007203 | MAP3K7CL |
| ENSG00000156273 | 1.581 | 3.871e-41 | 5.34e-40 | BACH1 |
| ENSG00000156299 | -1.859 | 2.85e-30 | 2.572e-29 | TIAM1 |
| ENSG00000156345 | -1.413 | 4e-11 | 1.448e-10 | CDK20 |
| ENSG00000156381 | -2.273 | 6.482e-18 | 3.388e-17 | ANKRD9 |
| ENSG00000156384 | -1.546 | 4.774e-12 | 1.827e-11 | SFR1 |
| ENSG00000156398 | -1.875 | 5.267e-17 | 2.629e-16 | SFXN2 |
| ENSG00000156413 | 1.47 | 0.008116 | 0.01427 | FUT6 |
| ENSG00000156453 | -1.374 | 1.74e-22 | 1.122e-21 | PCDH1 |
| ENSG00000156469 | -1.548 | 3.721e-27 | 2.96e-26 | MTERFD1 |
| ENSG00000156500 | 1.019 | 0.001337 | 0.002616 | FAM122C |
| ENSG00000156502 | 1.5 | 6.863e-70 | 2.198e-68 | SUPV3L1 |
| ENSG00000156510 | 1.469 | 2.504e-36 | 2.899e-35 | HKDC1 |
| ENSG00000156521 | -3.523 | 1.363e-78 | 5.41e-77 | TYSND1 |
| ENSG00000156599 | -1.211 | 8.308e-34 | 8.722e-33 | ZDHHC5 |
| ENSG00000156639 | 1.783 | 1.842e-103 | 1.283e-101 | ZFAND3 |
| ENSG00000156802 | -1.319 | 3.27e-28 | 2.711e-27 | ATAD2 |
| ENSG00000156860 | 1.357 | 3.646e-46 | 5.884e-45 | FBRS |
| ENSG00000156869 | -1.252 | 1.186e-11 | 4.439e-11 | FRRS1 |
| ENSG00000156928 | -2.034 | 9.18e-21 | 5.48e-20 | MALSU1 |
| ENSG00000156959 | 3.873 | 2.478e-06 | 6.302e-06 | LHFPL4 |
| ENSG00000156966 | -4.818 | 7.312e-11 | 2.6e-10 | B3GNT7 |
| ENSG00000156970 | -3.293 | 3.115e-147 | 4.628e-145 | BUB1B |
| ENSG00000156976 | -1.046 | 3.297e-29 | 2.865e-28 | EIF4A2 |
| ENSG00000156983 | -1.857 | 8.009e-27 | 6.288e-26 | BRPF1 |
| ENSG00000156990 | -1.297 | 2.162e-21 | 1.334e-20 | RPUSD3 |
| ENSG00000157014 | -1.638 | 1.556e-48 | 2.693e-47 | TATDN2 |
| ENSG00000157036 | -1.194 | 3.823e-07 | 1.046e-06 | EXOG |
| ENSG00000157111 | -1.724 | 2.494e-13 | 1.032e-12 | TMEM171 |
| ENSG00000157168 | 4.635 | 2.22e-80 | 9.246e-79 | NRG1 |
| ENSG00000157184 | -2.376 | 9.228e-87 | 4.556e-85 | CPT2 |
| ENSG00000157212 | -1.501 | 1.336e-23 | 9.079e-23 | PAXIP1 |
| ENSG00000157214 | -4.08 | 5.406e-64 | 1.483e-62 | STEAP2 |
| ENSG00000157216 | -1.931 | 6.202e-35 | 6.844e-34 | SSBP3 |
| ENSG00000157227 | -1.747 | 1.253e-09 | 4.117e-09 | MMP14 |
| ENSG00000157240 | -3.921 | 1.156e-42 | 1.673e-41 | FZD1 |
| ENSG00000157259 | -1.118 | 4.264e-18 | 2.249e-17 | GATAD1 |
| ENSG00000157335 | 1.786 | 0.004793 | 0.008696 | CLEC18C |
| ENSG00000157350 | -2.104 | 6.145e-12 | 2.335e-11 | ST3GAL2 |
| ENSG00000157429 | -1.137 | 0.004451 | 0.008105 | ZNF19 |
| ENSG00000157456 | -2.572 | 1.94e-40 | 2.605e-39 | CCNB2 |
| ENSG00000157470 | -2.139 | 1.988e-19 | 1.116e-18 | FAM81A |
| ENSG00000157500 | -1.679 | 3.84e-58 | 8.721e-57 | APPL1 |
| ENSG00000157540 | 1.8 | 2.017e-59 | 4.836e-58 | DYRK1A |
| ENSG00000157542 | 5.14 | 2.119e-07 | 5.906e-07 | KCNJ6 |
| ENSG00000157570 | 2.373 | 0.02384 | 0.03874 | TSPAN18 |
| ENSG00000157578 | -1.056 | 0.008683 | 0.01518 | LCA5L |
| ENSG00000157601 | -1.619 | 4.327e-12 | 1.66e-11 | MX1 |
| ENSG00000157617 | -1.241 | 4.817e-16 | 2.29e-15 | C2CD2 |
| ENSG00000157637 | -1.64 | 2.085e-49 | 3.695e-48 | SLC38A10 |
| ENSG00000157680 | 5.176 | 5.445e-07 | 1.468e-06 | DGKI |
| ENSG00000157734 | 2.527 | 0.0005385 | 0.001101 | SNX22 |
| ENSG00000157778 | -1.768 | 8.126e-22 | 5.101e-21 | PSMG3 |
| ENSG00000157833 | -2.899 | 1.784e-11 | 6.603e-11 | GAREML |
| ENSG00000157851 | -2.56 | 3.432e-06 | 8.633e-06 | DPYSL5 |
| ENSG00000157870 | -2.178 | 1.903e-32 | 1.893e-31 | FAM213B |
| ENSG00000157873 | -1.414 | 0.002304 | 0.004376 | TNFRSF14 |
| ENSG00000157881 | -1.688 | 2.17e-20 | 1.276e-19 | PANK4 |
| ENSG00000157911 | -1.509 | 4.744e-17 | 2.375e-16 | PEX10 |
| ENSG00000157927 | 5.055 | 1.368e-16 | 6.676e-16 | RADIL |
| ENSG00000157933 | -1.222 | 7.193e-36 | 8.197e-35 | SKI |
| ENSG00000157978 | -3.072 | 4.746e-97 | 2.918e-95 | LDLRAP1 |
| ENSG00000158042 | -1.756 | 4.552e-50 | 8.198e-49 | MRPL17 |
| ENSG00000158050 | 4.342 | 3.337e-53 | 6.543e-52 | DUSP2 |
| ENSG00000158079 | -1.282 | 3.053e-13 | 1.255e-12 | PTPDC1 |
| ENSG00000158092 | -1.039 | 1.319e-18 | 7.138e-18 | NCK1 |
| ENSG00000158104 | 3.063 | 3.058e-34 | 3.286e-33 | HPD |
| ENSG00000158106 | -2.638 | 9.858e-28 | 8.041e-27 | RHPN1 |
| ENSG00000158156 | -3.317 | 9.272e-15 | 4.132e-14 | XKR8 |
| ENSG00000158161 | 1.329 | 1.929e-21 | 1.194e-20 | EYA3 |
| ENSG00000158163 | -3.507 | 1.829e-08 | 5.508e-08 | DZIP1L |
| ENSG00000158169 | -1.613 | 2.17e-23 | 1.461e-22 | FANCC |
| ENSG00000158186 | -1.108 | 9.617e-06 | 2.33e-05 | MRAS |
| ENSG00000158201 | 1.274 | 4.458e-20 | 2.578e-19 | ABHD3 |
| ENSG00000158220 | -1.843 | 8.472e-18 | 4.396e-17 | ESYT3 |
| ENSG00000158234 | -2.093 | 2.141e-28 | 1.791e-27 | FAIM |
| ENSG00000158286 | -2.9 | 1.24e-27 | 1.006e-26 | RNF207 |
| ENSG00000158292 | -1.102 | 2.176e-09 | 7.011e-09 | GPR153 |
| ENSG00000158301 | -1.809 | 0.001204 | 0.002369 | GPRASP2 |
| ENSG00000158402 | -1.175 | 6.177e-16 | 2.917e-15 | CDC25C |
| ENSG00000158406 | 1.598 | 4.203e-06 | 1.05e-05 | HIST1H4H |
| ENSG00000158435 | -1.186 | 2.1e-25 | 1.558e-24 | CNOT11 |
| ENSG00000158480 | 2.584 | 1.31e-53 | 2.588e-52 | SPATA2 |
| ENSG00000158483 | -2.253 | 2.129e-43 | 3.172e-42 | FAM86C1 |
| ENSG00000158528 | -1.158 | 1.151e-21 | 7.192e-21 | PPP1R9A |
| ENSG00000158555 | -1.252 | 2.573e-09 | 8.249e-09 | GDPD5 |
| ENSG00000158615 | 1.116 | 9.61e-32 | 9.287e-31 | PPP1R15B |
| ENSG00000158683 | 1.572 | 0.02481 | 0.04022 | PKD1L1 |
| ENSG00000158691 | -2.66 | 1.586e-43 | 2.373e-42 | ZSCAN12 |
| ENSG00000158711 | -1.518 | 2.379e-28 | 1.987e-27 | ELK4 |
| ENSG00000158715 | -2.716 | 6.808e-62 | 1.761e-60 | SLC45A3 |
| ENSG00000158716 | -1.277 | 2.513e-06 | 6.387e-06 | DUSP23 |
| ENSG00000158717 | -1.766 | 8.904e-27 | 6.971e-26 | RNF166 |
| ENSG00000158764 | 3.082 | 0.01674 | 0.02798 | ITLN2 |
| ENSG00000158786 | -1.28 | 0.00163 | 0.00316 | PLA2G2F |
| ENSG00000158792 | -2.791 | 2.227e-11 | 8.202e-11 | SPATA2L |
| ENSG00000158806 | 2.476 | 0.009342 | 0.01627 | NPM2 |
| ENSG00000158815 | 4.928 | 8.439e-07 | 2.243e-06 | FGF17 |
| ENSG00000158859 | 3.077 | 0.01421 | 0.02406 | ADAMTS4 |
| ENSG00000158863 | -1.764 | 5.534e-40 | 7.326e-39 | FAM160B2 |
| ENSG00000158865 | 1.068 | 0.002293 | 0.004355 | SLC5A11 |
| ENSG00000158882 | -1.155 | 3.868e-05 | 8.884e-05 | TOMM40L |
| ENSG00000158966 | -1.277 | 1.777e-05 | 4.202e-05 | CACHD1 |
| ENSG00000158985 | -1.871 | 8.836e-60 | 2.137e-58 | CDC42SE2 |
| ENSG00000159079 | -1.2 | 9.162e-17 | 4.506e-16 | C21orf59 |
| ENSG00000159110 | -1.499 | 5.532e-11 | 1.984e-10 | IFNAR2 |
| ENSG00000159128 | -1.371 | 1.087e-20 | 6.471e-20 | IFNGR2 |
| ENSG00000159164 | 3.592 | 2.043e-07 | 5.709e-07 | SV2A |
| ENSG00000159167 | 1.789 | 1.919e-10 | 6.635e-10 | STC1 |
| ENSG00000159173 | -1.827 | 0.001 | 0.001985 | TNNI1 |
| ENSG00000159202 | -1.309 | 2.556e-25 | 1.883e-24 | UBE2Z |
| ENSG00000159212 | 3.486 | 0.002884 | 0.005398 | CLIC6 |
| ENSG00000159214 | -1.417 | 8.357e-16 | 3.917e-15 | CCDC24 |
| ENSG00000159217 | -1.967 | 6.626e-100 | 4.311e-98 | IGF2BP1 |
| ENSG00000159231 | -1.081 | 0.0001707 | 0.0003678 | CBR3 |
| ENSG00000159239 | -1.971 | 8.869e-05 | 0.000197 | C2orf81 |
| ENSG00000159256 | 2.297 | 4.792e-63 | 1.279e-61 | MORC3 |
| ENSG00000159259 | -1.944 | 1.255e-39 | 1.643e-38 | CHAF1B |
| ENSG00000159289 | 3.884 | 1.014e-09 | 3.354e-09 | GOLGA6A |
| ENSG00000159314 | -1.601 | 3.618e-28 | 2.993e-27 | ARHGAP27 |
| ENSG00000159337 | 2.554 | 1.294e-07 | 3.676e-07 | PLA2G4D |
| ENSG00000159374 | -1.791 | 2.736e-11 | 1.002e-10 | M1AP |
| ENSG00000159387 | -3.859 | 3.749e-23 | 2.492e-22 | IRX6 |
| ENSG00000159388 | -1.603 | 3.477e-07 | 9.548e-07 | BTG2 |
| ENSG00000159399 | 1.572 | 1.27e-42 | 1.833e-41 | HK2 |
| ENSG00000159433 | -1.688 | 0.00321 | 0.005969 | STARD9 |
| ENSG00000159445 | -2.351 | 4.129e-55 | 8.493e-54 | THEM4 |
| ENSG00000159450 | -2.475 | 1.781e-07 | 4.995e-07 | TCHH |
| ENSG00000159579 | 1.015 | 3.813e-31 | 3.577e-30 | RSPRY1 |
| ENSG00000159648 | 4.902 | 4.339e-09 | 1.369e-08 | TEPP |
| ENSG00000159692 | -2.349 | 4.187e-81 | 1.775e-79 | CTBP1 |
| ENSG00000159714 | -1.484 | 0.0002584 | 0.0005466 | ZDHHC1 |
| ENSG00000159733 | -2.032 | 2.946e-21 | 1.805e-20 | ZFYVE28 |
| ENSG00000159784 | 3.088 | 0.01218 | 0.02082 | FAM131B |
| ENSG00000159788 | -2.27 | 5.1e-29 | 4.395e-28 | RGS12 |
| ENSG00000159792 | -2.549 | 2.278e-24 | 1.606e-23 | PSKH1 |
| ENSG00000159840 | 1.529 | 1.831e-17 | 9.339e-17 | ZYX |
| ENSG00000159871 | -2.758 | 0.0005613 | 0.001146 | LYPD5 |
| ENSG00000159873 | 1.297 | 5.309e-38 | 6.601e-37 | CCDC117 |
| ENSG00000159885 | 2.608 | 1.798e-37 | 2.188e-36 | ZNF222 |
| ENSG00000159921 | -2.725 | 1.529e-72 | 5.237e-71 | GNE |
| ENSG00000159958 | -1.746 | 0.02286 | 0.03728 | TNFRSF13C |
| ENSG00000160007 | -1.855 | 7.465e-96 | 4.492e-94 | ARHGAP35 |
| ENSG00000160014 | -1.172 | 4.725e-31 | 4.401e-30 | CALM3 |
| ENSG00000160051 | -2.029 | 1.864e-29 | 1.637e-28 | IQCC |
| ENSG00000160062 | -1.466 | 2.059e-15 | 9.463e-15 | ZBTB8A |
| ENSG00000160094 | -1.599 | 4.569e-19 | 2.52e-18 | ZNF362 |
| ENSG00000160113 | -3.132 | 1.449e-62 | 3.848e-61 | NR2F6 |
| ENSG00000160117 | 1.315 | 0.00212 | 0.004049 | ANKLE1 |
| ENSG00000160145 | -1.371 | 0.0003949 | 0.0008198 | KALRN |
| ENSG00000160161 | 2.306 | 2.747e-10 | 9.418e-10 | CILP2 |
| ENSG00000160179 | 3.285 | 1.691e-15 | 7.796e-15 | ABCG1 |
| ENSG00000160190 | -1.554 | 1.062e-38 | 1.353e-37 | SLC37A1 |
| ENSG00000160194 | -1.886 | 5.61e-37 | 6.679e-36 | NDUFV3 |
| ENSG00000160201 | 1.17 | 3.116e-35 | 3.485e-34 | U2AF1 |
| ENSG00000160208 | -1.872 | 1.665e-69 | 5.285e-68 | RRP1B |
| ENSG00000160221 | -1.589 | 1.131e-34 | 1.238e-33 | C21orf33 |
| ENSG00000160223 | -1.892 | 2.102e-28 | 1.761e-27 | ICOSLG |
| ENSG00000160233 | -2.381 | 1.351e-07 | 3.832e-07 | LRRC3 |
| ENSG00000160256 | -1.563 | 2.051e-15 | 9.431e-15 | FAM207A |
| ENSG00000160271 | -1.616 | 1.035e-24 | 7.4e-24 | RALGDS |
| ENSG00000160282 | 2.886 | 1.474e-06 | 3.835e-06 | FTCD |
| ENSG00000160284 | -1.628 | 2.158e-10 | 7.446e-10 | SPATC1L |
| ENSG00000160285 | -1.476 | 3.511e-22 | 2.239e-21 | LSS |
| ENSG00000160298 | -3.919 | 2.564e-29 | 2.241e-28 | C21orf58 |
| ENSG00000160299 | -1.304 | 2.37e-41 | 3.293e-40 | PCNT |
| ENSG00000160310 | -1.014 | 3.503e-16 | 1.678e-15 | PRMT2 |
| ENSG00000160326 | -1.228 | 1.176e-07 | 3.349e-07 | SLC2A6 |
| ENSG00000160360 | 1.485 | 1.52e-22 | 9.835e-22 | GPSM1 |
| ENSG00000160401 | -1.575 | 2.426e-06 | 6.176e-06 | C9orf117 |
| ENSG00000160404 | -2.973 | 4.107e-19 | 2.273e-18 | TOR2A |
| ENSG00000160445 | -1.278 | 2.107e-24 | 1.486e-23 | ZER1 |
| ENSG00000160446 | -1.275 | 1.317e-12 | 5.213e-12 | ZDHHC12 |
| ENSG00000160447 | -1.454 | 6.838e-09 | 2.127e-08 | PKN3 |
| ENSG00000160471 | 3.905 | 4.78e-43 | 7.007e-42 | COX6B2 |
| ENSG00000160472 | 2.792 | 2.554e-11 | 9.375e-11 | TMEM190 |
| ENSG00000160505 | 5.546 | 9.709e-09 | 2.985e-08 | NLRP4 |
| ENSG00000160570 | 2.996 | 3.962e-272 | 2.375e-269 | DEDD2 |
| ENSG00000160588 | 1.25 | 2.156e-11 | 7.95e-11 | MPZL3 |
| ENSG00000160593 | 3.556 | 0.002745 | 0.00516 | AMICA1 |
| ENSG00000160606 | -3.326 | 7.57e-35 | 8.322e-34 | TLCD1 |
| ENSG00000160688 | -1.536 | 2.756e-17 | 1.394e-16 | FLAD1 |
| ENSG00000160695 | 1.067 | 1.009e-23 | 6.911e-23 | VPS11 |
| ENSG00000160703 | -3.319 | 8.233e-39 | 1.052e-37 | NLRX1 |
| ENSG00000160710 | -1.324 | 1.232e-51 | 2.315e-50 | ADAR |
| ENSG00000160712 | 1.832 | 1.84e-26 | 1.422e-25 | IL6R |
| ENSG00000160741 | 1.971 | 1.036e-19 | 5.894e-19 | CRTC2 |
| ENSG00000160753 | -1.139 | 1.69e-21 | 1.05e-20 | RUSC1 |
| ENSG00000160767 | -1.573 | 2.793e-21 | 1.712e-20 | FAM189B |
| ENSG00000160781 | -1.057 | 0.002376 | 0.004504 | PAQR6 |
| ENSG00000160785 | 1.346 | 2.871e-24 | 2.016e-23 | SLC25A44 |
| ENSG00000160796 | -1.736 | 2.014e-69 | 6.367e-68 | NBEAL2 |
| ENSG00000160803 | -1.365 | 3.258e-21 | 1.992e-20 | UBQLN4 |
| ENSG00000160813 | -1.773 | 5.125e-12 | 1.957e-11 | PPP1R35 |
| ENSG00000160877 | -1.389 | 1.838e-48 | 3.161e-47 | NACC1 |
| ENSG00000160908 | 2.456 | 1.552e-74 | 5.625e-73 | ZNF394 |
| ENSG00000160917 | -2.741 | 1.534e-25 | 1.146e-24 | CPSF4 |
| ENSG00000160949 | -2.683 | 1.198e-42 | 1.732e-41 | TONSL |
| ENSG00000160957 | -1.572 | 4.101e-29 | 3.552e-28 | RECQL4 |
| ENSG00000160959 | -2.608 | 9.17e-44 | 1.378e-42 | LRRC14 |
| ENSG00000160972 | -1.027 | 2.593e-18 | 1.383e-17 | PPP1R16A |
| ENSG00000160991 | -1.346 | 4.702e-10 | 1.586e-09 | ORAI2 |
| ENSG00000161011 | 1.52 | 5.496e-82 | 2.366e-80 | SQSTM1 |
| ENSG00000161021 | -1.121 | 6.148e-38 | 7.617e-37 | MAML1 |
| ENSG00000161040 | 1.36 | 4.226e-13 | 1.721e-12 | FBXL13 |
| ENSG00000161048 | -2.945 | 1.398e-68 | 4.328e-67 | NAPEPLD |
| ENSG00000161103 | -1.675 | 0.0264 | 0.04262 | AC008132.13 |
| ENSG00000161179 | -2.548 | 4.348e-70 | 1.401e-68 | YDJC |
| ENSG00000161180 | 1.731 | 0.002685 | 0.005056 | CCDC116 |
| ENSG00000161243 | -1.077 | 2.106e-12 | 8.225e-12 | FBXO27 |
| ENSG00000161265 | 1.206 | 1.075e-13 | 4.538e-13 | U2AF1L4 |
| ENSG00000161267 | -2.084 | 1.038e-66 | 3.085e-65 | BDH1 |
| ENSG00000161277 | -1.119 | 0.008703 | 0.01521 | THAP8 |
| ENSG00000161298 | 2.967 | 6.519e-10 | 2.178e-09 | ZNF382 |
| ENSG00000161381 | 1.923 | 1.887e-08 | 5.678e-08 | PLXDC1 |
| ENSG00000161395 | -1.449 | 7.616e-06 | 1.862e-05 | PGAP3 |
| ENSG00000161509 | 1.757 | 0.008553 | 0.01498 | GRIN2C |
| ENSG00000161526 | 1.169 | 8.927e-35 | 9.8e-34 | SAP30BP |
| ENSG00000161533 | -1.015 | 2.002e-13 | 8.327e-13 | ACOX1 |
| ENSG00000161542 | -1.429 | 3.76e-19 | 2.084e-18 | PRPSAP1 |
| ENSG00000161544 | 2.262 | 0.006846 | 0.01217 | CYGB |
| ENSG00000161551 | -2.149 | 2.673e-10 | 9.172e-10 | ZNF577 |
| ENSG00000161558 | -1.351 | 4.107e-09 | 1.298e-08 | TMEM143 |
| ENSG00000161618 | -1.537 | 5.574e-12 | 2.124e-11 | ALDH16A1 |
| ENSG00000161638 | 1.123 | 1.89e-18 | 1.014e-17 | ITGA5 |
| ENSG00000161640 | 3.597 | 6.802e-06 | 1.669e-05 | SIGLEC11 |
| ENSG00000161664 | -4.199 | 1.412e-10 | 4.931e-10 | ASB16 |
| ENSG00000161681 | 2.98 | 0.01713 | 0.02858 | SHANK1 |
| ENSG00000161692 | -1.157 | 1.004e-17 | 5.191e-17 | DBF4B |
| ENSG00000161791 | -1.646 | 7.298e-21 | 4.38e-20 | FMNL3 |
| ENSG00000161800 | -2.048 | 2.264e-84 | 1.047e-82 | RACGAP1 |
| ENSG00000161847 | -1.54 | 2.007e-28 | 1.682e-27 | RAVER1 |
| ENSG00000161860 | 2.1 | 2.27e-08 | 6.794e-08 | SYCE2 |
| ENSG00000161888 | -3.863 | 7.021e-19 | 3.84e-18 | SPC24 |
| ENSG00000161920 | -1.051 | 4.212e-10 | 1.424e-09 | MED11 |
| ENSG00000161955 | -1.131 | 6.944e-13 | 2.793e-12 | TNFSF13 |
| ENSG00000161956 | -2.245 | 8.6e-07 | 2.285e-06 | SENP3 |
| ENSG00000161958 | -1.975 | 8.83e-18 | 4.579e-17 | FGF11 |
| ENSG00000161980 | -1.043 | 3.231e-07 | 8.893e-07 | POLR3K |
| ENSG00000161996 | -1.946 | 1.255e-25 | 9.402e-25 | WDR90 |
| ENSG00000162062 | -3.357 | 4.572e-46 | 7.344e-45 | C16orf59 |
| ENSG00000162063 | -3.201 | 4.699e-145 | 6.8e-143 | CCNF |
| ENSG00000162065 | -2.84 | 3.244e-59 | 7.659e-58 | TBC1D24 |
| ENSG00000162066 | -1.602 | 4.988e-17 | 2.493e-16 | AMDHD2 |
| ENSG00000162073 | -4.228 | 5.668e-154 | 1.002e-151 | PAQR4 |
| ENSG00000162076 | -2.072 | 1.033e-22 | 6.722e-22 | FLYWCH2 |
| ENSG00000162078 | -1.275 | 2.623e-06 | 6.659e-06 | ZG16B |
| ENSG00000162104 | -1.992 | 1.919e-30 | 1.743e-29 | ADCY9 |
| ENSG00000162139 | -2.376 | 6.704e-46 | 1.074e-44 | NEU3 |
| ENSG00000162144 | -1.103 | 5.788e-12 | 2.203e-11 | CYB561A3 |
| ENSG00000162148 | 2.164 | 3.413e-06 | 8.594e-06 | PPP1R32 |
| ENSG00000162194 | -1.154 | 3.865e-21 | 2.355e-20 | C11orf48 |
| ENSG00000162222 | -2.69 | 2.436e-34 | 2.63e-33 | TTC9C |
| ENSG00000162227 | -1.471 | 1.415e-08 | 4.291e-08 | TAF6L |
| ENSG00000162231 | 1.677 | 3.172e-97 | 1.965e-95 | NXF1 |
| ENSG00000162241 | -1.399 | 4.169e-08 | 1.224e-07 | SLC25A45 |
| ENSG00000162337 | -1.904 | 1.964e-60 | 4.878e-59 | LRP5 |
| ENSG00000162341 | -1.594 | 1.354e-08 | 4.113e-08 | TPCN2 |
| ENSG00000162344 | 2.839 | 5.237e-10 | 1.76e-09 | FGF19 |
| ENSG00000162377 | -1.903 | 6.177e-32 | 6.007e-31 | SELRC1 |
| ENSG00000162378 | -1.863 | 2.507e-58 | 5.725e-57 | ZYG11B |
| ENSG00000162385 | 1.027 | 1.785e-13 | 7.449e-13 | MAGOH |
| ENSG00000162390 | -1.728 | 1.923e-25 | 1.427e-24 | ACOT11 |
| ENSG00000162396 | -1.13 | 6.407e-08 | 1.856e-07 | PARS2 |
| ENSG00000162407 | 1.05 | 8.164e-13 | 3.272e-12 | PPAP2B |
| ENSG00000162413 | 1.361 | 1.421e-11 | 5.289e-11 | KLHL21 |
| ENSG00000162415 | -2.687 | 4.761e-14 | 2.045e-13 | ZSWIM5 |
| ENSG00000162419 | -1.011 | 5.765e-06 | 1.424e-05 | GMEB1 |
| ENSG00000162433 | -1.08 | 1.205e-33 | 1.254e-32 | AK4 |
| ENSG00000162437 | -1.241 | 2.223e-06 | 5.677e-06 | RAVER2 |
| ENSG00000162482 | -2.343 | 3.541e-05 | 8.161e-05 | AKR7A3 |
| ENSG00000162496 | -2.068 | 2.473e-45 | 3.888e-44 | DHRS3 |
| ENSG00000162512 | -1.41 | 4.27e-24 | 2.97e-23 | SDC3 |
| ENSG00000162521 | -1.067 | 2.174e-19 | 1.216e-18 | RBBP4 |
| ENSG00000162522 | -1.629 | 2.604e-94 | 1.528e-92 | KIAA1522 |
| ENSG00000162526 | 1.362 | 0.004541 | 0.008257 | TSSK3 |
| ENSG00000162543 | -3.377 | 1.372e-32 | 1.373e-31 | UBXN10 |
| ENSG00000162552 | -1.41 | 0.01282 | 0.02184 | WNT4 |
| ENSG00000162572 | 1.398 | 0.008331 | 0.01461 | SCNN1D |
| ENSG00000162585 | -1.166 | 1.347e-12 | 5.328e-12 | C1orf86 |
| ENSG00000162591 | -1.569 | 1.518e-07 | 4.289e-07 | MEGF6 |
| ENSG00000162595 | 3.949 | 0.002279 | 0.004331 | DIRAS3 |
| ENSG00000162599 | -2.384 | 4.301e-22 | 2.731e-21 | NFIA |
| ENSG00000162600 | -1.293 | 1.831e-21 | 1.136e-20 | OMA1 |
| ENSG00000162607 | -1.744 | 4.182e-82 | 1.805e-80 | USP1 |
| ENSG00000162614 | -1.452 | 3.434e-07 | 9.432e-07 | NEXN |
| ENSG00000162616 | 2.177 | 3.01e-71 | 9.965e-70 | DNAJB4 |
| ENSG00000162620 | -2.709 | 0.001879 | 0.003615 | LRRIQ3 |
| ENSG00000162636 | -2.719 | 6.707e-30 | 5.972e-29 | FAM102B |
| ENSG00000162639 | -2.764 | 7.533e-18 | 3.92e-17 | HENMT1 |
| ENSG00000162642 | 2.09 | 4.136e-86 | 1.995e-84 | C1orf52 |
| ENSG00000162688 | -1.363 | 5.916e-41 | 8.099e-40 | AGL |
| ENSG00000162694 | -2.252 | 9.089e-37 | 1.07e-35 | EXTL2 |
| ENSG00000162702 | 1.462 | 3.767e-27 | 2.995e-26 | ZNF281 |
| ENSG00000162714 | -1.279 | 2.836e-19 | 1.581e-18 | ZNF496 |
| ENSG00000162733 | 3.774 | 2.357e-62 | 6.221e-61 | DDR2 |
| ENSG00000162734 | 1.592 | 2.375e-34 | 2.569e-33 | PEA15 |
| ENSG00000162738 | -1.859 | 6.448e-35 | 7.112e-34 | VANGL2 |
| ENSG00000162755 | -2.087 | 5.427e-05 | 0.0001231 | KLHDC9 |
| ENSG00000162757 | -1.829 | 1.655e-11 | 6.136e-11 | C1orf74 |
| ENSG00000162769 | -1.126 | 3.321e-12 | 1.28e-11 | FLVCR1 |
| ENSG00000162772 | 6.965 | 2.755e-242 | 1.445e-239 | ATF3 |
| ENSG00000162777 | -1.901 | 4.36e-52 | 8.298e-51 | DENND2D |
| ENSG00000162783 | 4.06 | 1.201e-70 | 3.922e-69 | IER5 |
| ENSG00000162817 | -2.224 | 3.296e-27 | 2.628e-26 | C1orf115 |
| ENSG00000162836 | -1.687 | 1.254e-30 | 1.149e-29 | ACP6 |
| ENSG00000162852 | 1.749 | 6.088e-69 | 1.896e-67 | CNST |
| ENSG00000162888 | -2.278 | 0.01261 | 0.02152 | C1orf147 |
| ENSG00000162892 | 3.436 | 9.296e-05 | 0.0002061 | IL24 |
| ENSG00000162894 | 1.155 | 0.01486 | 0.02506 | FAIM3 |
| ENSG00000162897 | 4.276 | 6.08e-05 | 0.0001373 | FCAMR |
| ENSG00000162910 | -1.017 | 1.533e-05 | 3.644e-05 | MRPL55 |
| ENSG00000162923 | 1.268 | 2.851e-56 | 6.089e-55 | WDR26 |
| ENSG00000162928 | -3.442 | 1.361e-144 | 1.937e-142 | PEX13 |
| ENSG00000162929 | -1.524 | 3.754e-24 | 2.617e-23 | KIAA1841 |
| ENSG00000162971 | -1.309 | 4.225e-20 | 2.448e-19 | TYW5 |
| ENSG00000162972 | -1.245 | 9.882e-11 | 3.484e-10 | C2orf47 |
| ENSG00000162976 | -1.007 | 4.099e-05 | 9.398e-05 | PQLC3 |
| ENSG00000162981 | -3.291 | 0.003005 | 0.005613 | FAM84A |
| ENSG00000162998 | 1.717 | 0.001243 | 0.00244 | FRZB |
| ENSG00000162999 | -1.542 | 0.0007998 | 0.001603 | DUSP19 |
| ENSG00000163006 | -2.472 | 5.453e-21 | 3.3e-20 | CCDC138 |
| ENSG00000163009 | -1.224 | 0.0004274 | 0.0008837 | C2orf48 |
| ENSG00000163013 | -1.376 | 1.736e-23 | 1.173e-22 | FBXO41 |
| ENSG00000163029 | -1.038 | 1.461e-08 | 4.425e-08 | SMC6 |
| ENSG00000163050 | -1.61 | 1.874e-37 | 2.278e-36 | ADCK3 |
| ENSG00000163053 | -1.303 | 0.01788 | 0.02975 | SLC16A14 |
| ENSG00000163069 | -1.276 | 0.0001161 | 0.0002547 | SGCB |
| ENSG00000163072 | -2.698 | 5.16e-05 | 0.0001172 | NOSTRIN |
| ENSG00000163082 | 1.627 | 4.053e-21 | 2.467e-20 | SGPP2 |
| ENSG00000163083 | -3.089 | 1.008e-146 | 1.484e-144 | INHBB |
| ENSG00000163092 | 2.902 | 0.00448 | 0.008155 | XIRP2 |
| ENSG00000163093 | -2.411 | 1.034e-09 | 3.418e-09 | BBS5 |
| ENSG00000163110 | 1.509 | 1.548e-92 | 8.722e-91 | PDLIM5 |
| ENSG00000163132 | 3.188 | 5.302e-09 | 1.663e-08 | MSX1 |
| ENSG00000163138 | -1.109 | 9.556e-11 | 3.371e-10 | PACRGL |
| ENSG00000163141 | -1.729 | 0.003976 | 0.007294 | BNIPL |
| ENSG00000163159 | -1.578 | 1.471e-28 | 1.241e-27 | VPS72 |
| ENSG00000163171 | -1.338 | 4.45e-18 | 2.345e-17 | CDC42EP3 |
| ENSG00000163214 | -1.83 | 1.68e-43 | 2.506e-42 | DHX57 |
| ENSG00000163219 | 6.573 | 2.9e-13 | 1.194e-12 | ARHGAP25 |
| ENSG00000163251 | -2.587 | 2.282e-41 | 3.176e-40 | FZD5 |
| ENSG00000163257 | -1.889 | 8.047e-60 | 1.949e-58 | DCAF16 |
| ENSG00000163328 | -1.337 | 0.01417 | 0.02399 | GPR155 |
| ENSG00000163346 | -1.65 | 7.693e-18 | 4e-17 | PBXIP1 |
| ENSG00000163352 | 2.773 | 0.01738 | 0.02897 | LENEP |
| ENSG00000163359 | 4.407 | 2.051e-12 | 8.015e-12 | COL6A3 |
| ENSG00000163374 | 1.208 | 1.662e-15 | 7.662e-15 | YY1AP1 |
| ENSG00000163376 | 4.534 | 1.608e-57 | 3.588e-56 | KBTBD8 |
| ENSG00000163389 | -2.101 | 4.095e-47 | 6.84e-46 | POGLUT1 |
| ENSG00000163395 | 2.058 | 4.132e-42 | 5.893e-41 | IGFN1 |
| ENSG00000163406 | -1.398 | 0.005992 | 0.01073 | SLC15A2 |
| ENSG00000163428 | -1.746 | 1.783e-73 | 6.26e-72 | LRRC58 |
| ENSG00000163430 | -1.06 | 9.985e-19 | 5.429e-18 | FSTL1 |
| ENSG00000163435 | -1.224 | 2.576e-14 | 1.122e-13 | ELF3 |
| ENSG00000163462 | -2.882 | 1.371e-21 | 8.535e-21 | TRIM46 |
| ENSG00000163464 | 4.458 | 2.633e-05 | 6.131e-05 | CXCR1 |
| ENSG00000163467 | 1.374 | 4.811e-08 | 1.406e-07 | TSACC |
| ENSG00000163479 | 1.045 | 3.851e-31 | 3.611e-30 | SSR2 |
| ENSG00000163482 | -1.638 | 1.851e-30 | 1.682e-29 | STK36 |
| ENSG00000163485 | -5.607 | 5.062e-47 | 8.421e-46 | ADORA1 |
| ENSG00000163492 | 3.127 | 0.02126 | 0.03488 | CCDC141 |
| ENSG00000163510 | 1.865 | 1.1e-25 | 8.272e-25 | CWC22 |
| ENSG00000163512 | 1.228 | 1.2e-23 | 8.177e-23 | AZI2 |
| ENSG00000163513 | -2.426 | 1.311e-68 | 4.068e-67 | TGFBR2 |
| ENSG00000163516 | -2.035 | 1.538e-47 | 2.586e-46 | ANKZF1 |
| ENSG00000163517 | -1.211 | 4.431e-11 | 1.598e-10 | HDAC11 |
| ENSG00000163521 | -1.064 | 0.000194 | 0.0004156 | GLB1L |
| ENSG00000163528 | -1.718 | 5.525e-30 | 4.926e-29 | CHCHD4 |
| ENSG00000163531 | 1.71 | 0.01422 | 0.02407 | NFASC |
| ENSG00000163535 | -1.977 | 1.349e-27 | 1.09e-26 | SGOL2 |
| ENSG00000163545 | -1.496 | 6.939e-29 | 5.951e-28 | NUAK2 |
| ENSG00000163577 | 1.013 | 1.427e-20 | 8.442e-20 | EIF5A2 |
| ENSG00000163590 | -2.496 | 3.591e-28 | 2.972e-27 | PPM1L |
| ENSG00000163596 | -1.323 | 0.002317 | 0.004397 | ICA1L |
| ENSG00000163602 | 1.754 | 8.985e-39 | 1.147e-37 | RYBP |
| ENSG00000163606 | 3.495 | 3.549e-19 | 1.971e-18 | CD200R1 |
| ENSG00000163611 | -1.326 | 2.747e-17 | 1.39e-16 | SPICE1 |
| ENSG00000163623 | 1.179 | 0.0009872 | 0.00196 | NKX6-1 |
| ENSG00000163626 | -1.729 | 8.918e-20 | 5.09e-19 | COX18 |
| ENSG00000163629 | -2.022 | 4.365e-23 | 2.884e-22 | PTPN13 |
| ENSG00000163635 | -1.133 | 1.982e-09 | 6.408e-09 | ATXN7 |
| ENSG00000163636 | 1.103 | 9.032e-15 | 4.03e-14 | PSMD6 |
| ENSG00000163660 | 1.109 | 1.075e-40 | 1.454e-39 | CCNL1 |
| ENSG00000163686 | -1.163 | 2.538e-05 | 5.917e-05 | ABHD6 |
| ENSG00000163687 | 4.373 | 3.032e-07 | 8.363e-07 | DNASE1L3 |
| ENSG00000163694 | -1.378 | 2.338e-57 | 5.198e-56 | RBM47 |
| ENSG00000163701 | -1.865 | 5.681e-20 | 3.271e-19 | IL17RE |
| ENSG00000163702 | -1.034 | 1.504e-07 | 4.249e-07 | IL17RC |
| ENSG00000163704 | 1.224 | 0.0001973 | 0.0004224 | PRRT3 |
| ENSG00000163710 | -1.284 | 1.465e-05 | 3.488e-05 | PCOLCE2 |
| ENSG00000163719 | 1.223 | 5.196e-16 | 2.463e-15 | MTMR14 |
| ENSG00000163734 | 1.525 | 0.03095 | 0.04931 | CXCL3 |
| ENSG00000163739 | 4.349 | 5.633e-84 | 2.598e-82 | CXCL1 |
| ENSG00000163755 | -1.492 | 4.801e-17 | 2.402e-16 | HPS3 |
| ENSG00000163795 | 1.297 | 4.58e-19 | 2.525e-18 | ZNF513 |
| ENSG00000163806 | 2.321 | 1.971e-17 | 1.003e-16 | SPDYA |
| ENSG00000163808 | -2.781 | 9.18e-75 | 3.35e-73 | KIF15 |
| ENSG00000163820 | -2.049 | 1.456e-51 | 2.725e-50 | FYCO1 |
| ENSG00000163832 | -1.662 | 1.078e-08 | 3.299e-08 | ELP6 |
| ENSG00000163840 | -2.07 | 2.195e-98 | 1.375e-96 | DTX3L |
| ENSG00000163864 | -3.331 | 0.002793 | 0.005242 | NMNAT3 |
| ENSG00000163866 | -2.513 | 3.585e-42 | 5.117e-41 | SMIM12 |
| ENSG00000163872 | -1.718 | 1.606e-59 | 3.867e-58 | YEATS2 |
| ENSG00000163874 | 2.842 | 1.404e-175 | 3.273e-173 | ZC3H12A |
| ENSG00000163877 | 2.914 | 4.192e-120 | 4.188e-118 | SNIP1 |
| ENSG00000163879 | 4.173 | 1.951e-08 | 5.866e-08 | DNALI1 |
| ENSG00000163888 | -1.308 | 0.001596 | 0.003096 | CAMK2N2 |
| ENSG00000163898 | -1.567 | 1.387e-28 | 1.174e-27 | LIPH |
| ENSG00000163900 | -2.596 | 1.903e-39 | 2.475e-38 | TMEM41A |
| ENSG00000163909 | -2.339 | 2.354e-06 | 6e-06 | HEYL |
| ENSG00000163915 | 2.003 | 0.002321 | 0.004405 | C3orf65 |
| ENSG00000163918 | -2.04 | 3.99e-66 | 1.163e-64 | RFC4 |
| ENSG00000163923 | -1.483 | 1.646e-09 | 5.357e-09 | RPL39L |
| ENSG00000163933 | -1.312 | 3.671e-10 | 1.247e-09 | RFT1 |
| ENSG00000163935 | -2.029 | 1.345e-57 | 3.005e-56 | SFMBT1 |
| ENSG00000163958 | 4.452 | 1.988e-05 | 4.683e-05 | ZDHHC19 |
| ENSG00000163959 | 2.538 | 1.448e-21 | 9.003e-21 | SLC51A |
| ENSG00000163964 | -1.23 | 8.331e-19 | 4.539e-18 | PIGX |
| ENSG00000164002 | -3.222 | 3.542e-30 | 3.177e-29 | EXO5 |
| ENSG00000164031 | -1.379 | 3.133e-23 | 2.092e-22 | DNAJB14 |
| ENSG00000164038 | -1.035 | 1.198e-05 | 2.874e-05 | SLC9B2 |
| ENSG00000164045 | -3.574 | 1.816e-58 | 4.165e-57 | CDC25A |
| ENSG00000164048 | -2.15 | 3.37e-24 | 2.357e-23 | ZNF589 |
| ENSG00000164050 | -1.081 | 7.315e-16 | 3.44e-15 | PLXNB1 |
| ENSG00000164051 | -2.442 | 1.032e-36 | 1.211e-35 | CCDC51 |
| ENSG00000164053 | -3.33 | 1.744e-44 | 2.67e-43 | ATRIP |
| ENSG00000164056 | -3.218 | 5.094e-17 | 2.544e-16 | SPRY1 |
| ENSG00000164061 | -1.98 | 1.03e-09 | 3.405e-09 | BSN |
| ENSG00000164066 | -1.29 | 3.613e-13 | 1.479e-12 | INTU |
| ENSG00000164070 | 1.416 | 1.118e-25 | 8.402e-25 | HSPA4L |
| ENSG00000164073 | -1.738 | 1.273e-29 | 1.124e-28 | MFSD8 |
| ENSG00000164074 | -1.731 | 2.51e-15 | 1.148e-14 | C4orf29 |
| ENSG00000164077 | -1.095 | 2.055e-05 | 4.832e-05 | MON1A |
| ENSG00000164078 | -2.044 | 7.494e-33 | 7.578e-32 | MST1R |
| ENSG00000164080 | -1.217 | 1.331e-13 | 5.595e-13 | RAD54L2 |
| ENSG00000164081 | -1.187 | 7.348e-12 | 2.777e-11 | TEX264 |
| ENSG00000164086 | -1.864 | 3.023e-14 | 1.31e-13 | DUSP7 |
| ENSG00000164087 | -1.978 | 1.855e-17 | 9.453e-17 | POC1A |
| ENSG00000164099 | -1.452 | 3.638e-06 | 9.132e-06 | PRSS12 |
| ENSG00000164104 | -2.917 | 1.242e-118 | 1.185e-116 | HMGB2 |
| ENSG00000164109 | -2.002 | 1.366e-77 | 5.358e-76 | MAD2L1 |
| ENSG00000164111 | 1.217 | 1.606e-33 | 1.662e-32 | ANXA5 |
| ENSG00000164114 | -2.694 | 7.972e-15 | 3.567e-14 | MAP9 |
| ENSG00000164116 | -2.329 | 0.01194 | 0.02044 | GUCY1A3 |
| ENSG00000164122 | 5.577 | 3.392e-09 | 1.079e-08 | ASB5 |
| ENSG00000164124 | -2.022 | 2.143e-25 | 1.587e-24 | TMEM144 |
| ENSG00000164125 | -2.84 | 0.001574 | 0.003055 | FAM198B |
| ENSG00000164142 | 1.554 | 7.437e-31 | 6.886e-30 | FAM160A1 |
| ENSG00000164161 | 2.098 | 0.009665 | 0.01678 | HHIP |
| ENSG00000164167 | -1.462 | 4.164e-13 | 1.697e-12 | LSM6 |
| ENSG00000164171 | 1.605 | 3.222e-59 | 7.617e-58 | ITGA2 |
| ENSG00000164211 | 1.408 | 3.951e-08 | 1.163e-07 | STARD4 |
| ENSG00000164220 | 2.935 | 1.105e-32 | 1.11e-31 | F2RL2 |
| ENSG00000164221 | -1.008 | 4.395e-06 | 1.096e-05 | CCDC112 |
| ENSG00000164241 | -1.592 | 6.817e-21 | 4.103e-20 | C5orf63 |
| ENSG00000164251 | 1.06 | 5.185e-28 | 4.275e-27 | F2RL1 |
| ENSG00000164252 | -2.031 | 1.047e-58 | 2.418e-57 | AGGF1 |
| ENSG00000164266 | 6.464 | 2.718e-12 | 1.054e-11 | SPINK1 |
| ENSG00000164283 | 3.01 | 0.003907 | 0.007175 | ESM1 |
| ENSG00000164291 | -1.885 | 9.611e-13 | 3.838e-12 | ARSK |
| ENSG00000164294 | -1.944 | 2.989e-31 | 2.814e-30 | GPX8 |
| ENSG00000164306 | -2.127 | 3.688e-15 | 1.676e-14 | PRIMPOL |
| ENSG00000164318 | 3.509 | 0.007316 | 0.01295 | EGFLAM |
| ENSG00000164323 | -1.309 | 9.425e-27 | 7.372e-26 | KIAA1430 |
| ENSG00000164327 | 1.553 | 6.391e-64 | 1.744e-62 | RICTOR |
| ENSG00000164342 | -3.501 | 2.432e-35 | 2.73e-34 | TLR3 |
| ENSG00000164362 | -1.064 | 0.009766 | 0.01695 | TERT |
| ENSG00000164366 | -1.812 | 5.328e-31 | 4.958e-30 | CCDC127 |
| ENSG00000164379 | -2.753 | 1.69e-77 | 6.599e-76 | FOXQ1 |
| ENSG00000164400 | 4.397 | 5.156e-16 | 2.446e-15 | CSF2 |
| ENSG00000164402 | -1.513 | 1.177e-50 | 2.157e-49 | SEPT8 |
| ENSG00000164403 | -2.642 | 3.976e-45 | 6.22e-44 | SHROOM1 |
| ENSG00000164404 | 6.681 | 2.493e-07 | 6.921e-07 | GDF9 |
| ENSG00000164406 | 2.843 | 1.168e-20 | 6.943e-20 | LEAP2 |
| ENSG00000164414 | -2.07 | 8.045e-27 | 6.313e-26 | SLC35A1 |
| ENSG00000164463 | 3.386 | 2.436e-192 | 7.435e-190 | CREBRF |
| ENSG00000164465 | 1.087 | 1.025e-13 | 4.333e-13 | DCBLD1 |
| ENSG00000164466 | -1.195 | 1.795e-46 | 2.923e-45 | SFXN1 |
| ENSG00000164509 | 2.804 | 0.03087 | 0.0492 | IL31RA |
| ENSG00000164543 | 1.329 | 4.816e-46 | 7.728e-45 | STK17A |
| ENSG00000164556 | 5.281 | 1.435e-07 | 4.062e-07 | FAM183B |
| ENSG00000164574 | -1.344 | 1.859e-67 | 5.642e-66 | GALNT10 |
| ENSG00000164603 | 1.762 | 2.194e-37 | 2.658e-36 | C7orf60 |
| ENSG00000164609 | 2.172 | 9.383e-154 | 1.641e-151 | SLU7 |
| ENSG00000164610 | -1.332 | 7.043e-06 | 1.726e-05 | RP9 |
| ENSG00000164611 | -1.388 | 1.544e-27 | 1.246e-26 | PTTG1 |
| ENSG00000164626 | -4.646 | 4.368e-54 | 8.74e-53 | KCNK5 |
| ENSG00000164647 | -2.145 | 4.436e-21 | 2.696e-20 | STEAP1 |
| ENSG00000164649 | -1.888 | 1.381e-58 | 3.181e-57 | CDCA7L |
| ENSG00000164654 | -1.983 | 2.191e-41 | 3.055e-40 | MIOS |
| ENSG00000164659 | -1.708 | 2.927e-16 | 1.407e-15 | KIAA1324L |
| ENSG00000164674 | -1.035 | 0.0005303 | 0.001085 | SYTL3 |
| ENSG00000164675 | 2.574 | 2.13e-06 | 5.444e-06 | IQUB |
| ENSG00000164683 | 1.662 | 1.336e-07 | 3.793e-07 | HEY1 |
| ENSG00000164684 | -2.697 | 1.036e-179 | 2.52e-177 | ZNF704 |
| ENSG00000164715 | 1.159 | 8.626e-53 | 1.672e-51 | LMTK2 |
| ENSG00000164742 | -1.843 | 4.307e-06 | 1.075e-05 | ADCY1 |
| ENSG00000164743 | -2.823 | 0.0002963 | 0.0006236 | C8orf48 |
| ENSG00000164746 | 2.801 | 3.242e-08 | 9.604e-08 | C7orf57 |
| ENSG00000164751 | -1.812 | 1.058e-32 | 1.062e-31 | PEX2 |
| ENSG00000164764 | -1.741 | 0.001187 | 0.002335 | SBSPON |
| ENSG00000164776 | 1.282 | 2.593e-05 | 6.043e-05 | PHKG1 |
| ENSG00000164794 | -2.166 | 0.003194 | 0.005942 | KCNV1 |
| ENSG00000164815 | -1.461 | 1.17e-33 | 1.22e-32 | ORC5 |
| ENSG00000164818 | -1.449 | 8.132e-50 | 1.455e-48 | HEATR2 |
| ENSG00000164823 | 1.087 | 3.053e-28 | 2.533e-27 | OSGIN2 |
| ENSG00000164850 | 1.858 | 5.457e-07 | 1.471e-06 | GPER1 |
| ENSG00000164855 | -1.072 | 6.497e-18 | 3.395e-17 | TMEM184A |
| ENSG00000164867 | 2.392 | 2.464e-11 | 9.053e-11 | NOS3 |
| ENSG00000164871 | 2.971 | 0.0183 | 0.03039 | SPAG11B |
| ENSG00000164877 | -1.065 | 3.369e-12 | 1.298e-11 | MICALL2 |
| ENSG00000164896 | -1.693 | 2.959e-51 | 5.488e-50 | FASTK |
| ENSG00000164897 | -2.472 | 5.815e-80 | 2.381e-78 | TMUB1 |
| ENSG00000164920 | -2.559 | 5.651e-43 | 8.248e-42 | OSR2 |
| ENSG00000164930 | -1.747 | 4.085e-78 | 1.61e-76 | FZD6 |
| ENSG00000164949 | 4.783 | 1.282e-117 | 1.183e-115 | GEM |
| ENSG00000164967 | -3.347 | 4.958e-19 | 2.731e-18 | RPP25L |
| ENSG00000164970 | 2.503 | 1.233e-147 | 1.865e-145 | FAM219A |
| ENSG00000164972 | 4.173 | 9.901e-10 | 3.277e-09 | C9orf24 |
| ENSG00000164976 | -1.017 | 6.027e-11 | 2.157e-10 | KIAA1161 |
| ENSG00000164985 | -1.696 | 4.856e-15 | 2.193e-14 | PSIP1 |
| ENSG00000164989 | -1.596 | 8.275e-05 | 0.0001841 | CCDC171 |
| ENSG00000165006 | 1.087 | 1.189e-31 | 1.144e-30 | UBAP1 |
| ENSG00000165025 | -2.303 | 1.489e-48 | 2.579e-47 | SYK |
| ENSG00000165029 | 1.421 | 1.708e-14 | 7.5e-14 | ABCA1 |
| ENSG00000165060 | -1.868 | 1.368e-19 | 7.747e-19 | FXN |
| ENSG00000165102 | -1.395 | 4.848e-27 | 3.833e-26 | HGSNAT |
| ENSG00000165105 | -1.009 | 2.535e-20 | 1.485e-19 | RASEF |
| ENSG00000165115 | -1.636 | 1.256e-10 | 4.403e-10 | KIF27 |
| ENSG00000165118 | -1.102 | 2.194e-09 | 7.066e-09 | C9orf64 |
| ENSG00000165125 | -1.929 | 3.557e-24 | 2.483e-23 | TRPV6 |
| ENSG00000165138 | -2.054 | 6.99e-84 | 3.215e-82 | ANKS6 |
| ENSG00000165156 | -1.275 | 3.96e-21 | 2.411e-20 | ZHX1 |
| ENSG00000165168 | -1.34 | 0.02153 | 0.0353 | CYBB |
| ENSG00000165181 | 1.375 | 0.003517 | 0.0065 | C9orf84 |
| ENSG00000165185 | -2.223 | 1.45e-44 | 2.229e-43 | KIAA1958 |
| ENSG00000165195 | 3.325 | 2.322e-132 | 2.804e-130 | PIGA |
| ENSG00000165215 | -1.691 | 7.445e-10 | 2.478e-09 | CLDN3 |
| ENSG00000165238 | -2.071 | 3.548e-65 | 1.013e-63 | WNK2 |
| ENSG00000165244 | -3.133 | 5.316e-80 | 2.182e-78 | ZNF367 |
| ENSG00000165275 | -1.053 | 6.922e-08 | 2.002e-07 | TRMT10B |
| ENSG00000165300 | -3.56 | 1.557e-05 | 3.699e-05 | SLITRK5 |
| ENSG00000165304 | -1.481 | 1.311e-52 | 2.53e-51 | MELK |
| ENSG00000165376 | 2.433 | 0.002191 | 0.004175 | CLDN2 |
| ENSG00000165388 | -6.172 | 4.422e-23 | 2.921e-22 | ZNF488 |
| ENSG00000165389 | -1.355 | 4.328e-20 | 2.506e-19 | SPTSSA |
| ENSG00000165424 | -1.648 | 2.091e-06 | 5.353e-06 | ZCCHC24 |
| ENSG00000165458 | -1.077 | 2.755e-31 | 2.604e-30 | INPPL1 |
| ENSG00000165474 | -2.759 | 1.896e-17 | 9.654e-17 | GJB2 |
| ENSG00000165480 | -2.113 | 5.131e-13 | 2.079e-12 | SKA3 |
| ENSG00000165495 | 5.197 | 2.432e-07 | 6.757e-07 | PKNOX2 |
| ENSG00000165501 | -1.605 | 2.699e-28 | 2.246e-27 | LRR1 |
| ENSG00000165506 | -2.863 | 2.614e-66 | 7.698e-65 | DNAAF2 |
| ENSG00000165507 | -1.988 | 1.198e-14 | 5.294e-14 | C10orf10 |
| ENSG00000165512 | -3.691 | 1.98e-187 | 5.449e-185 | ZNF22 |
| ENSG00000165516 | -1.39 | 6.225e-26 | 4.724e-25 | KLHDC2 |
| ENSG00000165521 | -1.555 | 1.596e-13 | 6.685e-13 | EML5 |
| ENSG00000165527 | -1.693 | 2.535e-51 | 4.722e-50 | ARF6 |
| ENSG00000165533 | -2.149 | 3.471e-44 | 5.272e-43 | TTC8 |
| ENSG00000165572 | -2.838 | 5.92e-41 | 8.099e-40 | KBTBD6 |
| ENSG00000165606 | 4.224 | 2.466e-08 | 7.364e-08 | DRGX |
| ENSG00000165643 | 2.901 | 0.01131 | 0.01943 | SOHLH1 |
| ENSG00000165644 | -1.868 | 4.217e-25 | 3.067e-24 | COMTD1 |
| ENSG00000165655 | -2.389 | 7.033e-34 | 7.42e-33 | ZNF503 |
| ENSG00000165671 | -2.064 | 8.205e-110 | 6.436e-108 | NSD1 |
| ENSG00000165689 | -2.096 | 3.433e-28 | 2.843e-27 | SDCCAG3 |
| ENSG00000165695 | -1.257 | 0.01129 | 0.0194 | AK8 |
| ENSG00000165699 | 1.326 | 1.211e-37 | 1.487e-36 | TSC1 |
| ENSG00000165714 | -1.723 | 2.611e-11 | 9.574e-11 | LOH12CR1 |
| ENSG00000165716 | -2.409 | 9.649e-27 | 7.544e-26 | FAM69B |
| ENSG00000165724 | -2.329 | 7.578e-48 | 1.281e-46 | ZMYND19 |
| ENSG00000165730 | -4.085 | 2.94e-35 | 3.292e-34 | STOX1 |
| ENSG00000165732 | 1.186 | 5.549e-41 | 7.628e-40 | DDX21 |
| ENSG00000165752 | -1.111 | 3.994e-06 | 9.996e-06 | STK32C |
| ENSG00000165757 | -2.264 | 1.056e-119 | 1.036e-117 | KIAA1462 |
| ENSG00000165792 | -1.336 | 2.874e-15 | 1.312e-14 | METTL17 |
| ENSG00000165795 | -1.558 | 8.795e-10 | 2.917e-09 | NDRG2 |
| ENSG00000165801 | -2.502 | 5.09e-15 | 2.294e-14 | ARHGEF40 |
| ENSG00000165805 | 3.323 | 0.005393 | 0.009728 | C12orf50 |
| ENSG00000165807 | 2.278 | 0.02048 | 0.03371 | PPP1R36 |
| ENSG00000165810 | -1.676 | 7.419e-25 | 5.336e-24 | BTNL9 |
| ENSG00000165813 | 1.602 | 1.637e-18 | 8.819e-18 | C10orf118 |
| ENSG00000165837 | 1.696 | 0.001046 | 0.002071 | FAM194B |
| ENSG00000165879 | -2.022 | 0.0008038 | 0.00161 | FRAT1 |
| ENSG00000165887 | -1.502 | 1.497e-16 | 7.283e-16 | ANKRD2 |
| ENSG00000165891 | -1.968 | 5.737e-27 | 4.528e-26 | E2F7 |
| ENSG00000165905 | -2.373 | 3.958e-09 | 1.252e-08 | GYLTL1B |
| ENSG00000165912 | -2.974 | 9.442e-93 | 5.354e-91 | PACSIN3 |
| ENSG00000165915 | -1.48 | 8.35e-21 | 4.997e-20 | SLC39A13 |
| ENSG00000165917 | -1.177 | 0.02344 | 0.03816 | RAPSN |
| ENSG00000165923 | -1.934 | 5.579e-05 | 0.0001264 | AGBL2 |
| ENSG00000165934 | -1.491 | 7.482e-47 | 1.239e-45 | CPSF2 |
| ENSG00000165983 | -1.315 | 3.139e-18 | 1.666e-17 | PTER |
| ENSG00000165985 | -1.049 | 0.02164 | 0.03546 | C1QL3 |
| ENSG00000165997 | 2.422 | 2.284e-74 | 8.228e-73 | ARL5B |
| ENSG00000166002 | -2.082 | 4.276e-07 | 1.164e-06 | SMCO4 |
| ENSG00000166025 | -1.36 | 3.741e-53 | 7.318e-52 | AMOTL1 |
| ENSG00000166046 | 2.928 | 2.887e-57 | 6.393e-56 | TCP11L2 |
| ENSG00000166068 | -1.187 | 2.867e-18 | 1.526e-17 | SPRED1 |
| ENSG00000166086 | -1.378 | 0.0006952 | 0.001402 | JAM3 |
| ENSG00000166106 | -5.601 | 7.509e-10 | 2.499e-09 | ADAMTS15 |
| ENSG00000166130 | -1.716 | 3.069e-16 | 1.473e-15 | IKBIP |
| ENSG00000166133 | -1.549 | 3.069e-07 | 8.456e-07 | RPUSD2 |
| ENSG00000166145 | -1.402 | 6.483e-44 | 9.804e-43 | SPINT1 |
| ENSG00000166159 | 5.874 | 6.32e-09 | 1.972e-08 | LRTM2 |
| ENSG00000166166 | -1.373 | 2.111e-19 | 1.182e-18 | TRMT61A |
| ENSG00000166169 | -1.921 | 6.102e-27 | 4.809e-26 | POLL |
| ENSG00000166170 | -1.533 | 1.584e-47 | 2.661e-46 | BAG5 |
| ENSG00000166188 | -1.977 | 1.098e-29 | 9.712e-29 | ZNF319 |
| ENSG00000166189 | -4.342 | 3.991e-69 | 1.245e-67 | HPS6 |
| ENSG00000166200 | 1.247 | 3.877e-34 | 4.148e-33 | COPS2 |
| ENSG00000166206 | -1.871 | 9.562e-19 | 5.203e-18 | GABRB3 |
| ENSG00000166225 | 1.012 | 1.61e-14 | 7.075e-14 | FRS2 |
| ENSG00000166257 | 2.343 | 0.003367 | 0.006238 | SCN3B |
| ENSG00000166260 | -1.166 | 1.291e-11 | 4.814e-11 | COX11 |
| ENSG00000166263 | -1.033 | 1.047e-09 | 3.457e-09 | STXBP4 |
| ENSG00000166265 | 4.337 | 0.0002112 | 0.0004511 | CYYR1 |
| ENSG00000166278 | 1.885 | 1.468e-05 | 3.495e-05 | C2 |
| ENSG00000166311 | -2.507 | 2.623e-24 | 1.845e-23 | SMPD1 |
| ENSG00000166317 | 1.702 | 0.006089 | 0.0109 | SYNPO2L |
| ENSG00000166321 | 1.473 | 1.374e-11 | 5.113e-11 | NUDT13 |
| ENSG00000166343 | -3.157 | 7.627e-06 | 1.864e-05 | MSS51 |
| ENSG00000166348 | -1.506 | 1.708e-19 | 9.632e-19 | USP54 |
| ENSG00000166359 | -2.137 | 0.005725 | 0.01028 | WDR88 |
| ENSG00000166387 | -1.094 | 0.0001581 | 0.0003417 | PPFIBP2 |
| ENSG00000166391 | 4.155 | 0.0001629 | 0.0003515 | MOGAT2 |
| ENSG00000166396 | -1.49 | 7.509e-20 | 4.305e-19 | SERPINB7 |
| ENSG00000166401 | 1.754 | 1.418e-23 | 9.621e-23 | SERPINB8 |
| ENSG00000166436 | -2.158 | 1.786e-32 | 1.779e-31 | TRIM66 |
| ENSG00000166444 | -2.209 | 1.466e-30 | 1.339e-29 | ST5 |
| ENSG00000166446 | -1.429 | 8.581e-07 | 2.28e-06 | CDYL2 |
| ENSG00000166448 | 3.296 | 0.01023 | 0.0177 | TMEM130 |
| ENSG00000166450 | -2.668 | 2.629e-51 | 4.893e-50 | PRTG |
| ENSG00000166452 | -1.234 | 1.698e-09 | 5.515e-09 | AKIP1 |
| ENSG00000166455 | 1.055 | 1.174e-05 | 2.822e-05 | C16orf46 |
| ENSG00000166471 | 1.743 | 1.031e-41 | 1.452e-40 | TMEM41B |
| ENSG00000166478 | 2.108 | 1.31e-44 | 2.019e-43 | ZNF143 |
| ENSG00000166484 | -1.06 | 2.486e-05 | 5.802e-05 | MAPK7 |
| ENSG00000166507 | -3.704 | 7.662e-34 | 8.058e-33 | NDST2 |
| ENSG00000166510 | -1.242 | 3.602e-08 | 1.063e-07 | CCDC68 |
| ENSG00000166529 | -1.892 | 9.13e-15 | 4.072e-14 | ZSCAN21 |
| ENSG00000166532 | -1.202 | 2.936e-11 | 1.072e-10 | RIMKLB |
| ENSG00000166535 | 2.447 | 0.004392 | 0.00801 | A2ML1 |
| ENSG00000166548 | -3.243 | 6.875e-38 | 8.505e-37 | TK2 |
| ENSG00000166592 | 5.701 | 4.149e-45 | 6.479e-44 | RRAD |
| ENSG00000166598 | 1.051 | 8.523e-29 | 7.285e-28 | HSP90B1 |
| ENSG00000166670 | 11.08 | 7.082e-39 | 9.088e-38 | MMP10 |
| ENSG00000166676 | 5.167 | 3.046e-66 | 8.956e-65 | TVP23A |
| ENSG00000166685 | -1.71 | 5.477e-24 | 3.798e-23 | COG1 |
| ENSG00000166704 | -2.137 | 9.803e-27 | 7.657e-26 | ZNF606 |
| ENSG00000166707 | 2.66 | 0.01573 | 0.02643 | ZCCHC18 |
| ENSG00000166747 | 1.098 | 8.226e-42 | 1.165e-40 | AP1G1 |
| ENSG00000166793 | 4.069 | 2.896e-20 | 1.689e-19 | YPEL4 |
| ENSG00000166797 | -1.351 | 1.23e-15 | 5.722e-15 | FAM96A |
| ENSG00000166800 | 1.988 | 1.754e-05 | 4.148e-05 | LDHAL6A |
| ENSG00000166801 | -2.699 | 5.538e-122 | 5.738e-120 | FAM111A |
| ENSG00000166813 | -3.226 | 4.419e-30 | 3.954e-29 | KIF7 |
| ENSG00000166822 | -1.38 | 1.908e-25 | 1.417e-24 | TMEM170A |
| ENSG00000166823 | -4.854 | 3.3e-07 | 9.077e-07 | MESP1 |
| ENSG00000166825 | -1.585 | 1.207e-20 | 7.168e-20 | ANPEP |
| ENSG00000166831 | -1.892 | 0.004783 | 0.008679 | RBPMS2 |
| ENSG00000166845 | -1.463 | 5.358e-18 | 2.813e-17 | C18orf54 |
| ENSG00000166851 | -3.506 | 4.068e-218 | 1.846e-215 | PLK1 |
| ENSG00000166860 | -4.546 | 3.284e-80 | 1.354e-78 | ZBTB39 |
| ENSG00000166881 | -1.051 | 7.618e-26 | 5.76e-25 | TMEM194A |
| ENSG00000166888 | -1.216 | 8.971e-37 | 1.057e-35 | STAT6 |
| ENSG00000166896 | -1.757 | 1.546e-10 | 5.383e-10 | XRCC6BP1 |
| ENSG00000166897 | -2.258 | 5.656e-41 | 7.77e-40 | ELFN2 |
| ENSG00000166900 | 1.768 | 1.51e-83 | 6.851e-82 | STX3 |
| ENSG00000166902 | -2.141 | 3.518e-30 | 3.159e-29 | MRPL16 |
| ENSG00000166922 | 3.724 | 1.488e-17 | 7.615e-17 | SCG5 |
| ENSG00000166938 | -2.546 | 2.025e-69 | 6.389e-68 | DIS3L |
| ENSG00000166949 | -1.742 | 7.902e-62 | 2.038e-60 | SMAD3 |
| ENSG00000166965 | -2.085 | 1.943e-15 | 8.944e-15 | RCCD1 |
| ENSG00000166986 | 1.223 | 1.037e-31 | 1.001e-30 | MARS |
| ENSG00000166997 | -1.663 | 1.684e-07 | 4.734e-07 | CNPY4 |
| ENSG00000167034 | 1.107 | 1.318e-11 | 4.911e-11 | NKX3-1 |
| ENSG00000167037 | -2.067 | 1.012e-08 | 3.107e-08 | SGSM1 |
| ENSG00000167065 | -1.848 | 1.22e-14 | 5.394e-14 | DUSP18 |
| ENSG00000167074 | -1.817 | 1.266e-14 | 5.587e-14 | TEF |
| ENSG00000167100 | 3.384 | 0.000148 | 0.0003209 | SAMD14 |
| ENSG00000167103 | 2.832 | 8.691e-13 | 3.477e-12 | PIP5KL1 |
| ENSG00000167105 | 1.853 | 1.309e-27 | 1.059e-26 | TMEM92 |
| ENSG00000167112 | -1.588 | 3.921e-13 | 1.602e-12 | TRUB2 |
| ENSG00000167113 | -1.118 | 2.925e-13 | 1.204e-12 | COQ4 |
| ENSG00000167114 | -1.9 | 2.834e-38 | 3.548e-37 | SLC27A4 |
| ENSG00000167123 | -1.26 | 1.334e-23 | 9.067e-23 | CERCAM |
| ENSG00000167136 | -2.008 | 3.1e-10 | 1.059e-09 | ENDOG |
| ENSG00000167165 | -1.245 | 5.119e-43 | 7.484e-42 | UGT1A6 |
| ENSG00000167182 | 1.143 | 6.351e-05 | 0.0001431 | SP2 |
| ENSG00000167183 | -5.214 | 2.999e-26 | 2.303e-25 | PRR15L |
| ENSG00000167191 | -2.012 | 3.073e-51 | 5.693e-50 | GPRC5B |
| ENSG00000167195 | 5.527 | 1.073e-08 | 3.286e-08 | GOLGA6C |
| ENSG00000167196 | -1.532 | 6.277e-22 | 3.953e-21 | FBXO22 |
| ENSG00000167202 | -1.584 | 3.387e-36 | 3.913e-35 | TBC1D2B |
| ENSG00000167207 | 2.188 | 4.955e-05 | 0.0001127 | NOD2 |
| ENSG00000167257 | -1.854 | 4.008e-26 | 3.063e-25 | RNF214 |
| ENSG00000167264 | -1.211 | 5.164e-16 | 2.448e-15 | DUS2 |
| ENSG00000167272 | -1.82 | 4.731e-18 | 2.49e-17 | POP5 |
| ENSG00000167281 | 1.956 | 0.02328 | 0.03793 | RBFOX3 |
| ENSG00000167333 | -1.385 | 1.385e-14 | 6.101e-14 | TRIM68 |
| ENSG00000167363 | -1.566 | 2.39e-05 | 5.589e-05 | FN3K |
| ENSG00000167383 | 2.64 | 4.16e-112 | 3.39e-110 | ZNF229 |
| ENSG00000167397 | -1.09 | 2.181e-13 | 9.056e-13 | VKORC1 |
| ENSG00000167476 | 1.564 | 5.67e-05 | 0.0001284 | JSRP1 |
| ENSG00000167513 | -2.235 | 2.85e-69 | 8.926e-68 | CDT1 |
| ENSG00000167522 | 2.235 | 1.569e-38 | 1.982e-37 | ANKRD11 |
| ENSG00000167523 | -2.193 | 4.462e-22 | 2.83e-21 | SPATA33 |
| ENSG00000167524 | -1.308 | 3.062e-09 | 9.765e-09 | SGK494 |
| ENSG00000167535 | -2.469 | 1.394e-28 | 1.18e-27 | CACNB3 |
| ENSG00000167536 | -2.314 | 5.859e-07 | 1.576e-06 | DHRS13 |
| ENSG00000167543 | -1.625 | 5.743e-16 | 2.713e-15 | TP53I13 |
| ENSG00000167549 | -2.222 | 8.411e-24 | 5.782e-23 | CORO6 |
| ENSG00000167565 | 1.419 | 1.353e-16 | 6.607e-16 | SERTAD3 |
| ENSG00000167604 | -1.301 | 1.509e-06 | 3.916e-06 | NFKBID |
| ENSG00000167654 | 4.23 | 8.621e-05 | 0.0001917 | ATCAY |
| ENSG00000167656 | -1.751 | 1.707e-22 | 1.101e-21 | LY6D |
| ENSG00000167670 | -2.76 | 4.066e-58 | 9.224e-57 | CHAF1A |
| ENSG00000167674 | -1.256 | 6.155e-20 | 3.539e-19 | HDGFRP2 |
| ENSG00000167695 | -1.946 | 1.632e-40 | 2.202e-39 | FAM57A |
| ENSG00000167700 | -1.613 | 5.951e-08 | 1.727e-07 | MFSD3 |
| ENSG00000167702 | -1.464 | 5.963e-35 | 6.589e-34 | KIFC2 |
| ENSG00000167703 | -1.887 | 4.023e-19 | 2.228e-18 | SLC43A2 |
| ENSG00000167705 | -1.464 | 1.028e-05 | 2.484e-05 | RILP |
| ENSG00000167723 | 4.21 | 5.367e-90 | 2.815e-88 | TRPV3 |
| ENSG00000167747 | -1.795 | 8.866e-37 | 1.046e-35 | C19orf48 |
| ENSG00000167759 | 2.702 | 0.02884 | 0.04618 | KLK13 |
| ENSG00000167767 | -1.942 | 2.582e-99 | 1.641e-97 | KRT80 |
| ENSG00000167771 | -1.991 | 0.002584 | 0.004875 | RCOR2 |
| ENSG00000167775 | -1.179 | 8.226e-09 | 2.546e-08 | CD320 |
| ENSG00000167799 | -1.528 | 1.478e-06 | 3.843e-06 | NUDT8 |
| ENSG00000167807 | -2.989 | 0.000169 | 0.0003643 | CTD-2369P2.10 |
| ENSG00000167840 | -1.55 | 5.064e-13 | 2.055e-12 | ZNF232 |
| ENSG00000167861 | -1.298 | 2.918e-32 | 2.886e-31 | HID1 |
| ENSG00000167862 | -1.09 | 3.741e-10 | 1.269e-09 | ICT1 |
| ENSG00000167874 | 2.402 | 0.0001541 | 0.0003334 | TMEM88 |
| ENSG00000167880 | -1.571 | 2.815e-44 | 4.292e-43 | EVPL |
| ENSG00000167889 | -1.137 | 1.433e-10 | 5.004e-10 | MGAT5B |
| ENSG00000167914 | 3.147 | 0.0001577 | 0.000341 | GSDMA |
| ENSG00000167930 | -1.042 | 9.023e-29 | 7.708e-28 | ITFG3 |
| ENSG00000167964 | -2.075 | 5.048e-14 | 2.165e-13 | RAB26 |
| ENSG00000167965 | -1.119 | 4.364e-20 | 2.526e-19 | MLST8 |
| ENSG00000167967 | -1.736 | 5.894e-36 | 6.74e-35 | E4F1 |
| ENSG00000167968 | -1.413 | 0.01877 | 0.03109 | DNASE1L2 |
| ENSG00000167972 | -1.456 | 6.294e-18 | 3.291e-17 | ABCA3 |
| ENSG00000167992 | 2.818 | 0.0001392 | 0.0003028 | VWCE |
| ENSG00000167994 | -1.519 | 1.189e-05 | 2.855e-05 | RAB3IL1 |
| ENSG00000167995 | 6.131 | 1.232e-07 | 3.502e-07 | BEST1 |
| ENSG00000168003 | 1.193 | 1.657e-09 | 5.39e-09 | SLC3A2 |
| ENSG00000168005 | -1.256 | 1.419e-14 | 6.245e-14 | C11orf84 |
| ENSG00000168010 | -1.594 | 5.533e-15 | 2.489e-14 | ATG16L2 |
| ENSG00000168016 | -2.131 | 6.465e-16 | 3.049e-15 | TRANK1 |
| ENSG00000168040 | -2.297 | 7.303e-36 | 8.316e-35 | FADD |
| ENSG00000168061 | -4.141 | 1.788e-48 | 3.084e-47 | SAC3D1 |
| ENSG00000168062 | 1.777 | 0.0009161 | 0.001824 | BATF2 |
| ENSG00000168077 | -2.052 | 2.904e-16 | 1.396e-15 | SCARA3 |
| ENSG00000168078 | -1.829 | 1.904e-45 | 3.012e-44 | PBK |
| ENSG00000168079 | 2.678 | 0.0001067 | 0.0002354 | SCARA5 |
| ENSG00000168101 | -1.3 | 2.575e-12 | 1e-11 | NUDT16L1 |
| ENSG00000168116 | -2.28 | 1.221e-20 | 7.245e-20 | KIAA1586 |
| ENSG00000168137 | 1.111 | 6.781e-17 | 3.362e-16 | SETD5 |
| ENSG00000168152 | -1.671 | 4.173e-10 | 1.412e-09 | THAP9 |
| ENSG00000168159 | -1.561 | 3.017e-17 | 1.523e-16 | RNF187 |
| ENSG00000168209 | 2.663 | 2.075e-24 | 1.464e-23 | DDIT4 |
| ENSG00000168259 | 1.149 | 5.283e-17 | 2.636e-16 | DNAJC7 |
| ENSG00000168264 | -1.507 | 1.731e-28 | 1.456e-27 | IRF2BP2 |
| ENSG00000168282 | -1.869 | 5.292e-38 | 6.587e-37 | MGAT2 |
| ENSG00000168283 | -1.083 | 1.398e-31 | 1.341e-30 | BMI1 |
| ENSG00000168286 | -1.185 | 7.094e-09 | 2.205e-08 | THAP11 |
| ENSG00000168300 | -1.049 | 0.000109 | 0.0002402 | PCMTD1 |
| ENSG00000168303 | -1.049 | 1.268e-14 | 5.596e-14 | MPLKIP |
| ENSG00000168334 | 4.516 | 2.206e-05 | 5.175e-05 | XIRP1 |
| ENSG00000168348 | 4.367 | 6.957e-05 | 0.0001561 | INSM2 |
| ENSG00000168374 | 1.288 | 4.554e-53 | 8.857e-52 | ARF4 |
| ENSG00000168386 | 1.031 | 1.124e-05 | 2.705e-05 | FILIP1L |
| ENSG00000168393 | -1.54 | 1.819e-12 | 7.135e-12 | DTYMK |
| ENSG00000168395 | -1.379 | 7.155e-20 | 4.105e-19 | ING5 |
| ENSG00000168427 | -1.193 | 0.0002324 | 0.0004943 | KLHL30 |
| ENSG00000168439 | 1.378 | 1.769e-41 | 2.473e-40 | STIP1 |
| ENSG00000168453 | -1.112 | 6.505e-15 | 2.918e-14 | HR |
| ENSG00000168491 | 1.249 | 0.005583 | 0.01005 | CCDC110 |
| ENSG00000168496 | -2.397 | 5.429e-56 | 1.148e-54 | FEN1 |
| ENSG00000168502 | -1.597 | 2.598e-28 | 2.164e-27 | SOGA2 |
| ENSG00000168517 | -2.684 | 1.231e-12 | 4.879e-12 | HEXIM2 |
| ENSG00000168530 | 4.796 | 2.976e-06 | 7.525e-06 | MYL1 |
| ENSG00000168538 | -1.104 | 3.426e-22 | 2.186e-21 | TRAPPC11 |
| ENSG00000168539 | 4.41 | 2.38e-05 | 5.568e-05 | CHRM1 |
| ENSG00000168569 | -2.693 | 1.283e-42 | 1.85e-41 | TMEM223 |
| ENSG00000168589 | 2.761 | 0.005043 | 0.00913 | DYNLRB2 |
| ENSG00000168591 | 1.412 | 1.788e-17 | 9.119e-17 | TMUB2 |
| ENSG00000168621 | 4.841 | 2.007e-06 | 5.149e-06 | GDNF |
| ENSG00000168658 | 3.024 | 2.618e-06 | 6.648e-06 | VWA3B |
| ENSG00000168661 | -3.217 | 5.446e-25 | 3.944e-24 | ZNF30 |
| ENSG00000168672 | -1.263 | 1.822e-19 | 1.025e-18 | FAM84B |
| ENSG00000168675 | 3.35 | 1.134e-09 | 3.737e-09 | LDLRAD4 |
| ENSG00000168685 | 1.156 | 4.959e-10 | 1.67e-09 | IL7R |
| ENSG00000168746 | 4.164 | 0.0001022 | 0.0002258 | C20orf62 |
| ENSG00000168758 | -3.217 | 1.108e-79 | 4.503e-78 | SEMA4C |
| ENSG00000168763 | -2.247 | 6.994e-46 | 1.119e-44 | CNNM3 |
| ENSG00000168778 | -1.785 | 2.06e-15 | 9.466e-15 | TCTN2 |
| ENSG00000168781 | -1.082 | 8.519e-16 | 3.99e-15 | PPIP5K1 |
| ENSG00000168785 | -1.43 | 4.704e-12 | 1.801e-11 | TSPAN5 |
| ENSG00000168792 | -3.311 | 2.885e-43 | 4.271e-42 | ABHD15 |
| ENSG00000168795 | 1.328 | 1.997e-24 | 1.411e-23 | ZBTB5 |
| ENSG00000168802 | -1.37 | 8.34e-15 | 3.725e-14 | CHTF8 |
| ENSG00000168803 | -1.296 | 3.092e-10 | 1.057e-09 | ADAL |
| ENSG00000168806 | -1.696 | 1.949e-15 | 8.967e-15 | LCMT2 |
| ENSG00000168807 | -2.18 | 7.399e-113 | 6.179e-111 | SNTB2 |
| ENSG00000168826 | 2.34 | 5.749e-53 | 1.117e-51 | ZBTB49 |
| ENSG00000168887 | -1.186 | 2.36e-16 | 1.139e-15 | C2orf68 |
| ENSG00000168890 | -1.264 | 4.078e-07 | 1.112e-06 | TMEM150A |
| ENSG00000168906 | -1.782 | 6.966e-93 | 3.991e-91 | MAT2A |
| ENSG00000168907 | -3.045 | 2.098e-07 | 5.852e-07 | PLA2G4F |
| ENSG00000168916 | -2.303 | 0.0001786 | 0.0003841 | ZNF608 |
| ENSG00000168925 | 2.695 | 0.004906 | 0.008892 | CTRB1 |
| ENSG00000168928 | 2.577 | 0.0005078 | 0.001041 | CTRB2 |
| ENSG00000168936 | -1.216 | 5.454e-12 | 2.08e-11 | TMEM129 |
| ENSG00000168944 | -1.574 | 1.586e-22 | 1.024e-21 | CEP120 |
| ENSG00000168952 | -1.951 | 3.071e-08 | 9.106e-08 | STXBP6 |
| ENSG00000168970 | -1.429 | 2.253e-06 | 5.75e-06 | JMJD7-PLA2G4B |
| ENSG00000169016 | 1.466 | 2.593e-39 | 3.355e-38 | E2F6 |
| ENSG00000169018 | -1.44 | 4.647e-36 | 5.335e-35 | FEM1B |
| ENSG00000169021 | -1.03 | 4.324e-09 | 1.364e-08 | UQCRFS1 |
| ENSG00000169047 | -2.736 | 2.927e-140 | 3.962e-138 | IRS1 |
| ENSG00000169067 | 1.03 | 0.0007481 | 0.001504 | ACTBL2 |
| ENSG00000169083 | 2.176 | 1.803e-06 | 4.645e-06 | AR |
| ENSG00000169085 | -2.507 | 6.729e-05 | 0.0001511 | C8orf46 |
| ENSG00000169105 | -3.132 | 1.993e-38 | 2.507e-37 | CHST14 |
| ENSG00000169129 | -2.263 | 1.622e-60 | 4.033e-59 | AFAP1L2 |
| ENSG00000169131 | -1.236 | 1.771e-06 | 4.569e-06 | ZNF354A |
| ENSG00000169136 | -3.961 | 3.945e-183 | 1.003e-180 | ATF5 |
| ENSG00000169155 | 3.36 | 0 | 0 | ZBTB43 |
| ENSG00000169169 | 2.874 | 1.06e-05 | 2.557e-05 | CPT1C |
| ENSG00000169174 | -1.029 | 1.222e-07 | 3.476e-07 | PCSK9 |
| ENSG00000169181 | 3.725 | 0.001056 | 0.00209 | GSG1L |
| ENSG00000169184 | -2.493 | 0.0002383 | 0.0005063 | MN1 |
| ENSG00000169188 | -1.775 | 1.926e-35 | 2.17e-34 | APEX2 |
| ENSG00000169203 | 1.496 | 2.097e-06 | 5.368e-06 | RP11-231C14.4 |
| ENSG00000169213 | 1.234 | 7.453e-19 | 4.071e-18 | RAB3B |
| ENSG00000169220 | -2.728 | 1.508e-05 | 3.587e-05 | RGS14 |
| ENSG00000169242 | -2.227 | 7.149e-26 | 5.415e-25 | EFNA1 |
| ENSG00000169246 | 1.044 | 0.03012 | 0.04809 | NPIPB3 |
| ENSG00000169255 | -1.597 | 4.376e-17 | 2.193e-16 | B3GALNT1 |
| ENSG00000169258 | -1.233 | 2.353e-12 | 9.169e-12 | GPRIN1 |
| ENSG00000169297 | -2.394 | 5.871e-29 | 5.043e-28 | NR0B1 |
| ENSG00000169371 | -1.515 | 4.324e-22 | 2.744e-21 | SNUPN |
| ENSG00000169402 | 2.454 | 0.0009338 | 0.001859 | RSPH10B2 |
| ENSG00000169410 | -1.591 | 1.018e-38 | 1.298e-37 | PTPN9 |
| ENSG00000169418 | 2.022 | 0.000426 | 0.000881 | NPR1 |
| ENSG00000169427 | 3.92 | 0.000444 | 0.000916 | KCNK9 |
| ENSG00000169429 | 7.366 | 0 | 0 | IL8 |
| ENSG00000169469 | 1.838 | 0.006548 | 0.01167 | SPRR1B |
| ENSG00000169499 | -1.426 | 8.669e-21 | 5.184e-20 | PLEKHA2 |
| ENSG00000169504 | 1.371 | 3.192e-65 | 9.143e-64 | CLIC4 |
| ENSG00000169519 | -1.918 | 1.286e-40 | 1.736e-39 | METTL15 |
| ENSG00000169554 | 1.315 | 0.0003994 | 0.0008289 | ZEB2 |
| ENSG00000169570 | -1.871 | 1.534e-18 | 8.282e-18 | DTWD2 |
| ENSG00000169594 | 2.667 | 4.398e-17 | 2.204e-16 | BNC1 |
| ENSG00000169598 | -1.314 | 6.811e-07 | 1.823e-06 | DFFB |
| ENSG00000169607 | -2.244 | 8.383e-65 | 2.365e-63 | CKAP2L |
| ENSG00000169629 | 1.221 | 2.456e-15 | 1.124e-14 | RGPD8 |
| ENSG00000169660 | -1.012 | 1.401e-07 | 3.971e-07 | HEXDC |
| ENSG00000169676 | 3.161 | 0.02602 | 0.04204 | DRD5 |
| ENSG00000169679 | -2.166 | 2.221e-95 | 1.332e-93 | BUB1 |
| ENSG00000169683 | -2.874 | 8.514e-40 | 1.122e-38 | LRRC45 |
| ENSG00000169688 | 3.758 | 0.004323 | 0.007895 | MT1B |
| ENSG00000169689 | -1.369 | 7.161e-09 | 2.226e-08 | STRA13 |
| ENSG00000169710 | -1.092 | 8.776e-32 | 8.496e-31 | FASN |
| ENSG00000169718 | -1.201 | 3.869e-38 | 4.832e-37 | DUS1L |
| ENSG00000169733 | -1.003 | 5.93e-08 | 1.722e-07 | RFNG |
| ENSG00000169740 | -1.455 | 3.84e-06 | 9.621e-06 | ZNF32 |
| ENSG00000169752 | -1.606 | 1.196e-06 | 3.138e-06 | NRG4 |
| ENSG00000169756 | 1.204 | 1.851e-24 | 1.312e-23 | LIMS1 |
| ENSG00000169760 | 3.304 | 0.001083 | 0.002143 | NLGN1 |
| ENSG00000169814 | -1.281 | 1.01e-10 | 3.558e-10 | BTD |
| ENSG00000169826 | 2.322 | 3.067e-71 | 1.013e-69 | CSGALNACT2 |
| ENSG00000169851 | -1.277 | 1.229e-39 | 1.61e-38 | PCDH7 |
| ENSG00000169860 | -1.615 | 0.02096 | 0.03442 | P2RY1 |
| ENSG00000169871 | -1.425 | 1.188e-19 | 6.749e-19 | TRIM56 |
| ENSG00000169884 | -2.515 | 8.938e-07 | 2.371e-06 | WNT10B |
| ENSG00000169894 | -2.654 | 0.0001818 | 0.0003907 | MUC3A |
| ENSG00000169896 | 1.9 | 0.0003184 | 0.000668 | ITGAM |
| ENSG00000169902 | -1.46 | 1.688e-08 | 5.097e-08 | TPST1 |
| ENSG00000169908 | 2.513 | 4.009e-137 | 5.216e-135 | TM4SF1 |
| ENSG00000169925 | -2.925 | 1.266e-115 | 1.095e-113 | BRD3 |
| ENSG00000169946 | 2.867 | 5.436e-23 | 3.577e-22 | ZFPM2 |
| ENSG00000169957 | -1.957 | 5.496e-40 | 7.282e-39 | ZNF768 |
| ENSG00000169964 | -2.172 | 1.025e-12 | 4.091e-12 | TMEM42 |
| ENSG00000169972 | -1.077 | 3.868e-13 | 1.581e-12 | PUSL1 |
| ENSG00000169991 | -1.672 | 5.464e-17 | 2.724e-16 | IFFO2 |
| ENSG00000169992 | -1.131 | 2.26e-14 | 9.858e-14 | NLGN2 |
| ENSG00000169994 | 3.574 | 2.338e-08 | 6.994e-08 | MYO7B |
| ENSG00000170006 | 1.409 | 2.097e-19 | 1.175e-18 | TMEM154 |
| ENSG00000170027 | 1.197 | 1.858e-35 | 2.097e-34 | YWHAG |
| ENSG00000170037 | -1.489 | 2.638e-35 | 2.958e-34 | CNTROB |
| ENSG00000170044 | 3.204 | 0.01801 | 0.02996 | ZPLD1 |
| ENSG00000170074 | 5.257 | 1.5e-11 | 5.574e-11 | FAM153A |
| ENSG00000170113 | -1.617 | 8.007e-44 | 1.207e-42 | NIPA1 |
| ENSG00000170145 | -1.389 | 1.55e-35 | 1.754e-34 | SIK2 |
| ENSG00000170190 | -2.534 | 1.413e-29 | 1.246e-28 | SLC16A5 |
| ENSG00000170191 | -1.949 | 1.128e-41 | 1.586e-40 | NANP |
| ENSG00000170214 | -2.296 | 9.267e-21 | 5.53e-20 | ADRA1B |
| ENSG00000170222 | 1.593 | 3.779e-09 | 1.197e-08 | ADPRM |
| ENSG00000170231 | -2.963 | 0.01757 | 0.02927 | FABP6 |
| ENSG00000170234 | -1.044 | 1.307e-09 | 4.286e-09 | PWWP2A |
| ENSG00000170264 | -1.979 | 5.386e-22 | 3.404e-21 | FAM161A |
| ENSG00000170271 | 1.463 | 1.794e-10 | 6.217e-10 | FAXDC2 |
| ENSG00000170291 | -1.209 | 4.317e-22 | 2.741e-21 | ELP5 |
| ENSG00000170312 | -2.022 | 5.382e-58 | 1.216e-56 | CDK1 |
| ENSG00000170315 | 1.322 | 5.949e-37 | 7.077e-36 | UBB |
| ENSG00000170345 | 3.946 | 4.084e-298 | 2.981e-295 | FOS |
| ENSG00000170365 | -2.493 | 2.562e-33 | 2.634e-32 | SMAD1 |
| ENSG00000170381 | -1.421 | 4.108e-06 | 1.028e-05 | SEMA3E |
| ENSG00000170382 | -2.454 | 4.546e-30 | 4.065e-29 | LRRN2 |
| ENSG00000170385 | 2.555 | 4.16e-98 | 2.586e-96 | SLC30A1 |
| ENSG00000170412 | -1.771 | 3.425e-06 | 8.619e-06 | GPRC5C |
| ENSG00000170417 | -1.042 | 6.349e-07 | 1.703e-06 | TMEM182 |
| ENSG00000170425 | -2.689 | 7.49e-54 | 1.495e-52 | ADORA2B |
| ENSG00000170426 | 3.218 | 0.01647 | 0.02756 | SDR9C7 |
| ENSG00000170442 | -1.38 | 0.002455 | 0.004644 | KRT86 |
| ENSG00000170456 | -2.433 | 1.608e-44 | 2.47e-43 | DENND5B |
| ENSG00000170465 | 1.7 | 1.728e-05 | 4.087e-05 | KRT6C |
| ENSG00000170473 | -1.158 | 1.158e-14 | 5.123e-14 | WIBG |
| ENSG00000170482 | 2.395 | 7.464e-16 | 3.507e-15 | SLC23A1 |
| ENSG00000170500 | -1.704 | 2.543e-45 | 3.993e-44 | LONRF2 |
| ENSG00000170522 | -1.84 | 1.275e-48 | 2.211e-47 | ELOVL6 |
| ENSG00000170540 | -1.419 | 8.279e-22 | 5.193e-21 | ARL6IP1 |
| ENSG00000170542 | 2.972 | 0.0286 | 0.04585 | SERPINB9 |
| ENSG00000170545 | -1.23 | 5.66e-20 | 3.261e-19 | SMAGP |
| ENSG00000170577 | -3.51 | 1.113e-20 | 6.625e-20 | SIX2 |
| ENSG00000170579 | 3.928 | 0.0003797 | 0.0007895 | DLGAP1 |
| ENSG00000170604 | -2.196 | 4.434e-39 | 5.712e-38 | IRF2BP1 |
| ENSG00000170606 | 1.315 | 1.55e-67 | 4.712e-66 | HSPA4 |
| ENSG00000170615 | 4.319 | 3.509e-19 | 1.95e-18 | SLC26A5 |
| ENSG00000170616 | 3.631 | 0.002413 | 0.004571 | SCRT1 |
| ENSG00000170619 | -1.777 | 2.697e-11 | 9.884e-11 | COMMD5 |
| ENSG00000170632 | -1.107 | 2.452e-15 | 1.122e-14 | ARMC10 |
| ENSG00000170638 | -1.911 | 7.271e-21 | 4.367e-20 | TRABD |
| ENSG00000170677 | -2.014 | 1.348e-62 | 3.586e-61 | SOCS6 |
| ENSG00000170683 | 1.858 | 0.019 | 0.03143 | OR10A3 |
| ENSG00000170684 | -1.708 | 8.316e-07 | 2.212e-06 | ZNF296 |
| ENSG00000170745 | -2.58 | 2.153e-40 | 2.885e-39 | KCNS3 |
| ENSG00000170779 | -2.404 | 5.63e-52 | 1.069e-50 | CDCA4 |
| ENSG00000170801 | 2.018 | 2.392e-34 | 2.586e-33 | HTRA3 |
| ENSG00000170802 | 2.779 | 5.484e-113 | 4.603e-111 | FOXN2 |
| ENSG00000170807 | 3.247 | 0.01871 | 0.03101 | LMOD2 |
| ENSG00000170835 | 1.532 | 0.006802 | 0.0121 | CEL |
| ENSG00000170854 | -2.062 | 1.255e-75 | 4.641e-74 | MINA |
| ENSG00000170855 | -1.133 | 1.225e-09 | 4.03e-09 | TRIAP1 |
| ENSG00000170871 | 1.491 | 5.271e-72 | 1.777e-70 | KIAA0232 |
| ENSG00000170873 | 1.367 | 1.129e-11 | 4.231e-11 | MTSS1 |
| ENSG00000170876 | -1.005 | 1.563e-26 | 1.213e-25 | TMEM43 |
| ENSG00000170890 | 3.669 | 0.001256 | 0.002464 | PLA2G1B |
| ENSG00000170909 | 1.633 | 0.009221 | 0.01607 | OSCAR |
| ENSG00000170915 | -3.651 | 1.693e-13 | 7.08e-13 | PAQR8 |
| ENSG00000170917 | -3.437 | 1.275e-15 | 5.924e-15 | NUDT6 |
| ENSG00000170949 | -1.007 | 1.042e-08 | 3.194e-08 | ZNF160 |
| ENSG00000170955 | -1.41 | 0.02219 | 0.03629 | PRKCDBP |
| ENSG00000170962 | -1.16 | 1.334e-10 | 4.667e-10 | PDGFD |
| ENSG00000170965 | 1.796 | 7.558e-07 | 2.017e-06 | PLAC1 |
| ENSG00000171004 | -1.545 | 6.336e-60 | 1.548e-58 | HS6ST2 |
| ENSG00000171016 | -1.725 | 2.024e-09 | 6.537e-09 | PYGO1 |
| ENSG00000171017 | -1.598 | 0.006139 | 0.01098 | LRRC8E |
| ENSG00000171044 | 2.231 | 0.01713 | 0.02858 | XKR6 |
| ENSG00000171056 | -1.746 | 5.814e-14 | 2.485e-13 | SOX7 |
| ENSG00000171103 | -1.47 | 7.898e-15 | 3.534e-14 | TRMT61B |
| ENSG00000171116 | -3.577 | 0.0007027 | 0.001416 | HSFX1 |
| ENSG00000171121 | -2.398 | 7.782e-15 | 3.484e-14 | KCNMB3 |
| ENSG00000171126 | -3.374 | 1.284e-08 | 3.908e-08 | KCNG3 |
| ENSG00000171129 | -3.281 | 0.0006399 | 0.001299 | HSFX2 |
| ENSG00000171130 | -1.432 | 1.873e-16 | 9.065e-16 | ATP6V0E2 |
| ENSG00000171135 | -1.621 | 2.72e-13 | 1.124e-12 | JAGN1 |
| ENSG00000171155 | -1.397 | 5.029e-12 | 1.922e-11 | C1GALT1C1 |
| ENSG00000171160 | -1.7 | 3.257e-21 | 1.992e-20 | MORN4 |
| ENSG00000171161 | -2.488 | 1.153e-53 | 2.282e-52 | ZNF672 |
| ENSG00000171189 | 2.369 | 4.19e-05 | 9.597e-05 | GRIK1 |
| ENSG00000171208 | -1.137 | 2.467e-21 | 1.517e-20 | NETO2 |
| ENSG00000171219 | -2.161 | 1.867e-48 | 3.207e-47 | CDC42BPG |
| ENSG00000171222 | -1.084 | 2.01e-05 | 4.729e-05 | SCAND1 |
| ENSG00000171236 | -2.644 | 3.32e-25 | 2.429e-24 | LRG1 |
| ENSG00000171241 | -1.728 | 7.339e-23 | 4.804e-22 | SHCBP1 |
| ENSG00000171246 | -2.801 | 3.288e-109 | 2.555e-107 | NPTX1 |
| ENSG00000171282 | -2.489 | 1.176e-85 | 5.592e-84 | RP11-1055B8.7 |
| ENSG00000171303 | 2.36 | 0.00025 | 0.0005298 | KCNK3 |
| ENSG00000171320 | -1.726 | 2.17e-20 | 1.276e-19 | ESCO2 |
| ENSG00000171357 | 3.491 | 0.004413 | 0.008045 | LURAP1 |
| ENSG00000171396 | 3.282 | 0.01513 | 0.02549 | KRTAP4-4 |
| ENSG00000171403 | 1.229 | 9.64e-06 | 2.335e-05 | KRT9 |
| ENSG00000171421 | -1.091 | 4.101e-08 | 1.205e-07 | MRPL36 |
| ENSG00000171443 | -1.097 | 4.264e-08 | 1.251e-07 | ZNF524 |
| ENSG00000171444 | -1.177 | 8.004e-09 | 2.48e-08 | MCC |
| ENSG00000171450 | 1.689 | 0.0008934 | 0.001781 | CDK5R2 |
| ENSG00000171451 | 1.607 | 5.172e-14 | 2.216e-13 | DSEL |
| ENSG00000171462 | -1.701 | 1.68e-05 | 3.979e-05 | DLK2 |
| ENSG00000171466 | 1.566 | 4.408e-46 | 7.088e-45 | ZNF562 |
| ENSG00000171467 | -2.387 | 4.493e-49 | 7.897e-48 | ZNF318 |
| ENSG00000171471 | 2.069 | 1.081e-06 | 2.847e-06 | MAP1LC3B2 |
| ENSG00000171487 | 3.94 | 0.0008084 | 0.001619 | NLRP5 |
| ENSG00000171492 | -1.342 | 6.556e-52 | 1.244e-50 | LRRC8D |
| ENSG00000171497 | 1.231 | 2.043e-39 | 2.65e-38 | PPID |
| ENSG00000171502 | 2.652 | 0.0009834 | 0.001953 | COL24A1 |
| ENSG00000171533 | 8.149 | 1.887e-17 | 9.612e-17 | MAP6 |
| ENSG00000171566 | 1.371 | 2.291e-28 | 1.915e-27 | PLRG1 |
| ENSG00000171570 | 4.76 | 3.106e-05 | 7.186e-05 | RAB4B-EGLN2 |
| ENSG00000171574 | -1.552 | 3.434e-30 | 3.086e-29 | ZNF584 |
| ENSG00000171603 | -1.014 | 1.765e-21 | 1.095e-20 | CLSTN1 |
| ENSG00000171604 | -3.555 | 9.75e-119 | 9.352e-117 | CXXC5 |
| ENSG00000171606 | 1.368 | 1.643e-54 | 3.331e-53 | ZNF274 |
| ENSG00000171612 | -1.089 | 1.023e-08 | 3.138e-08 | SLC25A33 |
| ENSG00000171621 | 1.787 | 9.77e-64 | 2.658e-62 | SPSB1 |
| ENSG00000171631 | -2.498 | 9.689e-17 | 4.756e-16 | P2RY6 |
| ENSG00000171649 | 4.312 | 0.0001635 | 0.0003528 | ZIK1 |
| ENSG00000171700 | -2.041 | 1.277e-20 | 7.573e-20 | RGS19 |
| ENSG00000171722 | 1.56 | 0.004672 | 0.008485 | C1orf111 |
| ENSG00000171729 | -1.539 | 9.526e-14 | 4.034e-13 | TMEM51 |
| ENSG00000171757 | -1.605 | 5.001e-11 | 1.799e-10 | LRRC34 |
| ENSG00000171786 | 5.13 | 2.314e-28 | 1.934e-27 | NHLH1 |
| ENSG00000171790 | -2.814 | 2.776e-11 | 1.015e-10 | SLFNL1 |
| ENSG00000171792 | -1.82 | 1.274e-22 | 8.265e-22 | RHNO1 |
| ENSG00000171806 | -1.931 | 2.555e-18 | 1.364e-17 | METTL18 |
| ENSG00000171813 | -3.046 | 1.862e-55 | 3.873e-54 | PWWP2B |
| ENSG00000171823 | 1.508 | 8.05e-13 | 3.229e-12 | FBXL14 |
| ENSG00000171827 | -2.637 | 0.02347 | 0.0382 | ZNF570 |
| ENSG00000171848 | -1.094 | 1.203e-30 | 1.102e-29 | RRM2 |
| ENSG00000171867 | 2.341 | 7.948e-208 | 3.254e-205 | PRNP |
| ENSG00000171903 | 2.731 | 1.425e-210 | 6.134e-208 | CYP4F11 |
| ENSG00000171931 | 1.495 | 1.301e-28 | 1.103e-27 | FBXW10 |
| ENSG00000171940 | -1.194 | 2.048e-29 | 1.795e-28 | ZNF217 |
| ENSG00000171954 | 2.999 | 0.01673 | 0.02797 | CYP4F22 |
| ENSG00000171988 | 2.605 | 1.413e-64 | 3.961e-63 | JMJD1C |
| ENSG00000171989 | 2.389 | 0.003371 | 0.006245 | LDHAL6B |
| ENSG00000172000 | 3.826 | 0.001005 | 0.001994 | ZNF556 |
| ENSG00000172006 | -1.104 | 0.01548 | 0.02603 | ZNF554 |
| ENSG00000172007 | -1.315 | 1.541e-08 | 4.66e-08 | RAB33B |
| ENSG00000172009 | -1.323 | 1.049e-36 | 1.231e-35 | THOP1 |
| ENSG00000172031 | -1.136 | 0.0001205 | 0.0002639 | EPHX4 |
| ENSG00000172037 | -1.095 | 7.867e-39 | 1.007e-37 | LAMB2 |
| ENSG00000172057 | -1.005 | 1.567e-07 | 4.422e-07 | ORMDL3 |
| ENSG00000172059 | -1.923 | 2.244e-30 | 2.032e-29 | KLF11 |
| ENSG00000172061 | 2.987 | 1.631e-05 | 3.869e-05 | LRRC15 |
| ENSG00000172062 | 1.101 | 2.228e-06 | 5.69e-06 | SMN1 |
| ENSG00000172071 | 1.753 | 2.592e-55 | 5.371e-54 | EIF2AK3 |
| ENSG00000172081 | -1.282 | 6.158e-16 | 2.909e-15 | MOB3A |
| ENSG00000172086 | -1.147 | 5.051e-07 | 1.367e-06 | KRCC1 |
| ENSG00000172159 | 1.246 | 7.392e-06 | 1.809e-05 | FRMD3 |
| ENSG00000172164 | -1.436 | 0.0008802 | 0.001756 | SNTB1 |
| ENSG00000172167 | -1.678 | 1.148e-35 | 1.303e-34 | MTBP |
| ENSG00000172171 | -1.389 | 2.588e-15 | 1.183e-14 | TEFM |
| ENSG00000172183 | 2.825 | 1.631e-18 | 8.79e-18 | ISG20 |
| ENSG00000172197 | -2.842 | 6.241e-24 | 4.317e-23 | MBOAT1 |
| ENSG00000172209 | 2.89 | 0.0253 | 0.04097 | GPR22 |
| ENSG00000172232 | 4.33 | 7.119e-06 | 1.744e-05 | AZU1 |
| ENSG00000172244 | -1.303 | 2.779e-18 | 1.48e-17 | C5orf34 |
| ENSG00000172296 | -2.275 | 5.007e-10 | 1.685e-09 | SPTLC3 |
| ENSG00000172301 | -1.288 | 9.325e-08 | 2.672e-07 | COPRS |
| ENSG00000172315 | -2.486 | 7.394e-46 | 1.182e-44 | TP53RK |
| ENSG00000172336 | -1.934 | 2.116e-21 | 1.307e-20 | POP7 |
| ENSG00000172339 | -1.699 | 4.975e-17 | 2.488e-16 | ALG14 |
| ENSG00000172345 | -1.616 | 0.0004107 | 0.0008506 | STARD5 |
| ENSG00000172346 | 3.567 | 0.0002317 | 0.0004929 | CSDC2 |
| ENSG00000172349 | 4.049 | 1.222e-10 | 4.289e-10 | IL16 |
| ENSG00000172361 | 1.919 | 9.259e-11 | 3.269e-10 | CCDC11 |
| ENSG00000172366 | -1.807 | 3.26e-13 | 1.337e-12 | FAM195A |
| ENSG00000172382 | -1.905 | 1.325e-17 | 6.795e-17 | PRSS27 |
| ENSG00000172426 | 1.959 | 1.085e-05 | 2.616e-05 | RSPH9 |
| ENSG00000172458 | -2.677 | 2.87e-09 | 9.172e-09 | IL17D |
| ENSG00000172461 | -3.858 | 1.225e-108 | 9.347e-107 | FUT9 |
| ENSG00000172469 | -3.235 | 9.893e-37 | 1.162e-35 | MANEA |
| ENSG00000172476 | -1.818 | 1.563e-06 | 4.053e-06 | RAB40A |
| ENSG00000172478 | -2.362 | 2.596e-29 | 2.267e-28 | C2orf54 |
| ENSG00000172493 | 1.098 | 4.671e-18 | 2.46e-17 | AFF1 |
| ENSG00000172530 | 1.485 | 3.118e-32 | 3.074e-31 | BANP |
| ENSG00000172543 | 3.344 | 3.707e-08 | 1.093e-07 | CTSW |
| ENSG00000172586 | -1.396 | 7.075e-05 | 0.0001586 | CHCHD1 |
| ENSG00000172594 | -1.589 | 0.0004183 | 0.0008657 | SMPDL3A |
| ENSG00000172602 | 2.737 | 5.159e-26 | 3.933e-25 | RND1 |
| ENSG00000172638 | 3.27 | 1.086e-23 | 7.423e-23 | EFEMP2 |
| ENSG00000172667 | -1.106 | 9.486e-15 | 4.222e-14 | ZMAT3 |
| ENSG00000172671 | -1.775 | 2.298e-16 | 1.11e-15 | ZFAND4 |
| ENSG00000172673 | 3.52 | 0.003517 | 0.0065 | THEMIS |
| ENSG00000172687 | -1.611 | 0.004456 | 0.008112 | ZNF738 |
| ENSG00000172716 | -1.641 | 0.00414 | 0.007581 | SLFN11 |
| ENSG00000172728 | -2.396 | 5.42e-08 | 1.579e-07 | FUT10 |
| ENSG00000172731 | -3.49 | 2.682e-63 | 7.214e-62 | LRRC20 |
| ENSG00000172738 | 3.187 | 9.756e-20 | 5.561e-19 | TMEM217 |
| ENSG00000172789 | -3.047 | 0.0005443 | 0.001112 | HOXC5 |
| ENSG00000172803 | 2.705 | 1.625e-22 | 1.048e-21 | SNX32 |
| ENSG00000172818 | 2.174 | 8.84e-41 | 1.201e-39 | OVOL1 |
| ENSG00000172819 | -1.343 | 1.41e-28 | 1.192e-27 | RARG |
| ENSG00000172828 | -1.826 | 4.215e-09 | 1.331e-08 | CES3 |
| ENSG00000172830 | -1.623 | 2.924e-55 | 6.036e-54 | SSH3 |
| ENSG00000172845 | -1.504 | 1.054e-55 | 2.208e-54 | SP3 |
| ENSG00000172878 | -1.742 | 4.518e-34 | 4.797e-33 | METAP1D |
| ENSG00000172927 | -2.756 | 6.572e-120 | 6.489e-118 | MYEOV |
| ENSG00000172935 | 1.801 | 0.007097 | 0.01258 | MRGPRF |
| ENSG00000172936 | -1.469 | 8.101e-23 | 5.298e-22 | MYD88 |
| ENSG00000172938 | 4.684 | 4.748e-06 | 1.179e-05 | MRGPRD |
| ENSG00000172939 | 1.433 | 3.401e-76 | 1.283e-74 | OXSR1 |
| ENSG00000172940 | 2.876 | 0.0007933 | 0.00159 | SLC22A13 |
| ENSG00000172943 | 1.027 | 3.009e-14 | 1.305e-13 | PHF8 |
| ENSG00000172954 | -1.127 | 3.375e-12 | 1.3e-11 | LCLAT1 |
| ENSG00000172969 | 3.049 | 0.02328 | 0.03793 | FRG2C |
| ENSG00000173013 | -1.838 | 1.664e-05 | 3.943e-05 | CCDC96 |
| ENSG00000173077 | 2.522 | 0.0003562 | 0.0007431 | 1-Dec |
| ENSG00000173080 | 1.952 | 0.01116 | 0.01919 | RXFP4 |
| ENSG00000173088 | -2.885 | 0.004908 | 0.008894 | C10orf131 |
| ENSG00000173110 | 12.87 | 1.713e-112 | 1.416e-110 | HSPA6 |
| ENSG00000173120 | 1.133 | 1.393e-45 | 2.208e-44 | KDM2A |
| ENSG00000173141 | -1.522 | 5.383e-21 | 3.258e-20 | MRP63 |
| ENSG00000173153 | -1.29 | 1.708e-08 | 5.155e-08 | ESRRA |
| ENSG00000173157 | -1.787 | 0.01735 | 0.02893 | ADAMTS20 |
| ENSG00000173166 | 1.087 | 8.223e-06 | 2.002e-05 | RAPH1 |
| ENSG00000173209 | 1.83 | 4.86e-75 | 1.777e-73 | AHSA2 |
| ENSG00000173212 | 1.595 | 0.008384 | 0.0147 | MAB21L3 |
| ENSG00000173218 | -4.616 | 0 | 0 | VANGL1 |
| ENSG00000173221 | 1.999 | 1.113e-32 | 1.117e-31 | GLRX |
| ENSG00000173227 | -1.374 | 8.152e-08 | 2.346e-07 | SYT12 |
| ENSG00000173258 | 1.684 | 6.121e-07 | 1.644e-06 | ZNF483 |
| ENSG00000173269 | 1.148 | 0.01522 | 0.02562 | MMRN2 |
| ENSG00000173275 | -1.852 | 3.362e-25 | 2.458e-24 | ZNF449 |
| ENSG00000173276 | 1.156 | 6.226e-16 | 2.939e-15 | ZBTB21 |
| ENSG00000173327 | -1.234 | 3.887e-18 | 2.054e-17 | MAP3K11 |
| ENSG00000173334 | 3.006 | 1.398e-127 | 1.585e-125 | TRIB1 |
| ENSG00000173402 | -2.88 | 1.223e-290 | 8.209e-288 | DAG1 |
| ENSG00000173456 | -2.411 | 6.053e-85 | 2.862e-83 | RNF26 |
| ENSG00000173545 | 1.249 | 2.04e-37 | 2.476e-36 | ZNF622 |
| ENSG00000173546 | -1.222 | 0.0002838 | 0.0005984 | CSPG4 |
| ENSG00000173548 | -2.894 | 8.704e-103 | 6.012e-101 | SNX33 |
| ENSG00000173566 | -2.596 | 3.858e-11 | 1.4e-10 | NUDT18 |
| ENSG00000173581 | 1.561 | 3.899e-08 | 1.148e-07 | CCDC106 |
| ENSG00000173588 | -1.493 | 1.021e-15 | 4.767e-15 | CCDC41 |
| ENSG00000173611 | -1.417 | 2.42e-25 | 1.786e-24 | SCAI |
| ENSG00000173621 | -2.12 | 2.016e-21 | 1.247e-20 | LRFN4 |
| ENSG00000173638 | -3.036 | 1.269e-38 | 1.611e-37 | SLC19A1 |
| ENSG00000173662 | 4.461 | 1.98e-05 | 4.664e-05 | TAS1R1 |
| ENSG00000173692 | 1.244 | 2.96e-59 | 7.017e-58 | PSMD1 |
| ENSG00000173705 | -1.79 | 6.214e-26 | 4.718e-25 | SUSD5 |
| ENSG00000173786 | -1.981 | 5.549e-45 | 8.608e-44 | CNP |
| ENSG00000173812 | 1.026 | 2.917e-09 | 9.318e-09 | EIF1 |
| ENSG00000173818 | 1.014 | 2.897e-11 | 1.058e-10 | ENDOV |
| ENSG00000173846 | 1.525 | 2.691e-21 | 1.65e-20 | PLK3 |
| ENSG00000173848 | -1.511 | 4.186e-62 | 1.093e-60 | NET1 |
| ENSG00000173852 | -1.483 | 1.194e-25 | 8.959e-25 | DPY19L1 |
| ENSG00000173875 | 1.25 | 3.736e-18 | 1.975e-17 | ZNF791 |
| ENSG00000173898 | -2.149 | 4.622e-73 | 1.603e-71 | SPTBN2 |
| ENSG00000173905 | -1.549 | 4.851e-17 | 2.427e-16 | GOLIM4 |
| ENSG00000173914 | -1.091 | 8.826e-12 | 3.32e-11 | RBM4B |
| ENSG00000173918 | -1.133 | 0.000119 | 0.0002607 | C1QTNF1 |
| ENSG00000173926 | -1.704 | 0.0004696 | 0.0009662 | 3-Mar |
| ENSG00000173928 | -1.271 | 4.227e-05 | 9.678e-05 | SWSAP1 |
| ENSG00000173950 | -2.242 | 1.505e-33 | 1.56e-32 | XXYLT1 |
| ENSG00000173960 | -1.26 | 8.712e-21 | 5.208e-20 | UBXN2A |
| ENSG00000173991 | 2.287 | 2.333e-08 | 6.978e-08 | TCAP |
| ENSG00000174004 | -2.559 | 0.003387 | 0.006271 | NRROS |
| ENSG00000174007 | -1.68 | 1.34e-10 | 4.684e-10 | CEP19 |
| ENSG00000174032 | -2.708 | 2.991e-43 | 4.424e-42 | SLC25A30 |
| ENSG00000174038 | 4.546 | 1.893e-10 | 6.552e-10 | C9orf131 |
| ENSG00000174123 | 4.392 | 9.694e-05 | 0.0002146 | TLR10 |
| ENSG00000174145 | 3.351 | 2.74e-06 | 6.942e-06 | KIAA1239 |
| ENSG00000174151 | -1.558 | 4.2e-20 | 2.434e-19 | CYB561D1 |
| ENSG00000174165 | -1.403 | 1.536e-15 | 7.09e-15 | ZDHHC24 |
| ENSG00000174177 | -1.179 | 1.231e-09 | 4.049e-09 | CTU2 |
| ENSG00000174194 | 1.259 | 5.254e-09 | 1.649e-08 | AGAP8 |
| ENSG00000174225 | 1.464 | 0.001078 | 0.002133 | ARL13A |
| ENSG00000174233 | -1.726 | 1.66e-20 | 9.799e-20 | ADCY6 |
| ENSG00000174243 | -1.028 | 8.196e-22 | 5.143e-21 | DDX23 |
| ENSG00000174276 | -2.663 | 1.505e-29 | 1.326e-28 | ZNHIT2 |
| ENSG00000174282 | -1.179 | 8.35e-28 | 6.834e-27 | ZBTB4 |
| ENSG00000174292 | -1.019 | 1.258e-10 | 4.406e-10 | TNK1 |
| ENSG00000174306 | -2.785 | 3.612e-116 | 3.208e-114 | ZHX3 |
| ENSG00000174307 | -2.191 | 0.0002186 | 0.0004665 | PHLDA3 |
| ENSG00000174326 | 2.458 | 4.852e-05 | 0.0001105 | SLC16A11 |
| ENSG00000174327 | -1.138 | 1.981e-12 | 7.753e-12 | SLC16A13 |
| ENSG00000174343 | 1.692 | 0.008618 | 0.01508 | CHRNA9 |
| ENSG00000174358 | 5.293 | 6.627e-08 | 1.917e-07 | SLC6A19 |
| ENSG00000174371 | -2.691 | 2.788e-88 | 1.414e-86 | EXO1 |
| ENSG00000174374 | -1.289 | 1.372e-21 | 8.537e-21 | WBSCR16 |
| ENSG00000174417 | 2.215 | 0.03137 | 0.04993 | TRHR |
| ENSG00000174446 | -1.498 | 1.063e-13 | 4.485e-13 | SNAPC5 |
| ENSG00000174456 | -1.89 | 7.852e-08 | 2.262e-07 | C12orf76 |
| ENSG00000174460 | 2.89 | 0.0002215 | 0.0004722 | ZCCHC12 |
| ENSG00000174501 | 1.228 | 4.085e-08 | 1.2e-07 | ANKRD36C |
| ENSG00000174502 | 1.811 | 0.003077 | 0.005739 | SLC26A9 |
| ENSG00000174514 | -1.766 | 2.457e-05 | 5.739e-05 | MFSD4 |
| ENSG00000174516 | -1.16 | 3.685e-08 | 1.087e-07 | PELI3 |
| ENSG00000174521 | 2.528 | 4.731e-05 | 0.0001078 | TTC9B |
| ENSG00000174600 | 3.791 | 0.000872 | 0.00174 | CMKLR1 |
| ENSG00000174607 | -2.357 | 0.0003403 | 0.0007115 | UGT8 |
| ENSG00000174669 | -2.3 | 6.6e-66 | 1.913e-64 | SLC29A2 |
| ENSG00000174672 | -2.639 | 5.374e-19 | 2.957e-18 | BRSK2 |
| ENSG00000174684 | -4.719 | 6.044e-57 | 1.318e-55 | B3GNT1 |
| ENSG00000174697 | 3.451 | 0.004361 | 0.007959 | LEP |
| ENSG00000174721 | -3.259 | 2.441e-05 | 5.705e-05 | FGFBP3 |
| ENSG00000174788 | 2.037 | 0.02438 | 0.03956 | PCP2 |
| ENSG00000174791 | -2.449 | 1.062e-29 | 9.401e-29 | RIN1 |
| ENSG00000174792 | 2.32 | 0.00197 | 0.00378 | C4orf26 |
| ENSG00000174804 | -2.668 | 5.517e-45 | 8.566e-44 | FZD4 |
| ENSG00000174839 | -1.058 | 6.397e-19 | 3.506e-18 | DENND6A |
| ENSG00000174840 | -1.465 | 2.286e-43 | 3.4e-42 | PDE12 |
| ENSG00000174842 | -1.405 | 5.925e-22 | 3.738e-21 | GLMN |
| ENSG00000174871 | 2.833 | 4.502e-16 | 2.143e-15 | CNIH2 |
| ENSG00000174898 | 3.929 | 0.0004307 | 0.0008903 | CATSPERD |
| ENSG00000174928 | -1.014 | 9.336e-06 | 2.264e-05 | C3orf33 |
| ENSG00000174945 | 1.202 | 0.008067 | 0.01419 | AMZ1 |
| ENSG00000174989 | -1.458 | 1.198e-29 | 1.059e-28 | FBXW8 |
| ENSG00000175003 | 1.831 | 0.005837 | 0.01047 | SLC22A1 |
| ENSG00000175048 | -1.152 | 9.876e-08 | 2.825e-07 | ZDHHC14 |
| ENSG00000175054 | -2.256 | 3.408e-120 | 3.426e-118 | ATR |
| ENSG00000175063 | -1.164 | 7.362e-30 | 6.549e-29 | UBE2C |
| ENSG00000175066 | -1.364 | 8.504e-17 | 4.195e-16 | GK5 |
| ENSG00000175087 | -1.262 | 2.564e-12 | 9.964e-12 | PDIK1L |
| ENSG00000175105 | 1.952 | 2.613e-32 | 2.589e-31 | ZNF654 |
| ENSG00000175106 | 1.001 | 0.002061 | 0.003945 | TVP23C |
| ENSG00000175121 | 1.915 | 0.02183 | 0.03577 | WFDC5 |
| ENSG00000175130 | -1.71 | 4.486e-81 | 1.897e-79 | MARCKSL1 |
| ENSG00000175182 | -2.316 | 1.036e-25 | 7.792e-25 | FAM131A |
| ENSG00000175183 | 2.708 | 4.484e-21 | 2.724e-20 | CSRP2 |
| ENSG00000175193 | -1.098 | 2.431e-12 | 9.458e-12 | PARL |
| ENSG00000175197 | 4.216 | 8.99299999999999e-195 | 2.795e-192 | DDIT3 |
| ENSG00000175216 | -2.067 | 2.024e-73 | 7.094e-72 | CKAP5 |
| ENSG00000175220 | -1.275 | 2.094e-36 | 2.427e-35 | ARHGAP1 |
| ENSG00000175221 | -1.332 | 1.175e-24 | 8.391e-24 | MED16 |
| ENSG00000175229 | 3.945 | 0.0008264 | 0.001654 | GAL3ST3 |
| ENSG00000175283 | -3.165 | 4.571e-44 | 6.925e-43 | DOLK |
| ENSG00000175305 | -3.267 | 3.472e-72 | 1.178e-70 | CCNE2 |
| ENSG00000175318 | -3.368 | 1.703e-74 | 6.163e-73 | GRAMD2 |
| ENSG00000175344 | 2.878 | 0.0235 | 0.03825 | CHRNA7 |
| ENSG00000175352 | 1.451 | 0.0003293 | 0.0006898 | NRIP3 |
| ENSG00000175356 | 2.94 | 0.0008458 | 0.00169 | SCUBE2 |
| ENSG00000175376 | 1.898 | 1.952e-38 | 2.458e-37 | EIF1AD |
| ENSG00000175414 | 2.615 | 7.354e-16 | 3.457e-15 | ARL10 |
| ENSG00000175445 | -1.648 | 0.001473 | 0.002869 | LPL |
| ENSG00000175455 | -1.129 | 5.265e-23 | 3.467e-22 | CCDC14 |
| ENSG00000175463 | 1.703 | 0.003765 | 0.006933 | TBC1D10C |
| ENSG00000175505 | 1.826 | 3.145e-60 | 7.752e-59 | CLCF1 |
| ENSG00000175514 | 3.236 | 0.008717 | 0.01524 | GPR152 |
| ENSG00000175536 | -2.547 | 0.0001939 | 0.0004154 | LIPT2 |
| ENSG00000175548 | -1.389 | 2.286e-15 | 1.047e-14 | ALG10B |
| ENSG00000175556 | 1.71 | 3.477e-54 | 6.997e-53 | LONRF3 |
| ENSG00000175573 | 1.069 | 1.594e-16 | 7.738e-16 | C11orf68 |
| ENSG00000175591 | -5.436 | 5.232e-92 | 2.908e-90 | P2RY2 |
| ENSG00000175592 | 2.986 | 1.522e-12 | 6.004e-12 | FOSL1 |
| ENSG00000175595 | -2.22 | 1.359e-46 | 2.227e-45 | ERCC4 |
| ENSG00000175602 | 1.329 | 4.406e-19 | 2.435e-18 | CCDC85B |
| ENSG00000175643 | -2.903 | 1.387e-09 | 4.54e-09 | RMI2 |
| ENSG00000175707 | -2.834 | 1.386e-18 | 7.49e-18 | C1orf172 |
| ENSG00000175711 | -1.268 | 3.916e-10 | 1.327e-09 | B3GNTL1 |
| ENSG00000175745 | -1.561 | 1.17e-06 | 3.072e-06 | NR2F1 |
| ENSG00000175764 | -2.001 | 8.907e-10 | 2.952e-09 | TTLL11 |
| ENSG00000175782 | -1.185 | 7.4e-11 | 2.631e-10 | SLC35E3 |
| ENSG00000175792 | -1.056 | 3.818e-18 | 2.018e-17 | RUVBL1 |
| ENSG00000175806 | -1.035 | 1.724e-05 | 4.08e-05 | MSRA |
| ENSG00000175866 | -1.068 | 1.802e-28 | 1.514e-27 | BAIAP2 |
| ENSG00000175874 | -1.912 | 8.499e-09 | 2.626e-08 | CREG2 |
| ENSG00000175879 | -2.796 | 0.01376 | 0.02333 | HOXD8 |
| ENSG00000175894 | 3.624 | 0.002424 | 0.00459 | TSPEAR |
| ENSG00000175906 | -1.639 | 0.0001401 | 0.0003046 | ARL4D |
| ENSG00000175920 | -1.626 | 1.358e-07 | 3.852e-07 | DOK7 |
| ENSG00000175970 | -1.883 | 1.73e-31 | 1.653e-30 | UNC119B |
| ENSG00000175984 | 4.911 | 0 | 0 | DENND2C |
| ENSG00000175985 | 1.824 | 0.002579 | 0.004868 | PLEKHD1 |
| ENSG00000176014 | -1.024 | 1.337e-15 | 6.204e-15 | TUBB6 |
| ENSG00000176018 | 2.167 | 1.675e-69 | 5.303e-68 | LYSMD3 |
| ENSG00000176022 | -1.754 | 4.452e-23 | 2.94e-22 | B3GALT6 |
| ENSG00000176024 | -1.721 | 1.287e-09 | 4.225e-09 | ZNF613 |
| ENSG00000176046 | 1.095 | 0.02718 | 0.04378 | NUPR1 |
| ENSG00000176055 | -4.67 | 1.675e-76 | 6.347e-75 | MBLAC2 |
| ENSG00000176058 | -2.942 | 2.187e-60 | 5.416e-59 | TPRN |
| ENSG00000176087 | -1.106 | 3.482e-15 | 1.585e-14 | SLC35A4 |
| ENSG00000176101 | -1.216 | 1.454e-08 | 4.406e-08 | SSNA1 |
| ENSG00000176108 | -2.866 | 8.123e-33 | 8.184e-32 | CHMP6 |
| ENSG00000176124 | -2.27 | 1.86e-18 | 9.991e-18 | DLEU1 |
| ENSG00000176148 | -1.523 | 1.132e-30 | 1.039e-29 | TCP11L1 |
| ENSG00000176165 | -1.698 | 0.002865 | 0.005364 | FOXG1 |
| ENSG00000176170 | 1.037 | 1.215e-05 | 2.914e-05 | SPHK1 |
| ENSG00000176182 | -1.364 | 2.389e-07 | 6.642e-07 | MYPOP |
| ENSG00000176208 | -2.218 | 3.961e-54 | 7.934e-53 | ATAD5 |
| ENSG00000176222 | -1.216 | 0.02641 | 0.04263 | ZNF404 |
| ENSG00000176225 | -1.023 | 2.364e-10 | 8.141e-10 | RTTN |
| ENSG00000176273 | -1.551 | 1.088e-23 | 7.432e-23 | SLC35G1 |
| ENSG00000176274 | 2.51 | 2.032e-24 | 1.436e-23 | SLC25A53 |
| ENSG00000176349 | 2.487 | 0.002087 | 0.003992 | AC110781.3 |
| ENSG00000176383 | 1.481 | 3.588e-05 | 8.264e-05 | B3GNT4 |
| ENSG00000176387 | -2.152 | 3.591e-10 | 1.221e-09 | HSD11B2 |
| ENSG00000176396 | -1.765 | 6.232e-14 | 2.66e-13 | EID2 |
| ENSG00000176399 | -1.537 | 0.003098 | 0.005777 | DMRTA1 |
| ENSG00000176401 | -1.628 | 0.006464 | 0.01153 | EID2B |
| ENSG00000176402 | 2.673 | 0.01629 | 0.02728 | GJC3 |
| ENSG00000176407 | 1.065 | 1.14e-11 | 4.268e-11 | KCMF1 |
| ENSG00000176410 | -1.165 | 1.135e-07 | 3.235e-07 | DNAJC30 |
| ENSG00000176422 | -1.553 | 1.187e-11 | 4.44e-11 | SPRYD4 |
| ENSG00000176444 | -1.321 | 1.026e-24 | 7.335e-24 | CLK2 |
| ENSG00000176454 | -1.91 | 2.872e-13 | 1.183e-12 | LPCAT4 |
| ENSG00000176472 | -1.768 | 0.000209 | 0.0004466 | ZNF575 |
| ENSG00000176473 | -1.211 | 9.289e-05 | 0.000206 | WDR25 |
| ENSG00000176531 | 1.088 | 7.998e-14 | 3.395e-13 | PHLDB3 |
| ENSG00000176532 | -4.061 | 5.295e-94 | 3.075e-92 | PRR15 |
| ENSG00000176542 | 1.386 | 7.309e-16 | 3.439e-15 | KIAA2018 |
| ENSG00000176563 | 2.364 | 3.37e-15 | 1.534e-14 | CNTD1 |
| ENSG00000176595 | -1.51 | 1.195e-05 | 2.869e-05 | KBTBD11 |
| ENSG00000176601 | 3.537 | 0.006849 | 0.01218 | MAP3K19 |
| ENSG00000176619 | -1.658 | 9.647e-46 | 1.536e-44 | LMNB2 |
| ENSG00000176697 | -1.228 | 1.722e-05 | 4.075e-05 | BDNF |
| ENSG00000176714 | 2.082 | 8.553e-31 | 7.901e-30 | CCDC121 |
| ENSG00000176715 | -1.671 | 2.745e-29 | 2.392e-28 | ACSF3 |
| ENSG00000176720 | -2.839 | 2.674e-58 | 6.099e-57 | BOK |
| ENSG00000176753 | -3.131 | 0.00518 | 0.009361 | C15orf56 |
| ENSG00000176771 | 3.529 | 0.005416 | 0.009767 | NCKAP5 |
| ENSG00000176834 | -1.867 | 6.038e-38 | 7.491e-37 | VSIG10 |
| ENSG00000176853 | 1.198 | 5.448e-49 | 9.555e-48 | FAM91A1 |
| ENSG00000176884 | -2.267 | 5.244e-32 | 5.124e-31 | GRIN1 |
| ENSG00000176890 | -1.165 | 7.032e-17 | 3.483e-16 | TYMS |
| ENSG00000176903 | -1.355 | 1.008e-19 | 5.742e-19 | PNMA1 |
| ENSG00000176907 | -2.841 | 4.324e-36 | 4.972e-35 | C8orf4 |
| ENSG00000176909 | -3.535 | 0.001179 | 0.002321 | MAMSTR |
| ENSG00000176915 | 1.03 | 3.619e-20 | 2.103e-19 | ANKLE2 |
| ENSG00000176920 | -1.571 | 0.0007417 | 0.001491 | FUT2 |
| ENSG00000176928 | -1.041 | 0.0005771 | 0.001177 | GCNT4 |
| ENSG00000176945 | -1.118 | 6.777e-10 | 2.261e-09 | MUC20 |
| ENSG00000176946 | -1.059 | 1.033e-18 | 5.61e-18 | THAP4 |
| ENSG00000176974 | -1.793 | 5.879e-22 | 3.71e-21 | SHMT1 |
| ENSG00000176988 | 2.6 | 0.0006969 | 0.001405 | FMR1NB |
| ENSG00000176994 | 1.114 | 5.24e-11 | 1.884e-10 | SMCR8 |
| ENSG00000177045 | -1.759 | 3.161e-08 | 9.371e-08 | SIX5 |
| ENSG00000177084 | -1.092 | 4.541e-42 | 6.471e-41 | POLE |
| ENSG00000177106 | -1.037 | 4.627e-16 | 2.2e-15 | EPS8L2 |
| ENSG00000177169 | 1.211 | 5.676e-28 | 4.673e-27 | ULK1 |
| ENSG00000177181 | -2.592 | 3.497e-19 | 1.944e-18 | RIMKLA |
| ENSG00000177200 | -2.086 | 1.754e-41 | 2.453e-40 | CHD9 |
| ENSG00000177225 | -2.417 | 1.791e-48 | 3.087e-47 | PDDC1 |
| ENSG00000177239 | -1.556 | 2.132e-14 | 9.31e-14 | MAN1B1 |
| ENSG00000177303 | -2.944 | 2.665e-38 | 3.338e-37 | CASKIN2 |
| ENSG00000177335 | -1.807 | 1.413e-19 | 8.002e-19 | C8orf31 |
| ENSG00000177352 | -2.391 | 4.588e-15 | 2.076e-14 | CCDC71 |
| ENSG00000177370 | -2.444 | 2.05e-47 | 3.441e-46 | TIMM22 |
| ENSG00000177380 | -1.375 | 2.099e-08 | 6.298e-08 | PPFIA3 |
| ENSG00000177383 | -1.533 | 1.286e-10 | 4.503e-10 | MAGEF1 |
| ENSG00000177409 | -1.243 | 2.103e-08 | 6.311e-08 | SAMD9L |
| ENSG00000177427 | -1.923 | 3.994e-08 | 1.175e-07 | MIEF2 |
| ENSG00000177453 | 1.538 | 0.006595 | 0.01175 | NIM1 |
| ENSG00000177459 | -1.208 | 1.315e-05 | 3.147e-05 | C8orf47 |
| ENSG00000177464 | 3.453 | 0.003452 | 0.006387 | GPR4 |
| ENSG00000177465 | -3.228 | 3.975e-29 | 3.448e-28 | ACOT4 |
| ENSG00000177483 | 2.061 | 2.084e-05 | 4.898e-05 | RBM44 |
| ENSG00000177485 | -1.32 | 1.234e-27 | 1.001e-26 | ZBTB33 |
| ENSG00000177494 | -4.954 | 6.431e-48 | 1.091e-46 | ZBED2 |
| ENSG00000177508 | -3.566 | 8.249e-92 | 4.555e-90 | IRX3 |
| ENSG00000177542 | -2.066 | 6.59e-32 | 6.398e-31 | SLC25A22 |
| ENSG00000177548 | -1.172 | 3.278e-11 | 1.193e-10 | RABEP2 |
| ENSG00000177565 | -1.047 | 2.827e-28 | 2.35e-27 | TBL1XR1 |
| ENSG00000177570 | -1.878 | 3.63e-50 | 6.567e-49 | SAMD12 |
| ENSG00000177575 | 5.889 | 1.122e-18 | 6.087e-18 | CD163 |
| ENSG00000177595 | -1.258 | 9.288e-10 | 3.078e-09 | PIDD |
| ENSG00000177602 | -1.191 | 2.408e-10 | 8.283e-10 | GSG2 |
| ENSG00000177606 | 1.967 | 1.429e-104 | 1.021e-102 | JUN |
| ENSG00000177614 | 4.92 | 1.097e-06 | 2.888e-06 | PGBD5 |
| ENSG00000177669 | 2.091 | 0.00282 | 0.005287 | MBOAT4 |
| ENSG00000177688 | 4.082 | 3.317e-08 | 9.819e-08 | SUMO4 |
| ENSG00000177692 | -1.489 | 0.009742 | 0.01691 | DNAJC28 |
| ENSG00000177700 | -1.025 | 0.0002207 | 0.0004706 | POLR2L |
| ENSG00000177706 | -2.684 | 1.135e-46 | 1.869e-45 | FAM20C |
| ENSG00000177732 | -3.327 | 2.72e-98 | 1.697e-96 | SOX12 |
| ENSG00000177764 | -1.683 | 1.796e-34 | 1.953e-33 | ZCCHC3 |
| ENSG00000177791 | -1.307 | 0.007333 | 0.01297 | MYOZ1 |
| ENSG00000177830 | -1.021 | 9.18e-06 | 2.228e-05 | CHID1 |
| ENSG00000177842 | -3.55 | 9.032e-28 | 7.371e-27 | ZNF620 |
| ENSG00000177854 | -2.624 | 1.92e-11 | 7.098e-11 | TMEM187 |
| ENSG00000177873 | -4.214 | 8.24e-34 | 8.656e-33 | ZNF619 |
| ENSG00000177932 | -2.045 | 0.01708 | 0.02852 | ZNF354C |
| ENSG00000177943 | 1.485 | 7.378e-05 | 0.000165 | MAMDC4 |
| ENSG00000177946 | -1.634 | 6.805e-20 | 3.906e-19 | CENPBD1 |
| ENSG00000177963 | -1.05 | 3.968e-15 | 1.8e-14 | RIC8A |
| ENSG00000177971 | -3.451 | 1.989e-60 | 4.931e-59 | IMP3 |
| ENSG00000178021 | 3.546 | 1.59e-06 | 4.12e-06 | TSPYL6 |
| ENSG00000178026 | -1.451 | 0.009949 | 0.01725 | FAM211B |
| ENSG00000178028 | 1.234 | 2.734e-29 | 2.384e-28 | DMAP1 |
| ENSG00000178033 | 2.609 | 0.007184 | 0.01273 | FAM26E |
| ENSG00000178053 | 1.194 | 4.21e-22 | 2.675e-21 | MLF1 |
| ENSG00000178074 | -2.096 | 3.009e-35 | 3.368e-34 | C2orf69 |
| ENSG00000178075 | -2.705 | 2.42e-35 | 2.719e-34 | GRAMD1C |
| ENSG00000178096 | -3.241 | 7.252e-13 | 2.912e-12 | BOLA1 |
| ENSG00000178150 | 1.791 | 2.81e-40 | 3.749e-39 | ZNF114 |
| ENSG00000178163 | -1.054 | 3.363e-18 | 1.783e-17 | ZNF518B |
| ENSG00000178184 | 1.114 | 1.085e-10 | 3.82e-10 | PARD6G |
| ENSG00000178188 | -1.75 | 2.204e-27 | 1.769e-26 | SH2B1 |
| ENSG00000178202 | -2.359 | 6.494e-99 | 4.098e-97 | KDELC2 |
| ENSG00000178234 | -1.816 | 3.039e-55 | 6.266e-54 | GALNT11 |
| ENSG00000178252 | -2.084 | 2.032e-26 | 1.568e-25 | WDR6 |
| ENSG00000178279 | 3.065 | 0.03109 | 0.04952 | TNP2 |
| ENSG00000178301 | -4.849 | 3.628e-11 | 1.317e-10 | AQP11 |
| ENSG00000178338 | -1.496 | 2.46e-13 | 1.018e-12 | ZNF354B |
| ENSG00000178342 | 3.523 | 5.766e-08 | 1.677e-07 | KCNG2 |
| ENSG00000178343 | 3.122 | 0.0228 | 0.0372 | SHISA3 |
| ENSG00000178381 | 5.512 | 0 | 0 | ZFAND2A |
| ENSG00000178397 | -2.642 | 4.606e-30 | 4.115e-29 | FAM220A |
| ENSG00000178401 | -2.486 | 1.547e-05 | 3.676e-05 | DNAJC22 |
| ENSG00000178409 | -2.689 | 2.748e-63 | 7.381e-62 | BEND3 |
| ENSG00000178445 | -1.455 | 0.0001299 | 0.0002834 | GLDC |
| ENSG00000178460 | -1.991 | 0.0001054 | 0.0002327 | MCMDC2 |
| ENSG00000178531 | -2.061 | 3.178e-17 | 1.601e-16 | CTXN1 |
| ENSG00000178597 | -1.858 | 0.009744 | 0.01691 | PSAPL1 |
| ENSG00000178607 | 2.706 | 1.826e-150 | 2.975e-148 | ERN1 |
| ENSG00000178685 | -3.219 | 1.819e-27 | 1.463e-26 | PARP10 |
| ENSG00000178695 | -1.633 | 2.101e-06 | 5.375e-06 | KCTD12 |
| ENSG00000178700 | -3.324 | 1.046e-37 | 1.286e-36 | DHFRL1 |
| ENSG00000178718 | -4.873 | 4.185e-95 | 2.482e-93 | RPP25 |
| ENSG00000178722 | 5.418 | 6e-08 | 1.741e-07 | C5orf64 |
| ENSG00000178726 | -2.895 | 3.276e-32 | 3.224e-31 | THBD |
| ENSG00000178761 | -1.515 | 1.366e-11 | 5.086e-11 | FAM219B |
| ENSG00000178773 | -1.638 | 7.299e-33 | 7.385e-32 | CPNE7 |
| ENSG00000178789 | 3.288 | 0.008229 | 0.01445 | CD300LB |
| ENSG00000178802 | -1.383 | 6.616e-11 | 2.362e-10 | MPI |
| ENSG00000178814 | -1.158 | 1.517e-05 | 3.609e-05 | OPLAH |
| ENSG00000178826 | -2.559 | 1.142e-79 | 4.632e-78 | TMEM139 |
| ENSG00000178913 | 1.442 | 6.905e-35 | 7.606e-34 | TAF7 |
| ENSG00000178917 | -2.132 | 3.569e-07 | 9.792e-07 | ZNF852 |
| ENSG00000178919 | -4.386 | 2.14e-31 | 2.036e-30 | FOXE1 |
| ENSG00000178921 | -1.562 | 1.035e-19 | 5.891e-19 | PFAS |
| ENSG00000178927 | -1.886 | 8.684e-54 | 1.731e-52 | C17orf62 |
| ENSG00000178935 | -1.714 | 2.122e-34 | 2.301e-33 | ZNF552 |
| ENSG00000178966 | -2.121 | 8.212e-45 | 1.271e-43 | RMI1 |
| ENSG00000178971 | -2.648 | 8.95e-56 | 1.885e-54 | CTC1 |
| ENSG00000178974 | -1.012 | 4.954e-15 | 2.234e-14 | FBXO34 |
| ENSG00000178996 | -3.417 | 2.71e-69 | 8.504e-68 | SNX18 |
| ENSG00000178999 | -3.215 | 1.528e-106 | 1.12e-104 | AURKB |
| ENSG00000179021 | 1.043 | 8.483e-16 | 3.975e-15 | C3orf38 |
| ENSG00000179023 | -3.159 | 0.007023 | 0.01247 | KLHDC7A |
| ENSG00000179029 | -2.039 | 1.556e-23 | 1.053e-22 | TMEM107 |
| ENSG00000179038 | 2.881 | 0.02439 | 0.03957 | AP001885.1 |
| ENSG00000179041 | -2.624 | 4.165e-75 | 1.526e-73 | RRS1 |
| ENSG00000179046 | 2.185 | 1.146e-42 | 1.66e-41 | TRIML2 |
| ENSG00000179111 | -3.151 | 1.697e-13 | 7.094e-13 | HES7 |
| ENSG00000179119 | 1.424 | 2.236e-64 | 6.235e-63 | SPTY2D1 |
| ENSG00000179148 | 2.669 | 1.564e-46 | 2.554e-45 | ALOXE3 |
| ENSG00000179163 | -1.06 | 7.075e-12 | 2.677e-11 | FUCA1 |
| ENSG00000179168 | 4.51 | 4.387e-16 | 2.09e-15 | GGN |
| ENSG00000179178 | -2.042 | 2.778e-25 | 2.043e-24 | TMEM125 |
| ENSG00000179240 | -4.045 | 7.338e-27 | 5.77e-26 | RP11-111M22.2 |
| ENSG00000179241 | -1.169 | 1.682e-27 | 1.355e-26 | LDLRAD3 |
| ENSG00000179270 | 3.09 | 6.137e-05 | 0.0001385 | C2orf71 |
| ENSG00000179292 | 3.913 | 0.001982 | 0.003802 | TMEM151A |
| ENSG00000179294 | -2.426 | 4.548e-21 | 2.761e-20 | C17orf96 |
| ENSG00000179299 | -2.748 | 2.43e-13 | 1.006e-12 | NSUN7 |
| ENSG00000179314 | -2.422 | 1.841e-22 | 1.187e-21 | WSCD1 |
| ENSG00000179331 | 3.29 | 0.01266 | 0.0216 | RAB39A |
| ENSG00000179335 | 1.261 | 2.488e-28 | 2.076e-27 | CLK3 |
| ENSG00000179348 | -1.85 | 3.542e-51 | 6.555e-50 | GATA2 |
| ENSG00000179361 | 1.034 | 5.487e-11 | 1.969e-10 | ARID3B |
| ENSG00000179363 | 3.465 | 0.004375 | 0.007983 | TMEM31 |
| ENSG00000179364 | -1.356 | 6.664e-22 | 4.196e-21 | PACS2 |
| ENSG00000179387 | -1.332 | 7.59e-28 | 6.221e-27 | ELMOD2 |
| ENSG00000179388 | 6.022 | 2.162e-73 | 7.562e-72 | EGR3 |
| ENSG00000179403 | -2.219 | 3.519e-31 | 3.305e-30 | VWA1 |
| ENSG00000179409 | -1.857 | 2.902e-80 | 1.2e-78 | GEMIN4 |
| ENSG00000179431 | -4.321 | 1.225e-72 | 4.214e-71 | FJX1 |
| ENSG00000179454 | 1.088 | 2.37e-20 | 1.389e-19 | KLHL28 |
| ENSG00000179477 | 2.216 | 0.005571 | 0.01003 | ALOX12B |
| ENSG00000179520 | 4.201 | 1.595e-07 | 4.499e-07 | SLC17A8 |
| ENSG00000179546 | -1.349 | 1.379e-08 | 4.185e-08 | HTR1D |
| ENSG00000179562 | 1.274 | 9.151e-13 | 3.657e-12 | GCC1 |
| ENSG00000179564 | 4.179 | 8.152e-09 | 2.525e-08 | LSMEM2 |
| ENSG00000179580 | 2.564 | 0.01982 | 0.03271 | RNF151 |
| ENSG00000179598 | -1.777 | 1.337e-18 | 7.231e-18 | PLD6 |
| ENSG00000179604 | -2.294 | 4.578e-36 | 5.26e-35 | CDC42EP4 |
| ENSG00000179627 | -3.269 | 1.5e-17 | 7.675e-17 | ZBTB42 |
| ENSG00000179632 | -1.046 | 8.443e-30 | 7.491e-29 | MAF1 |
| ENSG00000179673 | 3.314 | 0.01437 | 0.0243 | RPRML |
| ENSG00000179674 | 1.989 | 3.77e-35 | 4.197e-34 | ARL14 |
| ENSG00000179715 | -2.054 | 5.314e-11 | 1.91e-10 | PCED1B |
| ENSG00000179761 | 2.592 | 0.004174 | 0.007637 | PIPOX |
| ENSG00000179820 | 1.754 | 1.662e-18 | 8.948e-18 | MYADM |
| ENSG00000179837 | -1.052 | 2.146e-19 | 1.2e-18 | RBM15B |
| ENSG00000179841 | -1.019 | 0.001633 | 0.003165 | AKAP5 |
| ENSG00000179862 | -2.74 | 9.425e-11 | 3.327e-10 | CITED4 |
| ENSG00000179869 | 2.393 | 1.654e-05 | 3.919e-05 | ABCA13 |
| ENSG00000179886 | -2.673 | 1.821e-14 | 7.982e-14 | TIGD5 |
| ENSG00000179902 | 3.171 | 0.01088 | 0.01874 | C1orf194 |
| ENSG00000179913 | -2.687 | 4.046e-97 | 2.497e-95 | B3GNT3 |
| ENSG00000179933 | -2.399 | 1.964e-67 | 5.952e-66 | C14orf119 |
| ENSG00000179954 | 1.797 | 4.74e-08 | 1.386e-07 | SSC5D |
| ENSG00000179958 | -2.277 | 4.839e-28 | 3.993e-27 | DCTPP1 |
| ENSG00000179979 | -1.67 | 6.139e-08 | 1.78e-07 | CRIPAK |
| ENSG00000179981 | -2.325 | 2.262e-22 | 1.452e-21 | TSHZ1 |
| ENSG00000180011 | -4.114 | 2.261e-108 | 1.717e-106 | ZADH2 |
| ENSG00000180035 | -3.437 | 1.066e-52 | 2.06e-51 | ZNF48 |
| ENSG00000180043 | 4.947 | 9.744e-07 | 2.579e-06 | FAM71E2 |
| ENSG00000180061 | 3.05 | 0.01676 | 0.02801 | TMEM150B |
| ENSG00000180071 | -1.624 | 5.457e-10 | 1.832e-09 | ANKRD18A |
| ENSG00000180089 | -2.434 | 1.188e-28 | 1.009e-27 | TMEM86B |
| ENSG00000180096 | 1.27 | 0.01082 | 0.01865 | SEPT1 |
| ENSG00000180185 | -1.604 | 1.853e-24 | 1.313e-23 | FAHD1 |
| ENSG00000180245 | 1.955 | 0.0004452 | 0.000918 | RRH |
| ENSG00000180257 | -1.172 | 0.008093 | 0.01423 | ZNF816 |
| ENSG00000180304 | -1.294 | 4.44e-09 | 1.4e-08 | OAZ2 |
| ENSG00000180316 | 3.017 | 9.182e-09 | 2.827e-08 | PNPLA1 |
| ENSG00000180332 | 3.072 | 0.01318 | 0.02241 | KCTD4 |
| ENSG00000180336 | 2.739 | 5.913e-18 | 3.099e-17 | C17orf104 |
| ENSG00000180340 | -4.45 | 1.642e-65 | 4.729e-64 | FZD2 |
| ENSG00000180346 | -3.703 | 5.753e-59 | 1.338e-57 | TIGD2 |
| ENSG00000180353 | 2.286 | 0.0002165 | 0.0004623 | HCLS1 |
| ENSG00000180376 | -1.808 | 1.947e-19 | 1.094e-18 | CCDC66 |
| ENSG00000180423 | -2.253 | 4.372e-16 | 2.084e-15 | HARBI1 |
| ENSG00000180432 | 1.229 | 0.0002996 | 0.0006301 | CYP8B1 |
| ENSG00000180438 | 3.155 | 0.02353 | 0.03829 | TPRXL |
| ENSG00000180447 | -2.648 | 2.947e-06 | 7.454e-06 | GAS1 |
| ENSG00000180448 | -1.724 | 6.793e-37 | 8.064e-36 | HMHA1 |
| ENSG00000180479 | -1.548 | 0.003552 | 0.006562 | ZNF571 |
| ENSG00000180488 | -1.008 | 1.433e-16 | 6.984e-16 | FAM73A |
| ENSG00000180530 | -2.252 | 4.551e-102 | 3.131e-100 | NRIP1 |
| ENSG00000180535 | 2.772 | 7.212e-05 | 0.0001615 | BHLHA15 |
| ENSG00000180537 | -1.79 | 0.002117 | 0.004043 | RNF182 |
| ENSG00000180543 | -2.664 | 9.3e-53 | 1.799e-51 | TSPYL5 |
| ENSG00000180573 | -1.149 | 1.129e-06 | 2.969e-06 | HIST1H2AC |
| ENSG00000180592 | -3.509 | 1.036e-08 | 3.176e-08 | SKIDA1 |
| ENSG00000180596 | -1.004 | 0.0136 | 0.02308 | HIST1H2BC |
| ENSG00000180611 | -1.779 | 2.81e-19 | 1.567e-18 | MB21D2 |
| ENSG00000180626 | -1.527 | 6.335e-17 | 3.146e-16 | ZNF594 |
| ENSG00000180638 | -2.01 | 2.568e-05 | 5.986e-05 | SLC47A2 |
| ENSG00000180694 | -1.979 | 1.631e-46 | 2.661e-45 | TMEM64 |
| ENSG00000180730 | 4.739 | 4.141e-06 | 1.036e-05 | SHISA2 |
| ENSG00000180739 | -5.666 | 2.936e-10 | 1.005e-09 | S1PR5 |
| ENSG00000180758 | -2.255 | 7.089e-28 | 5.816e-27 | GPR157 |
| ENSG00000180777 | 5.05 | 3.644e-10 | 1.238e-09 | ANKRD30B |
| ENSG00000180806 | -1.906 | 1.438e-09 | 4.7e-09 | HOXC9 |
| ENSG00000180815 | 3.505 | 0.008629 | 0.0151 | MAP3K15 |
| ENSG00000180818 | -1.443 | 0.0002554 | 0.0005404 | HOXC10 |
| ENSG00000180822 | -1.497 | 1.204e-21 | 7.511e-21 | PSMG4 |
| ENSG00000180834 | -3.522 | 4.03e-22 | 2.563e-21 | MAP6D1 |
| ENSG00000180855 | -1.743 | 0.01285 | 0.02188 | ZNF443 |
| ENSG00000180871 | 4.893 | 2.561e-06 | 6.509e-06 | CXCR2 |
| ENSG00000180875 | 2.513 | 0.007598 | 0.01342 | GREM2 |
| ENSG00000180881 | 1.197 | 0.0006989 | 0.001409 | CAPS2 |
| ENSG00000180884 | -3.474 | 4.716e-10 | 1.59e-09 | ZNF792 |
| ENSG00000180891 | -1.15 | 7.473e-11 | 2.656e-10 | CUEDC1 |
| ENSG00000180901 | -1.348 | 6.986e-24 | 4.818e-23 | KCTD2 |
| ENSG00000180902 | -2.335 | 1.733e-64 | 4.841e-63 | D2HGDH |
| ENSG00000180921 | -1.674 | 2.415e-63 | 6.508e-62 | FAM83H |
| ENSG00000180938 | -2.361 | 0.000364 | 0.0007586 | ZNF572 |
| ENSG00000180953 | -1.476 | 1.874e-05 | 4.42e-05 | ST20 |
| ENSG00000180957 | 1.487 | 3.023e-57 | 6.677e-56 | PITPNB |
| ENSG00000180964 | -1.146 | 1.167e-13 | 4.916e-13 | TCEAL8 |
| ENSG00000180998 | -1.697 | 2.665e-08 | 7.941e-08 | GPR137C |
| ENSG00000181016 | 2.597 | 6.956e-13 | 2.797e-12 | LSMEM1 |
| ENSG00000181027 | -1.198 | 1.11e-14 | 4.92e-14 | FKRP |
| ENSG00000181031 | -1.023 | 3.472e-08 | 1.026e-07 | RPH3AL |
| ENSG00000181035 | -1.371 | 0.003457 | 0.006395 | SLC25A42 |
| ENSG00000181038 | -1.377 | 8.603e-17 | 4.242e-16 | METTL23 |
| ENSG00000181090 | -1.403 | 4.948e-34 | 5.251e-33 | EHMT1 |
| ENSG00000181104 | -1.951 | 7.638e-06 | 1.866e-05 | F2R |
| ENSG00000181191 | -1.195 | 6.459e-25 | 4.657e-24 | PJA1 |
| ENSG00000181192 | -1.236 | 7.558e-23 | 4.944e-22 | DHTKD1 |
| ENSG00000181220 | 1.096 | 3.948e-36 | 4.548e-35 | ZNF746 |
| ENSG00000181222 | -2.408 | 8.145e-25 | 5.841e-24 | POLR2A |
| ENSG00000181264 | -2.971 | 6.617e-10 | 2.209e-09 | TMEM136 |
| ENSG00000181274 | -2.528 | 8.048e-11 | 2.852e-10 | FRAT2 |
| ENSG00000181284 | -1.332 | 1.671e-09 | 5.431e-09 | TMEM102 |
| ENSG00000181322 | -1.839 | 0.008635 | 0.01511 | NME9 |
| ENSG00000181350 | -1.246 | 4.719e-05 | 0.0001076 | FAM211A |
| ENSG00000181355 | -3.12 | 0.004527 | 0.008234 | OFCC1 |
| ENSG00000181378 | 2.207 | 0.0006651 | 0.001346 | CCDC108 |
| ENSG00000181392 | -1.205 | 2.314e-10 | 7.976e-10 | SYNE4 |
| ENSG00000181396 | -1.567 | 2.484e-21 | 1.527e-20 | OGFOD3 |
| ENSG00000181444 | -1.063 | 0.007619 | 0.01345 | ZNF467 |
| ENSG00000181450 | 1.695 | 8.764e-37 | 1.035e-35 | ZNF678 |
| ENSG00000181464 | 1.118 | 0.01047 | 0.0181 | CDRT1 |
| ENSG00000181481 | -2.498 | 3.996e-25 | 2.91e-24 | RNF135 |
| ENSG00000181513 | -1.811 | 6.873e-12 | 2.602e-11 | ACBD4 |
| ENSG00000181523 | -2.488 | 4.377e-49 | 7.701e-48 | SGSH |
| ENSG00000181544 | -2.358 | 4.148e-43 | 6.096e-42 | FANCB |
| ENSG00000181610 | -1.66 | 4.763e-45 | 7.402e-44 | MRPS23 |
| ENSG00000181619 | -2.135 | 1.955e-18 | 1.048e-17 | GPR135 |
| ENSG00000181634 | 1.062 | 2.924e-10 | 1.001e-09 | TNFSF15 |
| ENSG00000181638 | -2.033 | 4.09e-38 | 5.105e-37 | ZFP41 |
| ENSG00000181649 | 1.974 | 1.051e-56 | 2.275e-55 | PHLDA2 |
| ENSG00000181652 | 2.228 | 6.567e-12 | 2.489e-11 | ATG9B |
| ENSG00000181690 | -1.377 | 8.197e-07 | 2.18e-06 | PLAG1 |
| ENSG00000181722 | 2.789 | 4.369e-15 | 1.978e-14 | ZBTB20 |
| ENSG00000181751 | -2.532 | 2.348e-58 | 5.37e-57 | C5orf30 |
| ENSG00000181754 | -4.456 | 2.111e-59 | 5.056e-58 | AMIGO1 |
| ENSG00000181817 | -3.192 | 2.96e-41 | 4.09e-40 | LSM10 |
| ENSG00000181830 | -1.729 | 1.331e-13 | 5.595e-13 | SLC35C1 |
| ENSG00000181856 | -2.946 | 0.01518 | 0.02556 | SLC2A4 |
| ENSG00000181873 | -1.789 | 9.801e-31 | 9.025e-30 | IBA57 |
| ENSG00000181894 | 1.161 | 7.375e-10 | 2.456e-09 | ZNF329 |
| ENSG00000181904 | -1.78 | 3.815e-35 | 4.244e-34 | C5orf24 |
| ENSG00000181915 | -2.267 | 2.871e-25 | 2.109e-24 | ADO |
| ENSG00000181924 | -1.369 | 1.105e-23 | 7.546e-23 | COA4 |
| ENSG00000181938 | -2.352 | 1.166e-28 | 9.908e-28 | GINS3 |
| ENSG00000182010 | -2.112 | 4.06e-82 | 1.757e-80 | RTKN2 |
| ENSG00000182013 | 5.415 | 1.483e-08 | 4.489e-08 | PNMAL1 |
| ENSG00000182093 | -1.111 | 2.758e-11 | 1.009e-10 | WRB |
| ENSG00000182095 | -1.035 | 1.959e-31 | 1.867e-30 | TNRC18 |
| ENSG00000182107 | -2.138 | 1.221e-79 | 4.94e-78 | TMEM30B |
| ENSG00000182108 | -2.341 | 1.557e-15 | 7.188e-15 | DEXI |
| ENSG00000182141 | -1.372 | 0.0179 | 0.02978 | ZNF708 |
| ENSG00000182154 | -1.372 | 1.973e-13 | 8.212e-13 | MRPL41 |
| ENSG00000182156 | 2.561 | 0.01181 | 0.02023 | ENPP7 |
| ENSG00000182173 | -2.733 | 1.253e-50 | 2.291e-49 | TSEN54 |
| ENSG00000182175 | -2.833 | 5.848e-11 | 2.095e-10 | RGMA |
| ENSG00000182180 | -1.844 | 2.554e-21 | 1.568e-20 | MRPS16 |
| ENSG00000182197 | 1.114 | 4.052e-32 | 3.977e-31 | EXT1 |
| ENSG00000182230 | 3.879 | 0.0004749 | 0.0009764 | FAM153B |
| ENSG00000182253 | -3.307 | 2.085e-54 | 4.212e-53 | SYNM |
| ENSG00000182257 | -3.398 | 0.00151 | 0.002938 | C22orf26 |
| ENSG00000182261 | 1.487 | 4.973e-06 | 1.233e-05 | NLRP10 |
| ENSG00000182263 | -1.789 | 5.688e-17 | 2.831e-16 | FIGN |
| ENSG00000182264 | 1.78 | 0.008944 | 0.01561 | IZUMO1 |
| ENSG00000182272 | -1.817 | 6.144e-33 | 6.239e-32 | B4GALNT4 |
| ENSG00000182319 | -3.611 | 1.017e-39 | 1.336e-38 | SGK223 |
| ENSG00000182325 | -1.295 | 7.454e-18 | 3.88e-17 | FBXL6 |
| ENSG00000182327 | -3.012 | 0.007246 | 0.01283 | GLTPD2 |
| ENSG00000182359 | -1.925 | 0.0001785 | 0.000384 | KBTBD3 |
| ENSG00000182372 | 1.289 | 4.869e-15 | 2.197e-14 | CLN8 |
| ENSG00000182389 | 1.913 | 1.53e-07 | 4.32e-07 | CACNB4 |
| ENSG00000182450 | 2.046 | 0.01393 | 0.0236 | KCNK4 |
| ENSG00000182459 | 3.58 | 1.274e-11 | 4.753e-11 | TEX19 |
| ENSG00000182489 | 1.612 | 2.163e-13 | 8.983e-13 | XKRX |
| ENSG00000182500 | -2.385 | 3.863e-36 | 4.456e-35 | ORAI1 |
| ENSG00000182511 | 3.447 | 0.000254 | 0.0005378 | FES |
| ENSG00000182512 | -1.004 | 1.121e-15 | 5.229e-15 | GLRX5 |
| ENSG00000182534 | 1.486 | 4.786e-06 | 1.188e-05 | MXRA7 |
| ENSG00000182541 | -1.418 | 1.315e-17 | 6.749e-17 | LIMK2 |
| ENSG00000182544 | -2.25 | 1.894e-35 | 2.135e-34 | MFSD5 |
| ENSG00000182551 | -1.003 | 1.251e-11 | 4.673e-11 | ADI1 |
| ENSG00000182580 | -1.092 | 1.189e-11 | 4.445e-11 | EPHB3 |
| ENSG00000182613 | 3.144 | 0.02664 | 0.04298 | OR2V2 |
| ENSG00000182676 | 3.304 | 0.006761 | 0.01203 | PPP1R27 |
| ENSG00000182685 | -1.157 | 0.03035 | 0.04842 | BRICD5 |
| ENSG00000182687 | 3.426 | 0.0005375 | 0.001099 | GALR2 |
| ENSG00000182700 | -3.384 | 5.043e-31 | 4.695e-30 | IGIP |
| ENSG00000182704 | -3.932 | 4.843e-188 | 1.378e-185 | TSKU |
| ENSG00000182742 | -3.561 | 1.285e-05 | 3.076e-05 | HOXB4 |
| ENSG00000182749 | -3.265 | 3.959e-72 | 1.337e-70 | PAQR7 |
| ENSG00000182759 | 5.155 | 2.736e-13 | 1.13e-12 | MAFA |
| ENSG00000182791 | -2.248 | 0.03015 | 0.04814 | CCDC87 |
| ENSG00000182810 | -2.64 | 5.244e-33 | 5.342e-32 | DDX28 |
| ENSG00000182836 | -3.458 | 1.632e-30 | 1.487e-29 | PLCXD3 |
| ENSG00000182851 | 5.288 | 4.562e-07 | 1.239e-06 | GPIHBP1 |
| ENSG00000182870 | 4.051 | 8.331e-116 | 7.284e-114 | GALNT9 |
| ENSG00000182885 | 1.585 | 0.02141 | 0.0351 | GPR97 |
| ENSG00000182896 | 3.077 | 0.01485 | 0.02504 | TMEM95 |
| ENSG00000182901 | -2.273 | 1.76e-23 | 1.189e-22 | RGS7 |
| ENSG00000182903 | 1.197 | 5.913e-10 | 1.98e-09 | ZNF721 |
| ENSG00000182909 | -2.976 | 2.969e-25 | 2.18e-24 | LENG9 |
| ENSG00000182912 | 2.676 | 0.0005285 | 0.001082 | C21orf90 |
| ENSG00000182923 | -1.167 | 1.558e-12 | 6.139e-12 | CEP63 |
| ENSG00000182944 | 1.316 | 3.891e-56 | 8.278e-55 | EWSR1 |
| ENSG00000182952 | -1.313 | 1.623e-10 | 5.637e-10 | HMGN4 |
| ENSG00000182983 | 1.211 | 0.000209 | 0.0004465 | ZNF662 |
| ENSG00000182986 | -1.744 | 1.136e-09 | 3.743e-09 | ZNF320 |
| ENSG00000183018 | -2.416 | 1.016e-55 | 2.132e-54 | SPNS2 |
| ENSG00000183023 | -3.155 | 1.79e-14 | 7.849e-14 | SLC8A1 |
| ENSG00000183048 | -1.74 | 3.392e-42 | 4.846e-41 | SLC25A10 |
| ENSG00000183060 | -1.022 | 0.0002541 | 0.000538 | LYSMD4 |
| ENSG00000183072 | -1.352 | 1.705e-06 | 4.405e-06 | NKX2-5 |
| ENSG00000183077 | -1.027 | 3.79e-06 | 9.499e-06 | AFMID |
| ENSG00000183087 | -1.658 | 1.745e-18 | 9.386e-18 | GAS6 |
| ENSG00000183111 | -1.037 | 1.369e-06 | 3.571e-06 | ARHGEF37 |
| ENSG00000183117 | -2.377 | 0.001643 | 0.003183 | CSMD1 |
| ENSG00000183137 | -2.188 | 8.386e-20 | 4.791e-19 | CEP57L1 |
| ENSG00000183150 | -2.328 | 3.51e-10 | 1.195e-09 | GPR19 |
| ENSG00000183154 | 3.448 | 0.003382 | 0.006264 | RP11-863K10.7 |
| ENSG00000183160 | 2.632 | 0.03123 | 0.04973 | TMEM119 |
| ENSG00000183161 | -2.173 | 3.686e-25 | 2.687e-24 | FANCF |
| ENSG00000183186 | -2.6 | 3.529e-06 | 8.869e-06 | C2CD4C |
| ENSG00000183196 | -3.274 | 0.004631 | 0.008414 | CHST6 |
| ENSG00000183246 | -3.485 | 2.209e-05 | 5.18e-05 | RIMBP3C |
| ENSG00000183250 | -2.331 | 6.046e-07 | 1.625e-06 | C21orf67 |
| ENSG00000183281 | 2.347 | 5.09e-08 | 1.485e-07 | PLGLB1 |
| ENSG00000183287 | -1.874 | 8.997e-24 | 6.177e-23 | CCBE1 |
| ENSG00000183309 | -1.393 | 3.295e-34 | 3.537e-33 | ZNF623 |
| ENSG00000183317 | -2.112 | 3.622e-08 | 1.069e-07 | EPHA10 |
| ENSG00000183323 | -1.867 | 4.143e-32 | 4.065e-31 | CCDC125 |
| ENSG00000183340 | -1.974 | 7.42e-22 | 4.661e-21 | JRKL |
| ENSG00000183354 | 1.394 | 1.712e-07 | 4.811e-07 | KIAA2026 |
| ENSG00000183395 | -2.627 | 0.02646 | 0.0427 | PMCH |
| ENSG00000183423 | 2.308 | 0.0007503 | 0.001508 | LRIT3 |
| ENSG00000183426 | -1.006 | 1.616e-08 | 4.883e-08 | NPIPA1 |
| ENSG00000183474 | -1.263 | 1.15e-27 | 9.355e-27 | GTF2H2C |
| ENSG00000183484 | 2.311 | 0.01841 | 0.03056 | GPR132 |
| ENSG00000183508 | 2.035 | 1.309e-37 | 1.602e-36 | FAM46C |
| ENSG00000183513 | -1.08 | 3.276e-12 | 1.264e-11 | COA5 |
| ENSG00000183520 | 1.262 | 1.171e-31 | 1.128e-30 | UTP11L |
| ENSG00000183527 | -1.091 | 2.103e-19 | 1.178e-18 | PSMG1 |
| ENSG00000183558 | 1.546 | 2.656e-06 | 6.737e-06 | HIST2H2AA3 |
| ENSG00000183562 | 1.671 | 0.0003259 | 0.000683 | AC131971.1 |
| ENSG00000183570 | 1.961 | 0.0004403 | 0.0009089 | PCBP3 |
| ENSG00000183605 | -1.105 | 5.689e-13 | 2.3e-12 | SFXN4 |
| ENSG00000183624 | -1.975 | 1.868e-26 | 1.443e-25 | HMCES |
| ENSG00000183665 | -1.021 | 5.115e-09 | 1.607e-08 | TRMT12 |
| ENSG00000183671 | -1.744 | 2.247e-06 | 5.736e-06 | GPR1 |
| ENSG00000183675 | -1.682 | 3.427e-14 | 1.48e-13 | PTPN20B |
| ENSG00000183688 | 1.89 | 1.892e-07 | 5.295e-07 | FAM101B |
| ENSG00000183691 | -5.334 | 3.527e-37 | 4.244e-36 | NOG |
| ENSG00000183696 | 2.149 | 4.212e-76 | 1.585e-74 | UPP1 |
| ENSG00000183718 | -2.048 | 6.117e-12 | 2.325e-11 | TRIM52 |
| ENSG00000183722 | -1.586 | 0.001426 | 0.002783 | LHFP |
| ENSG00000183723 | -1.905 | 4.274e-37 | 5.12e-36 | CMTM4 |
| ENSG00000183741 | -2.005 | 2.167e-48 | 3.715e-47 | CBX6 |
| ENSG00000183748 | 3.956 | 8.778e-12 | 3.303e-11 | MRC1L1 |
| ENSG00000183760 | 1.08 | 2.791e-09 | 8.93e-09 | PAPL |
| ENSG00000183765 | -1.331 | 1.144e-15 | 5.335e-15 | CHEK2 |
| ENSG00000183773 | -2.816 | 5.894e-08 | 1.713e-07 | AIFM3 |
| ENSG00000183779 | -1.87 | 4.696e-17 | 2.352e-16 | ZNF703 |
| ENSG00000183780 | -1.317 | 1.219e-10 | 4.277e-10 | SLC35F3 |
| ENSG00000183785 | 1.802 | 0.0001132 | 0.0002489 | TUBA8 |
| ENSG00000183840 | -2.094 | 1.537e-17 | 7.863e-17 | GPR39 |
| ENSG00000183853 | -1.191 | 4.786e-29 | 4.132e-28 | KIRREL |
| ENSG00000183856 | -1.174 | 6.237e-28 | 5.127e-27 | IQGAP3 |
| ENSG00000183864 | -1.665 | 5.766e-61 | 1.451e-59 | TOB2 |
| ENSG00000183876 | -4.658 | 4.208e-23 | 2.786e-22 | ARSI |
| ENSG00000183891 | -1.701 | 1.164e-15 | 5.425e-15 | TTC32 |
| ENSG00000183914 | -1.075 | 2.873e-14 | 1.247e-13 | DNAH2 |
| ENSG00000183921 | 3.214 | 0.01779 | 0.02961 | SDR42E2 |
| ENSG00000183943 | -1.09 | 4.259e-15 | 1.93e-14 | PRKX |
| ENSG00000183963 | 1.292 | 3.166e-25 | 2.319e-24 | SMTN |
| ENSG00000183977 | 1.295 | 0.007438 | 0.01315 | PP2D1 |
| ENSG00000183978 | -1.851 | 4.817e-19 | 2.655e-18 | COA3 |
| ENSG00000183979 | 2.93 | 1.229e-05 | 2.946e-05 | NPB |
| ENSG00000184005 | 2.404 | 0.003073 | 0.005733 | ST6GALNAC3 |
| ENSG00000184012 | -1.803 | 0.00642 | 0.01146 | TMPRSS2 |
| ENSG00000184040 | 2.92 | 1.804e-34 | 1.96e-33 | TMEM236 |
| ENSG00000184056 | -2.88 | 3.022e-33 | 3.1e-32 | VPS33B |
| ENSG00000184058 | 1.805 | 0.01735 | 0.02893 | TBX1 |
| ENSG00000184083 | -2.481 | 5.41e-19 | 2.975e-18 | FAM120C |
| ENSG00000184108 | 3.402 | 0.004038 | 0.007403 | TRIML1 |
| ENSG00000184154 | 1.004 | 2.672e-12 | 1.037e-11 | LRTOMT |
| ENSG00000184156 | 1.979 | 1.228e-12 | 4.867e-12 | KCNQ3 |
| ENSG00000184162 | -2.42 | 1.258e-46 | 2.064e-45 | NR2C2AP |
| ENSG00000184185 | 2.338 | 1.646e-16 | 7.989e-16 | KCNJ12 |
| ENSG00000184205 | 2.23 | 4.891e-32 | 4.787e-31 | TSPYL2 |
| ENSG00000184207 | -2.749 | 4.682e-74 | 1.672e-72 | PGP |
| ENSG00000184227 | -2.411 | 4.636e-10 | 1.564e-09 | ACOT1 |
| ENSG00000184292 | -2.004 | 9.029e-82 | 3.866e-80 | TACSTD2 |
| ENSG00000184305 | -1.354 | 0.01474 | 0.02488 | CCSER1 |
| ENSG00000184307 | -2.345 | 1.274e-18 | 6.898e-18 | ZDHHC23 |
| ENSG00000184313 | 3.141 | 1.375e-06 | 3.584e-06 | MROH7 |
| ENSG00000184343 | -1.402 | 0.02674 | 0.04311 | SRPK3 |
| ENSG00000184349 | -1.178 | 1.03e-13 | 4.355e-13 | EFNA5 |
| ENSG00000184363 | -1.823 | 3.117e-33 | 3.197e-32 | PKP3 |
| ENSG00000184368 | 1.373 | 0.0272 | 0.0438 | MAP7D2 |
| ENSG00000184378 | 1.474 | 1.694e-08 | 5.115e-08 | ACTRT3 |
| ENSG00000184381 | 1.046 | 3.026e-08 | 8.976e-08 | PLA2G6 |
| ENSG00000184384 | -2.637 | 9.161e-34 | 9.599e-33 | MAML2 |
| ENSG00000184436 | -2.773 | 1.214e-51 | 2.284e-50 | THAP7 |
| ENSG00000184459 | 4.585 | 8.991e-06 | 2.184e-05 | BPIFC |
| ENSG00000184465 | -1.115 | 3.812e-19 | 2.113e-18 | WDR27 |
| ENSG00000184470 | -1.024 | 1.174e-05 | 2.822e-05 | TXNRD2 |
| ENSG00000184489 | -1.73 | 2.677e-10 | 9.183e-10 | PTP4A3 |
| ENSG00000184502 | 2.286 | 0.006418 | 0.01146 | GAST |
| ENSG00000184508 | -2.452 | 1.634e-23 | 1.105e-22 | HDDC3 |
| ENSG00000184517 | -2.034 | 6.45e-24 | 4.456e-23 | ZFP1 |
| ENSG00000184564 | -2.981 | 2.912e-14 | 1.264e-13 | SLITRK6 |
| ENSG00000184574 | -2.341 | 2.345e-21 | 1.443e-20 | LPAR5 |
| ENSG00000184584 | -1.545 | 1.35e-05 | 3.226e-05 | TMEM173 |
| ENSG00000184602 | -2.145 | 2.209e-13 | 9.168e-13 | SNN |
| ENSG00000184619 | -1.9 | 0.00416 | 0.007614 | KRBA2 |
| ENSG00000184634 | -1.069 | 1.114e-17 | 5.746e-17 | MED12 |
| ENSG00000184661 | -2.277 | 3.944e-56 | 8.38e-55 | CDCA2 |
| ENSG00000184675 | -2.215 | 4.365e-86 | 2.099e-84 | AMER1 |
| ENSG00000184677 | 1.103 | 2.288e-31 | 2.173e-30 | ZBTB40 |
| ENSG00000184702 | -1.205 | 0.001945 | 0.003737 | SEPT5 |
| ENSG00000184708 | -1.457 | 4.544e-22 | 2.879e-21 | EIF4ENIF1 |
| ENSG00000184709 | -3.889 | 5.337e-13 | 2.16e-12 | LRRC26 |
| ENSG00000184716 | 3.969 | 0.0008828 | 0.001761 | SERINC4 |
| ENSG00000184730 | -1.32 | 0.003597 | 0.006642 | APOBR |
| ENSG00000184743 | -1.859 | 4.632e-40 | 6.146e-39 | ATL3 |
| ENSG00000184785 | -2.099 | 1.28e-05 | 3.063e-05 | SMIM10 |
| ENSG00000184787 | -1.193 | 7.367e-32 | 7.144e-31 | UBE2G2 |
| ENSG00000184788 | -1.028 | 0.0008833 | 0.001761 | SATL1 |
| ENSG00000184838 | -2.401 | 0.006376 | 0.01139 | PRR16 |
| ENSG00000184860 | -2.797 | 2.185e-20 | 1.284e-19 | SDR42E1 |
| ENSG00000184886 | -2.407 | 1.226e-53 | 2.425e-52 | PIGW |
| ENSG00000184887 | -1.245 | 4.082e-10 | 1.382e-09 | BTBD6 |
| ENSG00000184897 | -4.073 | 4.435e-116 | 3.918e-114 | H1FX |
| ENSG00000184898 | -3.653 | 1.387e-25 | 1.037e-24 | RBM43 |
| ENSG00000184916 | -3.25 | 5.55e-106 | 4.051e-104 | JAG2 |
| ENSG00000184922 | -1.328 | 5.529e-12 | 2.107e-11 | FMNL1 |
| ENSG00000184937 | 4.007 | 0.0003774 | 0.0007851 | WT1 |
| ENSG00000184939 | -2.072 | 1.57e-31 | 1.504e-30 | ZFP90 |
| ENSG00000184949 | -2.453 | 5.278e-33 | 5.373e-32 | FAM227A |
| ENSG00000184979 | -1.378 | 2.584e-11 | 9.478e-11 | USP18 |
| ENSG00000184986 | -2.811 | 0.01449 | 0.02448 | TMEM121 |
| ENSG00000184988 | -1.834 | 5.492e-07 | 1.48e-06 | TMEM106A |
| ENSG00000184990 | -1.433 | 4.82e-09 | 1.517e-08 | SIVA1 |
| ENSG00000184992 | -2.001 | 1.191e-33 | 1.24e-32 | BRI3BP |
| ENSG00000185013 | -3.397 | 0.002587 | 0.004882 | NT5C1B |
| ENSG00000185019 | 1.181 | 5.786e-21 | 3.499e-20 | UBOX5 |
| ENSG00000185022 | 3.02 | 3.012e-75 | 1.109e-73 | MAFF |
| ENSG00000185024 | -2.885 | 9.568e-34 | 1.001e-32 | BRF1 |
| ENSG00000185038 | -1.945 | 0.008006 | 0.01409 | MROH2A |
| ENSG00000185085 | -2.447 | 1.73e-62 | 4.58e-61 | INTS5 |
| ENSG00000185090 | -2.59 | 5.289e-43 | 7.727e-42 | MANEAL |
| ENSG00000185100 | 1.666 | 2.075e-07 | 5.792e-07 | ADSSL1 |
| ENSG00000185101 | -1.383 | 9.144e-17 | 4.499e-16 | ANO9 |
| ENSG00000185105 | 3.723 | 0.002807 | 0.005265 | MYADML2 |
| ENSG00000185129 | -2.235 | 8.951e-52 | 1.69e-50 | PURA |
| ENSG00000185189 | -1.343 | 2.549e-20 | 1.492e-19 | NRBP2 |
| ENSG00000185215 | -2.495 | 1.07e-82 | 4.716e-81 | TNFAIP2 |
| ENSG00000185219 | -2.519 | 2.653e-51 | 4.931e-50 | ZNF445 |
| ENSG00000185220 | -2.325 | 1.209e-38 | 1.536e-37 | PGBD2 |
| ENSG00000185247 | 3.943 | 0.0008862 | 0.001767 | MAGEA11 |
| ENSG00000185252 | -2.296 | 9.278e-24 | 6.365e-23 | ZNF74 |
| ENSG00000185272 | -1.391 | 0.01477 | 0.02492 | RBM11 |
| ENSG00000185291 | 3.018 | 6.018e-10 | 2.014e-09 | IL3RA |
| ENSG00000185304 | 2.66 | 7.489e-34 | 7.896e-33 | RGPD2 |
| ENSG00000185324 | -1.239 | 8.036e-15 | 3.594e-14 | CDK10 |
| ENSG00000185332 | -2.965 | 7.02e-13 | 2.822e-12 | TMEM105 |
| ENSG00000185338 | 2.174 | 1.022e-05 | 2.468e-05 | SOCS1 |
| ENSG00000185340 | -2.903 | 3.993e-56 | 8.473e-55 | GAS2L1 |
| ENSG00000185345 | 2.732 | 0.000148 | 0.0003209 | PARK2 |
| ENSG00000185347 | -4.134 | 2.863e-30 | 2.582e-29 | C14orf80 |
| ENSG00000185361 | -2.084 | 2.741e-41 | 3.794e-40 | TNFAIP8L1 |
| ENSG00000185379 | -2.301 | 7.028e-12 | 2.66e-11 | RAD51D |
| ENSG00000185386 | -1.57 | 8.324e-11 | 2.948e-10 | MAPK11 |
| ENSG00000185436 | -1.936 | 2.938e-13 | 1.208e-12 | IFNLR1 |
| ENSG00000185442 | -2.55 | 2.104e-44 | 3.219e-43 | FAM174B |
| ENSG00000185453 | -1.523 | 5.638e-09 | 1.764e-08 | C19orf68 |
| ENSG00000185467 | 1.792 | 1.097e-14 | 4.864e-14 | KPNA7 |
| ENSG00000185479 | 2.038 | 1.824e-37 | 2.219e-36 | KRT6B |
| ENSG00000185480 | -1.263 | 1.632e-07 | 4.595e-07 | PARPBP |
| ENSG00000185482 | 1.572 | 1.059e-07 | 3.024e-07 | STAC3 |
| ENSG00000185483 | -2.364 | 1.953e-70 | 6.329e-69 | ROR1 |
| ENSG00000185504 | -1.607 | 1.028e-16 | 5.041e-16 | C17orf70 |
| ENSG00000185513 | -1.519 | 9.691e-14 | 4.102e-13 | L3MBTL1 |
| ENSG00000185515 | -1.241 | 1.496e-23 | 1.014e-22 | BRCC3 |
| ENSG00000185527 | 2.422 | 1.648e-05 | 3.907e-05 | PDE6G |
| ENSG00000185551 | -2.781 | 2.368e-70 | 7.658e-69 | NR2F2 |
| ENSG00000185565 | 4.206 | 1.018e-08 | 3.124e-08 | LSAMP |
| ENSG00000185585 | -2.231 | 1.425e-30 | 1.302e-29 | OLFML2A |
| ENSG00000185619 | 1.056 | 2.49e-25 | 1.836e-24 | PCGF3 |
| ENSG00000185621 | -1.73 | 6.85e-23 | 4.493e-22 | LMLN |
| ENSG00000185630 | -1.645 | 9.171e-25 | 6.568e-24 | PBX1 |
| ENSG00000185633 | 2.361 | 0.0002053 | 0.0004389 | NDUFA4L2 |
| ENSG00000185640 | 2.586 | 0.009606 | 0.01669 | KRT79 |
| ENSG00000185670 | 2.029 | 0.0274 | 0.04407 | ZBTB3 |
| ENSG00000185697 | -2.322 | 4.829e-29 | 4.168e-28 | MYBL1 |
| ENSG00000185722 | -1.345 | 3.142e-47 | 5.258e-46 | ANKFY1 |
| ENSG00000185728 | 1.121 | 5.148e-18 | 2.705e-17 | YTHDF3 |
| ENSG00000185730 | -2.356 | 2.754e-22 | 1.763e-21 | ZNF696 |
| ENSG00000185745 | -3.237 | 4.995e-13 | 2.027e-12 | IFIT1 |
| ENSG00000185774 | -1.091 | 0.0173 | 0.02886 | KCNIP4 |
| ENSG00000185792 | 3.133 | 7.332e-21 | 4.399e-20 | NLRP9 |
| ENSG00000185803 | -1.305 | 3.732e-12 | 1.435e-11 | SLC52A2 |
| ENSG00000185813 | -1.244 | 2.315e-15 | 1.06e-14 | PCYT2 |
| ENSG00000185818 | -3.664 | 1.072e-23 | 7.331e-23 | NAT8L |
| ENSG00000185829 | -1.38 | 1.422e-33 | 1.476e-32 | ARL17A |
| ENSG00000185875 | -3.144 | 4.093e-48 | 6.968e-47 | THNSL1 |
| ENSG00000185880 | -2.4 | 3.255e-14 | 1.409e-13 | TRIM69 |
| ENSG00000185883 | 1.021 | 1.599e-07 | 4.51e-07 | ATP6V0C |
| ENSG00000185909 | 1.773 | 0.02053 | 0.03379 | KLHDC8B |
| ENSG00000185920 | -1.933 | 3.586e-33 | 3.67e-32 | PTCH1 |
| ENSG00000185924 | -5.65 | 7.111e-16 | 3.348e-15 | RTN4RL1 |
| ENSG00000185928 | -3.221 | 6.364e-51 | 1.173e-49 | PAGR1 |
| ENSG00000185947 | 1.791 | 5.642e-33 | 5.733e-32 | ZNF267 |
| ENSG00000185950 | 1.1 | 1.295e-23 | 8.811e-23 | IRS2 |
| ENSG00000185958 | -2.309 | 0.02174 | 0.03562 | FAM186A |
| ENSG00000185989 | -2.341 | 1.041e-37 | 1.28e-36 | RASA3 |
| ENSG00000185990 | -4.209 | 4.54e-43 | 6.661e-42 | F8A3 |
| ENSG00000186001 | -1.223 | 2.707e-30 | 2.444e-29 | LRCH3 |
| ENSG00000186007 | 1.49 | 0.001772 | 0.00342 | LEMD1 |
| ENSG00000186017 | -2.082 | 1.369e-27 | 1.106e-26 | ZNF566 |
| ENSG00000186026 | 1.143 | 4.91e-06 | 1.218e-05 | ZNF284 |
| ENSG00000186073 | -1.075 | 7.4e-21 | 4.437e-20 | C15orf41 |
| ENSG00000186104 | -2.628 | 9.08e-21 | 5.422e-20 | CYP2R1 |
| ENSG00000186106 | -3.098 | 6.264e-17 | 3.112e-16 | ANKRD46 |
| ENSG00000186111 | -1.597 | 2.721e-27 | 2.177e-26 | PIP5K1C |
| ENSG00000186115 | 2.353 | 5.895e-06 | 1.454e-05 | CYP4F2 |
| ENSG00000186185 | -3.071 | 2.315e-118 | 2.195e-116 | KIF18B |
| ENSG00000186187 | -2.174 | 1.395e-56 | 3.01e-55 | ZNRF1 |
| ENSG00000186188 | -2.858 | 1.943e-05 | 4.58e-05 | FFAR4 |
| ENSG00000186193 | -2.91 | 4.339e-30 | 3.886e-29 | SAPCD2 |
| ENSG00000186204 | 1.178 | 0.01084 | 0.01868 | CYP4F12 |
| ENSG00000186205 | -2.314 | 4.894e-60 | 1.201e-58 | MARC1 |
| ENSG00000186212 | -3.688 | 5.413e-16 | 2.562e-15 | SOWAHB |
| ENSG00000186222 | -1.831 | 1.455e-14 | 6.4e-14 | BLOC1S4 |
| ENSG00000186260 | -2.064 | 3.909e-28 | 3.231e-27 | MKL2 |
| ENSG00000186280 | 1.483 | 1.925e-06 | 4.95e-06 | KDM4D |
| ENSG00000186281 | -1.583 | 8.765e-09 | 2.704e-08 | GPAT2 |
| ENSG00000186283 | -1.512 | 5.04e-25 | 3.658e-24 | TOR3A |
| ENSG00000186318 | -1.799 | 1.278e-27 | 1.035e-26 | BACE1 |
| ENSG00000186340 | -1.549 | 0.02357 | 0.03834 | THBS2 |
| ENSG00000186350 | -2.827 | 2.419e-23 | 1.625e-22 | RXRA |
| ENSG00000186352 | 3.047 | 2.656e-29 | 2.319e-28 | ANKRD37 |
| ENSG00000186354 | 1.373 | 0.002703 | 0.005087 | C9orf47 |
| ENSG00000186364 | 1.245 | 0.001956 | 0.003756 | NUDT17 |
| ENSG00000186399 | -2.095 | 0.01133 | 0.01947 | GOLGA8R |
| ENSG00000186409 | -1.071 | 9.417e-05 | 0.0002087 | CCDC30 |
| ENSG00000186432 | 1.468 | 1.578e-71 | 5.257e-70 | KPNA4 |
| ENSG00000186446 | -2.058 | 0.001653 | 0.0032 | ZNF501 |
| ENSG00000186472 | -2.25 | 3.504e-25 | 2.557e-24 | PCLO |
| ENSG00000186474 | 3.253 | 0.006908 | 0.01227 | KLK12 |
| ENSG00000186480 | 2.364 | 4.949e-88 | 2.487e-86 | INSIG1 |
| ENSG00000186493 | -1.178 | 0.0001265 | 0.0002763 | C5orf38 |
| ENSG00000186517 | 1.349 | 0.001753 | 0.003384 | ARHGAP30 |
| ENSG00000186523 | -2.104 | 1.495e-13 | 6.267e-13 | FAM86B1 |
| ENSG00000186529 | 1.305 | 1.133e-21 | 7.085e-21 | CYP4F3 |
| ENSG00000186532 | -2.696 | 9.519e-45 | 1.469e-43 | SMYD4 |
| ENSG00000186564 | -3.989 | 4.393e-20 | 2.541e-19 | FOXD2 |
| ENSG00000186591 | 1.397 | 2.815e-77 | 1.094e-75 | UBE2H |
| ENSG00000186603 | -4.569 | 9.715e-47 | 1.603e-45 | HPDL |
| ENSG00000186628 | 2.352 | 0.02116 | 0.03473 | FSD2 |
| ENSG00000186635 | -1.235 | 5.011e-23 | 3.305e-22 | ARAP1 |
| ENSG00000186638 | -2.356 | 4.149e-35 | 4.603e-34 | KIF24 |
| ENSG00000186642 | 1.637 | 5.241e-06 | 1.298e-05 | PDE2A |
| ENSG00000186654 | -1.054 | 5.05e-06 | 1.252e-05 | PRR5 |
| ENSG00000186665 | -2.849 | 1.222e-22 | 7.935e-22 | C17orf58 |
| ENSG00000186687 | -1.483 | 4.409e-30 | 3.947e-29 | LYRM7 |
| ENSG00000186714 | 4.177 | 1.253e-19 | 7.111e-19 | CCDC73 |
| ENSG00000186716 | -1.492 | 3.133e-43 | 4.629e-42 | BCR |
| ENSG00000186732 | 2.977 | 0.01899 | 0.03143 | MPPED1 |
| ENSG00000186767 | -3.106 | 3.235e-196 | 1.025e-193 | SPIN4 |
| ENSG00000186787 | -2.946 | 3.455e-16 | 1.656e-15 | SPIN2B |
| ENSG00000186812 | -2.015 | 0.003913 | 0.007185 | ZNF397 |
| ENSG00000186832 | 1.447 | 2.61e-23 | 1.749e-22 | KRT16 |
| ENSG00000186834 | -1.685 | 1.134e-30 | 1.04e-29 | HEXIM1 |
| ENSG00000186847 | 4.17 | 5.644e-10 | 1.893e-09 | KRT14 |
| ENSG00000186854 | 2 | 0.008691 | 0.0152 | TRABD2A |
| ENSG00000186866 | 1.056 | 7.153e-17 | 3.541e-16 | POFUT2 |
| ENSG00000186871 | -3.458 | 7.124e-118 | 6.681e-116 | ERCC6L |
| ENSG00000186907 | -1.281 | 0.001907 | 0.003667 | RTN4RL2 |
| ENSG00000186918 | -1.465 | 5.595e-26 | 4.261e-25 | ZNF395 |
| ENSG00000186919 | 3.339 | 3.415e-07 | 9.382e-07 | ZACN |
| ENSG00000186951 | -1.011 | 2.617e-10 | 8.981e-10 | PPARA |
| ENSG00000187010 | 2.806 | 1.208e-06 | 3.168e-06 | RHD |
| ENSG00000187024 | -1.322 | 1.768e-07 | 4.96e-07 | PTRH1 |
| ENSG00000187049 | -1.708 | 6.524e-14 | 2.781e-13 | TMEM216 |
| ENSG00000187066 | 1.394 | 2.333e-12 | 9.096e-12 | AP003068.6 |
| ENSG00000187097 | -1.004 | 1.844e-14 | 8.077e-14 | ENTPD5 |
| ENSG00000187123 | -2.052 | 5.212e-12 | 1.988e-11 | LYPD6 |
| ENSG00000187147 | -1.065 | 1.386e-23 | 9.409e-23 | RNF220 |
| ENSG00000187185 | 2.316 | 0.00229 | 0.00435 | CTD-2600O9.1 |
| ENSG00000187187 | 1.17 | 0.0141 | 0.02389 | ZNF546 |
| ENSG00000187193 | 3.856 | 1.48e-89 | 7.669e-88 | MT1X |
| ENSG00000187210 | -2.338 | 5.404e-20 | 3.116e-19 | GCNT1 |
| ENSG00000187240 | -1.147 | 6.429e-12 | 2.438e-11 | DYNC2H1 |
| ENSG00000187456 | -1.318 | 0.0001794 | 0.0003857 | RDM1 |
| ENSG00000187479 | 3.404 | 0.003786 | 0.00697 | C11orf96 |
| ENSG00000187486 | -1.849 | 0.026 | 0.04201 | KCNJ11 |
| ENSG00000187492 | -2.763 | 0.01693 | 0.02828 | CDHR4 |
| ENSG00000187498 | 2.22 | 1.192e-141 | 1.626e-139 | COL4A1 |
| ENSG00000187513 | 3.299 | 0.006793 | 0.01208 | GJA4 |
| ENSG00000187535 | -1.138 | 7.722e-12 | 2.915e-11 | IFT140 |
| ENSG00000187550 | -1.935 | 2.515e-19 | 1.404e-18 | SBK2 |
| ENSG00000187554 | -2.258 | 0.000854 | 0.001706 | TLR5 |
| ENSG00000187566 | -1.795 | 0.0003722 | 0.0007745 | NHLRC1 |
| ENSG00000187583 | -2.366 | 5.989e-18 | 3.137e-17 | PLEKHN1 |
| ENSG00000187608 | -1.028 | 0.000541 | 0.001106 | ISG15 |
| ENSG00000187626 | -2.07 | 7.986e-13 | 3.204e-12 | ZKSCAN4 |
| ENSG00000187627 | 2.419 | 3.357e-19 | 1.867e-18 | RGPD1 |
| ENSG00000187634 | -1.096 | 0.01166 | 0.02 | SAMD11 |
| ENSG00000187672 | 4.077 | 1.223e-13 | 5.148e-13 | ERC2 |
| ENSG00000187676 | -1.931 | 1.721e-28 | 1.448e-27 | B3GALTL |
| ENSG00000187695 | 2.392 | 7.023e-05 | 0.0001575 | RP11-723O4.6 |
| ENSG00000187713 | -2.745 | 4.063e-59 | 9.512e-58 | TMEM203 |
| ENSG00000187736 | -1.054 | 1.674e-05 | 3.966e-05 | NHEJ1 |
| ENSG00000187741 | -1.376 | 1.358e-24 | 9.68e-24 | FANCA |
| ENSG00000187775 | 1.717 | 5.928e-31 | 5.509e-30 | DNAH17 |
| ENSG00000187790 | -2.917 | 3.099e-57 | 6.835e-56 | FANCM |
| ENSG00000187792 | -2.505 | 1.107e-23 | 7.551e-23 | ZNF70 |
| ENSG00000187796 | -1.461 | 0.0001138 | 0.0002502 | CARD9 |
| ENSG00000187801 | -1.46 | 8.45e-05 | 0.0001879 | ZFP69B |
| ENSG00000187815 | -2.223 | 1.553e-06 | 4.026e-06 | ZFP69 |
| ENSG00000187824 | 1.483 | 0.0001395 | 0.0003033 | TMEM220 |
| ENSG00000187862 | 2.474 | 0.02386 | 0.03876 | TTC24 |
| ENSG00000187867 | -3.314 | 8.371e-26 | 6.318e-25 | PALM3 |
| ENSG00000187902 | 5.454 | 1.871e-52 | 3.601e-51 | SHISA7 |
| ENSG00000187944 | -2.526 | 0.009231 | 0.01608 | C2orf66 |
| ENSG00000187951 | -2.875 | 4.024e-68 | 1.239e-66 | ARHGAP11B |
| ENSG00000187961 | -2.435 | 6.346e-24 | 4.388e-23 | KLHL17 |
| ENSG00000187994 | -1.043 | 0.008213 | 0.01443 | RINL |
| ENSG00000188026 | -1.097 | 6.106e-10 | 2.042e-09 | RILPL1 |
| ENSG00000188042 | -2.485 | 4.407e-37 | 5.273e-36 | ARL4C |
| ENSG00000188051 | -3.171 | 0.00124 | 0.002435 | TMEM221 |
| ENSG00000188056 | 3.389 | 7.408e-23 | 4.848e-22 | TREML4 |
| ENSG00000188064 | -2.102 | 4.836e-117 | 4.388e-115 | WNT7B |
| ENSG00000188089 | 4.873 | 1.79e-06 | 4.614e-06 | PLA2G4E |
| ENSG00000188095 | -3.289 | 0.002789 | 0.005236 | MESP2 |
| ENSG00000188100 | 1.938 | 0.0001517 | 0.0003285 | FAM25A |
| ENSG00000188107 | -2.35 | 0.0004953 | 0.001017 | EYS |
| ENSG00000188130 | -2.371 | 4.497e-53 | 8.757e-52 | MAPK12 |
| ENSG00000188157 | -1.124 | 2.103e-23 | 1.416e-22 | AGRN |
| ENSG00000188167 | -1.921 | 8.588e-11 | 3.038e-10 | TMPPE |
| ENSG00000188175 | 4.89 | 5.907e-06 | 1.457e-05 | HEPACAM2 |
| ENSG00000188177 | -1.085 | 1.503e-07 | 4.247e-07 | ZC3H6 |
| ENSG00000188191 | -2.396 | 3.59e-72 | 1.215e-70 | PRKAR1B |
| ENSG00000188215 | 1.379 | 6.543e-12 | 2.48e-11 | DCUN1D3 |
| ENSG00000188223 | -4.349 | 0.001011 | 0.002005 | AC002398.9 |
| ENSG00000188227 | -2.433 | 0.006453 | 0.01151 | ZNF793 |
| ENSG00000188229 | -1.593 | 1.022e-24 | 7.31e-24 | TUBB4B |
| ENSG00000188242 | -2.314 | 7.758e-05 | 0.0001732 | CTD-2228K2.5 |
| ENSG00000188263 | 3.269 | 0.000777 | 0.001559 | IL17REL |
| ENSG00000188290 | -1.513 | 1.05e-10 | 3.697e-10 | HES4 |
| ENSG00000188295 | 1.412 | 1.788e-19 | 1.007e-18 | ZNF669 |
| ENSG00000188306 | -2.644 | 0.03059 | 0.04877 | LRRIQ4 |
| ENSG00000188316 | -2.187 | 0.00109 | 0.002155 | ENO4 |
| ENSG00000188322 | -4.149 | 1.403e-36 | 1.638e-35 | SBK1 |
| ENSG00000188368 | -1.348 | 0.005586 | 0.01005 | PRR19 |
| ENSG00000188386 | 4.072 | 0.0006232 | 0.001266 | PPP3R2 |
| ENSG00000188396 | 3.481 | 4.571e-27 | 3.621e-26 | TCTEX1D4 |
| ENSG00000188425 | 2.78 | 0.008031 | 0.01413 | NANOS2 |
| ENSG00000188501 | -1.696 | 0.02094 | 0.03439 | LCTL |
| ENSG00000188505 | -2.342 | 3.431e-25 | 2.508e-24 | NCCRP1 |
| ENSG00000188522 | 1.257 | 1.387e-38 | 1.755e-37 | FAM83G |
| ENSG00000188549 | -1.782 | 2.845e-31 | 2.686e-30 | C15orf52 |
| ENSG00000188582 | 2.47 | 0.003215 | 0.005978 | PAQR9 |
| ENSG00000188610 | -2.417 | 3.968e-50 | 7.154e-49 | FAM72B |
| ENSG00000188613 | -2.347 | 1.2e-22 | 7.8e-22 | NANOS1 |
| ENSG00000188629 | -2.21 | 2.683e-05 | 6.238e-05 | ZNF177 |
| ENSG00000188636 | -2.978 | 5.936e-64 | 1.623e-62 | LDOC1L |
| ENSG00000188647 | -1.093 | 1.149e-28 | 9.778e-28 | PTAR1 |
| ENSG00000188747 | -2.245 | 0.0002388 | 0.0005072 | NOXA1 |
| ENSG00000188761 | 2.865 | 4.21e-11 | 1.521e-10 | BCL2L15 |
| ENSG00000188766 | 3.598 | 5.977e-21 | 3.609e-20 | SPRED3 |
| ENSG00000188770 | 1.289 | 0.02691 | 0.04338 | OPTC |
| ENSG00000188779 | -2.76 | 0.02021 | 0.03331 | SKOR1 |
| ENSG00000188783 | 1.89 | 1.909e-06 | 4.912e-06 | PRELP |
| ENSG00000188807 | -1.673 | 1.131e-34 | 1.238e-33 | TMEM201 |
| ENSG00000188811 | -1.486 | 7.394e-25 | 5.32e-24 | NHLRC3 |
| ENSG00000188818 | 3.145 | 2.093e-45 | 3.299e-44 | ZDHHC11 |
| ENSG00000188878 | -2.223 | 8.745e-21 | 5.226e-20 | FBF1 |
| ENSG00000188886 | 6.263 | 1.195e-11 | 4.467e-11 | ASTL |
| ENSG00000188895 | -1.461 | 2.647e-24 | 1.861e-23 | MSL1 |
| ENSG00000188897 | 2.774 | 6.339e-08 | 1.837e-07 | CTD-3088G3.8 |
| ENSG00000188958 | 2.18 | 0.02392 | 0.03885 | UTS2B |
| ENSG00000188959 | 1.531 | 0.003551 | 0.006559 | C9orf152 |
| ENSG00000188981 | 1.492 | 0.02945 | 0.04711 | MSANTD1 |
| ENSG00000188986 | -2.195 | 5.026e-49 | 8.825e-48 | NELFB |
| ENSG00000188994 | 2.341 | 9.401e-56 | 1.977e-54 | ZNF292 |
| ENSG00000189001 | 3.234 | 6.131e-05 | 0.0001383 | SBSN |
| ENSG00000189007 | -1.887 | 3.113e-21 | 1.906e-20 | ADAT2 |
| ENSG00000189042 | -1.025 | 9.813e-09 | 3.015e-08 | ZNF567 |
| ENSG00000189046 | -2.39 | 3.7e-35 | 4.124e-34 | ALKBH2 |
| ENSG00000189056 | 1.543 | 6.046e-41 | 8.265e-40 | RELN |
| ENSG00000189057 | -3.143 | 3.623e-37 | 4.353e-36 | FAM111B |
| ENSG00000189060 | -4.814 | 7.478e-179 | 1.793e-176 | H1F0 |
| ENSG00000189090 | 2.381 | 0.008342 | 0.01463 | FAM25B |
| ENSG00000189108 | 1.052 | 0.001848 | 0.00356 | IL1RAPL2 |
| ENSG00000189120 | -2.249 | 1.379e-25 | 1.032e-24 | SP6 |
| ENSG00000189127 | -3.046 | 3.019e-29 | 2.627e-28 | ANKRD34B |
| ENSG00000189144 | -2.232 | 3.168e-05 | 7.327e-05 | ZNF573 |
| ENSG00000189157 | -1.529 | 0.0003433 | 0.0007174 | FAM47E |
| ENSG00000189159 | -1.586 | 2.513e-16 | 1.211e-15 | HN1 |
| ENSG00000189195 | -2.074 | 0.001304 | 0.002555 | BTBD8 |
| ENSG00000189221 | 1.582 | 2.149e-09 | 6.932e-09 | MAOA |
| ENSG00000189227 | -1.649 | 0.002232 | 0.004248 | C15orf61 |
| ENSG00000189233 | 1.565 | 0.01413 | 0.02393 | NUGGC |
| ENSG00000189252 | 7.317 | 9.446e-17 | 4.643e-16 | SPANXN3 |
| ENSG00000189269 | -1.525 | 0.0005298 | 0.001085 | C22orf43 |
| ENSG00000189280 | -2.777 | 3.18e-28 | 2.637e-27 | GJB5 |
| ENSG00000189319 | -1.206 | 4.665e-11 | 1.681e-10 | FAM53B |
| ENSG00000189320 | 3.166 | 0.002575 | 0.00486 | FAM180A |
| ENSG00000189337 | -1.714 | 6.34e-14 | 2.704e-13 | KAZN |
| ENSG00000189339 | -1.441 | 8.307e-15 | 3.711e-14 | SLC35E2B |
| ENSG00000189350 | 4.854 | 7.352e-13 | 2.952e-12 | FAM179A |
| ENSG00000189362 | -1.5 | 3.832e-15 | 1.74e-14 | TMEM194B |
| ENSG00000189367 | 3.371 | 0.01085 | 0.0187 | KIAA0408 |
| ENSG00000189377 | 1.058 | 0.0128 | 0.02179 | CXCL17 |
| ENSG00000196074 | -1.245 | 0.0004213 | 0.0008717 | SYCP2 |
| ENSG00000196091 | 4.038 | 0.0002271 | 0.0004836 | MYBPC1 |
| ENSG00000196110 | 1.729 | 3.01e-06 | 7.609e-06 | ZNF699 |
| ENSG00000196116 | -1.189 | 1.181e-17 | 6.085e-17 | TDRD7 |
| ENSG00000196123 | -1.858 | 1.066e-28 | 9.075e-28 | KIAA0895L |
| ENSG00000196132 | 4.017 | 0.0004761 | 0.0009786 | MYT1 |
| ENSG00000196151 | -1.498 | 0.0004321 | 0.0008928 | WDSUB1 |
| ENSG00000196152 | 1.89 | 9.769e-41 | 1.325e-39 | ZNF79 |
| ENSG00000196155 | -2.62 | 4.608e-44 | 6.975e-43 | PLEKHG4 |
| ENSG00000196172 | -3.053 | 3.249e-07 | 8.942e-07 | ZNF681 |
| ENSG00000196182 | 2.12 | 1.765e-63 | 4.77e-62 | STK40 |
| ENSG00000196187 | -1.19 | 6.964e-43 | 1.013e-41 | TMEM63A |
| ENSG00000196189 | -1.853 | 2.958e-28 | 2.456e-27 | SEMA4A |
| ENSG00000196227 | -3.975 | 1.082e-115 | 9.41e-114 | FAM217B |
| ENSG00000196230 | -1.82 | 1.223e-117 | 1.134e-115 | TUBB |
| ENSG00000196290 | -1.968 | 3.354e-38 | 4.196e-37 | NIF3L1 |
| ENSG00000196326 | 1.192 | 0.0004096 | 0.0008486 | AKR1CL1 |
| ENSG00000196337 | 1.365 | 0.0002088 | 0.0004462 | CGB7 |
| ENSG00000196338 | 1.105 | 0.0166 | 0.02775 | NLGN3 |
| ENSG00000196352 | 4.204 | 3.058e-66 | 8.973e-65 | CD55 |
| ENSG00000196361 | 5.087 | 2.058e-07 | 5.747e-07 | ELAVL3 |
| ENSG00000196363 | -1.527 | 1.218e-17 | 6.268e-17 | WDR5 |
| ENSG00000196367 | -1.474 | 3.917e-50 | 7.07e-49 | TRRAP |
| ENSG00000196371 | -2.536 | 3.018e-14 | 1.308e-13 | FUT4 |
| ENSG00000196372 | -5.554 | 3.139e-53 | 6.162e-52 | ASB13 |
| ENSG00000196378 | 2.231 | 2.444e-42 | 3.507e-41 | ZNF34 |
| ENSG00000196405 | -1.268 | 6.1e-12 | 2.319e-11 | EVL |
| ENSG00000196406 | 2.516 | 0.00677 | 0.01204 | SPANXD |
| ENSG00000196409 | -3.727 | 5.971e-12 | 2.271e-11 | ZNF658 |
| ENSG00000196411 | -2.562 | 1.018e-80 | 4.283e-79 | EPHB4 |
| ENSG00000196422 | -1.276 | 2.576e-15 | 1.178e-14 | PPP1R26 |
| ENSG00000196428 | 1.387 | 1.018e-58 | 2.357e-57 | TSC22D2 |
| ENSG00000196436 | 1.562 | 0.001933 | 0.003713 | NPIPB15 |
| ENSG00000196440 | -1.454 | 1.025e-08 | 3.144e-08 | ARMCX4 |
| ENSG00000196449 | 1.601 | 1.53e-08 | 4.627e-08 | YRDC |
| ENSG00000196453 | -2.128 | 1.055e-23 | 7.219e-23 | ZNF777 |
| ENSG00000196455 | -1.069 | 1.567e-16 | 7.613e-16 | PIK3R4 |
| ENSG00000196456 | -2.388 | 6.031e-10 | 2.018e-09 | ZNF775 |
| ENSG00000196458 | -2.056 | 3.757e-22 | 2.391e-21 | ZNF605 |
| ENSG00000196460 | 3.045 | 0.002887 | 0.005401 | RFX8 |
| ENSG00000196466 | -2.272 | 0.0004165 | 0.0008621 | ZNF799 |
| ENSG00000196497 | -1.024 | 6.676e-11 | 2.382e-10 | IPO4 |
| ENSG00000196498 | -1.371 | 2.933e-14 | 1.272e-13 | NCOR2 |
| ENSG00000196502 | -1.784 | 1.325e-49 | 2.356e-48 | SULT1A1 |
| ENSG00000196503 | 3.478 | 0.003094 | 0.00577 | ARL9 |
| ENSG00000196505 | 1.613 | 4.808e-59 | 1.121e-57 | GDAP2 |
| ENSG00000196535 | -1.495 | 1.055e-56 | 2.281e-55 | MYO18A |
| ENSG00000196544 | -2.312 | 8.489e-09 | 2.623e-08 | C17orf59 |
| ENSG00000196547 | -1.128 | 4.165e-25 | 3.03e-24 | MAN2A2 |
| ENSG00000196550 | -1.671 | 3.362e-25 | 2.458e-24 | FAM72A |
| ENSG00000196562 | -1.957 | 1.797e-56 | 3.858e-55 | SULF2 |
| ENSG00000196576 | -1.373 | 4.277e-60 | 1.051e-58 | PLXNB2 |
| ENSG00000196584 | -1.089 | 1.228e-11 | 4.591e-11 | XRCC2 |
| ENSG00000196605 | -1.215 | 0.00219 | 0.004175 | ZNF846 |
| ENSG00000196611 | 8.551 | 4.72e-21 | 2.864e-20 | MMP1 |
| ENSG00000196622 | -2.894 | 3.837e-07 | 1.05e-06 | RIMBP3 |
| ENSG00000196628 | -1.897 | 7.544e-30 | 6.707e-29 | TCF4 |
| ENSG00000196636 | -1.061 | 4.739e-06 | 1.178e-05 | ACN9 |
| ENSG00000196639 | 2.27 | 2.546e-69 | 8.002e-68 | HRH1 |
| ENSG00000196644 | 1.291 | 7.405e-05 | 0.0001656 | GPR89C |
| ENSG00000196652 | 1.092 | 4.428e-16 | 2.109e-15 | ZKSCAN5 |
| ENSG00000196653 | -1.268 | 0.002161 | 0.004123 | ZNF502 |
| ENSG00000196655 | -1.354 | 1.515e-09 | 4.941e-09 | TRAPPC4 |
| ENSG00000196659 | -2.474 | 3.586e-20 | 2.086e-19 | TTC30B |
| ENSG00000196663 | -1.876 | 1.829e-71 | 6.08e-70 | TECPR2 |
| ENSG00000196670 | -1.932 | 5.955e-60 | 1.457e-58 | ZFP62 |
| ENSG00000196684 | -2.046 | 2.788e-08 | 8.296e-08 | HSH2D |
| ENSG00000196693 | -1.85 | 3.923e-53 | 7.649e-52 | ZNF33B |
| ENSG00000196700 | -3.117 | 2.223e-135 | 2.806e-133 | ZNF512B |
| ENSG00000196705 | 1.151 | 1.072e-05 | 2.585e-05 | ZNF431 |
| ENSG00000196715 | -1.123 | 1.862e-39 | 2.426e-38 | VKORC1L1 |
| ENSG00000196724 | -2.529 | 2.374e-07 | 6.602e-07 | ZNF418 |
| ENSG00000196730 | -1.775 | 5.659e-40 | 7.48e-39 | DAPK1 |
| ENSG00000196739 | 1.89 | 2.413e-05 | 5.643e-05 | COL27A1 |
| ENSG00000196754 | 1.128 | 0.002787 | 0.005234 | S100A2 |
| ENSG00000196757 | -2.454 | 1.542e-32 | 1.54e-31 | ZNF700 |
| ENSG00000196776 | -1.059 | 1.864e-14 | 8.165e-14 | CD47 |
| ENSG00000196792 | 2.042 | 1.012e-89 | 5.291e-88 | STRN3 |
| ENSG00000196793 | -2.441 | 1.202e-39 | 1.576e-38 | ZNF239 |
| ENSG00000196812 | -1.686 | 2.036e-06 | 5.218e-06 | ZSCAN16 |
| ENSG00000196814 | -1.527 | 7.9e-11 | 2.801e-10 | MVB12B |
| ENSG00000196865 | -3.287 | 6.878e-149 | 1.089e-146 | NHLRC2 |
| ENSG00000196872 | -1.921 | 1.29e-27 | 1.044e-26 | KIAA1211L |
| ENSG00000196878 | 3.177 | 1.332e-121 | 1.364e-119 | LAMB3 |
| ENSG00000196911 | -1.908 | 1.853e-21 | 1.149e-20 | KPNA5 |
| ENSG00000196917 | -4.75 | 4.916e-20 | 2.84e-19 | HCAR1 |
| ENSG00000196934 | -3.921 | 6.989e-07 | 1.869e-06 | RIMBP3B |
| ENSG00000196954 | 2.308 | 6.857e-56 | 1.448e-54 | CASP4 |
| ENSG00000196966 | 1.914 | 2.15e-08 | 6.447e-08 | HIST1H3E |
| ENSG00000196968 | 1.855 | 1.053e-29 | 9.321e-29 | FUT11 |
| ENSG00000196987 | 1.694 | 0.02267 | 0.03699 | LCA10 |
| ENSG00000196993 | 1.096 | 2.022e-06 | 5.186e-06 | NPIPB9 |
| ENSG00000197024 | -1.562 | 1.041e-14 | 4.622e-14 | ZNF398 |
| ENSG00000197045 | -1.095 | 3.107e-20 | 1.81e-19 | GMFB |
| ENSG00000197046 | -2.379 | 0.002413 | 0.004571 | SIGLEC15 |
| ENSG00000197056 | -1.112 | 6.369e-11 | 2.276e-10 | ZMYM1 |
| ENSG00000197063 | 2.111 | 2.799e-65 | 8.033e-64 | MAFG |
| ENSG00000197070 | -1.771 | 5.802e-51 | 1.071e-49 | ARRDC1 |
| ENSG00000197077 | -1.719 | 1.686e-83 | 7.628e-82 | KIAA1671 |
| ENSG00000197106 | -2.764 | 1.854e-53 | 3.658e-52 | SLC6A17 |
| ENSG00000197119 | -1.11 | 4.789e-15 | 2.164e-14 | SLC25A29 |
| ENSG00000197121 | -1.471 | 2.252e-18 | 1.204e-17 | PGAP1 |
| ENSG00000197128 | -1.355 | 7.546e-11 | 2.681e-10 | ZNF772 |
| ENSG00000197134 | -2.845 | 0.0001188 | 0.0002602 | ZNF257 |
| ENSG00000197136 | -1.079 | 1.402e-31 | 1.344e-30 | PCNXL3 |
| ENSG00000197140 | 2.622 | 2.785e-16 | 1.339e-15 | ADAM32 |
| ENSG00000197170 | 1.452 | 2.114e-52 | 4.065e-51 | PSMD12 |
| ENSG00000197181 | 3.273 | 1.749e-07 | 4.91e-07 | PIWIL2 |
| ENSG00000197182 | -3.112 | 3.848e-21 | 2.346e-20 | FLJ27365 |
| ENSG00000197183 | -1.886 | 2.253e-21 | 1.389e-20 | C20orf112 |
| ENSG00000197191 | -3.162 | 2.857e-31 | 2.695e-30 | C9orf169 |
| ENSG00000197213 | 2.364 | 0.01606 | 0.02693 | ZSCAN5B |
| ENSG00000197217 | -1.126 | 1.338e-34 | 1.459e-33 | ENTPD4 |
| ENSG00000197223 | 1.399 | 3.02e-15 | 1.377e-14 | C1D |
| ENSG00000197226 | -1.546 | 4.475e-57 | 9.819e-56 | TBC1D9B |
| ENSG00000197256 | -2.427 | 1.406e-42 | 2.025e-41 | KANK2 |
| ENSG00000197261 | -1.424 | 8.276e-09 | 2.561e-08 | C6orf141 |
| ENSG00000197275 | -2.423 | 1.576e-72 | 5.377e-71 | RAD54B |
| ENSG00000197279 | 2.927 | 1.166e-190 | 3.435e-188 | ZNF165 |
| ENSG00000197283 | -1.385 | 4.044e-12 | 1.553e-11 | SYNGAP1 |
| ENSG00000197296 | -3.106 | 5.442e-54 | 1.088e-52 | FITM2 |
| ENSG00000197299 | -2.389 | 2.935e-67 | 8.861e-66 | BLM |
| ENSG00000197312 | -1.052 | 8.479e-34 | 8.89e-33 | DDI2 |
| ENSG00000197321 | -1.577 | 2.384e-57 | 5.294e-56 | SVIL |
| ENSG00000197343 | 1.12 | 2.33e-23 | 1.567e-22 | ZNF655 |
| ENSG00000197355 | -1.185 | 1.509e-08 | 4.568e-08 | UAP1L1 |
| ENSG00000197362 | -2.121 | 2.495e-23 | 1.674e-22 | ZNF786 |
| ENSG00000197363 | -1.914 | 5.206e-07 | 1.406e-06 | ZNF517 |
| ENSG00000197376 | 2.616 | 0.01415 | 0.02395 | OR8S1 |
| ENSG00000197380 | 4.079 | 6.118e-07 | 1.643e-06 | DACT3 |
| ENSG00000197381 | -1.001 | 3.5e-10 | 1.191e-09 | ADARB1 |
| ENSG00000197385 | -1.788 | 2.125e-05 | 4.991e-05 | ZNF860 |
| ENSG00000197405 | 2.821 | 3.895e-31 | 3.65e-30 | C5AR1 |
| ENSG00000197406 | -1.266 | 0.02058 | 0.03386 | DIO3 |
| ENSG00000197409 | 1.858 | 0.02011 | 0.03315 | HIST1H3D |
| ENSG00000197415 | -1.286 | 8.066e-06 | 1.966e-05 | VEPH1 |
| ENSG00000197417 | -1.62 | 3.05e-25 | 2.237e-24 | SHPK |
| ENSG00000197429 | -1.541 | 1.557e-29 | 1.371e-28 | IPP |
| ENSG00000197442 | -2.647 | 3.817e-65 | 1.086e-63 | MAP3K5 |
| ENSG00000197444 | -1.19 | 3.85e-07 | 1.053e-06 | OGDHL |
| ENSG00000197446 | 2.962 | 0.02073 | 0.03408 | CYP2F1 |
| ENSG00000197457 | -1.129 | 2.157e-09 | 6.955e-09 | STMN3 |
| ENSG00000197483 | 1.615 | 1.488e-37 | 1.817e-36 | ZNF628 |
| ENSG00000197496 | -2.894 | 1.066e-36 | 1.25e-35 | SLC2A10 |
| ENSG00000197506 | 3.036 | 2.569e-27 | 2.059e-26 | SLC28A3 |
| ENSG00000197536 | -2.317 | 1.012e-05 | 2.447e-05 | C5orf56 |
| ENSG00000197540 | 2.789 | 0.01973 | 0.03257 | GZMM |
| ENSG00000197557 | -2.136 | 3.163e-18 | 1.678e-17 | TTC30A |
| ENSG00000197562 | -1.151 | 3.802e-07 | 1.041e-06 | RAB40C |
| ENSG00000197576 | -2.789 | 0.02914 | 0.04663 | HOXA4 |
| ENSG00000197579 | -1.74 | 1.104e-30 | 1.013e-29 | TOPORS |
| ENSG00000197580 | 1.091 | 0.0001794 | 0.0003858 | BCO2 |
| ENSG00000197586 | -1.283 | 1.028e-12 | 4.102e-12 | ENTPD6 |
| ENSG00000197587 | -1.49 | 0.01106 | 0.01904 | DMBX1 |
| ENSG00000197603 | -2.14 | 1.151e-27 | 9.359e-27 | C5orf42 |
| ENSG00000197604 | -2.713 | 0.02234 | 0.0365 | AC022532.1 |
| ENSG00000197608 | 1.032 | 4.537e-21 | 2.755e-20 | ZNF841 |
| ENSG00000197632 | 7.097 | 5.266e-16 | 2.495e-15 | SERPINB2 |
| ENSG00000197694 | -1.038 | 1.382e-28 | 1.171e-27 | SPTAN1 |
| ENSG00000197712 | -1.663 | 1.782e-42 | 2.563e-41 | FAM114A1 |
| ENSG00000197714 | 2.137 | 4.293e-31 | 4.015e-30 | ZNF460 |
| ENSG00000197724 | -1.203 | 2.75e-20 | 1.604e-19 | PHF2 |
| ENSG00000197748 | 1.816 | 2.726e-20 | 1.591e-19 | WDR96 |
| ENSG00000197757 | -2.836 | 2.68e-20 | 1.564e-19 | HOXC6 |
| ENSG00000197763 | -2.714 | 1.612e-32 | 1.607e-31 | TXNRD3 |
| ENSG00000197768 | -2.696 | 0.01871 | 0.03101 | C9orf173 |
| ENSG00000197769 | -2.678 | 4.063e-05 | 9.318e-05 | MAP1LC3C |
| ENSG00000197771 | -1.773 | 2.74e-83 | 1.23e-81 | MCMBP |
| ENSG00000197774 | -1.651 | 2.447e-24 | 1.723e-23 | EME2 |
| ENSG00000197780 | 2.214 | 1.045e-46 | 1.723e-45 | TAF13 |
| ENSG00000197785 | -1.163 | 7.048e-23 | 4.618e-22 | ATAD3A |
| ENSG00000197808 | 1.107 | 9.663e-13 | 3.857e-12 | ZNF461 |
| ENSG00000197816 | -2.466 | 2.653e-17 | 1.343e-16 | CCDC180 |
| ENSG00000197841 | -1.051 | 2.808e-07 | 7.764e-07 | ZNF181 |
| ENSG00000197852 | -1.359 | 0.007395 | 0.01308 | FAM212B |
| ENSG00000197872 | -2.159 | 8.461e-28 | 6.921e-27 | FAM49A |
| ENSG00000197893 | 5.955 | 3.097e-10 | 1.058e-09 | NRAP |
| ENSG00000197905 | -1.079 | 2.877e-17 | 1.454e-16 | TEAD4 |
| ENSG00000197910 | 3.012 | 0.01008 | 0.01746 | FAM25G |
| ENSG00000197915 | 3.675 | 8.88e-37 | 1.047e-35 | HRNR |
| ENSG00000197919 | 2.377 | 0.002791 | 0.005239 | IFNA1 |
| ENSG00000197927 | -2.815 | 0.0146 | 0.02464 | C2orf27A |
| ENSG00000197932 | -4.227 | 1.511e-67 | 4.604e-66 | F8A1 |
| ENSG00000197935 | -3.928 | 1.201e-28 | 1.019e-27 | ZNF311 |
| ENSG00000197943 | -1.207 | 4.27e-06 | 1.067e-05 | PLCG2 |
| ENSG00000197951 | -1.747 | 7.725e-07 | 2.059e-06 | ZNF71 |
| ENSG00000197961 | 1.091 | 7.853e-28 | 6.43e-27 | ZNF121 |
| ENSG00000197965 | -1.306 | 6.404e-43 | 9.323e-42 | MPZL1 |
| ENSG00000197976 | 1.235 | 7.134e-25 | 5.135e-24 | AKAP17A |
| ENSG00000198010 | 4.384 | 7.64e-05 | 0.0001707 | DLGAP2 |
| ENSG00000198018 | 2.013 | 8.029e-42 | 1.138e-40 | ENTPD7 |
| ENSG00000198019 | 4.277 | 1.355e-06 | 3.535e-06 | FCGR1B |
| ENSG00000198021 | 3.604 | 2.903e-07 | 8.021e-07 | SPANXA1 |
| ENSG00000198028 | 1.326 | 0.02379 | 0.03867 | ZNF560 |
| ENSG00000198040 | -1.138 | 6.732e-19 | 3.684e-18 | ZNF84 |
| ENSG00000198053 | -1.443 | 1.714e-21 | 1.064e-20 | SIRPA |
| ENSG00000198056 | -1.461 | 3.942e-34 | 4.215e-33 | PRIM1 |
| ENSG00000198060 | 1.021 | 1.94e-11 | 7.169e-11 | 5-Mar |
| ENSG00000198077 | 3.28 | 0.0006366 | 0.001292 | CYP2A7 |
| ENSG00000198081 | -3.432 | 1.696e-50 | 3.084e-49 | ZBTB14 |
| ENSG00000198082 | 2.368 | 0.006565 | 0.01169 | H2AFB1 |
| ENSG00000198089 | -1.639 | 2.896e-27 | 2.315e-26 | SFI1 |
| ENSG00000198092 | 3.532 | 0.000135 | 0.0002939 | TMPRSS11F |
| ENSG00000198093 | -1.659 | 4.174e-20 | 2.42e-19 | ZNF649 |
| ENSG00000198105 | -1.959 | 3.572e-11 | 1.297e-10 | ZNF248 |
| ENSG00000198113 | -1.29 | 2.502e-12 | 9.729e-12 | TOR4A |
| ENSG00000198121 | -3.421 | 8.745e-33 | 8.801e-32 | LPAR1 |
| ENSG00000198131 | -1.447 | 6.804e-31 | 6.31e-30 | ZNF544 |
| ENSG00000198133 | -2.453 | 0.01869 | 0.03099 | TMEM229B |
| ENSG00000198142 | 1.015 | 5.638e-18 | 2.958e-17 | SOWAHC |
| ENSG00000198156 | 1.742 | 1.078e-08 | 3.299e-08 | NPIPB6 |
| ENSG00000198168 | -1.053 | 1.378e-20 | 8.164e-20 | SVIP |
| ENSG00000198176 | -1.138 | 3.856e-56 | 8.214e-55 | TFDP1 |
| ENSG00000198182 | -2.213 | 5.083e-08 | 1.483e-07 | ZNF607 |
| ENSG00000198205 | -2.538 | 6.094e-23 | 4.004e-22 | ZXDA |
| ENSG00000198208 | -2.013 | 1.75e-13 | 7.307e-13 | RPS6KL1 |
| ENSG00000198246 | -3.397 | 2.769e-82 | 1.207e-80 | SLC29A3 |
| ENSG00000198298 | -1.061 | 9.054e-07 | 2.4e-06 | ZNF485 |
| ENSG00000198315 | -2.462 | 0.0001606 | 0.0003469 | ZKSCAN8 |
| ENSG00000198324 | -2.427 | 1.678e-26 | 1.3e-25 | FAM109A |
| ENSG00000198331 | -2.36 | 9.578e-34 | 1.002e-32 | HYLS1 |
| ENSG00000198339 | 3.455 | 7.768e-12 | 2.931e-11 | HIST1H4I |
| ENSG00000198417 | 3.433 | 2.637e-83 | 1.187e-81 | MT1F |
| ENSG00000198420 | -1.972 | 1.509e-61 | 3.861e-60 | FAM115A |
| ENSG00000198431 | 1.039 | 5.1e-21 | 3.091e-20 | TXNRD1 |
| ENSG00000198435 | 2.3 | 4.103e-112 | 3.36e-110 | NRARP |
| ENSG00000198440 | 1.488 | 0.0006067 | 0.001234 | ZNF583 |
| ENSG00000198444 | -4.116 | 5.167e-47 | 8.587e-46 | F8A2 |
| ENSG00000198477 | -3.482 | 1.356e-44 | 2.087e-43 | ZNF280B |
| ENSG00000198483 | -3.031 | 1.756e-08 | 5.294e-08 | ANKRD35 |
| ENSG00000198498 | -1.067 | 3.612e-11 | 1.312e-10 | TMA16 |
| ENSG00000198513 | -2.367 | 3.972e-08 | 1.169e-07 | ATL1 |
| ENSG00000198517 | 1.942 | 7.559e-25 | 5.434e-24 | MAFK |
| ENSG00000198518 | 1.321 | 0.01818 | 0.03021 | HIST1H4E |
| ENSG00000198522 | 1.353 | 7.861e-41 | 1.069e-39 | GPN1 |
| ENSG00000198551 | -1.336 | 8.129e-15 | 3.634e-14 | ZNF627 |
| ENSG00000198553 | -2.399 | 0.004328 | 0.007903 | KCNRG |
| ENSG00000198554 | -2.212 | 3.535e-69 | 1.105e-67 | WDHD1 |
| ENSG00000198561 | -1.024 | 6.439e-31 | 5.978e-30 | CTNND1 |
| ENSG00000198573 | 4.555 | 4.499e-07 | 1.223e-06 | SPANXC |
| ENSG00000198576 | 8.582 | 2.353e-64 | 6.551e-63 | ARC |
| ENSG00000198585 | -2.558 | 7.227e-73 | 2.501e-71 | NUDT16 |
| ENSG00000198610 | 2.073 | 2.312e-09 | 7.432e-09 | AKR1C4 |
| ENSG00000198626 | 2.051 | 0.008236 | 0.01446 | RYR2 |
| ENSG00000198642 | -1.018 | 2.414e-10 | 8.302e-10 | KLHL9 |
| ENSG00000198650 | 4.474 | 2.351e-16 | 1.135e-15 | TAT |
| ENSG00000198680 | -2.887 | 2.263e-18 | 1.21e-17 | TUSC1 |
| ENSG00000198682 | -1.073 | 6.208e-09 | 1.938e-08 | PAPSS2 |
| ENSG00000198689 | -1.144 | 8.541e-23 | 5.581e-22 | SLC9A6 |
| ENSG00000198690 | -2.234 | 1.489e-41 | 2.089e-40 | FAN1 |
| ENSG00000198695 | 1.299 | 0.02884 | 0.04618 | MT-ND6 |
| ENSG00000198700 | -1.355 | 7.093e-33 | 7.19e-32 | IPO9 |
| ENSG00000198712 | 1.043 | 0.0261 | 0.04216 | MT-CO2 |
| ENSG00000198719 | 4.117 | 0.0003375 | 0.0007065 | DLL1 |
| ENSG00000198722 | -1.041 | 6.595e-05 | 0.0001482 | UNC13B |
| ENSG00000198728 | -2.001 | 9.416e-44 | 1.414e-42 | LDB1 |
| ENSG00000198732 | 3.247 | 0.01871 | 0.03101 | SMOC1 |
| ENSG00000198740 | -1.537 | 1.702e-44 | 2.609e-43 | ZNF652 |
| ENSG00000198742 | 1.585 | 1.593e-101 | 1.074e-99 | SMURF1 |
| ENSG00000198746 | 1.106 | 1.61e-09 | 5.246e-09 | GPATCH3 |
| ENSG00000198753 | -1.279 | 4.136e-20 | 2.4e-19 | PLXNB3 |
| ENSG00000198771 | 4.565 | 2.473e-05 | 5.773e-05 | RCSD1 |
| ENSG00000198774 | 2.745 | 6.135e-09 | 1.916e-08 | RASSF9 |
| ENSG00000198780 | -1.256 | 8.633e-15 | 3.855e-14 | FAM169A |
| ENSG00000198783 | 1.457 | 1.19e-33 | 1.24e-32 | ZNF830 |
| ENSG00000198788 | 4.795 | 1.129e-05 | 2.717e-05 | MUC2 |
| ENSG00000198793 | -1.038 | 4.266e-30 | 3.823e-29 | MTOR |
| ENSG00000198794 | -2.589 | 2.557e-41 | 3.547e-40 | SCAMP5 |
| ENSG00000198804 | 1.116 | 0.02671 | 0.04308 | MT-CO1 |
| ENSG00000198824 | -2.165 | 5.68e-22 | 3.585e-21 | CHAMP1 |
| ENSG00000198825 | -1.176 | 8.079e-11 | 2.862e-10 | INPP5F |
| ENSG00000198826 | -2.821 | 1.031e-188 | 2.984e-186 | ARHGAP11A |
| ENSG00000198829 | 3.771 | 1.763e-26 | 1.364e-25 | SUCNR1 |
| ENSG00000198837 | -3.4 | 5.599e-120 | 5.561e-118 | DENND4B |
| ENSG00000198839 | 1.753 | 5.025e-36 | 5.766e-35 | ZNF277 |
| ENSG00000198853 | 1.615 | 3.523e-61 | 8.933e-60 | RUSC2 |
| ENSG00000198855 | 2.138 | 5.068e-23 | 3.34e-22 | FICD |
| ENSG00000198870 | 2.945 | 6.056e-06 | 1.492e-05 | C9orf96 |
| ENSG00000198879 | -1.071 | 4.248e-06 | 1.062e-05 | SFMBT2 |
| ENSG00000198888 | 1.31 | 0.02037 | 0.03356 | MT-ND1 |
| ENSG00000198890 | -4.142 | 2.406e-107 | 1.795e-105 | PRMT6 |
| ENSG00000198894 | -2.28 | 4.581e-88 | 2.309e-86 | KIAA1737 |
| ENSG00000198900 | 1.453 | 2.297e-25 | 1.695e-24 | TOP1 |
| ENSG00000198901 | -1.61 | 3.976e-35 | 4.42e-34 | PRC1 |
| ENSG00000198912 | -1.331 | 1.942e-13 | 8.086e-13 | C1orf174 |
| ENSG00000198915 | -1.815 | 3.973e-23 | 2.636e-22 | RASGEF1A |
| ENSG00000198917 | -1.77 | 1.977e-30 | 1.794e-29 | C9orf114 |
| ENSG00000198924 | -3.038 | 1.029e-83 | 4.692e-82 | DCLRE1A |
| ENSG00000198934 | -2.991 | 9.169e-12 | 3.448e-11 | MAGEE1 |
| ENSG00000198944 | 2.37 | 0.0004332 | 0.0008949 | SOWAHA |
| ENSG00000198945 | -2.27 | 3.718e-17 | 1.869e-16 | L3MBTL3 |
| ENSG00000198948 | -2.046 | 2.537e-28 | 2.115e-27 | MFAP3L |
| ENSG00000198954 | -2.023 | 1.106e-53 | 2.192e-52 | KIAA1279 |
| ENSG00000198960 | -2.554 | 7.573e-30 | 6.729e-29 | ARMCX6 |
| ENSG00000203392 | -2.386 | 1.893e-06 | 4.873e-06 | AC105020.1 |
| ENSG00000203485 | -1.548 | 7.931e-30 | 7.043e-29 | INF2 |
| ENSG00000203667 | 1.724 | 2.024e-26 | 1.563e-25 | COX20 |
| ENSG00000203668 | -1.378 | 1.643e-62 | 4.358e-61 | CHML |
| ENSG00000203684 | 3.019 | 0.0004388 | 0.0009059 | IBA57-AS1 |
| ENSG00000203705 | -1.428 | 1.293e-14 | 5.699e-14 | TATDN3 |
| ENSG00000203724 | -1.343 | 0.001389 | 0.002715 | C1orf53 |
| ENSG00000203778 | -1.102 | 6.715e-06 | 1.648e-05 | FAM229B |
| ENSG00000203791 | -1.476 | 2.734e-22 | 1.751e-21 | METTL10 |
| ENSG00000203804 | 3.619 | 3.031e-14 | 1.313e-13 | ADAMTSL4-AS1 |
| ENSG00000203812 | 1.647 | 4.912e-06 | 1.218e-05 | HIST2H2AA4 |
| ENSG00000203814 | 4.49 | 7.693e-60 | 1.869e-58 | HIST2H2BF |
| ENSG00000203817 | -2.198 | 3.319e-11 | 1.208e-10 | FAM72C |
| ENSG00000203857 | -2.257 | 8.078e-15 | 3.612e-14 | HSD3B1 |
| ENSG00000203867 | -2.594 | 1.931e-11 | 7.138e-11 | RBM20 |
| ENSG00000203872 | -3.477 | 1.277e-06 | 3.339e-06 | C6orf163 |
| ENSG00000203880 | -1.265 | 2.673e-31 | 2.529e-30 | PCMTD2 |
| ENSG00000203907 | -1.877 | 0.003467 | 0.006413 | OOEP |
| ENSG00000203923 | 6.585 | 1.856e-13 | 7.739e-13 | SPANXN1 |
| ENSG00000203926 | 4.164 | 1.177e-12 | 4.676e-12 | SPANXA2 |
| ENSG00000203952 | -2.048 | 0.02983 | 0.04766 | CCDC160 |
| ENSG00000203995 | -2.882 | 6.054e-31 | 5.624e-30 | ZYG11A |
| ENSG00000204021 | 1.36 | 3.163e-05 | 7.316e-05 | LIPK |
| ENSG00000204052 | 2.901 | 3.645e-07 | 9.99e-07 | LRRC73 |
| ENSG00000204060 | -3.27 | 2.824e-10 | 9.671e-10 | FOXO6 |
| ENSG00000204103 | 2.982 | 1.714e-25 | 1.277e-24 | MAFB |
| ENSG00000204104 | -1.519 | 2.19e-33 | 2.254e-32 | TRAF3IP1 |
| ENSG00000204138 | -1.497 | 8.447e-08 | 2.427e-07 | PHACTR4 |
| ENSG00000204147 | -1.405 | 1.213e-08 | 3.698e-08 | ASAH2B |
| ENSG00000204149 | 1.065 | 1.129e-12 | 4.489e-12 | AGAP6 |
| ENSG00000204174 | -2.727 | 1.328e-18 | 7.187e-18 | NPY4R |
| ENSG00000204175 | -3.238 | 2.813e-14 | 1.223e-13 | GPRIN2 |
| ENSG00000204176 | -1.169 | 2.726e-13 | 1.126e-12 | SYT15 |
| ENSG00000204178 | 1.641 | 4.11e-34 | 4.385e-33 | TMEM57 |
| ENSG00000204179 | -1.589 | 4.27e-16 | 2.037e-15 | PTPN20A |
| ENSG00000204231 | -1.534 | 8.289e-17 | 4.093e-16 | RXRB |
| ENSG00000204237 | -2.464 | 1.441e-15 | 6.662e-15 | OXLD1 |
| ENSG00000204248 | 1.179 | 0.002803 | 0.005258 | COL11A2 |
| ENSG00000204252 | 3.072 | 0.01318 | 0.02241 | HLA-DOA |
| ENSG00000204264 | -1.028 | 5.503e-09 | 1.724e-08 | PSMB8 |
| ENSG00000204271 | -1.455 | 1.201e-08 | 3.664e-08 | SPIN3 |
| ENSG00000204291 | 2.09 | 8.612e-24 | 5.915e-23 | COL15A1 |
| ENSG00000204304 | -2.069 | 1.3e-49 | 2.313e-48 | PBX2 |
| ENSG00000204305 | -1.125 | 0.002758 | 0.005182 | AGER |
| ENSG00000204311 | -1.175 | 0.02416 | 0.03923 | DFNB59 |
| ENSG00000204314 | -1.687 | 6.512e-07 | 1.745e-06 | PRRT1 |
| ENSG00000204315 | -2.954 | 9.229e-20 | 5.265e-19 | FKBPL |
| ENSG00000204316 | -1.153 | 3.588e-16 | 1.717e-15 | MRPL38 |
| ENSG00000204323 | 1.827 | 0.0006483 | 0.001315 | SMIM5 |
| ENSG00000204363 | 5.506 | 2.201e-08 | 6.592e-08 | SPANXN5 |
| ENSG00000204366 | -3.002 | 1.525e-28 | 1.286e-27 | ZBTB12 |
| ENSG00000204371 | -1.534 | 1.693e-50 | 3.082e-49 | EHMT2 |
| ENSG00000204381 | -1.681 | 3.029e-05 | 7.016e-05 | LAYN |
| ENSG00000204388 | 6.309 | 0 | 0 | HSPA1B |
| ENSG00000204389 | 6.371 | 0 | 0 | HSPA1A |
| ENSG00000204390 | 4.158 | 9.242e-133 | 1.132e-130 | HSPA1L |
| ENSG00000204392 | -1.752 | 2.145e-35 | 2.413e-34 | LSM2 |
| ENSG00000204403 | 3.39 | 0.004536 | 0.008248 | CASP12 |
| ENSG00000204410 | -1.265 | 1.817e-20 | 1.071e-19 | MSH5 |
| ENSG00000204428 | 1.045 | 0.01422 | 0.02407 | LY6G5C |
| ENSG00000204439 | -2.385 | 3.946e-26 | 3.018e-25 | C6orf47 |
| ENSG00000204442 | 1.019 | 0.02262 | 0.03693 | FAM155A |
| ENSG00000204469 | -1.422 | 5.719e-67 | 1.714e-65 | PRRC2A |
| ENSG00000204514 | 1.101 | 1.963e-10 | 6.787e-10 | ZNF814 |
| ENSG00000204516 | -1.392 | 1.266e-36 | 1.48e-35 | MICB |
| ENSG00000204524 | 1.15 | 7.037e-07 | 1.881e-06 | ZNF805 |
| ENSG00000204531 | -1.47 | 0.02872 | 0.04602 | POU5F1 |
| ENSG00000204536 | -2.238 | 2.256e-25 | 1.666e-24 | CCHCR1 |
| ENSG00000204540 | 1.119 | 8.192e-09 | 2.537e-08 | PSORS1C1 |
| ENSG00000204560 | -1.931 | 2.832e-41 | 3.916e-40 | DHX16 |
| ENSG00000204564 | -1.491 | 2.281e-14 | 9.943e-14 | C6orf136 |
| ENSG00000204569 | 1.912 | 2.349e-44 | 3.592e-43 | PPP1R10 |
| ENSG00000204577 | 2.682 | 0.003442 | 0.00637 | LILRB3 |
| ENSG00000204580 | -1.132 | 3.303e-12 | 1.274e-11 | DDR1 |
| ENSG00000204610 | 4.171 | 0.0001076 | 0.0002373 | TRIM15 |
| ENSG00000204611 | -2.545 | 1.221e-12 | 4.84e-12 | ZNF616 |
| ENSG00000204616 | -3.27 | 1.03e-10 | 3.627e-10 | TRIM31 |
| ENSG00000204618 | 1.013 | 0.0002052 | 0.0004389 | RNF39 |
| ENSG00000204620 | 2.408 | 2.528e-08 | 7.544e-08 | AC115618.1 |
| ENSG00000204644 | 2.536 | 4.772e-14 | 2.05e-13 | ZFP57 |
| ENSG00000204653 | 1.124 | 0.02828 | 0.04539 | ASPDH |
| ENSG00000204677 | 3.232 | 0.008729 | 0.01526 | FAM153C |
| ENSG00000204682 | -4.262 | 2.138e-28 | 1.79e-27 | CASC10 |
| ENSG00000204767 | -3.242 | 2.207e-05 | 5.177e-05 | FAM196B |
| ENSG00000204839 | -1.245 | 1.441e-24 | 1.025e-23 | MROH6 |
| ENSG00000204856 | -2.171 | 1.158e-23 | 7.894e-23 | FAM216A |
| ENSG00000204922 | -1.436 | 1.169e-16 | 5.718e-16 | C11orf83 |
| ENSG00000204923 | 1.673 | 8.849e-13 | 3.539e-12 | FBXO48 |
| ENSG00000204941 | -1.611 | 1.213e-09 | 3.994e-09 | PSG5 |
| ENSG00000204946 | -3.059 | 2.874e-86 | 1.394e-84 | ZNF783 |
| ENSG00000204952 | 3.81 | 2.634e-05 | 6.132e-05 | FBXO47 |
| ENSG00000204957 | 3.648 | 1.273e-26 | 9.91e-26 | AC006486.1 |
| ENSG00000204963 | -2.925 | 0.01095 | 0.01886 | PCDHA7 |
| ENSG00000204977 | -1.656 | 1.26e-17 | 6.479e-17 | TRIM13 |
| ENSG00000204978 | 2.806 | 0.008627 | 0.0151 | C19orf69 |
| ENSG00000205060 | -2.102 | 8.746e-56 | 1.844e-54 | SLC35B4 |
| ENSG00000205078 | 1.699 | 0.0008519 | 0.001702 | SYCE1L |
| ENSG00000205084 | -2.625 | 7.646e-39 | 9.805e-38 | TMEM231 |
| ENSG00000205097 | 3.425 | 0.003966 | 0.007278 | FRG2 |
| ENSG00000205138 | -3.057 | 6.808e-18 | 3.553e-17 | SDHAF1 |
| ENSG00000205177 | 4.991 | 8.022e-07 | 2.136e-06 | C11orf91 |
| ENSG00000205208 | -1.893 | 8.519e-42 | 1.204e-40 | C4orf46 |
| ENSG00000205212 | 4.124 | 6.175e-06 | 1.521e-05 | CCDC144NL |
| ENSG00000205213 | -1.009 | 1.557e-24 | 1.107e-23 | LGR4 |
| ENSG00000205269 | -2.23 | 9.669e-40 | 1.272e-38 | TMEM170B |
| ENSG00000205309 | -2.159 | 0.001096 | 0.002166 | NT5M |
| ENSG00000205356 | -1.119 | 3.711e-06 | 9.307e-06 | TECPR1 |
| ENSG00000205362 | 2.597 | 0.0001055 | 0.0002329 | MT1A |
| ENSG00000205413 | -2.842 | 4.511e-94 | 2.629e-92 | SAMD9 |
| ENSG00000205420 | 1.414 | 1.467e-14 | 6.454e-14 | KRT6A |
| ENSG00000205476 | -2.311 | 1.191e-60 | 2.971e-59 | CCDC85C |
| ENSG00000205517 | -1.339 | 2.493e-30 | 2.255e-29 | RGL3 |
| ENSG00000205560 | -1.568 | 2.836e-13 | 1.169e-12 | CPT1B |
| ENSG00000205571 | 1.136 | 9.268e-06 | 2.249e-05 | SMN2 |
| ENSG00000205572 | -1.599 | 3.958e-09 | 1.252e-08 | SERF1B |
| ENSG00000205592 | 4.014 | 0.0004101 | 0.0008497 | MUC19 |
| ENSG00000205593 | -1.614 | 4.822e-10 | 1.625e-09 | DENND6B |
| ENSG00000205595 | 3.129 | 5.794e-25 | 4.19e-24 | AREGB |
| ENSG00000205643 | -3.189 | 4.941e-22 | 3.128e-21 | CDPF1 |
| ENSG00000205664 | -1.135 | 6.31e-13 | 2.544e-12 | RP11-706O15.1 |
| ENSG00000205693 | -2.464 | 0.00472 | 0.008568 | MANSC4 |
| ENSG00000205730 | -4.172 | 2.008e-107 | 1.505e-105 | ITPRIPL2 |
| ENSG00000205740 | 1.975 | 0.003535 | 0.006532 | AL359878.1 |
| ENSG00000205765 | 1.137 | 1.145e-12 | 4.549e-12 | C5orf51 |
| ENSG00000205808 | -2.939 | 3.568e-25 | 2.603e-24 | PPAPDC2 |
| ENSG00000205835 | 3.52 | 0.004926 | 0.008925 | GMNC |
| ENSG00000205856 | 4.122 | 0.000126 | 0.0002753 | C22orf42 |
| ENSG00000205867 | 3.065 | 0.02461 | 0.03991 | KRTAP5-2 |
| ENSG00000205869 | 4.099 | 2.497e-06 | 6.349e-06 | KRTAP5-1 |
| ENSG00000205978 | -3.096 | 2.642e-25 | 1.944e-24 | NYNRIN |
| ENSG00000205981 | -1.151 | 4.277e-14 | 1.841e-13 | DNAJC19 |
| ENSG00000206013 | 5.564 | 1.278e-08 | 3.891e-08 | IFITM5 |
| ENSG00000206044 | -1.512 | 0.01748 | 0.02913 | AC005841.1 |
| ENSG00000206053 | -1.605 | 6.615e-44 | 9.994e-43 | HN1L |
| ENSG00000206075 | 1.392 | 3.166e-39 | 4.095e-38 | SERPINB5 |
| ENSG00000206077 | 2.046 | 2.741e-05 | 6.369e-05 | ZDHHC11B |
| ENSG00000206127 | -1.759 | 0.009157 | 0.01596 | GOLGA8O |
| ENSG00000206150 | 2.713 | 0.02739 | 0.04406 | RNASE13 |
| ENSG00000206418 | -1.013 | 1.589e-22 | 1.026e-21 | RAB12 |
| ENSG00000206530 | -2.473 | 3.53e-66 | 1.031e-64 | WDR52 |
| ENSG00000206532 | 3.18 | 0.0001885 | 0.0004044 | RP11-553A10.1 |
| ENSG00000206535 | -1.8 | 1.532e-07 | 4.325e-07 | LNP1 |
| ENSG00000206538 | 1.258 | 4.949e-30 | 4.414e-29 | VGLL3 |
| ENSG00000206559 | 2.213 | 0.01306 | 0.02222 | ZCWPW2 |
| ENSG00000211448 | -1.25 | 7.516e-64 | 2.048e-62 | DIO2 |
| ENSG00000211455 | 1.146 | 1.363e-42 | 1.964e-41 | STK38L |
| ENSG00000211456 | -1.612 | 1.37e-46 | 2.244e-45 | SACM1L |
| ENSG00000211584 | -1.627 | 7.604e-43 | 1.105e-41 | SLC48A1 |
| ENSG00000212123 | -1.267 | 0.006907 | 0.01227 | PRR22 |
| ENSG00000212673 | 1.363 | 0.0006234 | 0.001267 | AL136115.1 |
| ENSG00000212719 | -1.156 | 4.155e-09 | 1.312e-08 | C17orf51 |
| ENSG00000212724 | 2.37 | 0.0001568 | 0.0003391 | KRTAP2-3 |
| ENSG00000212747 | -3.246 | 3.264e-24 | 2.285e-23 | FAM127C |
| ENSG00000212864 | -3.53 | 2.023e-32 | 2.009e-31 | RNF208 |
| ENSG00000212916 | -3.567 | 4.806e-37 | 5.747e-36 | MAP10 |
| ENSG00000212997 | 2.731 | 6.79e-05 | 0.0001524 | AC023632.1 |
| ENSG00000212998 | 1.961 | 1.37e-15 | 6.348e-15 | RBM12B-AS1 |
| ENSG00000213015 | 1.544 | 1.333e-44 | 2.054e-43 | ZNF580 |
| ENSG00000213024 | -1.469 | 3.517e-50 | 6.368e-49 | NUP62 |
| ENSG00000213047 | -1.037 | 3.1e-15 | 1.413e-14 | DENND1B |
| ENSG00000213066 | -1.781 | 7.07e-44 | 1.067e-42 | FGFR1OP |
| ENSG00000213123 | 4.283 | 8.655e-06 | 2.104e-05 | TCTEX1D2 |
| ENSG00000213139 | -2.175 | 6.408e-06 | 1.576e-05 | CRYGS |
| ENSG00000213160 | -2.882 | 4.964e-59 | 1.156e-57 | KLHL23 |
| ENSG00000213185 | -1.136 | 0.002846 | 0.00533 | FAM24B |
| ENSG00000213186 | -2.236 | 9.017e-24 | 6.188e-23 | TRIM59 |
| ENSG00000213190 | 1.307 | 4.604e-22 | 2.916e-21 | MLLT11 |
| ENSG00000213246 | 1.085 | 1.074e-31 | 1.035e-30 | SUPT4H1 |
| ENSG00000213337 | -1.285 | 2.877e-11 | 1.051e-10 | ANKRD39 |
| ENSG00000213339 | -1.3 | 7.383e-21 | 4.428e-20 | QTRT1 |
| ENSG00000213380 | -1.615 | 3.915e-36 | 4.514e-35 | COG8 |
| ENSG00000213445 | -1.223 | 2.501e-09 | 8.025e-09 | SIPA1 |
| ENSG00000213465 | -1.073 | 4.569e-15 | 2.068e-14 | ARL2 |
| ENSG00000213471 | 2.098 | 0.004866 | 0.008822 | TTLL13 |
| ENSG00000213516 | -1.409 | 3.148e-13 | 1.292e-12 | RBMXL1 |
| ENSG00000213533 | -2.045 | 0.0225 | 0.03674 | TMEM110 |
| ENSG00000213551 | -2.02 | 1.233e-70 | 4.018e-69 | DNAJC9 |
| ENSG00000213563 | -1.404 | 1.105e-10 | 3.888e-10 | C8orf82 |
| ENSG00000213588 | -2.54 | 7.153e-57 | 1.553e-55 | ZBTB9 |
| ENSG00000213626 | 2 | 1.385e-06 | 3.61e-06 | LBH |
| ENSG00000213638 | -1.172 | 0.00127 | 0.00249 | ADAT3 |
| ENSG00000213658 | 1.581 | 1.099e-05 | 2.648e-05 | LAT |
| ENSG00000213672 | -2.545 | 6.2e-43 | 9.033e-42 | NCKIPSD |
| ENSG00000213689 | -1.672 | 8.058e-06 | 1.964e-05 | TREX1 |
| ENSG00000213694 | -1.289 | 7.564e-19 | 4.13e-18 | S1PR3 |
| ENSG00000213699 | -1.063 | 7.83e-24 | 5.384e-23 | SLC35F6 |
| ENSG00000213760 | 4.242 | 3.762e-17 | 1.89e-16 | ATP6V1G2 |
| ENSG00000213762 | -1.729 | 0.01188 | 0.02034 | ZNF134 |
| ENSG00000213780 | -2.144 | 1.458e-09 | 4.762e-09 | GTF2H4 |
| ENSG00000213853 | -1.355 | 1.304e-24 | 9.301e-24 | EMP2 |
| ENSG00000213888 | -3.051 | 7.268e-05 | 0.0001627 | AC005003.1 |
| ENSG00000213920 | -1.011 | 0.02339 | 0.03809 | MDP1 |
| ENSG00000213922 | 3.793 | 0.0007195 | 0.001448 | AC011500.1 |
| ENSG00000213928 | -1.841 | 4.736e-12 | 1.813e-11 | IRF9 |
| ENSG00000213937 | -4.368 | 7.361e-62 | 1.901e-60 | CLDN9 |
| ENSG00000213949 | 1.268 | 8.933e-11 | 3.156e-10 | ITGA1 |
| ENSG00000213965 | -2.605 | 2.493e-65 | 7.165e-64 | NUDT19 |
| ENSG00000213977 | -1.102 | 2.304e-09 | 7.408e-09 | TAX1BP3 |
| ENSG00000214021 | 2.415 | 2.623e-64 | 7.29e-63 | TTLL3 |
| ENSG00000214022 | -2.175 | 2.709e-59 | 6.433e-58 | REPIN1 |
| ENSG00000214029 | -2.266 | 5.698e-09 | 1.783e-08 | ZNF891 |
| ENSG00000214050 | -1.896 | 2.754e-07 | 7.621e-07 | FBXO16 |
| ENSG00000214063 | -1 | 1.223e-05 | 2.931e-05 | TSPAN4 |
| ENSG00000214102 | 2.56 | 0.02048 | 0.03372 | WEE2 |
| ENSG00000214106 | -1.149 | 4.415e-05 | 0.0001009 | PAXIP1-AS2 |
| ENSG00000214194 | -1.459 | 4.155e-11 | 1.501e-10 | LINC00998 |
| ENSG00000214226 | 1.514 | 2.601e-07 | 7.209e-07 | C17orf67 |
| ENSG00000214300 | -1.474 | 0.003479 | 0.006433 | SPDYE3 |
| ENSG00000214309 | -1.438 | 0.01404 | 0.02378 | MBLAC1 |
| ENSG00000214338 | 1.884 | 1.585e-05 | 3.762e-05 | SOGA3 |
| ENSG00000214357 | -4.711 | 6.298e-85 | 2.961e-83 | NEURL1B |
| ENSG00000214376 | -2.499 | 0.0005592 | 0.001142 | VSTM5 |
| ENSG00000214402 | 3.157 | 0.00986 | 0.0171 | LCNL1 |
| ENSG00000214413 | 1.535 | 3.791e-16 | 1.812e-15 | BBIP1 |
| ENSG00000214491 | 1.694 | 0.02345 | 0.03817 | SEC14L6 |
| ENSG00000214753 | -2.11 | 5.499e-57 | 1.203e-55 | HNRNPUL2 |
| ENSG00000214756 | 1.107 | 2.555e-11 | 9.377e-11 | METTL12 |
| ENSG00000214827 | -2.007 | 8.682e-13 | 3.474e-12 | MTCP1 |
| ENSG00000214860 | -1.367 | 0.0002274 | 0.0004841 | EVPLL |
| ENSG00000214941 | -1.016 | 4.15e-06 | 1.038e-05 | ZSWIM7 |
| ENSG00000214944 | -1.507 | 3.741e-71 | 1.234e-69 | ARHGEF28 |
| ENSG00000215012 | -3.885 | 1.312e-91 | 7.22e-90 | C22orf29 |
| ENSG00000215033 | -1.823 | 4.503e-20 | 2.603e-19 | AL603965.1 |
| ENSG00000215041 | -1.814 | 3.521e-24 | 2.46e-23 | NEURL4 |
| ENSG00000215186 | 4.753 | 7.956e-06 | 1.941e-05 | GOLGA6B |
| ENSG00000215193 | -1.341 | 2.042e-30 | 1.852e-29 | PEX26 |
| ENSG00000215252 | -1.067 | 1.356e-09 | 4.44e-09 | GOLGA8B |
| ENSG00000215271 | -2.171 | 1.48e-22 | 9.58e-22 | HOMEZ |
| ENSG00000215343 | 4.542 | 1.948e-05 | 4.592e-05 | ZNF705D |
| ENSG00000215356 | 4.333 | 3.81e-05 | 8.755e-05 | ZNF705B |
| ENSG00000215405 | 3.633 | 0.001959 | 0.003761 | GOLGA6L6 |
| ENSG00000215440 | -1.288 | 1.713e-12 | 6.725e-12 | NPEPL1 |
| ENSG00000215621 | -2.01 | 0.004207 | 0.007696 | AC174470.1 |
| ENSG00000215712 | -1.047 | 1.643e-05 | 3.897e-05 | TMEM242 |
| ENSG00000215717 | -1.382 | 2.186e-20 | 1.284e-19 | TMEM167B |
| ENSG00000215784 | -2.321 | 9.384e-31 | 8.665e-30 | FAM72D |
| ENSG00000215790 | -1.914 | 6.744e-07 | 1.806e-06 | SLC35E2 |
| ENSG00000215883 | -1.597 | 3.133e-13 | 1.286e-12 | CYB5RL |
| ENSG00000215912 | 2.912 | 0.004963 | 0.008991 | TTC34 |
| ENSG00000216490 | -1.312 | 1.341e-10 | 4.688e-10 | IFI30 |
| ENSG00000216895 | -1.315 | 1.321e-05 | 3.158e-05 | AC009403.2 |
| ENSG00000217128 | 2.058 | 2.147e-48 | 3.685e-47 | FNIP1 |
| ENSG00000217555 | -1.121 | 1.426e-05 | 3.398e-05 | CKLF |
| ENSG00000217930 | -1.048 | 6.573e-08 | 1.902e-07 | PAM16 |
| ENSG00000218819 | -1.508 | 2.85e-09 | 9.11e-09 | TDRD15 |
| ENSG00000218891 | -1.231 | 2.536e-08 | 7.566e-08 | ZNF579 |
| ENSG00000219481 | -1.191 | 2.636e-23 | 1.765e-22 | NBPF1 |
| ENSG00000220201 | -1.757 | 0.02971 | 0.04747 | ZGLP1 |
| ENSG00000221829 | -1.121 | 9.624e-12 | 3.617e-11 | FANCG |
| ENSG00000221838 | -1.873 | 8.282e-21 | 4.958e-20 | AP4M1 |
| ENSG00000221843 | -1.523 | 0.001106 | 0.002185 | C2orf16 |
| ENSG00000221866 | -2.462 | 6.508e-07 | 1.744e-06 | PLXNA4 |
| ENSG00000221869 | -2.4 | 8.97e-08 | 2.573e-07 | CEBPD |
| ENSG00000221883 | 3.161 | 7.918e-14 | 3.363e-13 | ARIH2OS |
| ENSG00000221886 | -4.141 | 9.032e-29 | 7.712e-28 | C5orf54 |
| ENSG00000221890 | -3.33 | 1.375e-24 | 9.793e-24 | NPTXR |
| ENSG00000221914 | 1.27 | 7.316e-36 | 8.326e-35 | PPP2R2A |
| ENSG00000221916 | -2.564 | 0.02887 | 0.04623 | C19orf73 |
| ENSG00000221944 | -1.885 | 1.129e-23 | 7.701e-23 | TIGD1 |
| ENSG00000221978 | -1.269 | 7.575e-16 | 3.556e-15 | CCNL2 |
| ENSG00000221988 | -1.435 | 9.19e-07 | 2.435e-06 | PPT2 |
| ENSG00000222009 | 4.079 | 4.903e-41 | 6.752e-40 | BTBD19 |
| ENSG00000222040 | 7.236 | 6.365e-26 | 4.826e-25 | ADRA2B |
| ENSG00000222046 | 2.447 | 0.001876 | 0.003609 | DCDC2B |
| ENSG00000223569 | 3.415 | 0.003759 | 0.006923 | USP17L15 |
| ENSG00000223638 | 7.52 | 1.061e-17 | 5.477e-17 | RFPL4A |
| ENSG00000224051 | -2.81 | 4.484e-19 | 2.475e-18 | GLTPD1 |
| ENSG00000224361 | 3.035 | 0.01956 | 0.03231 | AC011239.1 |
| ENSG00000224470 | 1.099 | 1.423e-25 | 1.064e-24 | ATXN1L |
| ENSG00000224531 | -1.171 | 5.482e-17 | 2.731e-16 | SMIM13 |
| ENSG00000224870 | -1.485 | 2.89e-19 | 1.61e-18 | RP4-758J18.2 |
| ENSG00000224877 | -1.342 | 1.496e-06 | 3.886e-06 | C17orf89 |
| ENSG00000224940 | -4.696 | 1.007e-06 | 2.661e-06 | PRRT4 |
| ENSG00000224997 | 1.906 | 2.215e-22 | 1.423e-21 | AL049840.1 |
| ENSG00000225119 | -1.407 | 0.002231 | 0.004246 | LINC00999 |
| ENSG00000225485 | -1.289 | 4.042e-23 | 2.681e-22 | ARHGAP23 |
| ENSG00000225526 | -1.759 | 4.372e-05 | 9.999e-05 | C3orf83 |
| ENSG00000225556 | -2.683 | 0.02091 | 0.03435 | C2CD4D |
| ENSG00000225899 | 3.303 | 0.008789 | 0.01535 | FRG2B |
| ENSG00000225996 | -4.087 | 0.001698 | 0.003282 | AL356356.1 |
| ENSG00000226124 | -1.758 | 0.01596 | 0.02677 | FTCDNL1 |
| ENSG00000226180 | 2.364 | 4.155e-19 | 2.299e-18 | AC010536.1 |
| ENSG00000226763 | 3.149 | 1.173e-21 | 7.327e-21 | SRRM5 |
| ENSG00000226777 | -2.099 | 0.0006657 | 0.001347 | KIAA0125 |
| ENSG00000227234 | 4.494 | 8.661e-37 | 1.024e-35 | SPANXB2 |
| ENSG00000227500 | -1.346 | 7.475e-24 | 5.145e-23 | SCAMP4 |
| ENSG00000228300 | -1.081 | 1.075e-16 | 5.27e-16 | C19orf24 |
| ENSG00000228570 | 1.373 | 0.0004339 | 0.0008962 | NUTM2E |
| ENSG00000228594 | -3.632 | 3.527e-17 | 1.775e-16 | C1orf233 |
| ENSG00000228696 | -1.341 | 5.328e-13 | 2.157e-12 | ARL17B |
| ENSG00000228727 | -3.011 | 6.151e-07 | 1.652e-06 | SAPCD1 |
| ENSG00000228804 | 3.498 | 0.0009109 | 0.001814 | RP11-211G3.3 |
| ENSG00000229292 | 4.986 | 7.036e-07 | 1.881e-06 | RFPL4AL1 |
| ENSG00000229544 | -2.565 | 6.727e-05 | 0.0001511 | NKX1-2 |
| ENSG00000229644 | 1.155 | 1.763e-09 | 5.721e-09 | NAMPTL |
| ENSG00000229729 | 2.114 | 1.717e-14 | 7.538e-14 | RP11-159G9.5 |
| ENSG00000230055 | -1.216 | 8.949e-09 | 2.756e-08 | CISD3 |
| ENSG00000230453 | -2.104 | 1.848e-23 | 1.247e-22 | ANKRD18B |
| ENSG00000230510 | 2.238 | 6.088e-08 | 1.766e-07 | PPP5D1 |
| ENSG00000230567 | -2.603 | 2.715e-55 | 5.613e-54 | FAM203B |
| ENSG00000231738 | 3.443 | 0.005152 | 0.009314 | TSPAN19 |
| ENSG00000231861 | 3.63 | 0.0001227 | 0.0002682 | OR5K2 |
| ENSG00000231924 | 1.522 | 0.006532 | 0.01164 | PSG1 |
| ENSG00000231925 | -1.017 | 3.285e-16 | 1.575e-15 | TAPBP |
| ENSG00000232196 | 4.325 | 4.953e-14 | 2.125e-13 | MTRNR2L4 |
| ENSG00000232653 | -2.5 | 0.0004256 | 0.0008802 | GOLGA8N |
| ENSG00000232774 | 1.213 | 3.689e-07 | 1.011e-06 | RP11-47I22.3 |
| ENSG00000232810 | -1.452 | 0.01078 | 0.01859 | TNF |
| ENSG00000232838 | -4.667 | 8.336e-40 | 1.099e-38 | PET117 |
| ENSG00000232859 | -2.695 | 0.0001062 | 0.0002344 | LYRM9 |
| ENSG00000233136 | 2.698 | 0.02049 | 0.03374 | USP17L11 |
| ENSG00000233198 | -2.106 | 0.0002197 | 0.0004688 | RNF224 |
| ENSG00000233232 | 1.82 | 0.001701 | 0.003286 | NPIPB7 |
| ENSG00000233280 | -2.361 | 4.008e-30 | 3.594e-29 | CRYBG3 |
| ENSG00000233404 | -1.952 | 0.02728 | 0.04391 | FLJ20373 |
| ENSG00000233436 | 2.01 | 0.00367 | 0.00677 | BTBD18 |
| ENSG00000233608 | -2.142 | 1.654e-07 | 4.655e-07 | TWIST2 |
| ENSG00000234127 | 1.388 | 8.995e-51 | 1.65e-49 | TRIM26 |
| ENSG00000234602 | -1.837 | 2.485e-05 | 5.8e-05 | MCIDAS |
| ENSG00000234719 | 1.33 | 7.587e-07 | 2.024e-06 | RP11-166B2.1 |
| ENSG00000234769 | 1.268 | 3.104e-10 | 1.06e-09 | WASH4P |
| ENSG00000234776 | 3.481 | 0.002097 | 0.00401 | C11orf94 |
| ENSG00000235109 | 1.585 | 5.857e-17 | 2.911e-16 | ZSCAN31 |
| ENSG00000235156 | 3.27 | 0.01154 | 0.0198 | TMEM30C |
| ENSG00000235173 | -2.912 | 6.918e-47 | 1.146e-45 | FAM203A |
| ENSG00000235604 | 4.448 | 9.791e-37 | 1.151e-35 | SPANXB1 |
| ENSG00000235863 | -1.697 | 0.0002281 | 0.0004855 | B3GALT4 |
| ENSG00000236287 | -1.454 | 1.021e-28 | 8.696e-28 | ZBED5 |
| ENSG00000236669 | 4.042 | 0.0005823 | 0.001187 | AC006372.1 |
| ENSG00000237190 | -1.434 | 4.534e-10 | 1.531e-09 | CDKN2AIPNL |
| ENSG00000237330 | 2.3 | 1.005e-20 | 5.991e-20 | RNF223 |
| ENSG00000237515 | -1.887 | 2.483e-26 | 1.914e-25 | SHISA9 |
| ENSG00000237649 | -3.441 | 7.978e-178 | 1.886e-175 | KIFC1 |
| ENSG00000237651 | -1.541 | 6.751e-05 | 0.0001515 | C2orf74 |
| ENSG00000237693 | 2.973 | 0.001855 | 0.003573 | IRGM |
| ENSG00000238227 | -3.32 | 1.799e-111 | 1.431e-109 | C9orf69 |
| ENSG00000239305 | 1.201 | 1.409e-31 | 1.35e-30 | RNF103 |
| ENSG00000239306 | -1.84 | 6.004e-68 | 1.842e-66 | RBM14 |
| ENSG00000239388 | -2.072 | 0.001269 | 0.00249 | ASB14 |
| ENSG00000239789 | -1.18 | 8.706e-10 | 2.889e-09 | MRPS17 |
| ENSG00000240184 | -2.047 | 4.107e-15 | 1.863e-14 | PCDHGC3 |
| ENSG00000240204 | -2.755 | 8.293e-16 | 3.889e-15 | SMKR1 |
| ENSG00000240303 | -2.021 | 1.501e-36 | 1.747e-35 | ACAD11 |
| ENSG00000240563 | 2.417 | 0.01268 | 0.02163 | L1TD1 |
| ENSG00000240583 | -1.529 | 0.01895 | 0.03137 | AQP1 |
| ENSG00000240682 | 1.001 | 4.738e-07 | 1.286e-06 | ISY1 |
| ENSG00000240694 | -1.745 | 0.01012 | 0.01753 | PNMA2 |
| ENSG00000240771 | -2.037 | 8.004e-16 | 3.755e-15 | ARHGEF25 |
| ENSG00000240857 | -1.74 | 2.469e-27 | 1.979e-26 | RDH14 |
| ENSG00000241127 | -1.457 | 6.953e-08 | 2.01e-07 | YAE1D1 |
| ENSG00000241360 | -4.998 | 8.41e-13 | 3.367e-12 | PDXP |
| ENSG00000241404 | -1.593 | 7.695e-09 | 2.388e-08 | EGFL8 |
| ENSG00000241489 | 1.191 | 0.0001856 | 0.0003987 | IDS |
| ENSG00000241935 | -2.726 | 4.744e-06 | 1.179e-05 | HOGA1 |
| ENSG00000241945 | -1.202 | 1.313e-15 | 6.099e-15 | PWP2 |
| ENSG00000242110 | -3.497 | 1.108e-75 | 4.105e-74 | AMACR |
| ENSG00000242114 | -3.401 | 2.291e-41 | 3.187e-40 | MTFP1 |
| ENSG00000242259 | -1.489 | 4.93e-10 | 1.66e-09 | C22orf39 |
| ENSG00000242498 | -1.501 | 1.401e-32 | 1.401e-31 | C15orf38 |
| ENSG00000242574 | 3.034 | 0.003106 | 0.005789 | HLA-DMB |
| ENSG00000242612 | -1.178 | 1.946e-10 | 6.728e-10 | DECR2 |
| ENSG00000242802 | 2.267 | 2.855e-62 | 7.512e-61 | AP5Z1 |
| ENSG00000242866 | 2.2 | 0.001951 | 0.003746 | STRC |
| ENSG00000242950 | 2.328 | 0.02018 | 0.03327 | ERVW-1 |
| ENSG00000243232 | -4.527 | 3.038e-15 | 1.385e-14 | PCDHAC2 |
| ENSG00000243251 | -2.593 | 7.241e-18 | 3.773e-17 | PGBD3 |
| ENSG00000243335 | -1.837 | 1.564e-16 | 7.598e-16 | KCTD7 |
| ENSG00000243364 | -1.789 | 1.258e-08 | 3.831e-08 | EFNA4 |
| ENSG00000243477 | -1.945 | 1.304e-06 | 3.408e-06 | NAT6 |
| ENSG00000243566 | -1.351 | 2.105e-05 | 4.945e-05 | UPK3B |
| ENSG00000243660 | -1.148 | 5.349e-06 | 1.324e-05 | ZNF487 |
| ENSG00000243667 | -1.19 | 5.681e-17 | 2.828e-16 | WDR92 |
| ENSG00000243811 | -3.552 | 0.0001708 | 0.000368 | APOBEC3D |
[truncated: 13,506 more chars]
